# Gas Phase Conformation of Trisaccharides and Core Pentasaccharide: A Three-Step Tree-Based Sampling and Quantum Mechanical Computational Approach

**DOI:** 10.3390/molecules28248093

**Published:** 2023-12-14

**Authors:** Dong Chen, Jianming Gao, Danting Zheng, Zhiheng Guo, Zuncheng Zhao

**Affiliations:** 1 School of Physics and Electronics, Henan University, Kaifeng 475004, China; henu202309@163.com (J.G.); zdt184@163.com (D.Z.); guozh@henu.edu.cn (Z.G.); 2 Henan Province Engineering Research Center of Metal Matrix in situ Composites Based on Aluminum, Magnesium or Copper, Henan University, Kaifeng 475004, China

**Keywords:** trisaccharide, core pentasaccharide, IR vibration signature, three-step tree-based sampling

## Abstract

As an important component of N-linked glycoproteins, the core pentasaccharide is highly crucial to the potential application prospect of glycoprotein. However, the gas phase conformation study is a challenging one due to the size and complexity of the molecule, together with the necessity to rely on quantum chemistry modeling for relevant energetics and structures. In this paper, the structures of the trisaccharides and core pentasaccharides in N-linked glycans in the gas phase were constructed by a three-step tree-based (TSTB) sampling. Since single point energies of all the conformers are calculated at the temperature of zero, it is necessary to evaluate the stability at a high temperature. We calculate the Gibbs free energies using the standard thermochemistry model (T = 298.15 K). For trimannose, the energetic ordering at 298.15 K can be strongly changed compared to 0 K. Moreover, two structures of trimannose with high energies at 0 K are considered to provide a much better match of IR vibration signatures with the low Gibbs free energies. On this basis, the core pentasaccharide was constructed in three ways. The building configurations of core pentasaccharide were optimized to obtain reasonable low-energy stable conformers. Fortunately, the lowest-energy structure of core pentasaccharide is eventually the minimum at 0 K and 298.15 K. Furthermore, spectrum analysis of core pentasaccharide was carried out. Although poorly resolved, its contour from the experiment was in qualitative correspondence with the computed IR spectrum associated with its minimum free energy structure. A large number of strongly and weakly hydrogen-bonded hydroxyl and acetylamino groups contribute to a highly congested set of overlapping bands. Compared with traditional conformation generators, the TSTB sampling is employed to efficiently and comprehensively obtain preferred conformers of larger saccharides with lower energy.

## 1. Introduction

As important organic compounds, carbohydrates are widely found in nature [1,2,3,4]. The carbon backbone in carbohydrate molecules is directly or indirectly converted into various organic molecules such as proteins, nucleic acids, and lipids [5,6,7,8,9]. Carbohydrate molecules play an essential role in maintaining the biological activity of substances and achieving their functions in glycoproteins [10,11,12,13]. The study of carbohydrates is crucial to the potential application prospects of glycoproteins, but the diversity and instability of their structures seriously limit their application research. Experimentally, the configuration isomer is determined using the analysis of the mass spectrometry and the IR vibration spectrum data [14,15,16,17] or by means of gas chromatography-mass spectrometry measurement [18,19,20,21] that includes molecular configuration specificity. Recent infrared multiple photon dissociation (IRMPD) studies performed with enough structural resolution to identify closely related isomers [22]. The vibration signal of oligosaccharide molecules is provided, and the configuration information is assigned to the vibration peak of a specific structure [4,23,24] by combining with the results of *ab initio* calculations. A rotational spectroscopic study of erythrulose was obtained by the experiment, which enabled the determination of the equilibrium geometries together with quantum chemical calculations [25]. A combined experimental and theoretical investigation on 4-fluoro-threonine was carried out by Alonso et al. [26]. They developed a strategy employing machine learning to combine quantum chemistry and microwave spectroscopy, which can play an important role in studying the conformational energy as well as geometric and electronic structures of small molecules. The gas phase IRMPD spectra of protonated N-acetylated hexosamines and their methyl-blocked α and β anomers were obtained and compared with DFT simulation of the IR spectra [27].

In recent years, a significant increase in the development of computational methods has established many of the structural and dynamic features of complex carbohydrates. The generation of initial structures of oligosaccharides is primarily covered in the literature describing the methods commonly used: Quantum chemical methods and molecular simulation techniques [28]. Examples of Monte-Carlo Multiple Minimum (MCMM) conformational search with the Merk molecular force field static (MMFFs) can be used to generate the structures of the initial configurations [29,30]. However, the initial configurations generated using full-space conformational search are numerous [31,32,33,34], and configuration optimization takes a lot of time. In particular, further quantum chemical calculations cannot be completed when the configuration of the molecule is more complex, which brings a great challenge to obtain the lowest energy structures from traditional conformational search. Based on the above problems, we propose to use the three-step tree-based (TSTB) sampling [35,36] to study the preferred conformational structure of biomolecules, which is based on the analysis of molecular structural characteristics to construct the initial configuration of molecules and simplifies the calculation costs for a large number of repetitive conformers generated using the full-space conformational search. As an important bridge between proteins and polysaccharides [37,38], the core pentasaccharide is composed of three mannoses and two acetylglucosamines.

In this work, the low-energy structures of mannose diacetylglucose trisaccharide (Manβ(1,4)GlcNAcβ(1,4)GlcNAc) and trimannose (Manα(1,3)Manα(1,6)Man) in the gas phase were predicted using the TSTB sampling, and the results were compared with the theoretical and experimental results reported in the literature. According to the favorable conformers, the initial structure of core pentasaccharide was reasonably constructed. Further calculations were performed to obtain single-point energy and structure analysis. This work will introduce in detail the building process of complex molecular structures using the TSTB sampling so that readers can understand this method and apply it to different polysaccharide molecules, even peptide and protein structures. 

## 2. Results and Discussion

### 2.1. Construction of Manβ(1,4)GlcNAcβ(1,4)GlcNAc

**Figure 1:**
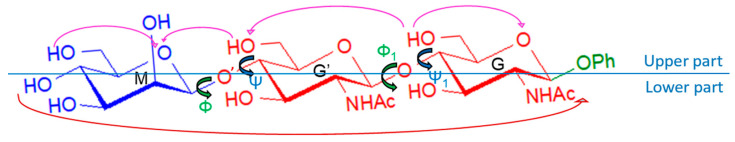
The structural formulas of Manβ(1,4)GlcNAcβ(1,4)GlcNAc. Here, glycosidic linkage torsion angles (Φ and ψ) have been used for distinguishing the structures of *cis* and *trans*. Φ and Ψ are the dihedral angles that rotate the glycosidic bond. Φ = OM-C1-O′-C4′, Ψ = C1-O′-C4′-C3′. Φ_1_ = OG′-C1′-O-C4, Ψ_1_ = C1′-O-C4-C3. The solid arrows refer to the possible orientations of the cooperative hydrogen bonding networks. The blue solid line is defined as a split line to separate the upper and lower parts.

**Figure 2:**
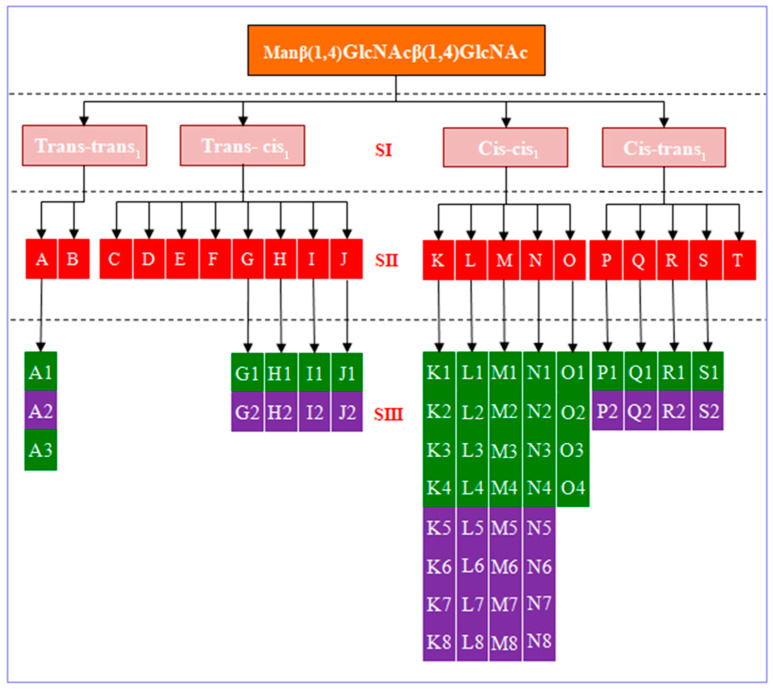
Building tree of Manβ(1,4)GlcNAcβ(1,4)GlcNAc. Regions SI, SII, and SIII reflect the constraint of glycosidic bond linkage, inter-ring H-bonds, and the orientation of intra-ring H-bonds, respectively. In region SIII, the directions of cooperative H-bonds counterclockwise and clockwise are indicated by green and purple colors, respectively.

Mannose diacetylglucose trisaccharide is formed by a trisaccharide chain of β-1,4-linked one mannose and two acetylaminose. The structural formula of Manβ(1,4)GlcNAcβ(1,4)GlcNAc is shown in Figure 1. The notations of the molecular groups mainly depend on the structural formula. As shown in Figure 1, three sugar rings are labeled as M, G′, and G. Corresponding molecular groups on each sugar ring are labeled. For example, OH6_M_ is the hydroxyl group at position C6 on ring M. OH6′, and OH6 refers to the hydroxyl groups at position C6′ and C6 on rings G′ and G, respectively. As shown in Figure 1, we connect the three oxygen atoms on the sugar rings (OM, OG′, and OG) as a split line. OM is labeled as the oxygen atom located on ring M of mannose. OG′ and OG are labeled as the oxygen atoms located on rings G′ and G of disaccharides with β-1,4-linked N-acetylglucosamine, respectively. The structure above the split line is defined as the upper part, and that below refers to the lower part. When hydrogen bonds (H-bonds) are properly considered in the building procedure, it is possible to construct all low-energy conformers easily and reasonably. Meanwhile, the combination of H-bonds in the upper and lower parts can effectively avoid conformer duplication. In Figure 2, we establish a tree diagram of Manβ(1,4)GlcNAcβ(1,4)GlcNAc. Region SI is classified according to *cis* and *trans* glycosidic bonds. *Cis* and *trans* conformations are defined as follows. For the glycosidic linkage, we only consider four cases: *cis* glycosidic linkage conformations for Manβ(1,4)GlcNAc with syn/syn (Φ, Ψ) ≈ −80°, 90° and *trans* glycosidic linkage conformations for Manβ(1,4)GlcNAc with anti/syn (Φ, Ψ) ≈ 50°, 120°; *cis*_1_ glycosidic linkage conformations for GlcNAcβ(1,4)GlcNAc with syn/syn (Φ_1_, Ψ_1_) ≈ −80°, 90° and *trans*_1_ glycosidic linkage conformations for GlcNAcβ(1,4)GlcNAc with anti/syn (Φ_1_, Ψ_1_) ≈ 50°, 120°. For example, a *trans*-*trans*_1_ conformation of Manβ(1,4)GlcNAcβ(1,4)GlcNAc is shown in Figure 1. Taking all four dihedral angles into account, four different forms are obtained for Manβ(1,4)GlcNAcβ(1,4)GlcNAc, already illustrating the structural variety of oligosaccharides. Region SII is constructed according to the structure of the H-bond. According to the H-bond types of each group of region SII, region SIII is combined by the upper and lower parts.

**Figure 3:**
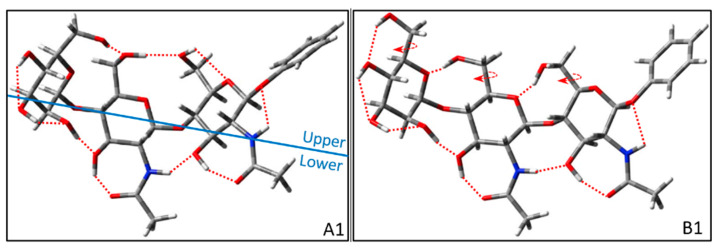
Representative configurations of *trans-trans*_1_ glycosidic linkage for Manβ(1,4)GlcNAcβ(1,4)GlcNAc before optimizations. The circular arrow in the figure represents the direction of the dihedral angle adjusted by rotation during construction, and the red dotted lines imply the possible H-bonds that are considered to be formed in the building process. In general, we define possible H-bonds according to minimum distances less than 2.8 Å. The blue solid line is defined as a split line to separate the upper and lower parts.

Next, we will illustrate the classification of each group configuration and the construction of the intra-group configuration in detail. Here, we build the basic skeleton of trisaccharides according to the position of glycosidic bonds and then construct the reasonable configurations of trisaccharides according to the H-bonds that could be formed. Among them, the skeleton of groups A and B is composed of two *trans*-glycosidic bonds. The upper and lower H-bonds of conformer A1 show cooperative inter-ring H-bonds clockwise and counterclockwise, respectively. The upper H-bond of A1 (see Figure 3 below) is OH6_M_→OH6′→OH6→OG, and the lower H-bond is OH4_M_→OH3_M_→OH2_M_→OH3′→NHCO′→OH3→NHCO→O1. Here, an arrow indicates the proton donor of the hydrogen atom directed toward the acceptor of the oxygen atom. While the upper H-bond of conformer A2 is OH6→OH6′→OH6_M_, which is the counterclockwise cooperative inter-ring H-bond in the upper part. The lower H-bond of conformer A2 is consistent with conformer A1. Conformer A3 is distinguished from conformer A1 by the clockwise and counterclockwise of the lower cooperative inter-ring H-bonds of OH3′→OH2_M_→OH3_M_→OH4_M_ and NHCO′→OH3→NHCO→O1. According to the building rules, there will be a conformer in group A with the upper H-bond of OH6→OH6′→OH6_M_ and the lower H-bond of OH3′→OH2_M_→OH3_M_→OH4_M_. However, the conformer will lead to high energy because OH6_M_ and OH4_M_ point to each other. Therefore, the conformer is not considered in group A. The difference between the configurations of groups B and A lies in the upper H-bonds. The upper part of group B does not have clockwise and counterclockwise cooperative inter-ring H-bonds. The upper H-bonds of conformer B1 (see Figure 3 below) are OH6→OG′, OH6′→OM and OH6_M_→OH4_M_, and the lower H-bond is OH4_M_→OH3_M_→OH2_M_→OH3′→NHCO′→OH3→NHCO→O1. Unfortunately, the clockwise cooperative inter-ring H-bonds of the lower part will lead to OH6_M_ and OH4_M_ pointing to each other. Therefore, there is only one conformer in group B. For Manβ(1,4)GlcNAcβ(1,4)GlcNAc of *trans-trans*_1_ glycosidic linkage, the conformers are classified according to two groups containing four conformers. Then, we will build the representative structures with *trans-cis*_1_ glycosidic linkage.

**Figure 4:**
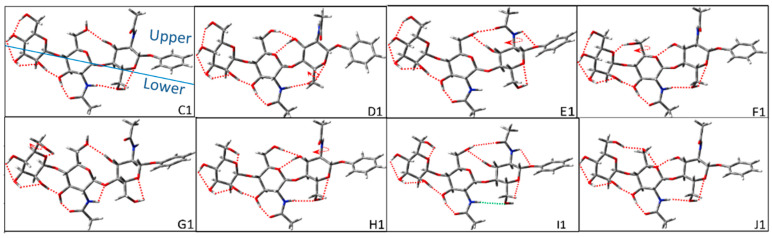
Representative configurations of *trans-cis*_1_ glycosidic linkage for Manβ(1,4)GlcNAcβ(1,4)GlcNAc before optimizations. The circular arrow in the figure represents the direction of the dihedral angle adjusted by rotation during construction, and the red dotted lines imply the possible H-bonds that were considered to be formed in the building process. The green dotted line indicates the disappearance of possible H-bond after the structural optimization. In general, we define possible H-bonds according to minimum distances of less than 2.8Å. The blue solid line is defined as a split line to separate the upper and lower parts.

The *trans-cis*_1_ glycosidic linkage of Manβ(1,4)GlcNAcβ(1,4)GlcNAc refers to the *trans* glycosidic linkage in Manβ(1,4)GlcNAc and the *cis* glycosidic linkage in GlcNAcβ(1,4)GlcNAc. As shown in Figure 4, we construct eight groups with the difference in the upper H-bond. For group C, the upper H-bonds are OH3→OH6′ and OH6_M_→OH4_M_, and the lower H-bond is OH4_M_→OH3_M_→OH2_M_→OH3′→NHCO′→OH6→OG. Because the lower clockwise cooperative inter-ring H-bond conflicts with the orientation of OH6_M_, there is only one conformer in group C. The upper H-bonds of conformer D1 are OH6′→OH3→OG′ and OH6_M_→OH4_M_. The upper H-bonds of conformer E1 are OH3→OH6′→NHCO→O1 and OH6_M_→OH4_M_. The upper H-bonds of conformer F1 are OH3→OG′, OH6′→OM and OH6_M_→OH4_M_. Their lower H-bonds are the same as conformer C1. Therefore, there is only one conformer in groups D, E, and F because the lower clockwise cooperative inter-ring H-bond conflicts with the orientation of OH6_M_.

As shown in Figure 4, the H-bond of NHCO′→OH6 in the lower part of conformer E1 is indicated by a green dotted line. After the structural optimization, the H-bond disappears due to the competition between the upper and lower H-bonds. The upper H-bonds of conformer G1 in group G are OH3→OH6′ and OH6_M_→OM. The counterclockwise cooperative H-bond of OH4_M_→OH3_M_→OH2_M_→OH3′→NHCO′→OH6→OG is built in the lower part. Correspondingly, the clockwise cooperative H-bond of conformer G2 forms OH3′→OH2_M_→OH3_M_→OH4_M_. The other H-bonds are the same as conformer G1. Therefore, Group G has two basic configurations. The upper H-bonds of conformer H1 are OH6′→OH3→OG′ and OH6_M_→OM. The upper H-bonds of conformer I1 are OH3→OH6′→NHCO→O1 and OH6_M_→OM. The upper H-bonds of conformer J1 are OH3→OG′ and OH6_M_→OH6′→OG′. For conformers H1, I1, and J1, their lower H-bond is the same as G1 with counterclockwise cooperative H-bond. Conformers H2, I2, and J2 have a clockwise cooperative H-bond, which is the same as conformer G2. Therefore, each group H, I, and J, has two configurations. The initial configurations of *trans-cis*_1_ glycosidic linkage are divided into eight groups with a total of 12 conformers.

**Figure 5:**
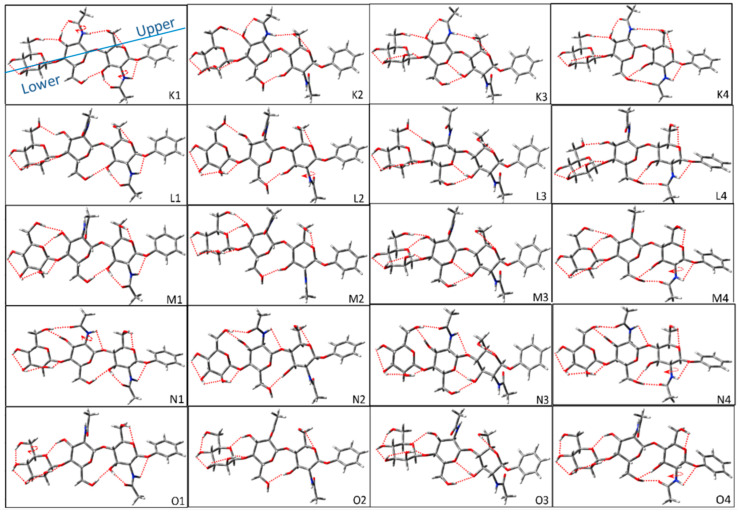
Representative configurations of *cis-cis*_1_ glycosidic linkage for Manβ(1,4)GlcNAcβ(1,4)GlcNAc before optimizations. The circular arrow in the figure represents the direction of the dihedral angle adjusted by rotation during construction, and the red dotted lines imply the possible H-bonds that were considered to be formed in the building process. In general, we define possible H-bonds according to minimum distances less than 2.8 Å. The blue solid line is defined as a split line to separate the upper and lower parts.

Figure 5 shows the representative configurations of *trans-trans* glycosidic linkage for Manβ(1,4)GlcNAcβ(1,4)GlcNAc. The structure difference for each group is the upper H-bond, and the difference for configurations in the same group relies on the lower H-bond. The upper H-bond of each configuration in the K group is OH6_M_→OH3′→NHCO′→OH6→OG, and the difference in the configuration in the group is that the lower H-bonds of OH4_M_→OH3_M_→OH2_M_→O′ and OH6′→OH3→NHCO→O1. Because the lower H-bonds of each configuration in the group have counterclockwise and clockwise cooperative H-bonds, there are eight configurations in the group K. In the same way, the upper H-bonds in groups L, M, and N are unchanged. The lower H-bonds have clockwise and counterclockwise cooperative H-bond directions. All three groups have eight configurations. All upper H-bonds of configurations are the same in-group O. Because the lower H-bonds of the M sugar ring in a clockwise direction conflict with the orientation of OH6_M_, there is only one counterclockwise cooperative H-bond direction. Therefore, group O contains four conformers.

**Figure 6:**
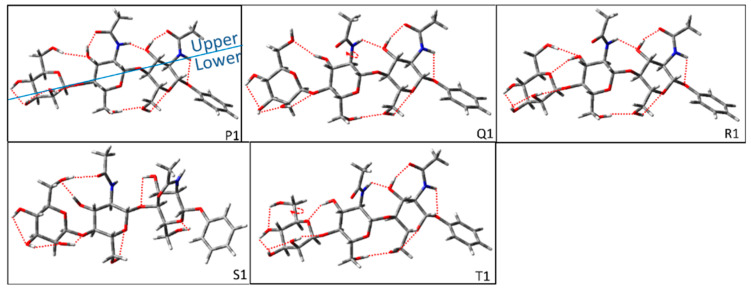
Representative configurations of *cis-trans*_1_ glycosidic linkage for Manβ(1,4)GlcNAcβ(1,4)GlcNAc before optimizations. The circular arrow in the figure represents the direction of the dihedral angle adjusted by rotation during construction, and the red dotted lines imply the possible H-bonds that are considered to be formed in the building process. In general, we define possible H-bonds according to minimum distances less than 2.8 Å. The blue solid line is defined as a split line to separate the upper and lower parts.

The initial structures of the *cis-trans*_1_ glycosidic linkage for Manβ(1,4)GlcNAcβ(1,4)GlcNAc are shown in Figure 6. The difference in various groups here is the upper H-bonds. The upper H-bond in group P is OH6_M_→OH3′→NHCO′→OH3→NHCO→O1. The upper H-bonds in group Q are OH3→OH6_M_ and NHCO′→OH3→NHCO→O1. The upper H-bonds in group R are OH6_M_→OH3′→OM and NHCO′→OH3→NHCO→O1. The upper H-bonds in group S are OH3′→OH6_M_→ NHCO′ and OH3→O. The upper H-bonds in group T are OH6_M_→OH4_M_, OH3′→OM, and NHCO′→OH3→NHCO→O1. The lower H-bonds of each group are OH4_M_→OH3_M_→OH2_M_→O′ and OH6′→OH6→OG. Due to the clockwise cooperative H-bonds of the M sugar ring in groups P, Q, R, and S, there are two conformers in each group. Since the clockwise cooperative H-bond in group T conflicts with the direction of OH6_M_, there is only one configuration in this group.

### 2.2. Construction of Manα(1,3)Manα(1,6)Man

The structural formula of Manα(1,3)Manα(1,6)Man is shown in Figure S1 in Supplementary Materials. The α-_D_-mannose structure contains four hydroxyl groups and one hydroxymethyl group. The hydroxyl groups at positions C1 and C2 are axial, which are perpendicular to the equatorial plane. The hydroxyl groups at positions C3 and C4 are equatorial. The orientation of the hydrogen atoms in these four hydroxyl groups could be any position so that the trimannose will have more spatial preference. The notations of the molecular groups mainly depend on the structural formulas. As shown in Figure S1, three sugar rings are labeled as M, M′ and M″. Corresponding molecular groups on each sugar ring are labeled. For example, OH6″ is the hydroxyl group at position C6 on ring M″.

Similar to the building process of Manβ(1,4)GlcNAcβ(1,4)GlcNAc, we still use the definition of *cis* and *trans-*glycosidic linkage to distinguish the glycosidic bonds between M and M″ rings of Manα(1,3)Manα(1,6)Man. The building tree is shown in Figure S2 in the Supplementary Materials, first determining the type of glycosidic linkage, then determining the type of H-bond, and finally selecting the appropriate cooperative H-bond direction.

Next, we will discuss the classification and the construction of configuration in each group in detail. As shown in Figure S3 in the Supplementary Materials, the skeleton of configuration in group A~D is composed of *cis-cis* glycosidic linkage, which is distinguished by the cooperative H-bonds. The cooperative H-bonds formed in conformer A1 are OH6″→OH4′→OH3′→OH2′, OH2″→OH3″→OH4″→OH6′→OH2→O and OH4→O′. Among them, the cooperative H-bonds of OH6″→OH4′→OH3′→OH2′ and OH2″→OH3″→OH4″→OH6′→OH2→O have clockwise and counterclockwise H-bond directions. Therefore, Group A has four representative structures. The difference between configurations in groups A and B is the cooperative H-bond of OH2′→OH3′→OH4′→OH6″→OM″. Referred to the H-bond of OH2′→OH3′→OH4′→OH6″→OM″, the counterclockwise cooperative H-bond is OH6″→OH4′→OH3′→OH2′, which will be consistent with group A. Therefore, there are only two conformers in group B. The inter-ring H-bonds in groups C and D are OH4″→OH4′ and OH3″→OH6′, which are different from the inter-ring H-bonds of OH4′→OH6″ and OH4″→OH6′ in groups A and B. The H-bonds of OH4″→OH4′→OH3′→OH2′ and OH2″→OH3″→OH6′→OH2→OM are clockwise and counterclockwise, which results in four conformers in group C. The only difference between groups D and C is the direction of the hydroxymethyl group on the M″ ring. Therefore, group D also has four configurations. Here, OH4′→OH4″ torsional flexibility of H-bonds between rings in groups C and D is not as flexible as OH4′→OH6″ containing H-bonds of hydroxymethyl group in groups A and B, which will make it difficult to achieve the equilibrium state for structural optimization and cause H-bond breakage.

We build the structures of *cis-trans* glycosidic linkage for Manα(1,3)Manα(1,6)Man, which are shown in Figure S4 in the Supplementary Materials. While keeping the glycosidic bond in *cis* formed by the M′ ring and the M ring unchanged, the glycosidic bond formed between M and M″ is rotated so that the glycosidic bond becomes a *trans* structure, and then the cooperative H-bond configuration is built. In group E, H-bonds of OH4″→OH3″→OH2″→OH6′→OH2→OM and OH4′→OH3′→OH2′ contain clockwise and counterclockwise H-bond directions. Group E has two configurations. Groups E and F are distinguished by hydroxymethyl orientation on the M″ ring. Therefore, group F also has two configurations.

The representative configurations of *trans-cis* glycosidic linkage for Manα(1,3)Manα(1,6)Man are shown in Figure S5 in the Supplementary Materials. In group G, the intra-ring H-bond of OH4′→OH3′→OH2′→OM′ on the M′ mannose ring and OH4″→OH3″→OH2″→OM″ on the M″ mannose ring, respectively. The inter-ring H-bond of OH4→OH6′→OH6″ can be formed by the rotation of the hydroxymethyl group. The difference between groups G, H, and I is the H-bonds direction that hydroxymethyl forms. The H-bond in group H is OH6′→OH6″→OM″, and the H-bond in group I is OH6′→OH4″→OH6″. Moreover, none of the H-bonds have clockwise and counterclockwise cooperative H-bond directions. Therefore, there is only one configuration in each group. On the basis of group G, we built the configurations of group J, which are conformers built by adjusting the glycosidic bond between M and M″ mannose rings to form more stable inter-ring H-bonds. The novel inter-ring H-bond of OH4″→OM′ of group J makes the structure between M′ and M″ sugar rings stronger. Considering that the H-bonds of OH4′→OH3′→OH2′→OM′ and OH4″→OM′ conflict in the direction and affect the stability of the inter-ring H-bond, the construction of conformer J2 changes the H-bond of OH4′→OH3′→OH2′→OM′ to OH2′→OH3′→OH4′. Thus, there are two conformers in group J. On the basis of the conformers in group J, group K is constructed by selecting the hydroxymethyl group on the M″ ring to form the H-bond of OH6′→OH6″→OM″. Similar to conformer J2, the H-bond direction of OH4′→OH3′→OH2′→OM′ changes to OH2′→OH3′→OH4′ forms conformer K2.

The configurations of the *trans-trans* glycosidic linkage for Manα(1,3) Manα(1,6)Man are constructed in Figure S6 in the Supplementary Materials. While keeping the glycosidic bond in *trans* formed by the M and the M′ ring unchanged, the glycosidic bond formed between M and M″ is rotated so that the glycosidic bond becomes a *trans* structure. Then, the inter-ring H-bond of OH2→OH6″ between M and M″ sugar rings is built in group L. Moreover, the hydroxymethyl group on the M″ ring can form another H-bond of OH6″→OH4″ in group L. In group M, the hydroxymethyl group on the M″ ring rotates to form the H-bond of OH6″→OM″. The H-bonds in each group especially do not have clockwise and counterclockwise cooperative H-bond directions. There is only one configuration in the group L and M.

### 2.3. Structural Optimization

Using the B3LYP/6-311+G* method of density functional theory, all initial construction structures are optimized. The relative energies of all configurations of trisaccharides are shown in Table S1 in the Supplementary Materials. After the optimization of Manβ(1,4)GlcNAcβ(1,4)GlcNAc, the H-bonds of four configurations change. The H-bond of NHCO′→OH6 in all configurations is broken due to the competition between H-bonds of OH3→OH6′→NHCO and NHCO′→OH6. After the structural optimization of Manα(1,3) Manα(1,6)Man, there were also four configurations in the broken H-bond. Because the inter-ring H-bond is formed by two hydroxyl groups with a small adjustable angle range, it was broken by the generation of new H-bonds during the configuration optimization.

After the analysis of all the optimized configurations, we find an interesting phenomenon that the type of H-bond formed in the low-energy configurations of Manβ(1,4)GlcNAcβ(1,4)GlcNAc is consistent with the type of H-bond in the low-energy configuration of the disaccharide in our previous work [36]. Therefore, when building a complex polysaccharide molecular structure, you can first separate the structure units of the polysaccharide, analyze the preferred structure units, and finally combine to build the favorable structures of the polysaccharide based on the formation of H-bond.

### 2.4. Analysis of Spectral Characteristics for Manβ(1,4)GlcNAcβ(1,4)GlcNAc and Manα(1,3) Manα(1,6)Man

**Figure 7:**
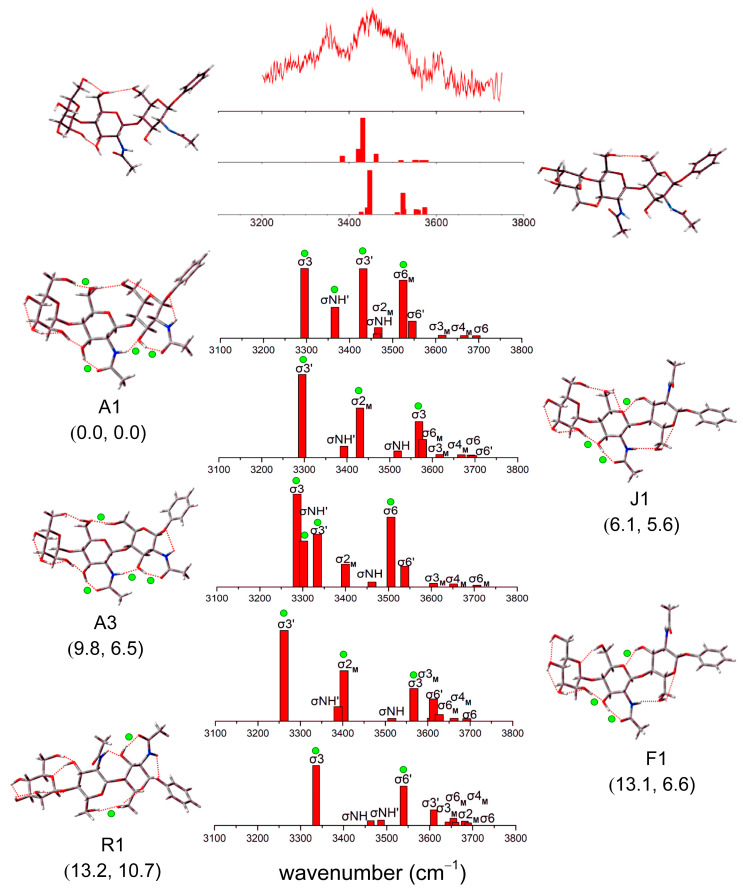
Comparison of experimental IR ion depletion spectrum and vibrational spectra for five conformers of Manβ(1,4)GlcNAcβ(1,4)GlcNAc. The relative energy (kJ/mol) and the five lowest-lying structures are shown, and several major infrared spectral vibration peaks are marked. Computed relative energies and Gibbs free energies (kJ/mol) are shown in the panel. The green dots identify the strong H-bonds in the structure and their corresponding features in the spectrum. The experimental and computational results of Manβ(1,4)GlcNAcβ(1,4)GlcNAc in Ref. [39] are shown at the top of the figure. Reproduced with permission from Ref. [39], American Chemical Society (ACS). Further permissions related to the material excerpted should be directed to the ACS.

Figure 7 shows the low-energy structures of Manβ(1,4)GlcNAcβ(1,4)GlcNAc within a relative energy of 15 kJ/mol, as well as the calculated IR spectra and characteristic peaks. It is worth noting that the Gibbs free energies at high temperatures possibly provide much better energetic estimates than 0 K [39]. Since energies of all the conformers are calculated at the temperature of zero, a discussion requires considering the Gibbs free energies using the standard thermochemistry model (T = 298.15 K) to evaluate the conformational stability at the typical room temperature. As you can see from Figure 7, the values of the Gibbs free energies are different from those at 0 K. Conformers F1 and R1 shown in Figure 7 are basically isoenergetic at 0 K, but there is a small difference in the Gibbs free energy of 4.1 kJ/mol. The reason for the discrepancy mainly relies on both enthalpic and entropic effects between conformations whose H-bonding contents are different. Fortunately, the energetic order at 298.15 K is unchanged compared to 0 K.

A detailed analysis of the IR spectra and comparisons with the experimental IRID spectrum and predictions of DFT calculations shown in the higher panel of Figure 7 are performed. The characteristic spectrum in our work is well consistent with the experimental value. In Figure 7, the experimental IR spectra of Manβ(1,4)GlcNAcβ(1,4)GlcNAc are mainly concentrated at ~3350 to ~3600 cm^−1^ with two strong peaks at ~3350 cm^−1^ and ~3450 cm^−1^. The majority of weak peaks crowd at higher wavenumbers, indicating the contribution from strongly and weakly hydrogen-bonded OH groups. In the experimental spectrum, the strong band at ~3450 cm^−1^ is related to the tensile vibration mode labeled σ3′ of the lowest-energy conformation in Figure 7. There are overlapping bands between ~3430 cm^−1^ and ~3520 cm^−1^, separated from the strong peaks at ~3350 cm^−1^, about 80 cm^−1^. The other strong, broad band at ~3350 cm^−1^ is accounted for by the σ3 vibration of conformer R1 with the Gibbs free energy of 10.7 kJ/mol. The assignments of the vibrations indicated the fact that high-temperature energetics are required in particular comparison to experimental data. These bands reflect the change of H-bonds in the isomer structures of Manβ(1,4)GlcNAcβ(1,4)GlcNAc to some extent. Compared with the lowest-energy conformer, the dihedral angles related to the glycosidic bond and the cooperative H-bonds in other conformers make the strength of H-bonds different, resulting in the corresponding red and blue shifts. In addition, the proximity of energy values and spectral congestion in the IR spectra can lead to difficulties in conformational assignment.

In Ref. [39], the initial structures were obtained by full-space conformational search, and then the low-energy configurations were selected for the optimization calculation using the M06-2X method. Similar to the lowest-energy conformer in our calculation, the two lowest-energy conformers are *trans-trans*_1_ structures. In order to assess the reasons for the discrepancy, optimized structures’ relative and free energies are calculated using DFT (M06-2X/6-31+G*). We build the starting structures of two lowest-energy conformers in Ref. [39], which can be found in Figure S7 in the Supplementary Materials. The correction factors of the frequency calculation are 0.9734 (OH stretch modes) and 0.9600 (NH stretch modes). The results are shown in Supporting Information. The calculations suggest that the lowest energy configuration we built is still the minimum at 298.15 K. When the molecular structure is complex, the number of full-space conformational searches will largely affect that of initial configurations, even missing important molecular configurations. When the number of full-space conformational searches is increased, a large number of initial configurations are obtained. It is possible to ignore some low-energy configurations in the calculation process by selecting some representative initial configurations. Clearly, the configurations obtained using the TSTB sampling can comprehensively and efficiently determine the low-energy configuration of complex molecules.

Among the low-energy configurations of Manα(1,3)Manα(1,6)Man, there are three low-energy configurations below 10 kJ/mol obtained by full-space conformational search and quantum chemical calculations in Ref. [37], while there are seven configurations with energies less than 10 kJ/mol established using the TSTB sampling. Since the energies of all the conformers in this paper are calculated at the temperature of zero, we also calculate the Gibbs free energies using the standard thermochemistry model (T = 298.15 K). Considering a 300 K stability, the energetic ordering can be strongly changed compared to 0 K. For example, conformer A1 is the lowest energy conformer at 0 K, but it is the second lowest one at 298.15 K. Conformer G1, which has a high relative energy of 20.1 kJ/mol at 0 K, turns to be the lowest conformer in Gibbs free energy. Conformer J2, the second lowest conformer in energy, ranks 5th lowest according to Gibbs free energy. In other words, at high temperatures, these conformers become a bit unstable. It is consistent with gas phase supersonic expansion experiments [40]. Due to the high *cis-trans* isomerization barrier being quite early inhibited along the expansion, it makes the observation of intrinsically unstable (at 0 K) forms provided possible because of entropic effects.

**Figure 8:**
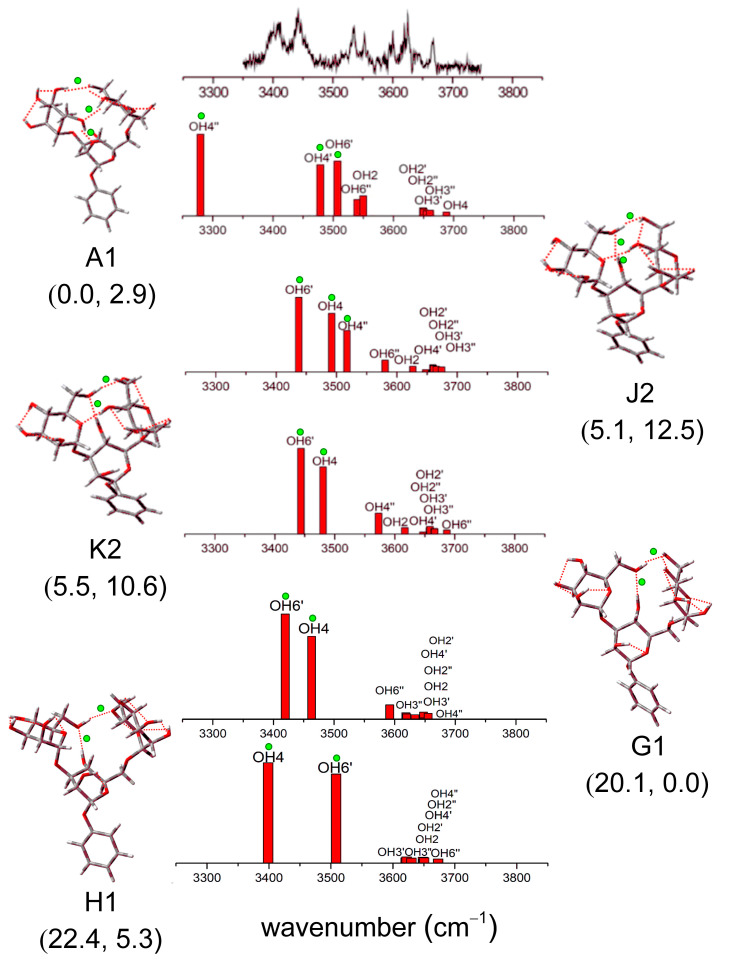
Comparison of vibration characteristics of Manα(1,3)Manα(1,6)Man. Five low-energy structures are shown, and several major infrared spectral vibration peaks are marked. Computed relative energies at 0 K and Gibbs free energies at 298.15 K, kJ/mol are shown in brackets. The green dots identify the strong H-bonds in the structure and their corresponding features in the spectrum. The experimental results of Manα(1,3)Manα(1,6)Man in Ref. [37] are shown at the top of the figure. Reproduced with permission from Ref. [37], American Chemical Society (ACS). Further permissions related to the material excerpted should be directed to the ACS.

The configurations within 6 kJ/mol and two conformers with relatively high energies are shown in Figure 8. As shown in Figure 8, two of the computed spectra are in remarkably good agreement with the experimental spectrum, which presents two intense, strongly displaced, and broadened bands at low wavenumbers, located at ~3400 cm^−1^ and ~3450 cm^−1^, together with a cluster of bands lying at higher wavenumbers. Although their associated structures do not correspond to the lowest relative energy, one of them possesses the minimum free energy. The conformation with the lowest Gibbs free energy is 20.1 kJ/mol higher in relative energy. Its calculated vibrational spectrum does reproduce the two strong bands located at ~3450 cm^−1^. The conformation with Gibbs free energy of 5.36 kJ/mol lies in 22.4 kJ/mol being higher in relative energy. The calculated vibrational spectrum located at ~3400 cm^−1^ does reproduce the experimental band at a lower wavenumber. Moreover, the lowest free energy configuration can well match the experimental spectrum, which indicates that the conformer dominates the structure of mannotriose. Other configurations reported in Ref. [37] are also predicted in our results. For other configurations, there are five low-energy configurations that are not reported in the literature.

### 2.5. Construction of Core Pentasaccharide

**Figure 9:**
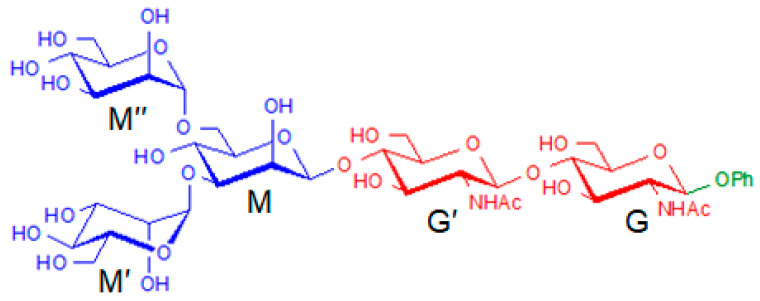
Schematic diagram of the molecular structure of core pentasaccharide. The blue part is the Manα(1,3)Manα(1,6)Man. The red part is GlcNAcβ(1,4)GlcNAc.

Considering our previous analysis of disaccharides and trisaccharides combined with the spatial structure of monosaccharides, we have a comprehensive understanding of the core pentasaccharide configuration. Figure 9 displays a schematic diagram of the molecular structure of core pentasaccharide. In order to build core pentasaccharide in detail, we use three ways to build core pentasaccharide. In the first way, the structure block of trisaccharide is used to build core pentasaccharide, which completely remains the cooperative inter-ring H-bonds in each block. However, duplicate monosaccharides will simultaneously exit H-bonds with both sides, which will make the building process difficult. In the second way, the core pentasaccharide is built by trisaccharides and disaccharides, which do not interfere with each other. The disadvantage is that H-bonds between disaccharides and trisaccharides may be ignored. In the third way, three monosaccharides and one disaccharide are used to build the core pentasaccharide. In this way, the formation of inter-ring H-bonds can be comprehensively considered. However, the construction process is complicated.

Firstly, we use the structure unit of trisaccharide to build the core pentasaccharide. For these two trisaccharides, the previous comprehensive analysis has been carried out. Here, we directly select the low-energy configurations within 10 kJ/mol to build core pentasaccharide. The main concern is the change of glycosidic bonds and inter-ring H-bonds. Changes in intra-ring H-bonds and the direction of cooperative H-bonds are no longer considered. Three and two lowest-energy configurations are chosen for Manα(1,3)Manα(1,6)Man and Manβ(1,4)GlcNAcβ(1,4)GlcNAc, respectively. Therefore, six pentasaccharide configurations are constructed after the combination of two trisaccharides. However, there are two problems with the repeated monosaccharide in the construction process. One is that OH2 on the M ring has two directions. When the monosaccharide belongs to Manα(1,3)Manα(1,6)Man, the H-bond formed by OH2 is OH2→OM. When the M ring belongs to Manβ(1,4)GlcNAcβ(1,4)GlcNAc, the H-bond formed by OH2 is OH2→OH3′. Considering the fullness of the initial structures in the building process, both conformers are constructed. However, the configuration with the H-bond of OH2→OM is repeated with the building configuration of the core pentasaccharide in the second way. The second issue is that OH6 on the M ring is the glycosidic bond of M and M″ on Manα(1,3)Manα(1,6)Man. Hydroxyl groups on the M and G′ rings form a H-bond of OH6→OH6′ in Manβ(1,4)GlcNAcβ(1,4)GlcNAc. Because OH6 on the M ring is formed by dehydration at the position of the glycosidic bond, the lack of hydrogen will no longer form the H-bond of OH6→OH6′. So only OH6 on the M ring belongs to Manα(1,3)Manα(1,6)Man is considered. In summary, six configurations of core pentasaccharide are actually constructed, which are shown in Figure S8 in the Supplementary Materials.

As shown in Figure 9, we use the structural unit of trisaccharide and disaccharide to build core pentasaccharide. According to the previous results, Manα(1,3)Manα(1,6)Man and GlcNAcβ(1,4)GlcNAc have three and two low-energy representative configurations within 10 kJ/mol, respectively. The combination results in a total of six initial configurations of core pentasaccharide. Considering that the glycosidic bond at the junction of trisaccharides and disaccharides will not affect their configuration, the glycosidic linkage is not limited to forming a new glycosidic bond type by rotation. Therefore, there will be two types of *cis* and *trans* glycosidic linkages. Actually, we have also built 12 configurations of core pentasaccharide. Representative configurations of *cis* glycosidic linkage for core pentasaccharide are shown in Figure S9 in the Supplementary Materials.

As shown in Figure 9, we have the structural unit of three monosaccharides and one disaccharide to build the core pentasaccharide. Disaccharide of GlcNAcβ(1,4)GlcNAc has two low-energy representative configurations. Considering that middle monosaccharides are linked to the sugar rings of upper and lower monosaccharides and disaccharides, the functional groups that can form H-bonds are severely limited. Four types are divided to describe the construction method for the core pentasaccharide. First of all, the upper monosaccharide can form H-bonds with disaccharides and middle monosaccharides. Secondly, the lower monosaccharides can form H-bonds with disaccharides and middle monosaccharides. A total of four groups are constructed. The specific structures are shown in Figure S10 in the Supplementary Materials. The construction method considers H-bonds between three monosaccharides, which transforms into the building problem of a mannotriose structure. It is equivalent to the construction method of trisaccharide and disaccharide.

**Figure 10:**
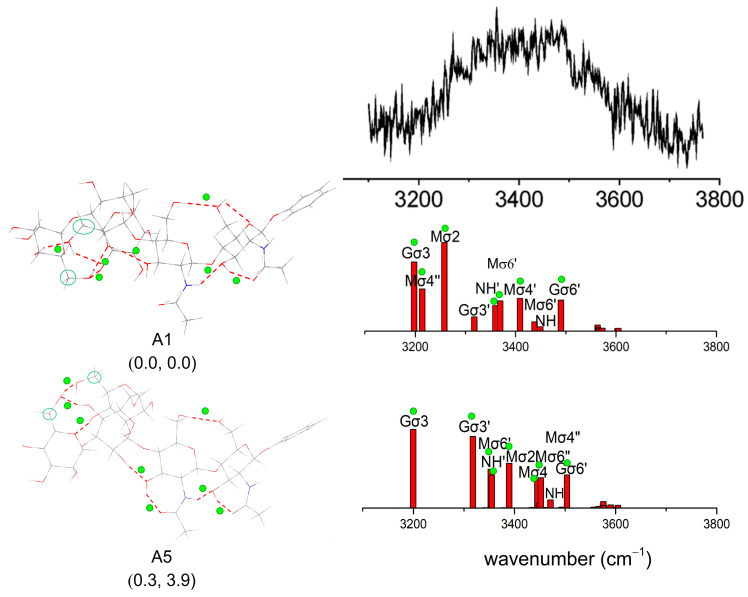
Comparison of vibration characteristics of core pentasaccharide. The lowest energy structures for relative energy (kJ/mol) are calculated as shown, and several major infrared spectral vibration peaks are labeled. The green dots identify the strong H-bonds in the structure and their corresponding features in the spectrum. The green circles indicate the rotation of the hydroxymethyl group Experimental results of core pentasaccharide in Ref. [39] are shown on the top of the panel. Reproduced with permission from Ref. [39], American Chemical Society (ACS). Further permissions related to the material excerpted should be directed to the ACS.

Since the pentasaccharide contains 14 hydroxy and 2 acetamido groups, structures that optimize global H-bonds of OH···O and NH···O are to be expected in the gas phase: not surprisingly, its IRID spectrum presents a broad red-shifted quasi-continuum, ranging from ~3100 to ~3700 cm^−1^. This suggests that a highly congested set of overlapping bands is associated with a large number of both strongly and weakly hydrogen-bonded OH and NHCO groups. Spectral congestion in the IRID spectrum could imply that the IR spectra are no longer conformer-selective and could actually be composite spectra made of several contributions. The vibration characteristics of core pentasaccharides are illustrated in Figure 10. The notations and labeling of the vibrational modes mainly depend on the structural formula. As shown in Figure 9, five sugar rings are labeled as M, M′, M″, G′, and G. The vibrational modes related to molecular groups on each sugar ring are labeled. For example, Gσ3 comes from the vibration of the hydroxyl group at position C3 on ring G.

After the initial configurations of core pentasaccharide are built, the optimized configurations are in good agreement with the initially expected structures. By good fortune, this structure of core pentasaccharide also has the lowest calculated relative energy (at 0 K and free energy at 298.15 K). Conformer A5, which has a slightly high relative energy of 0.3 kJ/mol^−1^, continues to be the second lowest conformer in the Gibbs free energy of 3.9 kJ/mol^−1^. Although poorly resolved, its contour from the experiment is in qualitative correspondence with the IR spectrum associated with its minimum free energy structure. Moreover, spectral congestion in the IRID spectrum could imply that the IR spectra are no longer conformer-selective and could be actually composite spectra made of several contributions.

Although the absorption band will displace due to the influence of chemical structure and external conditions, the band information, such as the absorption peak position, band intensity, band shape, and the presence of related peaks, can still comprehensively reflect the presence or absence of various functional groups. The vibration of the hydroxyl group is between ~3670 and ~3230 cm^−1^, and the vibration of the hydroxyl group in our calculation is ~3610 to ~3194 cm^−1^. The reason for the difference indicates the influence of H-bonds. The complex H-bonding network is often observed in a large size of the molecule. Additionally, the proximity of energy values and spectral congestion in the IR spectra can lead to difficulties in conformational assignment. Last but not least, due to the large size of the molecule and the difficulty of cooling it down during the supersonic expansion, the comparison with the experiment could imply that the IR spectra recorded are no longer conformer-selective and could be actually composite spectra made of several contributions.

The relative energies are shown in Table S1 in the Supplementary Materials. Finally, Cartesian coordinates of all optimized configurations are provided in the Supplementary Materials, which will give more details of geometric parameters for all the most stable structures.

## 3. Computational Methods

The initial structure of polysaccharides was constructed using the TSTB sampling considering the types of glycosidic linkage and H-bonds. As we all know, the structural motif can be attributed to a delicate balance among various interactions via covalent bonds, such as those H-bonds that determine their skeletal structures and the hydrogen bonding networks that are formed among multiple hydroxyl groups. A detailed investigation of the glycosidic linkage constraint and hydrogen bonding networks determining the structural preferences was conducted in our previous work [34]. The TSTB sampling has been successfully employed to build up reasonable starting structures manually so as to simplify the conformational determination. During the building processes, the main factors contain glycosidic linkage, inter-ring H-bonds, and cooperative intra-ring H-bonds. In general, glycosidic linkage dominates the skeletal structures of the saccharides, as well as the range of glycosidic dihedral angles. The species diversity in inter-ring H-bonds is highly restricted to the glycosidic linkage, and the structural stability depends to a great extent upon inter-ring H-bonds. Finally, intra-ring H-bonds can be arranged to form cooperative motifs with lower energies.

The initial structures of Manβ(1,4)GlcNAcβ(1,4)GlcNAc) and Manα(1,3)Manα(1,6)Man were optimized using the density functional B3LYP with 6-311+G* basis sets, and then the energy and frequency calculations were further performed. The single point energy was calculated using the two-order perturbation theory of MP2/6-311++G** and corrected by zero point energy using the frequency calculations at the B3LYP/6-311+G* basis sets. Theoretical harmonic IR spectra of these molecular structures are obtained with Density Functional Theory (DFT). In order to bring them into better accord with experimental spectra, a large number of empirical scaling factors have been used. The correction factors of the frequency calculation are 0.9734 (OH stretch modes) and 0.9600 (NH stretch modes) [39]. Due to the complex structure of core pentasaccharide, high-precision calculation is difficult to complete. In order to save calculation costs, we chose the B3LYP/6-31G* method to optimize the initial configurations. A correction factor of the frequency calculation is 0.9603 for B3LYP/6-31+G* [41]. The single point energy is calculated using the MP2/6-31++G** method. All calculations are carried out using the Gaussian 09 package [42].

## 4. Conclusions

In this paper, the structures of Manβ(1,4)GlcNAcβ(1,4)GlcNAc and Manα(1,3)Manα(1,6)Man were constructed using TSTB sampling, and their low-energy configurations were finally obtained after quantum chemical calculations. Their energies and spectral vibration characteristics were analyzed to obtain a reasonable structure. Based on the dihedral angles of glycosidic bonds, the H-bonds between rings, and the direction of cooperative H-bonds, the construction process in this paper introduced how to use the TSTB sampling to build polysaccharide structures in detail. Finally, the construction of complex core pentasaccharide structures was completed.

The low-energy configurations we obtained were in good agreement with the theoretical predictions and experimental values reported in the previous work. In the configuration of Manβ(1,4)GlcNAcβ(1,4)GlcNAc, the lowest-energy configuration was 16.2 kJ/mol, lower than the lowest energy in the literature. In these polysaccharide molecular configurations, we obtained some low-energy configurations that have not been reported previously. These low-energy configurations have significantly lower energies than the reported configurations due to the change of glycosidic bonds, inter-ring H-bonds, and intra-ring H-bonds. The reason why these configurations are not obtained in traditional full-space conformational search may be the lack of search times, the restrictions of the conditions set in the conformational search, or the artificial removal when selecting representative configurations. The results show that the TSTB sampling can accurately and comprehensively obtain the low-energy structures of polysaccharides. Due to the attention to the details of the structure, we can efficiently obtain low-energy configurations that may be missed in the full-space conformational search.

## Data Availability

Data are contained within the article and Supplementary Materials.

## Supplementary Materials

The following supporting information can be downloaded at: https://www.mdpi.com/article/10.3390/molecules28248093/s1, Figure S1: Structural formulas of Manα(1,3)Manα(1,6)Man, Figure S2: Building tree of Manβ(1,3) Manβ(1,6)Man, Figure S3: Representative configurations of *cis-cis* glycosidic linkage for Manα(1,3)Manα(1,6)Man, Figure S4: Representative configurations of *cis-trans* glycosidic linkage for Manα(1,3)Manα(1,6)Man, Figure S5: Representative configurations of *trans-cis* glycosidic linkage for Manα(1,3)Manα(1,6)Man, Figure S6: Representative configurations of *trans-trans* glycosidic linkage for Manα(1,3)Manα(1,6)Man, Figure S7: Computed vibrational spectra of the three lowest energy conformers. Figure S8: Representative structures of core pentasaccharide in the first construction way, Figure S9: Representative structures of core pentasaccharide in the second construction way, Figure S10: Representative structures of core pentasaccharide in the third construction way, Table S1: Relative energies of a trisaccharide and pentasaccharide configurations, Table S2: Cartesian coordinates of optimized configurations for Manβ(1,4)GlcNAcβ(1,4)GlcNAc, Table S3: Cartesian coordinates of optimized configurations for Manβ(1,3)Manβ(1,6)Man, Table S4. Cartesian coordinates of optimized configurations for core pentasaccharide.

## References

[1] Hsu H.C., Liew C.Y., Huang S.P., Tsai S.T., Ni C.K. (2018). Simple approach for de novo structural identification of mannose trisaccharides. J. Am. Soc. Mass. Spectr..

[2] Iramain M.A., Davies L., Brandán S.A. (2016). FTIR, HATR and FT-Raman studies on the anhydrous and monohydrate species of maltose in aqueous solution. Carbohyd. Res..

[3] Nunes S.C., Jesus A.L., Moreno M.J., Eusébio M.E.S. (2010). Conformational preferences of alpha, alpha-trehalose in gas phase and aqueous solution. Carbohyd. Res..

[4] Çarçabal P., Hünig I., Gamblin D.P., Liu B., Jockusch R.A., Kroemer R.T., Snoek L.C., Fairbanks A.J., Davis B.G., Simons J.P. (2006). Building up key segments of N-glycans in the gas phase: Intrinsic structural preferences of the alpha(1,3) and alpha(1,6) dimannosides. J. Am. Chem. Soc..

[5] Goel A., Kumar A., Hemberger Y., Raghuvanshi A., Jeet R., Tiwari G., Knauer M., Kureel J., Singh A.K., Gautam A. (2012). Synthesis, optical resolution, absolute configuration, and osteogenic activity of cis-pterocarpans. Org. Biomol. Chem..

[6] Zhang M., Geng Z., Yu Y. (2015). Density Functional Theory (DFT) study on the pyrolysis of cellulose: The pyran ring breaking mechanism. Comput. Theor. Chem..

[7] Schnupf U., Willett J.L., Bosma W.B., Momany F.A. (2008). DFT conformational studies of α-maltotriose. J. Comput. Chem..

[8] Bozell J.J., Petersen G.R. (2010). Technology development for the production of biobased products from biorefinery carbohydrates-the us department of energy′s ‘Top 10’ revisited. Green Chem..

[9] Zając A., Hanuza J., Wandas M., Dymińska L. (2015). Determination of N-acetylation degree in chitosan using Raman Spectroscopy. Mol. Biomol. Spectrosc..

[10] Gómez F.N., Combariza M.Y., Blanco-Tirado C. (2017). Facile cellulose nanofibrils amidation using a ‘one-pot’ approach. Cellulose.

[11] Bisinella R.Z.B., Ribeiro J.C.B., de Oliveira C.S., Colman T.A.D., Schnitzler E., Masson M.L. (2017). Some instrumental methods applied in food chemistry to characterize lactulose and lactobionic acid. Food Chem..

[12] Zheng T., Jiang H., Gros M., Soriano del Amo D., Sundaram S., Lauvau G., Marlow F., Liu L., Stanley P., Wu P. (2011). Tracking N-acetyllactosamine on cell-surface glycans in vivo. Angew. Chem. Int. Ed..

[13] Wang X., Zhang Z., Zhao M., Qi H. (2014). Effect of phthaloylation on radical-scavenging and moisture-preserving activities of polysaccharide from *Enteromorpha linza*. Carbohydr. Polym..

[14] Harvey D.J., Watanabe Y., Allen J.D., Rudd P., Pagel K., Crispin M., Struwe W.B. (2018). Collision Cross Sections and Ion Mobility Separation of Fragment Ions from Complex N-Glycans. J. Am. Soc. Mass Spectrom..

[15] Amarasekara A.S., Hasan M.A., Ha U. (2016). A two step method for the preparation of carbamate cross-linked cellulose films using an ionic liquid and their water retention properties. Carbohydr. Polym..

[16] Wang P., Hu Y., Zhan H., Chen J., Jin S., Song W., Li Y. (2017). Vibrational spectroscopy of the mass-selected tetrahydrofurfuryl alcohol monomers and its dimers in gas phase using IR depletion and VUV single photon ionization. Mol. Biomol. Spectrosc..

[17] Gray C.J., Schindler B., Migas L.G., Pičmanová M., Allouche A.R., Green A.P., Mandal S., Motawia M.S., Sánchez-Pérez R., Bjarnholt N. (2017). Bottom-up elucidation of glycosidic bond stereochemistry. Anal. Chem..

[18] Xia S., Gao B., Li A., Xiong J., Ao Z., Zhang C. (2014). Preliminary characterization, antioxidant properties and production of chrysolaminarin from marine diatom *Odontella aurita*. Mar. Drugs.

[19] Vinogradov E., MacLean L.L., Crump E.M., Perry M.B., Kay W.W. (2003). Structure of the polysaccharide chain of the lipopolysaccharide from *Flexibacter maritimus*. Eur. J. Biochem..

[20] Khan I., Ullah S., Oh D.H. (2016). Chitosan grafted monomethyl fumaric acid as a potential food preservative. Carbohyd. Polym..

[21] Buczek A., Makowski M., Jewgiński M., Latajka R., Kupka T., Broda M.A. (2014). Toward engineering efficient peptidomimetics. Screening conformational landscape of two modified dehydroaminoacids. Biopolymers.

[22] Schindler B., Laloy-Borgna G., Barnes L., Allouche A., Bouju E., Dugas V., Demesmay C., Compagnon I. (2018). Online Separation and Identification of Isomers Using Infrared Multiple Photon Dissociation Ion Spectroscopy Coupled to Liquid Chromatography: Application to the Analysis of Disaccharides Regio-Isomers and Monosaccharide Anomers. Anal. Chem..

[23] Carçabal P., Jockusch R.A., Hünig I., Snoek L.C., Kroemer R.T., Davis B.G., Gamblin D.P., Compagnon I., Oomens J., Simons J.P. (2005). Hydrogen bonding and cooperativity in isolated and hydrated sugars: Mannose, galactose, glucose, and gactose. J. Am. Chem. Soc..

[24] Hünig I., Painter A.J., Jockusch R.A., Çarçabal P., Marzluff E.M., Snoek L.C., Gamblin D.P., Davisc B.G., Simons J.P. (2005). Adding water to sugar: A spectroscopic and computational study of α- and β-phenylxyloside in the gas phase. Phys. Chem. Chem. Phys..

[25] Insausti A., Alonso E.R., Tercero B., Santos J.I., Calabrese C., Vogt N., Corzana F., Demaison J., Cernicharo J., Cocinero E.J. (2021). Laboratory Observation of, Astrochemical Search for, and Structure of Elusive Erythrulose in the Interstellar Medium. J. Phys. Chem. Lett..

[26] Barone V., Fuse M., Aguado R., Potenti S., León I., Alonso E.R., Mata S., Lazzari F., Mancini G., Spada L. (2023). Bringing Machine-Learning Enhanced Quantum Chemistry and Microwave Spectroscopy to Conformational Landscape Exploration: The Paradigmatic Case of 4-Fluoro-Threonine. Chem.–A Eur. J..

[27] Barnesa L., Schindlera B., Chambertb S., Allouchea A., Compagnon I. (2017). Conformational preferences of protonated N-acetylated hexosamines probed by InfraRed Multiple Photon Dissociation (IRMPD) spectroscopy and ab initio calculations. Int. J. Mass Spectrom..

[28] Perez S., Makshakova O. (2022). Multifaceted computational modeling in glycoscience. Chem. Rev..

[29] Wei Z., Chen D., Zhao H., Li Y., Zhu J., Liu B. (2014). Ab initio investigation of the first hydration shell of protonated glycine. J. Chem. Phys..

[30] Song R., Chen D., Suo C., Guo Z. (2020). Ab initio investigation of the first hydration shell of glucose. Carbohydr. Res..

[31] Carcabal P., Patsias T., Hünig I., Liu B., Kaposta C., Snoek L.C., Gamblin D.P., Davis B.G., Simons J.P. (2006). Spectral signatures and structural motifs in isolated and hydrated monosaccharides: Phenyl α- and β-fucopyranoside. Phys. Chem. Chem. Phys..

[32] Su Z., Cocinero E.J., Stanca-Kaposta E.C., Davis B.G., Simons J.P. (2009). Carbohydrate-aromatic interactions: A computational and IR spectroscopic investigation of the complex, methyl α-L-fucopyranoside toluene, isolated in the gas phase. Chem. Phys. Lett..

[33] Li Y., Liu X., Chen D., Wei Z., Liu B. (2013). Predicting the preferred conformations ofluteolin-4′-O-β-D-glucoside in gas phase: A comparison of two computational approaches. J. Mol. Model..

[34] Chen D., Yao Y., Wei Z., Zhang S., Tu P., Liu B., Dong M. (2013). Determining the structural preferences of dimannosides through the linkage constraint and hydrogen-bonded network. Comput. Theor. Chem..

[35] Chen D., Wei Z., Yao Y., Liu B. (2015). A tree-step computational approach to simplify conformational determination of cellobiose and lactose. Carbohyd. Res..

[36] Gao J., Chen D., Song R., Xue H., Wang T., Liu B. (2018). Preferred conformational structures of disaccharides with β-1,4-linked N-acetylglucosamine and D-mannose in the gas phase: A tree-step computational approach study. Comput. Theor. Chem..

[37] Stanca-Kaposta E.C., Gamblin D.P., Cocinero E.J., Frey J., Kroemer R.T., Fairbanks A.J., Davis B.G., Simons J.P. (2008). Solvent interactions and conformational choice in a core N-glycan segment: Gas phase conformation of the central, branching trimannose unit and its singly hydrated complex. J. Am. Chem. Soc..

[38] Jo S., Qi Y., Im W. (2016). Preferred conformations of N-glycan core pentasaccharide in solution and in glycoproteins. Glycobiology.

[39] Barry C.S., Cocinero E.J., Çarçabal P., Gamblin D.P., Stanca-Kaposta E.C., Remmert S.M., Fernández-Alonso M.C., Rudić S., Simons J.P., Davis B.G. (2013). ‘Naked’ and hydrated conformers of the conserved core pentasaccharide of N-linked glycoproteins and its building blocks. J. Am. Chem. Soc..

[40] Gloaguen E., Mons M., Schwing K., Gerhards M. (2020). Neutral Peptides in the Gas Phase: Conformation and Aggregation Issues. Chem. Rev..

[41] Johnson R.D. III.NIST Computational Chemistry Comparisonand Benchmark Database, Version 13; NIST Standard Reference Database Number 101, May 2022. https://cccbdb.nist.gov/vibscale.asp.

[42] Frisch M.J., Trucks G.W., Schlegel H.B., Scuseria G.E. (2009). Gaussian 09, Revision A.01.

