# Supplementary material for: Gas Phase Conformation of Trisaccharides and Core Pentasaccharide: A Three-Step Tree-Based Sampling and Quantum Mechanical Computational Approach
Source: Molecules. 2023 Dec 14;28(24):8093. doi: 10.3390/molecules28248093 (PMC10745714; doi:10.3390/molecules28248093)
Supplement: Supplementary file 1 [file molecules-28-08093-s001.zip › molecules-2726926-supplementary.pdf]

Supplementary Information

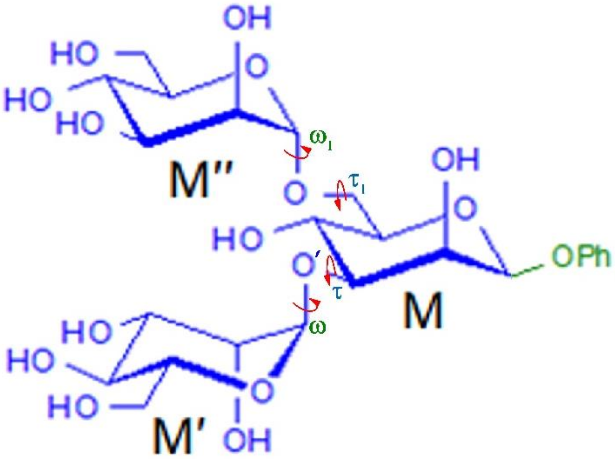

Figure S1. Structural formulas of Manα(1,3)Manα(1,6)Man. Here, glycosidic linkage torsion angles (ω, τ, ω_1 and τ_1) are used for distinguishing the structures of *cis*, *trans*, *cis* and *trans*, that is, ω=OM′-C1′-O′–C3，τ=C1′-O′-C3-C2，ω_1= OM′′-C1′′-O-C6，τ_1= C1′′-O-C6-C5.

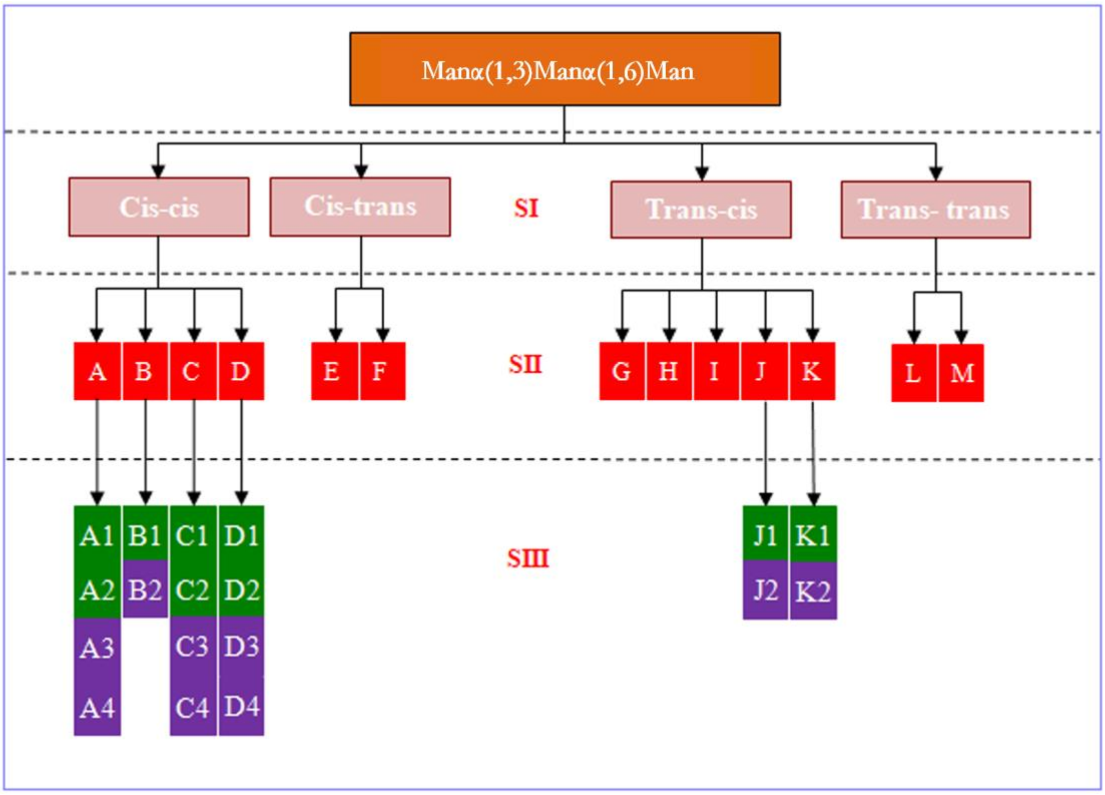

Figure S2. Building tree of Manα(1,3)Manα(1,6)Man. Regains SI, SII and SIII reflect tree-step construction. SI considers the constraint of glycosidic bond linkage. SII takes into account the inter-ring H-bonds. SIII refers to the cooperative hydrogen bonding orientation. The green and purple colors indicate the counterclockwise and clockwise orientations of the cooperative H-bonds, respectively.

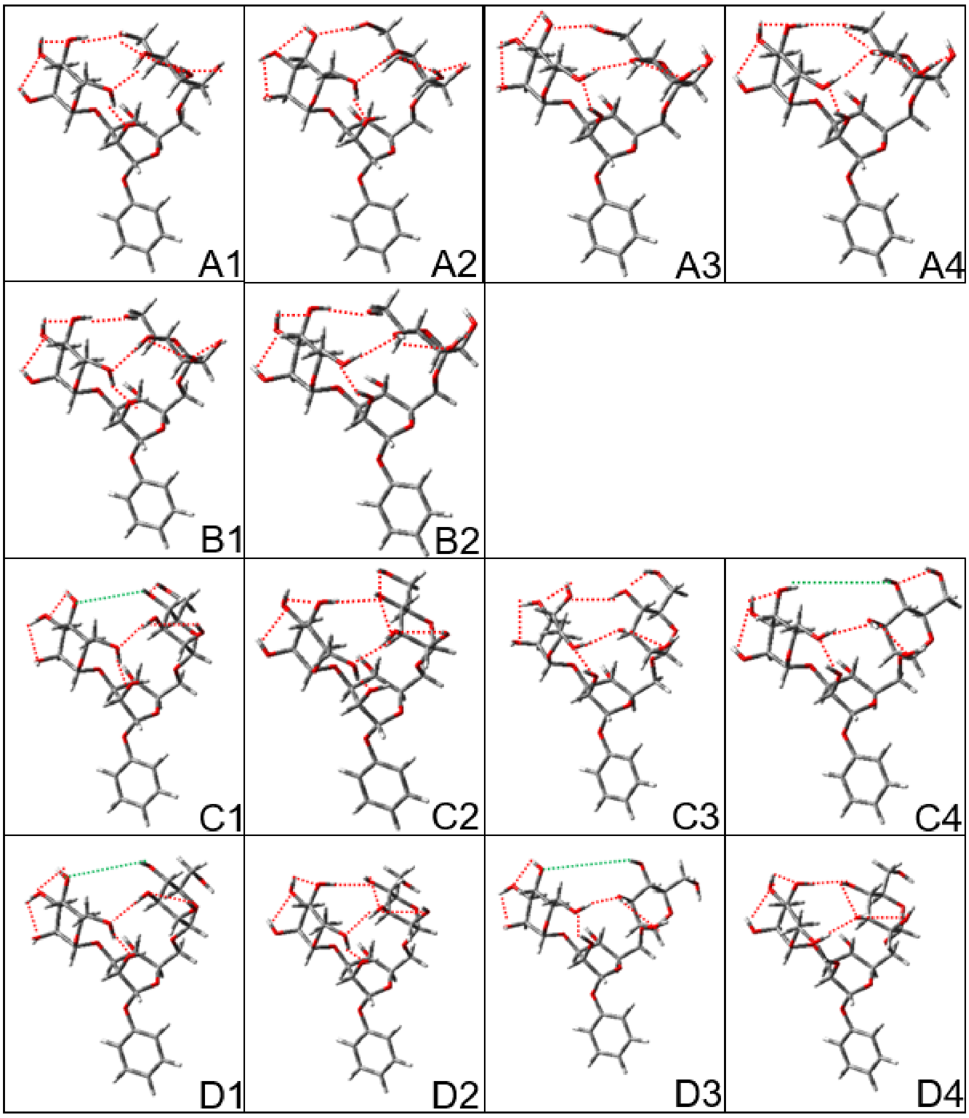

Figure S3. Representative configurations of *cis*-*cis* glycosidic linkage for Manα(1,3)Manα(1,6)Man.

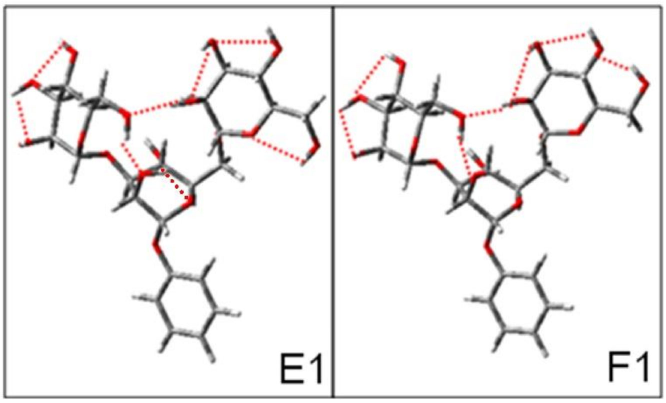

Figure S4. Representative configurations of *cis-trans* glycosidic linkage for Manα(1,3)Manα(1,6)Man.

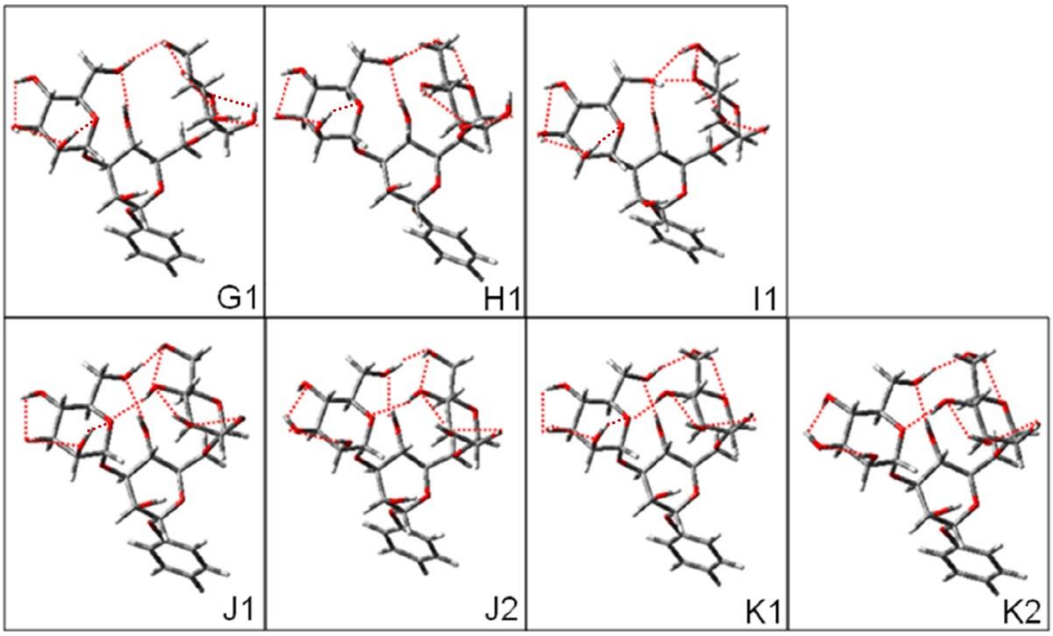

Figure S5. Representative configurations of *trans*-*cis* glycosidic linkage for Manα(1,3)Manα(1,6)Man.

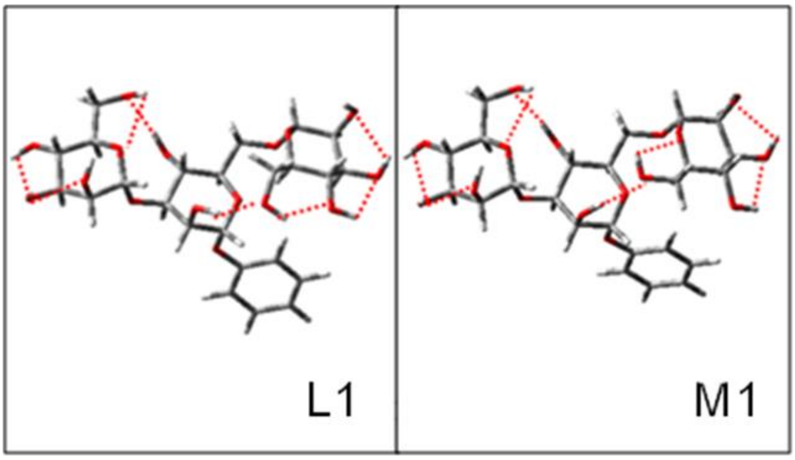

Figure S6. Representative configurations of *trans*-*trans* glycosidic linkage for Manα(1,3)Manα(1,6)Man.

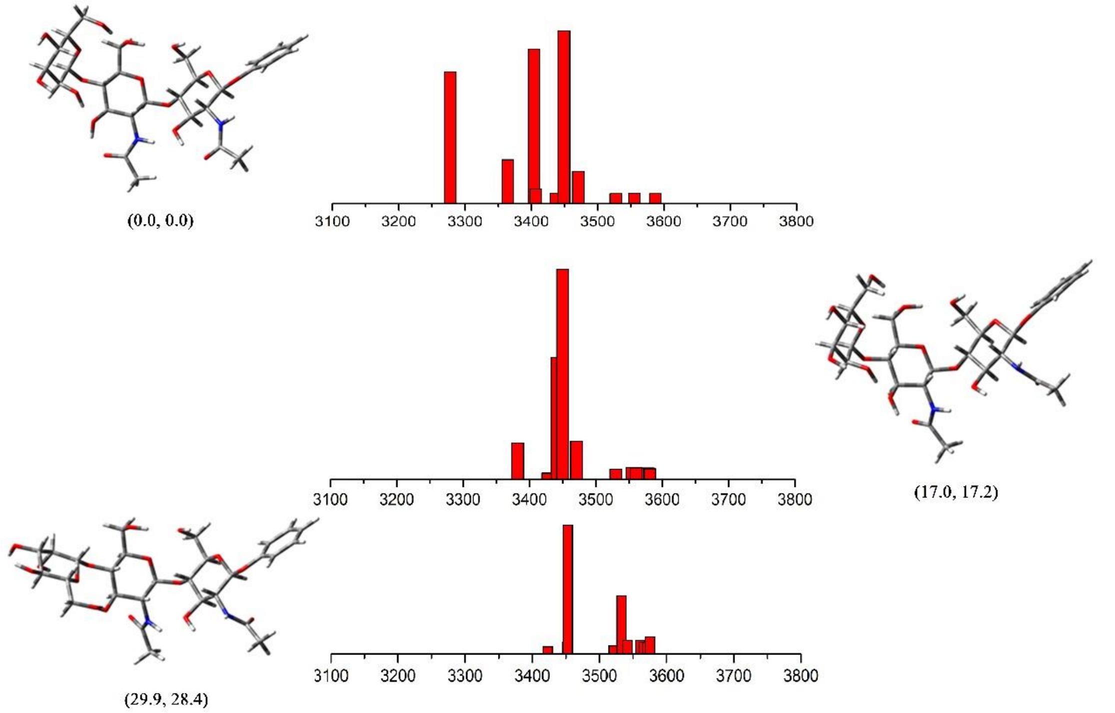

Figure S7. Computed vibrational spectra of the three lowest energy conformers. Calculated optimized structures and relative and free energies (kJ/mol^{-1} shown in brackets) are calculated using DFT (M06-2X/6-31+G*). Free energies are determined at 298.15 K.

Clearly, the lowest energy configuration obtained by B3LYP still processes the lowest relative energy at the M06-2X level. An overlay of the conformations for Manβ(1,4)GlcNAcβ(1,4)GlcNAc suggests that the second and third lowest energy configurations in Figure S7 are in good accord with the two lowest-energy conformers generated using the random search procedure in Simons' work except for the orientation of NHCO. The difference between the second and third lowest energy configurations is 12.9 kJ/mol in energy, which is 0.7 kJ/mol higher than that in Ref. [39]. The orders of relative energy are in agreement with that of Gibbs free energy. The results suggest that the lowest energy configuration we built is more favorable at 0 K and 298.15 K. For the lowest energy configuration, the agreement is quite good in terms of the correspondence between the strong band at 3450 cm^{-1} of the experimental and computed vibrational spectra. But there is a discrepancy of 70 cm^{-1} for the strong band at 3350 cm^{-1} in the experimental spectrum. The band located at a low wavenumber of 3278 cm^{-1} in the computed spectrum of the lowest energy configuration is assigned to the OH3 vibrations on G ring of the lowest energy conformation. In the comparison with conformational structures, we find that the difference between the lowest-energy configuration calculated by Ref. [39] and that in our calculations is the rotation of acetylamino groups on G and G' rings. The intra-ring H-bond related to the acetylamino group is not formed in Ref. [39]. However, the lowest-energy conformer in our work

will result to form the intra-ring H-bond of OH3→NHCO→O1, which further constructs a counterclockwise cooperative H-bond of $OH4_M$→$OH3_M$→$OH2_M$→OH3’→NHCO’→OH3→NHCO→O1.

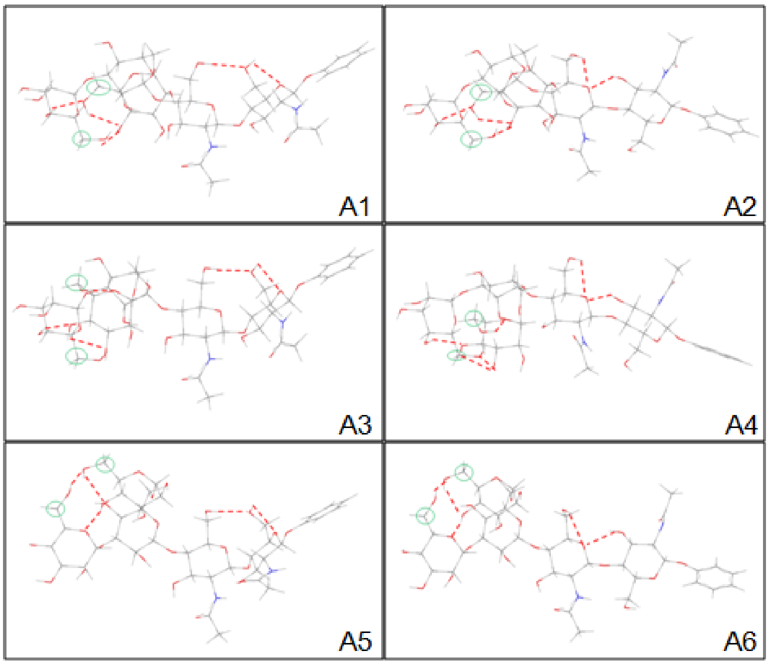

Figure S8. Representative structures of core pentasaccharide in the first construction way.

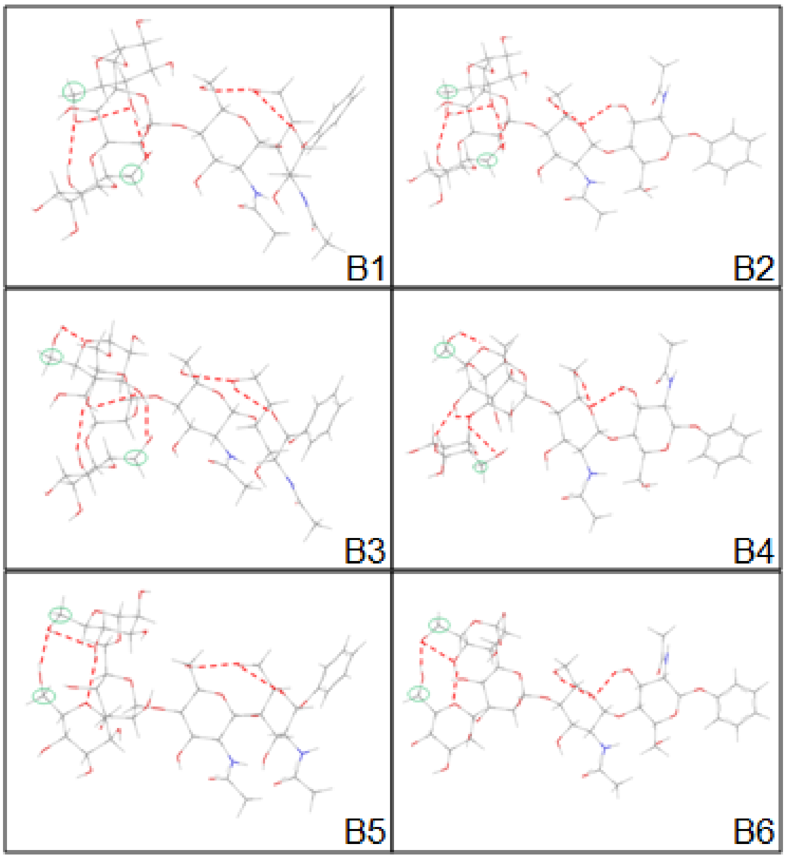

Figure S9. Representative structures of core pentasaccharide in the second construction way.

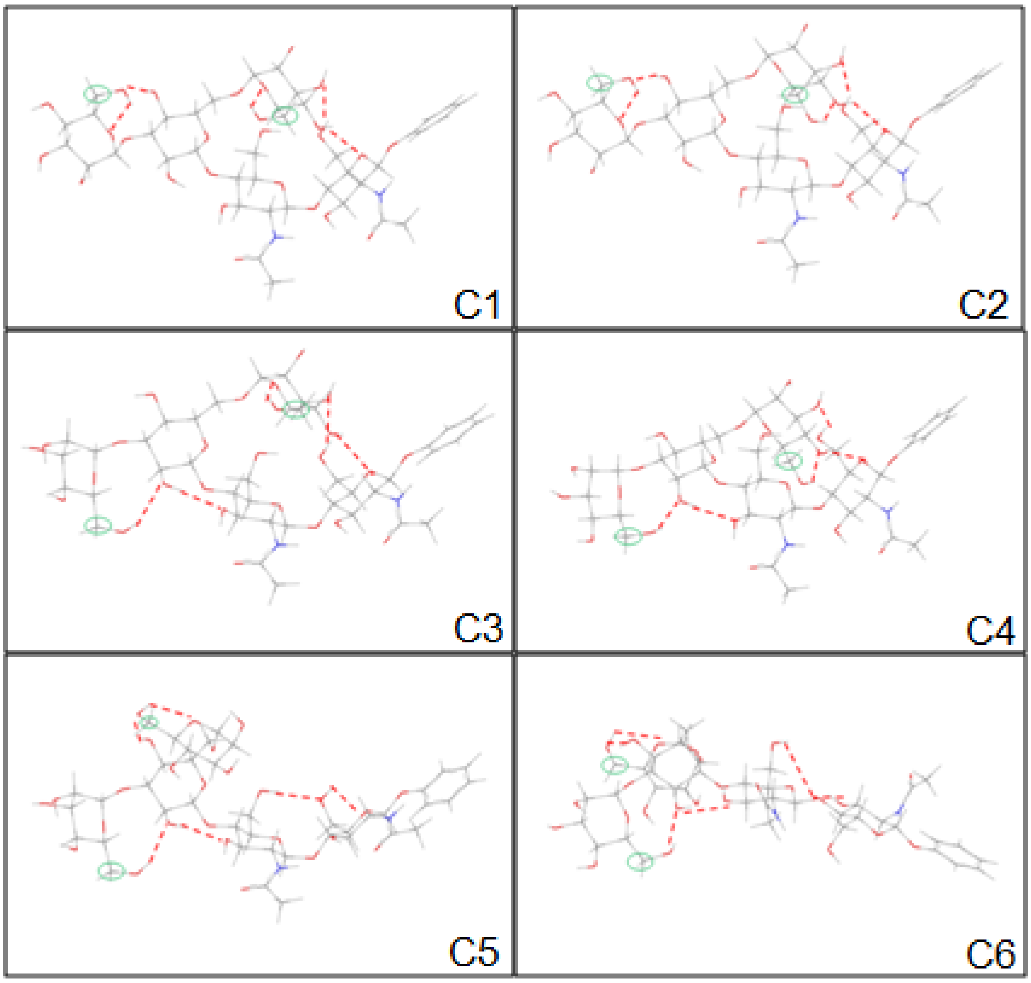

Figure S10. Representative structures of core pentasaccharide in the third construction way.

Table S1. Relative energies of trisaccharide and pentasaccharide conformers.

Manβ(1,4)GlcNAcβ(1,4) GlcNAc		Manα(1,3)Manα(1,6)Man		$Man_3GlcNAc_2$	
Conformer	Relative energy (kJ/mol)	Conformer	Relative energy (kJ/mol)	Conformer	Relative energy (kJ/mol)
A1	0.0	A1	0.0	A1	0.0
A2	20.7	A2	37.8	A2	8.6
A3	9.8	A3	18.8	A3	14.9
B1	36.5	A4	28.2	A4	17.6
C1	17.8	B1	7.6	A5	0.3
D1	19.9	B2	13.7	A6	6.3
E1	23.2	C1	11.2	B1	4.1
F1	13.1	C2	19.3	B2	13.2
G1	17.4	C3	38.3	B3	15.9
G2	50.6	C4	41.8	B4	31.4
H1	19.7	D1	8.4	B5	14.7
H2	48.7	D2	21.2	B6	19.2
I1	22.2	D3	32.5	B7	0.5
I2	51.9	D4	41.0	B8	5.9
J1	6.1	E1	44.1	B9	14.5
J2	26.6	F1	22.4	B10	18.5
K1	44.7	G1	20.1	B11	8.5
K2	37.3	H1	22.4	B12	14.0
K3	38.3	I1	17.9	C1	3.8
K4	40.5	J1	8.1	C2	6.4
K5	59.7	J2	5.1	C3	4.1
K6	40.5	K1	6.1	C4	6.6
K7	51.4	K2	5.5	C5	25.7
K8	55.0	L1	42.5	C6	20.1
L1	36.1	M1	46.2		
L2	30.5				
L3	31.7				
L4	32.2				
L5	60.1				
L6	50.1				
L7	53.4				
L8	53.0				
M1	31.1				
M2	30.5				

M3	29.9
M4	30.5
M5	48.9
M6	46.0
M7	45.2
M8	47.2
N1	34.0
N2	28.0
N3	26.7
N4	24.4
N5	54.8
N6	44.9
N7	42.6
N8	44.5
O1	37.1
O2	36.5
O3	36.4
O4	34.4
P1	24.8
P2	43.9
Q1	24.7
Q2	44.2
R1	13.2
R2	29.9
S1	40.1
S2	57.6
T1	24.5

Table S2. Cartesian coordinates of optimized configurations for Manβ(1,4)GlcNAcβ(1,4)GlcNAc

A1

C	2.52283000	1.87339800	-0.91076200
C	0.99962800	1.97083100	-0.71431100
C	0.29835200	1.21651700	-1.86591700
C	2.15355500	-0.31750700	-2.09235600
C	2.96412500	0.40568300	-0.99965900
H	0.75514500	1.47024000	0.22900800
H	2.80722900	2.36317700	-1.84872200
H	2.45565100	0.09328300	-3.06801800
H	0.51136000	1.73073600	-2.81270600
O	0.74316500	-0.11658100	-1.90948000
O	3.20886400	2.50522600	0.15947600
C	2.34013900	-1.83183900	-2.12635100
H	3.39590900	-2.06054400	-2.24856600
H	1.79424800	-2.22856900	-2.99191800
H	3.18213800	3.46282400	-0.03631600
O	1.90958500	-2.46834400	-0.93465400
H	0.93773600	-2.45974300	-0.88866700
C	-2.74076700	1.47953800	-0.07554500
C	-3.72414600	0.75037500	0.86842000
C	-1.78412700	0.48671300	-0.76632400
H	-3.31814700	1.98910600	-0.86151600
C	-4.37620700	-0.42084000	0.11528400
H	-3.15323800	0.33338800	1.70586100
C	-2.56284400	-0.68492900	-1.39344300
H	-1.07498600	0.09519600	-0.03392600
H	-5.00064400	-0.03704900	-0.70741500
H	-3.18685100	-0.29017000	-2.21018700
O	-3.40044400	-1.29113300	-0.39534000
O	-5.17221300	-1.11506200	1.03404600
O	-1.11165300	1.20668100	-1.80804200
C	-1.72212300	-1.82410400	-1.95214100
H	-2.39096600	-2.54315800	-2.43919100
H	-1.01836200	-1.44056200	-2.68505100
O	-0.94892700	-2.47689100	-0.95056200
H	-1.53744400	-2.87020600	-0.29439900
O	-1.93040000	2.40906900	0.61513500
H	-2.53513400	3.04814800	1.05717800
O	4.34429400	0.38925900	-1.39687900
C	-6.15922500	-1.97902100	0.58230300
C	-6.27758100	-2.41423300	-0.73635400
C	-7.06176800	-2.41050400	1.55524400
C	-7.32654900	-3.27107300	-1.07652300
H	-5.55425600	-2.12346300	-1.48643900
C	-8.09584600	-3.27064100	1.20303200
H	-6.93758600	-2.07046000	2.57763400
C	-8.23745100	-3.70154000	-0.11683500
H	-7.41736300	-3.61055800	-2.10315600
H	-8.79393500	-3.60417900	1.96371500
H	-9.04474200	-4.37188400	-0.39045400
H	2.82840500	-0.10296800	-0.04487500
N	-4.84190900	1.55816900	1.39030500
H	-5.61993700	0.98260300	1.68072400
C	-6.20370800	3.29440900	2.40986000
H	-6.00133800	3.59577600	3.44021400
H	-6.57731800	4.17408500	1.88346900
H	-6.97781200	2.52535600	2.41181600
N	0.45573800	3.32831400	-0.63553400
H	-0.51640200	3.31245700	-0.34104600
C	0.12206800	5.72794700	-0.62611100
H	0.47281400	6.36370900	0.18946700
H	0.18516200	6.31663700	-1.54388600
H	-0.92048000	5.46010400	-0.44797200
C	1.05395900	4.53690900	-0.75257100
O	2.25959400	4.72028500	-0.94894100
C	-4.90060600	2.86216100	1.76871600
O	-3.99212500	3.67914400	1.61567000
C	5.29066000	-0.29765500	-0.61139300
C	5.68388600	0.41034900	0.70434500
H	6.18898100	-0.31929300	-1.24760500
C	5.67297900	-2.49653700	0.32607100
C	6.63285800	-0.49375900	1.49559600
H	6.20315500	1.33873500	0.43125400
C	6.02936300	-1.88813100	1.68149800
H	6.60507600	-2.66169600	-0.23867300
H	7.58520700	-0.59672500	0.96495800
H	5.11745800	-1.81617500	2.28457900
O	4.81738800	-1.60943700	-0.40700100
O	6.97513400	-2.75043100	2.30722900
O	6.95144400	0.08385200	2.75807400
O	4.60319400	0.69716600	1.57569200
C	4.91923300	-3.82388500	0.42412100
H	4.83895600	-4.25365800	-0.58446500
H	5.50411700	-4.50934300	1.03951100
O	3.64770700	-3.69567200	1.02226400
H	3.05902500	-3.22298500	0.40732900
H	7.30361500	-2.28960700	3.09086300
H	6.13075300	0.48382700	3.08554800

H	4.11703200	1.46938100	1.22776900
A2			
C	2.53030200	2.11916700	-0.63591500
C	0.98881000	2.21458100	-0.57984900
C	0.32670300	1.55380200	-1.79944600
C	2.24714600	0.10917800	-2.12936600
C	2.95901700	0.66621400	-0.88222200
H	0.66203600	1.67969100	0.31376700
H	2.89380300	2.72831400	-1.46610200
H	2.58754700	0.67795500	-3.00804300
H	0.49543400	2.15914300	-2.69354100
O	0.82756100	0.24109900	-1.98539200
O	3.09039700	2.66877400	0.54431800
C	2.47414800	-1.37555300	-2.40304600
H	3.53908100	-1.56985300	-2.49953500
H	1.98551100	-1.62788300	-3.35234900
H	3.33308100	1.94903400	1.15023900
O	1.98735900	-2.21646500	-1.36412900
H	1.01359000	-2.20460600	-1.35845900
C	-2.52687300	1.44597800	0.23199700
C	-3.50407800	0.56794500	1.05375900
C	-1.66046700	0.59756100	-0.72216000
H	-3.11124800	2.14580400	-0.38357000
C	-4.21474900	-0.43973100	0.13670000
H	-2.91606200	-0.00445200	1.78043700
C	-2.49127000	-0.44937700	-1.48305700
H	-0.89141900	0.08366000	-0.14215800
H	-4.86680900	0.09114100	-0.57570200
H	-3.15351200	0.07667300	-2.18712900
O	-3.28283600	-1.22045000	-0.56376600
O	-4.98796300	-1.27475500	0.95547600
O	-1.08019100	1.46137000	-1.70374800
C	-1.67808000	-1.46887700	-2.26895200
H	-2.36443600	-2.09237800	-2.85355500
H	-0.99753000	-0.95655600	-2.94297500
O	-0.87291200	-2.29172500	-1.42738600
H	-1.44203700	-2.75410200	-0.79948200
O	-1.63906000	2.15263400	1.07590400
H	-2.19093200	2.70711200	1.66922000
O	4.38640200	0.66773100	-1.11706000
C	-5.99809800	-2.04567500	0.40215400
C	-6.18621400	-2.22985200	-0.96682400
C	-6.85236800	-2.65547200	1.32247100
C	-7.25380000	-3.01543500	-1.40616300
H	-5.50233400	-1.79972000	-1.68615100
C	-7.90586000	-3.44179400	0.86984400
H	-6.67560500	-2.50888200	2.38249700
C	-8.11601000	-3.62225800	-0.49809700
H	-7.39829700	-3.15849900	-2.47210700
H	-8.56564400	-3.91441600	1.59006300
H	-8.93867700	-4.23510800	-0.84981300
H	2.71826300	0.03245500	-0.02867400
N	-4.58221400	1.29248600	1.75296700
H	-5.38246900	0.70326600	1.93454000
C	-5.84227700	2.82687800	3.15617700
H	-5.61611900	2.89216900	4.22290500
H	-6.17714900	3.81477400	2.83580600
H	-6.65372700	2.11205900	3.00939700
N	0.51615900	3.58010200	-0.40940200
H	-0.16997600	3.69684900	0.31947200
C	0.13645900	5.93718900	-0.88298100
H	0.94332200	6.64166600	-0.66727000
H	-0.42279200	6.33181100	-1.73328000
H	-0.52086500	5.88494700	-0.01246900
C	0.75182800	4.60351600	-1.27215600
O	1.42573900	4.48657000	-2.29076600
C	-4.56728100	2.48153600	2.41204000
O	-3.61779200	3.26370400	2.42681600
C	5.22004900	-0.14431800	-0.35236900
C	5.40123700	0.26697500	1.12546000
H	6.20927000	-0.03399200	-0.82160700
C	5.56478900	-2.48971700	0.15232800
C	6.23961800	-0.78948400	1.84916900
H	5.92956500	1.22796200	1.13706900
C	5.64534100	-2.17887500	1.65110300
H	6.58384000	-2.50569300	-0.26859800
H	7.26419600	-0.78260100	1.45146900
H	4.63564500	-2.21262500	2.06858700
O	4.78213800	-1.48395500	-0.49004900
O	6.50407800	-3.08146700	2.35912400
O	6.25911100	-0.42270700	3.22676000
O	4.14994000	0.41109800	1.79957300
C	4.87658700	-3.81615000	-0.16984500
H	4.90075700	-3.96015000	-1.25808200
H	5.44249400	-4.63889200	0.27713200
O	3.56057000	-3.88378300	0.32982600
H	2.99260400	-3.29154200	-0.20209200
H	6.01485100	-3.88356200	2.57435600
H	6.60398200	-1.17740800	3.72317800
H	4.34318300	0.37922500	2.74914200

A3

C	2.42719900	1.93868700	-0.78382500
C	0.90001800	1.93704400	-0.58963500
C	0.23942100	1.16568600	-1.75912000
C	2.19280700	-0.20686500	-2.08435900
C	2.96342800	0.51335700	-0.96597800
H	0.69173300	1.40367500	0.34463500
H	2.68388700	2.50835600	-1.68473200
H	2.44035900	0.28046200	-3.04120100
H	0.41714500	1.72158400	-2.69028800
O	0.78643600	-0.12857300	-1.85361700
O	3.07958000	2.52800400	0.33222200
C	2.50090000	-1.69562100	-2.21654100
H	3.56430800	-1.82912400	-2.41240200
H	1.93693300	-2.07872300	-3.07309600
H	2.95025400	3.49696000	0.23220100
O	2.09524900	-2.42166000	-1.06347700
H	2.85451000	-2.78733600	-0.57661000
C	-2.94397900	1.43104800	-0.19463200
C	-3.99854900	0.79450700	0.73421200
C	-1.83568100	0.43564700	-0.59414700
H	-3.44324200	1.75703300	-1.11876300
C	-4.43045000	-0.55277400	0.13765200
H	-3.53534000	0.59469000	1.70660300
C	-2.40134500	-0.93595400	-1.01275800
H	-1.16289400	0.28171300	0.25059100
H	-4.92548600	-0.39263800	-0.83598300
H	-2.93599200	-0.82027900	-1.96927500
O	-3.32783800	-1.39260000	-0.01214900
O	-5.34375100	-1.13010800	1.03606800
O	-1.15802300	1.05666600	-1.70591000
C	-1.36777100	-2.04615400	-1.17325700
H	-1.92632000	-2.98034700	-1.31983500
H	-0.78647300	-1.84780000	-2.07438900
O	-0.53820600	-2.14802700	-0.03682900
H	0.38801500	-2.22052300	-0.32935000
O	-2.29462500	2.53142100	0.41999600
H	-2.99271900	3.16835600	0.69655500
O	4.34328000	0.61904600	-1.37062600
C	-6.16120800	-2.16454700	0.61494900
C	-5.96350500	-2.89083900	-0.55857300
C	-7.23359500	-2.46136700	1.45833800
C	-6.86594700	-3.90278400	-0.89117200
H	-5.10301400	-2.70392600	-1.18641400
C	-8.11844000	-3.47868800	1.11846100

H	-7.35507600	-1.89463300	2.37522500
C	-7.94336900	-4.20161600	-0.06249500
H	-6.70866600	-4.46911700	-1.80345400
H	-8.94852700	-3.70603800	1.77946100
H	-8.63445900	-4.99453200	-0.32705700
H	2.87223500	-0.05098000	-0.03785600
N	-5.23691600	1.57369800	0.92342800
H	-6.02527100	0.98158100	1.14470600
C	-6.83558000	3.32182700	1.46854200
H	-6.83605900	3.75762800	2.47030400
H	-7.14745100	4.10453800	0.77512800
H	-7.56208100	2.50819200	1.43663400
N	0.27713000	3.26211800	-0.50242400
H	-0.72328800	3.19085600	-0.31649100
C	-0.19395600	5.63276900	-0.37472400
H	0.01842700	6.22328100	0.51897500
H	-0.05418400	6.28975900	-1.23597500
H	-1.23206100	5.29989900	-0.34666400
C	0.81357400	4.50233800	-0.46321400
O	2.02224400	4.76686800	-0.49991000
C	-5.42026100	2.90267900	1.12478500
O	-4.53429300	3.75506800	1.03571900
C	5.34472800	-0.01811200	-0.63232500
C	5.65141700	0.60498000	0.75243000
H	6.24852100	0.10611400	-1.24745200
C	5.98051200	-2.22249700	0.10918500
C	6.66751700	-0.26974400	1.49603500
H	6.08571500	1.59576100	0.56592200
C	6.18061900	-1.72057500	1.53922600
H	6.95089300	-2.17138700	-0.41158700
H	7.63821200	-0.24488200	0.98955600
H	5.22060300	-1.74681700	2.07212100
O	5.02281000	-1.40091300	-0.55461700
O	7.12981400	-2.55953000	2.18282600
O	6.89762400	0.21346600	2.81232500
O	4.54089600	0.71717500	1.62353800
C	5.48310200	-3.66007000	-0.00019000
H	5.48818300	-3.96278900	-1.04850500
H	6.15574500	-4.31897600	0.55461000
O	4.13006200	-3.82279200	0.43070600
H	4.08080600	-3.81471100	1.39351700
H	7.43278300	-2.09355800	2.97495200
H	6.04483700	0.54868300	3.13235800
H	3.99327800	1.47703300	1.33496800

B1

C	2.29632700	1.79714600	-0.90328100
C	0.76910400	1.84087000	-0.72150600
C	0.11407000	0.95092700	-1.80060700
C	2.05182400	-0.49581700	-1.93915500
C	2.82261200	0.35378300	-0.90897100
H	0.53672000	1.42598400	0.26581800
H	2.56413700	2.24584100	-1.86619000
H	2.28771500	-0.12763900	-2.94647700
H	0.33938900	1.36732100	-2.79046500
O	0.63367800	-0.36820100	-1.70249900
O	2.94563200	2.51813100	0.13314900
C	2.32262800	-2.00912300	-1.88997200
H	1.55444800	-2.50282900	-2.48829900
H	2.21597800	-2.36143900	-0.85536600
H	2.85299700	3.46464100	-0.10872100
O	3.56408900	-2.38336000	-2.43970100
H	4.25231800	-2.15592700	-1.79604400
C	-2.99355000	1.33325100	-0.16972700
C	-4.02008400	0.73027200	0.81727300
C	-1.99639800	0.27535700	-0.68825400
H	-3.53744200	1.72858100	-1.04024700
C	-4.58955500	-0.56858400	0.22604300
H	-3.50258400	0.47524100	1.74901900
C	-2.73351400	-0.98895900	-1.17337600
H	-1.31208100	0.00222100	0.11957500
H	-5.18063500	-0.34464600	-0.67792800
H	-3.36329700	-0.72615000	-2.03544400
O	-3.55715200	-1.45832800	-0.09291300
O	-5.41714100	-1.13795900	1.20170400
O	-1.28562100	0.88406400	-1.78188200
C	-1.86859700	-2.19244400	-1.56962700
H	-1.10306900	-2.37056500	-0.80484200
H	-2.52564000	-3.06384900	-1.59055800
O	-1.30308700	-2.07266300	-2.85593600
H	-0.55110500	-1.46715900	-2.78211800
O	-2.21787700	2.35608600	0.42839200
H	-2.82048100	2.95519900	0.92749300
O	4.20100500	0.40012300	-1.31781900
C	-6.35088100	-2.09759100	0.83983000
C	-6.35557400	-2.76462200	-0.38397500
C	-7.32188000	-2.37500600	1.80310100
C	-7.36059800	-3.69887900	-0.64310200
H	-5.57672200	-2.59221700	-1.11466400
C	-8.31066100	-3.31486200	1.53398100
H	-7.28544000	-1.85378600	2.75359000
C	-8.33905200	-3.97796900	0.30620100
H	-7.36215300	-4.21920700	-1.59527000
H	-9.06229700	-3.52844400	2.28688400
H	-9.11126500	-4.71009900	0.09698800
H	2.72725100	-0.09302800	0.08329000
N	-5.18483100	1.58578200	1.11043800
H	-6.03169400	1.05549900	1.26090800
C	-6.54540100	3.37724300	2.04309400
H	-6.50049900	3.55133100	3.12095400
H	-6.73463900	4.34051000	1.56737500
H	-7.37690200	2.70377900	1.82925600
N	0.17084800	3.17652300	-0.79235900
H	-0.81634000	3.14438400	-0.54693800
C	-0.25535000	5.55346900	-0.97345100
H	0.02475600	6.27731100	-0.20574100
H	-0.16719200	6.05650600	-1.93909900
H	-1.29352400	5.25405600	-0.82412100
C	0.72956400	4.40072300	-0.94030900
O	1.93955200	4.62778000	-1.05365600
C	-5.19953900	2.86028700	1.58122900
O	-4.20545000	3.58573600	1.63578600
C	5.22885800	-0.06032900	-0.49134100
C	5.52420500	0.78518700	0.76500000
H	6.11906500	-0.01119400	-1.13424200
C	6.06277200	-2.08114600	0.51383200
C	6.65062900	0.12282200	1.56851100
H	5.85321100	1.77253100	0.41815800
C	6.31314600	-1.34440900	1.83117200
H	6.96545600	-2.03641000	-0.11248100
H	7.59307500	0.17256600	1.01386800
H	5.39739300	-1.39605400	2.43427900
O	4.97561100	-1.43140500	-0.16924000
O	7.38187400	-1.99466600	2.51725100
O	6.87747300	0.80339500	2.79529000
O	4.43360900	0.92207300	1.65952400
C	5.68480600	-3.55209500	0.70871500
H	4.87260300	-3.62699000	1.44591000
H	5.31834600	-3.95932600	-0.23550500
O	6.80047200	-4.33819100	1.08273100
H	7.22154400	-3.92326000	1.84992100
H	7.65225800	-1.42451400	3.25034000
H	6.00683400	1.08868900	3.11500300
H	3.84457600	1.61848100	1.30615500

C1

C	2.46539700	1.82820500	-0.59004800
C	1.08445600	2.02459500	0.09271000
C	0.13708500	0.88360400	-0.30827100
C	1.85969400	-0.60974300	-0.89279900
C	2.97728000	0.37137900	-0.51422700
H	1.22543400	1.97893000	1.17870200
H	2.36394200	2.08171900	-1.65489600
H	1.56957700	-0.44110200	-1.94060000
H	-0.09861600	0.95022300	-1.38234400
O	0.73909100	-0.35903100	-0.04357400
O	3.46499400	2.64473700	-0.00006400
C	2.18941800	-2.07865500	-0.72812900
H	2.39854000	-2.29823300	0.32419500
H	3.06616200	-2.33417200	-1.32388900
H	3.09741000	3.54819300	0.15054600
O	1.03125800	-2.79787000	-1.17733500
H	1.15942300	-3.74144900	-1.03299500
C	-2.43288800	-0.93668500	-0.32974900
C	-3.88465700	-1.25911600	-0.75747700
C	-2.25182700	0.58110900	-0.19127600
H	-2.21823400	-1.40796100	0.63310300
C	-4.95792700	-0.44295500	-0.01596500
H	-3.96856900	-0.97551500	-1.80964400
C	-3.37458900	1.19575800	0.64797000
H	-2.28631300	1.02365200	-1.19323700
H	-5.07854900	-0.78931900	1.01510300
H	-3.38582900	0.73652400	1.64816800
O	-4.61367000	0.94161300	-0.01515400
O	-6.14790500	-0.58984400	-0.73246000
O	-1.02926000	0.97617300	0.44404000
C	-3.23082100	2.70194700	0.82928900
H	-4.16101900	3.10047600	1.24741200
H	-2.42200400	2.90486500	1.52942400
O	-2.88450700	3.39231900	-0.37454800
H	-3.62032500	3.33220200	-0.99661100
O	-1.61346700	-1.47380600	-1.35753000
H	-0.76789100	-1.80440800	-1.01236100
O	4.04979600	0.23679300	-1.45998100
C	-7.35598600	-0.34714600	-0.11159700
C	-7.49934100	0.37878200	1.07094800
C	-8.47875400	-0.86915900	-0.75750900
C	-8.77412300	0.55891500	1.61061400
H	-6.63860700	0.81949300	1.55645000
C	-9.74327800	-0.67297600	-0.21405500
H	-8.34132200	-1.42656200	-1.67750200
C	-9.89869600	0.03853900	0.97675200
H	-8.88169600	1.12192300	2.53231400
H	-10.61086900	-1.08346000	-0.72074200
H	-10.88543500	0.18753600	1.40177300
H	3.32163900	0.15516800	0.49833100
N	-4.15774500	-2.69002700	-0.69626100
H	-4.22152700	-3.17847900	-1.57363000
C	-4.38994300	-4.91309800	0.28698900
H	-3.55496700	-5.45040400	0.74110400
H	-5.29237600	-5.19313000	0.83440700
H	-4.49499300	-5.23335400	-0.75164600
N	0.39568300	3.27863200	-0.26786300
H	-0.60233700	3.19072300	-0.42823300
C	-0.14857800	5.65305100	-0.31129300
H	-0.15017700	6.31350900	0.55801000
H	0.19284100	6.24616000	-1.16325300
H	-1.16192300	5.29896900	-0.49974100
C	0.84852200	4.54195800	-0.06503400
O	2.00427800	4.80732500	0.27798500
C	-4.16075900	-3.42251400	0.45457500
O	-4.00242500	-2.91676200	1.55891900
C	5.32125300	-0.18411300	-1.04450400
C	6.14599000	0.86831100	-0.27004700
H	5.84942000	-0.38139000	-1.98914200
C	6.45376500	-2.02240100	-0.03521000
C	7.50048700	0.25774600	0.10943900
H	6.30080200	1.72273000	-0.94165700
C	7.28664700	-1.07335100	0.82979100
H	6.98740000	-2.22303000	-0.97655300
H	8.10328800	0.08029000	-0.78688800
H	6.73886000	-0.88604400	1.76186900
O	5.19505200	-1.40033400	-0.32094900
O	8.53679200	-1.70004200	1.11989300
O	8.25730400	1.14261100	0.92557700
O	5.56330500	1.29804000	0.94651800
C	6.16666200	-3.36129300	0.64727200
H	5.75454500	-3.17993400	1.65039500
H	5.41433800	-3.90052500	0.06820500
O	7.31144700	-4.19267800	0.69883300
H	8.04153500	-3.67931200	1.07578900
H	9.11494500	-1.03361600	1.51613600
H	7.62117900	1.59105100	1.50510600
H	4.83312200	1.91871000	0.74158500

D1

C	2.48377100	1.91207000	-0.41856900
C	1.08038400	2.01332600	0.24209200
C	0.13362500	0.91184800	-0.25042000
C	1.88818300	-0.40933600	-1.16346600
C	3.00129400	0.46825600	-0.57914800
H	1.20239300	1.89020500	1.32337700
H	2.41641200	2.33706800	-1.42972200
H	1.57214700	0.00176900	-2.13286000
H	-0.19433700	1.12865800	-1.27970500
O	0.77850900	-0.34555600	-0.24370000
O	3.44840200	2.63681800	0.33384100
C	2.19939700	-1.88334900	-1.37238500
H	2.35545000	-2.37117700	-0.40227800
H	3.11508300	-1.98576000	-1.94865100
H	3.03917700	3.45451500	0.69681100
O	1.17701500	-2.52731600	-2.12237200
H	0.34714000	-2.52515500	-1.62720200
C	-2.46248400	-1.09651900	0.41046500
C	-3.81256000	-1.54061000	-0.18256600
C	-2.18898000	0.35791000	0.02242700
H	-2.50142900	-1.16211600	1.50293300
C	-4.94111400	-0.51155200	0.04813400
H	-3.68144100	-1.61116400	-1.26542700
C	-3.36410600	1.21950800	0.49582600
H	-2.12284600	0.43991700	-1.06708800
H	-5.28798800	-0.54102600	1.08530100
H	-3.55169800	1.04321700	1.56592200
O	-4.50395100	0.81406200	-0.26571100
O	-5.96950700	-0.84220400	-0.83365400
O	-0.97022800	0.84915500	0.59927800
C	-3.15302800	2.71512100	0.31464900
H	-4.10990100	3.23019500	0.44603300
H	-2.46432400	3.06973000	1.08067800
O	-2.55484800	3.07404800	-0.93448600
H	-3.18129800	2.91893400	-1.65190000
O	-1.47605500	-1.98898700	-0.09088000
H	-0.60920000	-1.65031200	0.18604400
O	4.09714900	0.50640500	-1.50370100
C	-7.25901300	-0.42050300	-0.57505600
C	-7.58522200	0.61013900	0.30610400
C	-8.26136400	-1.08978000	-1.27972700
C	-8.92669700	0.94959000	0.49098900
H	-6.81095200	1.15966000	0.82524700
C	-9.59230700	-0.73358700	-1.09375500
H	-7.98085200	-1.88479100	-1.96173100
C	-9.93381600	0.28550600	-0.20314800
H	-9.17817700	1.74981800	1.17973400
H	-10.36660500	-1.25938200	-1.64310600
H	-10.97284800	0.55973500	-0.05620600
H	3.31637200	0.06568900	0.38464200
N	-4.20340600	-2.86921000	0.27195600
H	-4.14335700	-3.61576000	-0.40039600
C	-4.71408300	-4.65927100	1.85287300
H	-3.90093900	-5.05327900	2.46716100
H	-5.63369500	-4.74697500	2.43345000
H	-4.80113400	-5.27161100	0.95309400
N	0.40853300	3.29388600	-0.03026100
H	-0.49462900	3.25105900	-0.49151400
C	-0.14002600	5.65487200	0.23774700
H	-0.43275400	6.06772100	1.20585300
H	0.40191300	6.43940100	-0.29418700
H	-1.03544000	5.38886700	-0.32512600
C	0.79596500	4.49053500	0.47521400
O	1.85384100	4.64581700	1.09110700
C	-4.44784400	-3.19232600	1.57684200
O	-4.45473100	-2.35530300	2.47047800
C	5.35608800	0.00466100	-1.13601400
C	6.16696100	0.91203900	-0.18474000
H	5.90302300	-0.04305900	-2.08939500
C	6.44545200	-1.98387900	-0.40695500
C	7.50653900	0.23397200	0.12626500
H	6.34571300	1.86142900	-0.70621900
C	7.26663200	-1.19433200	0.61635400
H	6.99946400	-2.04125900	-1.35602100
H	8.13085800	0.19638900	-0.77212500
H	6.70064400	-1.15608100	1.55584900
O	5.20065700	-1.30611700	-0.61863900
O	8.50603000	-1.87218200	0.82616600
O	8.24747000	0.96925700	1.09189500
O	5.55408400	1.14729100	1.07017900
C	6.12775700	-3.40973500	0.04831900
H	5.69544900	-3.38356600	1.05896500
H	5.38323600	-3.83999300	-0.62374700
O	7.26399000	-4.25314900	-0.00843000
H	7.98893500	-3.81745100	0.46362600
H	9.07830100	-1.28729600	1.34161400
H	7.59987100	1.32198100	1.72244700
H	4.82933600	1.79477900	0.95288800

E1

C	2.53346000	2.02867400	-0.50241400
C	1.14439600	2.43273200	0.06166400
C	0.06993000	1.47746600	-0.47786600
C	1.56251600	-0.26150800	-0.95899500
C	2.79234300	0.50258800	-0.45268300
H	1.17301500	2.32367300	1.15164900
H	2.58363400	2.32929300	-1.55807100
H	1.39103300	0.00914500	-2.01189100
H	-0.03561000	1.58771500	-1.56603500
O	0.44484200	0.15750300	-0.17588200
O	3.58476300	2.64850500	0.22091500
C	1.59432700	-1.78039200	-0.87687000
H	1.68569500	-2.10320100	0.16342800
H	2.44657200	-2.16819500	-1.43193000
H	3.44756700	3.62290000	0.18605500
O	0.39126700	-2.29364500	-1.46711000
H	-0.19201300	-2.68476000	-0.79867600
C	-2.32585800	-0.17354000	-0.89532700
C	-3.75219800	-0.69745000	-1.16068300
C	-2.36677600	1.29644100	-0.44695100
H	-1.86541100	-0.75898700	-0.10307900
C	-4.71068900	-0.36702700	-0.01086800
H	-4.13326900	-0.16479700	-2.03745400
C	-3.41629600	1.51969000	0.65322200
H	-2.59537000	1.92909500	-1.31165800
H	-4.43804100	-0.90981900	0.90601800
H	-3.09083000	0.98191800	1.55642300
O	-4.69261600	1.03007000	0.23204000
O	-5.99780900	-0.72323900	-0.42347600
O	-1.14014100	1.76107300	0.15769000
C	-3.61954600	2.98681000	1.01455700
H	-4.37992500	3.04978800	1.80255500
H	-2.68906900	3.39859900	1.40370700
O	-3.98040100	3.78657500	-0.10259300
H	-4.83195700	3.47710300	-0.43685500
O	-1.58478000	-0.29683300	-2.09839300
H	-0.89828700	-0.98186800	-1.98399100
O	3.90221700	0.22815100	-1.32057400
C	-6.99610300	-0.90442400	0.51468400
C	-6.92602000	-0.45132100	1.83182100
C	-8.13068500	-1.57993700	0.06094500
C	-7.99426900	-0.70032600	2.69565400
H	-6.07019200	0.11241300	2.17883200
C	-9.19116300	-1.81161800	0.92963100
H	-8.16516100	-1.91529500	-0.96983700
C	-9.12715000	-1.37770000	2.25470000
H	-7.93662000	-0.34733800	3.72031700
H	-10.06945300	-2.33796700	0.57016200
H	-9.95376900	-1.56166900	2.93227200
H	3.01568500	0.19942800	0.57150100
N	-3.79739500	-2.11977100	-1.50150900
H	-4.53527900	-2.37380300	-2.13817200
C	-3.32998200	-4.50342700	-1.62040000
H	-2.46275200	-4.84332300	-2.19128800
H	-3.47568600	-5.20722000	-0.79919300
H	-4.20710600	-4.52682900	-2.26988400
N	0.69573900	3.79145500	-0.29222700
H	-0.31030200	3.86397100	-0.35880000
C	0.55014300	6.20853900	-0.51847300
H	0.60973800	6.85511200	0.35986700
H	0.99329500	6.75573700	-1.35243800
H	-0.49922400	6.00891300	-0.74112900
C	1.37611700	4.96500800	-0.26016200
O	2.58750500	5.06201300	-0.05292600
C	-3.01982900	-3.13594600	-1.04076000
O	-2.10938900	-2.99333800	-0.22510700
C	5.04400600	-0.42260000	-0.83116800
C	5.97549900	0.45059100	0.03901600
H	5.60200500	-0.68624900	-1.74201100
C	5.75766100	-2.45513600	0.18375500
C	7.17059300	-0.39665100	0.49190100
H	6.32802400	1.28058600	-0.58714600
C	6.67641800	-1.68972200	1.14055700
H	6.31702400	-2.72303900	-0.72538800
H	7.80234000	-0.65176600	-0.36485500
H	6.10100700	-1.43662400	2.04016600
O	4.65234200	-1.61278500	-0.16392800
O	7.77523900	-2.53253800	1.49076200
O	8.00127000	0.32178700	1.39560500
O	5.38415400	0.94731700	1.22626000
C	5.19331300	-3.74027100	0.79305100
H	4.74639200	-3.51614700	1.77237200
H	4.40428500	-4.12461700	0.14421400
O	6.17243000	-4.75894300	0.89091600
H	6.94895300	-4.39007500	1.33740800
H	8.42541700	-1.98928900	1.95707200
H	7.40635700	0.85744100	1.94395900
H	4.78679200	1.68761900	0.99512700

F1

C	2.40327500	1.84784600	-0.52938600
C	0.99546500	1.93270400	0.11764300
C	0.07505500	0.80526500	-0.37695500
C	1.87005700	-0.53589400	-1.16732800
C	2.94067300	0.40899300	-0.60732000
H	1.10910000	1.81617200	1.20089900
H	2.33535100	2.22717300	-1.55868000
H	1.58858300	-0.20456800	-2.17956500
H	-0.21955500	0.99418400	-1.42322400
O	0.72060200	-0.45114000	-0.30933100
O	3.35047100	2.61986100	0.19718500
C	2.29428900	-2.00008400	-1.26432000
H	3.03510300	-2.08669000	-2.06633300
H	1.42128600	-2.59116200	-1.55268100
H	2.92722000	3.45716700	0.50041000
O	2.78049500	-2.53792100	-0.05404100
H	3.70356600	-2.25728200	0.03667600
C	-2.53825300	-1.14799600	-0.00412700
C	-3.95206500	-1.50712900	-0.50393900
C	-2.29075700	0.35436600	-0.15416100
H	-2.44465400	-1.40873900	1.05564000
C	-5.04299800	-0.53402900	-0.01542900
H	-3.93562500	-1.42061700	-1.59344900
C	-3.42533700	1.13056400	0.52051200
H	-2.27965800	0.61296800	-1.21838600
H	-5.28025300	-0.70882400	1.03771600
H	-3.52141500	0.81681800	1.57086500
O	-4.62664000	0.82262100	-0.18931900
O	-6.16152200	-0.74180100	-0.82320600
O	-1.05267300	0.77281900	0.43912100
C	-3.23185500	2.63953900	0.50615400
H	-4.17052100	3.12312100	0.79454900
H	-2.46709200	2.91056400	1.23270700
O	-2.77076200	3.14744400	-0.74998200
H	-3.46069000	3.03017000	-1.41481000
O	-1.64281000	-1.92199000	-0.78674600
H	-0.73659200	-1.74509100	-0.48422500
O	4.05999900	0.39501800	-1.51221500
C	-7.41115600	-0.35893800	-0.37952700
C	-7.63497800	0.50660300	0.69107300
C	-8.48797700	-0.89008000	-1.09250800
C	-8.94662600	0.81861400	1.05329200
H	-6.80658100	0.95191800	1.22624900
C	-9.78865300	-0.56227200	-0.72714000
H	-8.28729100	-1.55782600	-1.92301200
C	-10.02657700	0.29082800	0.35184400
H	-9.11718700	1.48939200	1.88935500
H	-10.62042200	-0.98069100	-1.28477000
H	-11.04191400	0.54244600	0.63857800
H	3.23310000	0.06526900	0.38536600
N	-4.29607900	-2.89275000	-0.20676400
H	-4.24497200	-3.54367600	-0.97281900
C	-4.61353200	-4.89671100	1.15296000
H	-3.72614000	-5.33893400	1.61248500
H	-5.45842300	-5.09876300	1.81285700
H	-4.78796300	-5.38261500	0.19081100
N	0.29463900	3.19697600	-0.17120300
H	-0.64911200	3.12077400	-0.53741400
C	-0.30048500	5.55388700	0.02540700
H	-0.49435100	6.05067800	0.97840400
H	0.17151200	6.29068200	-0.62859900
H	-1.24431000	5.23157400	-0.41409200
C	0.67572700	4.42354000	0.26481200
O	1.76007700	4.63804500	0.81461900
C	-4.41438600	-3.39646300	1.05796700
O	-4.36623800	-2.68761500	2.05517200
C	5.34087700	0.02271500	-1.09276900
C	6.04978800	0.99971100	-0.13055800
H	5.91978900	0.00398400	-2.02664700
C	6.62122400	-1.85673900	-0.35187700
C	7.44553000	0.45385400	0.19698700
H	6.14367200	1.96078900	-0.65152000
C	7.34471600	-0.99209600	0.68287400
H	7.17472800	-1.83916800	-1.30174600
H	8.08238300	0.48078200	-0.69297800
H	6.76520900	-1.01367100	1.61467900
O	5.30842600	-1.30564300	-0.56100800
O	8.64283200	-1.54146700	0.90728800
O	8.09703300	1.25585700	1.17270000
O	5.39860000	1.17785200	1.11385100
C	6.46560600	-3.31661000	0.08146100
H	6.03973300	-3.35867500	1.09440300
H	5.77232500	-3.82032200	-0.59476800
O	7.69070300	-4.02099100	0.00494900
H	8.35799400	-3.52240700	0.49943100
H	9.14694500	-0.90273600	1.43010500
H	7.41026600	1.53972900	1.79675200
H	4.65831200	1.80499000	0.98038800

G1

C	2.52795400	1.74339800	-0.55460100
C	1.14783500	1.95361800	0.12705500
C	0.18343700	0.83153800	-0.28516900
C	1.88188900	-0.68434100	-0.88208700
C	3.01733000	0.27730800	-0.50729100
H	1.28572900	1.89666100	1.21287700
H	2.43396400	2.01838400	-1.61477900
H	1.59250900	-0.50932700	-1.92890500
H	-0.04858600	0.91008000	-1.35920200
O	0.76706100	-0.42289800	-0.02977400
O	3.53927200	2.52991600	0.05495200
C	2.19385600	-2.15773100	-0.72252800
H	2.38532800	-2.38284000	0.33310900
H	3.07569600	-2.41962000	-1.30841100
H	3.19021800	3.43990600	0.20895900
O	1.03439100	-2.86381400	-1.18391700
H	1.14971400	-3.80893100	-1.03812400
C	-2.42265500	-0.94540200	-0.31920000
C	-3.87758200	-1.23758500	-0.75739300
C	-2.21049200	0.56768400	-0.17759500
H	-2.22418400	-1.42173900	0.64462900
C	-4.94040800	-0.39547500	-0.02922600
H	-3.94591400	-0.95669000	-1.81139600
C	-3.32597400	1.20372200	0.65550000
H	-2.22975000	1.01301800	-1.17865800
H	-5.08350900	-0.74015200	0.99951400
H	-3.35700500	0.74012300	1.65322200
O	-4.56378500	0.98047400	-0.02099600
O	-6.12279500	-0.51310500	-0.76336300
O	-0.98286700	0.93456100	0.46525500
C	-3.15055100	2.70532400	0.84511700
H	-4.07462100	3.12238700	1.25863200
H	-2.34231300	2.88741400	1.55162900
O	-2.78105000	3.39296500	-0.35342700
H	-3.51358900	3.35155900	-0.98086900
O	-1.60782700	-1.49946000	-1.34212600
H	-0.76643300	-1.83541700	-0.99299200
O	4.06851300	0.13163100	-1.47420100
C	-7.33409400	-0.23540800	-0.16371200
C	-7.47752100	0.48649900	1.02124300
C	-8.45999900	-0.71615900	-0.83572200
C	-8.75652400	0.70481300	1.53638500
H	-6.61318500	0.89557800	1.52751900
C	-9.72820500	-0.48228400	-0.31640300

H	-8.32212000	-1.27120600	-1.75707200
C	-9.88441400	0.22610300	0.87618000
H	-8.86434800	1.26485000	2.45985800
H	-10.59825900	-0.86055600	-0.84350700
H	-10.87416900	0.40487900	1.28226600
H	3.37574200	0.04592300	0.49718900
N	-4.18290100	-2.66174800	-0.69235300
H	-4.25652400	-3.15190800	-1.56799000
C	-4.47417000	-4.87397600	0.29998000
H	-3.65316600	-5.42889300	0.75839900
H	-5.38416200	-5.13034300	0.84644300
H	-4.58437200	-5.19597200	-0.73758000
N	0.48113100	3.22175100	-0.22523600
H	-0.51743500	3.15320700	-0.39192000
C	-0.01809100	5.60648200	-0.25859700
H	-0.01218000	6.26152800	0.61477900
H	0.33934100	6.19828800	-1.10487000
H	-1.03675400	5.27268400	-0.45491400
C	0.95679400	4.47551300	-0.01395800
O	2.11575400	4.71744600	0.33494700
C	-4.21034700	-3.38857200	0.46185200
O	-4.04571500	-2.88135000	1.56456600
C	5.35546900	-0.27273200	-1.08947200
C	6.18504700	0.77081900	-0.31635000
H	5.86224700	-0.45175000	-2.04895400
C	6.53962000	-2.12342000	-0.12795800
C	7.56806500	0.17719100	-0.03296100
H	6.28839700	1.65492400	-0.95835200
C	7.44852900	-1.18109000	0.66441000
H	7.02362100	-2.35903600	-1.08825200
H	8.11437900	0.03718400	-0.97152600
H	7.02106100	-1.03603100	1.66284700
O	5.26703700	-1.49893300	-0.37692700
O	8.72391200	-1.80564500	0.75805100
O	8.35788200	1.06479800	0.74860400
O	5.64397300	1.13545000	0.94066700
C	6.23585300	-3.42847300	0.59694900
H	5.66144800	-4.08088300	-0.07501800
H	7.17149300	-3.92746700	0.84840400
O	5.53895400	-3.22551700	1.81731900
H	4.79386300	-2.63664400	1.64203900
H	9.32862900	-1.16722800	1.15931400
H	7.76101100	1.45776100	1.40430700
H	4.91500200	1.76991200	0.78675000

G2

C	2.61557300	1.96354300	-0.41396100
C	1.21279700	2.05428500	0.23754900
C	0.21706200	1.05932000	-0.37893500
C	1.97990200	-0.33336000	-1.17656400
C	3.08822100	0.50371300	-0.51559000
H	1.31056000	1.77233400	1.29012700
H	2.55053000	2.36336000	-1.42887500
H	1.77364000	0.07079800	-2.17798500
H	-0.03458800	1.33219600	-1.40727900
O	0.80984500	-0.24159700	-0.36700500
O	3.52057100	2.79661800	0.29420100
C	2.28000900	-1.81208600	-1.31809900
H	2.39458000	-2.26087600	-0.32358500
H	3.20439600	-1.95375500	-1.88178800
H	4.07312600	2.25407700	0.88135000
O	1.16249500	-2.38469300	-2.00370600
H	1.26118900	-3.34161200	-2.05012800
C	-2.35763600	-0.74187900	-0.57313800
C	-3.82779100	-1.04441200	-0.93289200
C	-2.17265000	0.74477700	-0.22247800
H	-2.06760700	-1.33670400	0.29857300
C	-4.82730900	-0.42647100	0.05513400
H	-4.02663600	-0.57223200	-1.89855600
C	-3.24302900	1.20955900	0.77333400
H	-2.24552900	1.34473400	-1.13574100
H	-4.79653700	-0.93552000	1.02349500
H	-3.11854300	0.65683300	1.71714200
O	-4.54031400	0.95547900	0.22623000
O	-6.09813100	-0.53272800	-0.52009400
O	-0.91864500	1.01048100	0.42010700
C	-3.19730400	2.70109200	1.08336400
H	-3.96123200	2.92251200	1.83998500
H	-2.21981100	2.95616600	1.49009500
O	-3.38090700	3.51252000	-0.06773900
H	-4.22689700	3.27681800	-0.47037400
O	-1.60973900	-1.12729200	-1.71710500
H	-0.70385500	-1.37660100	-1.47511100
O	4.27219200	0.45444800	-1.34094100
C	-7.21985100	-0.42848700	0.27246800
C	-7.22292400	0.08063500	1.57170200
C	-8.41155200	-0.86123400	-0.31457900
C	-8.42364000	0.13346600	2.28171200
H	-6.31249600	0.45481400	2.02072000
C	-9.60144000	-0.79336300	0.40091500
H	-8.38427200	-1.24819200	-1.32727700
C	-9.61484800	-0.29958200	1.70659500
H	-8.42062000	0.52843700	3.29279200
H	-10.52227600	-1.13278300	-0.06284100
H	-10.54345000	-0.24996100	2.26508100
H	3.30052000	0.09631400	0.47518900
N	-4.05535300	-2.47327800	-1.12341100
H	-4.12476300	-2.79123900	-2.07554700
C	-4.11621800	-4.85303900	-0.58798000
H	-3.23240300	-5.42177100	-0.29206500
H	-4.97059900	-5.28230000	-0.06035100
H	-4.27153600	-4.97167900	-1.66242700
N	0.69556900	3.41760900	0.22177200
H	0.70273600	3.91937900	1.09374100
C	-0.22594200	5.48771500	-0.67338600
H	0.03984100	6.12539300	-1.51594100
H	-1.31645400	5.41117200	-0.63115700
H	0.12452600	5.94883200	0.25350800
C	0.33621500	4.09918500	-0.90729400
O	0.41177700	3.60731800	-2.02574800
C	-3.94194200	-3.41178400	-0.14209100
O	-3.73229700	-3.12686800	1.03089800
C	5.47571100	-0.01775900	-0.83879200
C	6.17680200	0.85828500	0.21567300
H	6.13972500	-0.03318500	-1.71501100
C	6.55497200	-2.01443600	-0.08534200
C	7.50179900	0.19995100	0.60919800
H	6.37101000	1.83749200	-0.23719000
C	7.28460300	-1.24955700	1.02946200
H	7.18329100	-2.02514400	-0.99014800
H	8.18931900	0.22164800	-0.24775400
H	6.66790500	-1.27476500	1.93145900
O	5.31345500	-1.35741500	-0.36875400
O	8.58049800	-1.78814900	1.30526400
O	8.03798300	0.97448400	1.67681900
O	5.36715500	1.02495600	1.38166200
C	6.21954800	-3.45402000	0.28114800
H	5.64428300	-3.90025600	-0.53888100
H	7.13843100	-4.03231600	0.39394600
O	5.52604500	-3.56516500	1.51576100
H	4.68668000	-3.09326900	1.44311000
H	8.49884600	-2.51445400	1.93404000
H	8.79497800	0.49695400	2.04169700
H	5.95777100	1.32451900	2.08983200

H1

C	2.55739000	1.84690400	-0.41370400
C	1.15630300	1.95789800	0.25149700
C	0.18851200	0.88479200	-0.26200500
C	1.91775900	-0.44769800	-1.20607800
C	3.04890100	0.39785500	-0.60930700
H	1.27849800	1.80874700	1.32951500
H	2.49675300	2.29661300	-1.41460900
H	1.60850700	-0.01262600	-2.16709800
H	-0.13753200	1.12875000	-1.28576200
O	0.81140800	-0.38443400	-0.28376000
O	3.53524400	2.53438400	0.35509300
C	2.20370400	-1.92223200	-1.44504500
H	2.33958300	-2.43288500	-0.48248300
H	3.12273500	-2.02971700	-2.01523200
H	3.14271200	3.35332600	0.73317900
O	1.17717900	-2.53481500	-2.21330000
H	0.34199800	-2.52136400	-1.72700300
C	-2.43939900	-1.09138500	0.36145700
C	-3.80223400	-1.49903000	-0.22873100
C	-2.14367600	0.36700000	0.00671200
H	-2.46948200	-1.18270600	1.45227900
C	-4.91077100	-0.45516800	0.03192400
H	-3.68139900	-1.54992000	-1.31390400
C	-3.30101700	1.23660800	0.50799400
H	-2.08150100	0.47461300	-1.08078200
H	-5.25117200	-0.50044900	1.07063800
H	-3.48578500	1.03735200	1.57452400
O	-4.45169100	0.86893400	-0.25640400
O	-5.95085300	-0.74882700	-0.84912100
O	-0.91369900	0.82356000	0.58907600
C	-3.06458800	2.73227900	0.36235300
H	-4.01115900	3.26093700	0.51277000
H	-2.36500900	3.05543200	1.13245600
O	-2.46772300	3.11118000	-0.88152200
H	-3.10225400	2.98789900	-1.59800000
O	-1.47272100	-1.98814500	-0.17062700
H	-0.59899500	-1.67543700	0.11458000
O	4.13830200	0.42992000	-1.54036300
C	-7.23132300	-0.31202500	-0.57029200
C	-7.53199700	0.71195200	0.32755200
C	-8.25134000	-0.95721900	-1.27196400
C	-8.86605400	1.06885300	0.53214800
H	-6.74354400	1.24326400	0.84426500
C	-9.57464200	-0.58369900	-1.06626100

H	-7.99043200	-1.74748700	-1.96712600
C	-9.89071100	0.42873600	-0.15884800
H	-9.09782900	1.86378500	1.23381500
H	-10.36294500	-1.09073500	-1.61331100
H	-10.92379600	0.71641600	0.00351700
H	3.36203200	-0.02727400	0.34550900
N	-4.21295200	-2.82934000	0.20251800
H	-4.17567200	-3.56278600	-0.48567800
C	-4.74716900	-4.64000100	1.75214100
H	-3.94262000	-5.05994900	2.36043500
H	-5.66920800	-4.72094600	2.32994900
H	-4.84435100	-5.23439500	0.84144600
N	0.50656000	3.25570500	0.00898600
H	-0.40000400	3.23880600	-0.44711800
C	-0.00084400	5.61918900	0.33250000
H	-0.28245900	6.01478200	1.31106200
H	0.55219500	6.40636600	-0.18385100
H	-0.90304300	5.38131700	-0.23216700
C	0.91636600	4.43429900	0.53952800
O	1.97909000	4.55791200	1.15376600
C	-4.45323400	-3.17361200	1.50285100
O	-4.43582300	-2.35446000	2.41269500
C	5.39994500	-0.07632000	-1.18430800
C	6.21267900	0.80142900	-0.21285600
H	5.94242800	-0.10248000	-2.14075100
C	6.49316000	-2.09444700	-0.49031200
C	7.56360200	0.12581200	0.04086200
H	6.37268500	1.77352900	-0.69696100
C	7.37437300	-1.32436500	0.49584300
H	7.02462200	-2.18031400	-1.45061000
H	8.15846200	0.12427400	-0.87845400
H	6.89103400	-1.33389500	1.47909300
O	5.25003000	-1.39869000	-0.69514100
O	8.62914800	-1.99330600	0.55322100
O	8.32729800	0.84954700	0.99777900
O	5.61258800	0.97760100	1.05852000
C	6.12382800	-3.49364900	-0.01262800
H	5.57136500	-4.00667900	-0.81089400
H	7.03488200	-4.05400800	0.19574200
O	5.37049400	-3.47800400	1.19098100
H	4.60780000	-2.90136800	1.05735500
H	9.22151900	-1.44929800	1.08924400
H	7.70291900	1.14742200	1.67740600
H	4.90090000	1.64312900	0.97720000

H2

C	-2.62016800	2.07772300	-0.08271100
C	-1.18938800	2.04023400	-0.68267200
C	-0.19421000	1.28901400	0.21154700
C	-1.97742500	0.12382000	1.34035800
C	-3.07458100	0.70290000	0.43366300
H	-1.23354000	1.47675300	-1.61859800
H	-2.61663700	2.75628200	0.77410900
H	-1.80224300	0.80591900	2.18267200
H	0.02979800	1.83660100	1.13243400
O	-0.78266000	0.02587600	0.55161900
O	-3.49268700	2.63451900	-1.05549400
C	-2.23051700	-1.27136000	1.89528700
H	-2.34164800	-1.97979400	1.06187700
H	-3.15509400	-1.27834300	2.46802800
H	-4.09709700	1.94942700	-1.38922700
O	-1.20192100	-1.68334800	2.78028500
H	-0.35923700	-1.71497300	2.30622900
C	2.29963600	-0.95750100	-0.14017200
C	3.64726300	-1.47959100	0.38033900
C	2.10059600	0.52465700	0.20198600
H	2.27992100	-1.05237900	-1.23158900
C	4.79994400	-0.54621700	-0.02369800
H	3.61429900	-1.47187300	1.47302000
C	3.32590800	1.34492000	-0.22207100
H	1.94110600	0.64748800	1.27748500
H	4.97690300	-0.58034700	-1.10329300
H	3.41113100	1.32180300	-1.31885500
O	4.50020200	0.78536100	0.37750400
O	5.93388800	-0.96014600	0.67919000
O	0.96051200	1.02942000	-0.52179500
C	3.28131100	2.79636100	0.24453700
H	4.14614200	3.32633400	-0.17540000
H	2.37328100	3.27454500	-0.12263300
O	3.25855800	2.90432800	1.65773700
H	4.05079300	2.47116100	2.00171700
O	1.29031300	-1.78399500	0.43046600
H	0.43394500	-1.38414700	0.20704100
O	-4.27528200	0.88752900	1.21360800
C	7.18633400	-0.58786000	0.23845500
C	7.42671500	0.44757900	-0.66512800
C	8.25145800	-1.31492800	0.77488600
C	8.74038400	0.73363700	-1.04091300
H	6.61102900	1.04248900	-1.05431400
C	9.55520000	-1.01220900	0.39913100
H	8.03837400	-2.11206600	1.47852500
C	9.80799100	0.01175800	-0.51533300
H	8.92343500	1.53896800	-1.74526000
H	10.37730800	-1.58282800	0.81934800
H	10.82546300	0.24511400	-0.81013800
H	-3.26030200	0.01494000	-0.39388000
N	3.88981700	-2.86284200	-0.01446200
H	3.80635600	-3.55827100	0.70811500
C	4.12307700	-4.79150500	-1.49085700
H	3.22833000	-5.14974200	-2.00583500
H	4.97777500	-5.00217300	-2.13540000
H	4.23524600	-5.34717100	-0.55752700
N	-0.73277500	3.38223500	-1.03520600
H	-0.82288400	3.63744500	-2.00522200
C	-0.34213600	5.77320700	-0.72223500
H	-1.25700700	6.36311300	-0.61975800
H	0.43754000	6.27391900	-0.14751800
H	-0.05790400	5.75532400	-1.77673100
C	-0.58316200	4.39703600	-0.12903400
O	-0.63646300	4.21488600	1.07858300
C	4.00919600	-3.28958100	-1.30484100
O	4.02671600	-2.52096200	-2.25791000
C	-5.45224600	0.22467200	0.88880100
C	-6.18937900	0.73203700	-0.36508300
H	-6.11608200	0.42834600	1.74144800
C	-6.41766700	-1.95485300	0.73077600
C	-7.46055500	-0.09404600	-0.57562900
H	-6.45694800	1.78174300	-0.19533000
C	-7.14632900	-1.58551800	-0.56936000
H	-7.06668100	-1.72788900	1.59146800
H	-8.17374600	0.12076900	0.23229400
H	-6.49152600	-1.82131500	-1.41204900
O	-5.21526800	-1.17923000	0.81941000
O	-8.39749800	-2.26181500	-0.71322400
O	-8.00817600	0.31798200	-1.82393800
O	-5.36183000	0.63493100	-1.52603500
C	-6.01665000	-3.42204300	0.81178700
H	-5.42491300	-3.57680400	1.72110500
H	-6.91127600	-4.04286700	0.88827200
O	-5.31837400	-3.87344700	-0.34060100
H	-4.46290200	-3.42829600	-0.38018300
H	-8.24677200	-3.13486500	-1.09391900
H	-8.71616800	-0.29762000	-2.05665900
H	-5.95071300	0.69037100	-2.29434300

I1

C	2.61431200	1.92917700	-0.50577400
C	1.23390700	2.36039000	0.06066500
C	0.13580900	1.43740500	-0.48741900
C	1.58787200	-0.33155200	-0.98490400
C	2.83629500	0.39672300	-0.47134500
H	1.25937200	2.24019700	1.14960100
H	2.67362600	2.23851200	-1.55847400
H	1.42651300	-0.04742800	-2.03581400
H	0.03105800	1.56137800	-1.57410300
O	0.47887300	0.10491100	-0.20027500
O	3.67788500	2.51545200	0.22624800
C	1.58134700	-1.85135600	-0.91967000
H	1.63808800	-2.18534400	0.12040100
H	2.43685600	-2.25451600	-1.45905200
H	3.56686800	3.49319500	0.19507000
O	0.37923100	-2.32917200	-1.53786000
H	-0.22247100	-2.72357600	-0.88727700
C	-2.30056900	-0.15189100	-0.91456600
C	-3.73979500	-0.63779500	-1.18039000
C	-2.30475900	1.31415200	-0.45094100
H	-1.85118800	-0.75659300	-0.13047700
C	-4.68576700	-0.29646900	-0.02328000
H	-4.11078400	-0.08595500	-2.04968600
C	-3.34480700	1.55198200	0.65526200
H	-2.52087700	1.96088100	-1.30837600
H	-4.42269300	-0.85521500	0.88685000
H	-3.02932100	0.99813700	1.55227400
O	-4.63391500	1.09698800	0.23399000
O	-5.98219100	-0.61847500	-0.43484200
O	-1.06541500	1.74271000	0.15414200
C	-3.51161800	3.02014400	1.03092500
H	-4.26818400	3.09398700	1.82162000
H	-2.57058200	3.40573000	1.42149800
O	-3.85590100	3.83868300	-0.07780000
H	-4.71655700	3.55444100	-0.41109600
O	-1.56707300	-0.27966000	-2.12208000
H	-0.89256700	-0.97752800	-2.01726400
O	3.93458900	0.09649300	-1.34370900
C	-6.98150100	-0.78684500	0.50494700
C	-6.89581800	-0.35212200	1.82728700
C	-8.13366200	-1.42880800	0.04694400
C	-7.96687100	-0.58601000	2.69191000
H	-6.02544200	0.18627500	2.17836200
C	-9.19651200	-1.64556500	0.91658600
H	-8.17982700	-1.75026700	-0.98780800
C	-9.11741800	-1.22998200	2.24668900
H	-7.89712200	-0.24747900	3.72067400
H	-10.08860800	-2.14578900	0.55384200
H	-9.94596500	-1.40225500	2.92496700
H	3.05332600	0.08232900	0.55098100
N	-3.82341600	-2.05509300	-1.53560500
H	-4.58410500	-2.28457100	-2.15463700
C	-3.41877800	-4.44775900	-1.69233800
H	-2.57345100	-4.80528400	-2.28486600
H	-3.56584300	-5.15542700	-0.87467200
H	-4.30988900	-4.44136000	-2.32284600
N	0.81826600	3.73299600	-0.27977000
H	-0.18595300	3.83161700	-0.33983600
C	0.73135300	6.15444200	-0.48740400
H	0.81415400	6.79388600	0.39423000
H	1.18135300	6.69552100	-1.32170600
H	-0.32444700	5.98230100	-0.70270400
C	1.52813400	4.88917700	-0.24297300
O	2.74249700	4.95469400	-0.04223300
C	-3.05947700	-3.09494600	-1.10663600
O	-2.12468700	-2.98501300	-0.31355000
C	5.07522300	-0.56259800	-0.85975500
C	6.02119200	0.29396800	0.00422800
H	5.62085100	-0.83433200	-1.77539500
C	5.77262800	-2.61305400	0.17013900
C	7.22612600	-0.56386500	0.40133300
H	6.35827400	1.13912400	-0.60986900
C	6.77392000	-1.87106900	1.05878100
H	6.28692800	-2.94411200	-0.74521000
H	7.81818400	-0.80927300	-0.48641800
H	6.29243100	-1.64349100	2.01629200
O	4.68014100	-1.74656700	-0.18407500
O	7.88575100	-2.73748600	1.25723500
O	8.10025100	0.14879000	1.26823200
O	5.45108500	0.76095900	1.21431100
C	5.14731900	-3.82798200	0.84479400
H	4.51428000	-4.35116600	0.11576000
H	5.93646400	-4.50530500	1.17038700
O	4.40107900	-3.48321800	2.00283000
H	3.78238700	-2.78260500	1.76034900
H	8.56394100	-2.23237400	1.72533700
H	7.53354400	0.65203900	1.87329100
H	4.86733000	1.51833100	1.01033900

I2

C	2.63286900	2.10316500	-0.23873500
C	1.23492300	2.43046600	0.35307400
C	0.13036300	1.61445100	-0.33086500
C	1.65790100	-0.05293500	-1.05456300
C	2.87793700	0.58853500	-0.37464800
H	1.23229800	2.12563800	1.40320900
H	2.68250100	2.52774000	-1.24562900
H	1.53817100	0.39324500	-2.05303500
H	-0.00094600	1.89722000	-1.37711500
O	0.52246100	0.24335400	-0.25271200
O	3.61726100	2.76364300	0.54634200
C	1.67578900	-1.56680800	-1.21995300
H	1.73106300	-2.04863500	-0.23838700
H	2.54499800	-1.87039800	-1.80272800
H	4.16047800	2.10904500	1.01659700
O	0.49698600	-1.98034300	-1.91678300
H	-0.11848600	-2.44585600	-1.32777200
C	-2.26764400	0.00748700	-0.92366500
C	-3.69689000	-0.49761200	-1.19535700
C	-2.28944300	1.40521200	-0.27464900
H	-1.76752700	-0.67202900	-0.23756800
C	-4.59581800	-0.34541900	0.03542000
H	-4.12675800	0.14523600	-1.96991900
C	-3.32195300	1.49576500	0.86129200
H	-2.50833600	2.15403400	-1.04360000
H	-4.25675000	-0.98821100	0.86153500
H	-2.95339400	0.89165100	1.70366300
O	-4.60395600	1.00758600	0.44528400
O	-5.89680800	-0.69687500	-0.34719400
O	-1.05654300	1.74743600	0.38386400
C	-3.56397500	2.92005600	1.35139700
H	-4.21524800	2.88153400	2.23474300
H	-2.61485500	3.37110400	1.63846600
O	-4.12511700	3.75266600	0.34928000
H	-4.95417200	3.35248300	0.05666800
O	-1.58613500	0.05079900	-2.16671200
H	-0.84651600	-0.58263400	-2.15550200
O	4.04445600	0.39200700	-1.20657200
C	-6.83291900	-1.01586100	0.61520300
C	-6.70213900	-0.70828000	1.96974900
C	-7.97168100	-1.67793300	0.15082700
C	-7.71143300	-1.08924900	2.85585000
H	-5.84569400	-0.15412500	2.33017300
C	-8.97349800	-2.04185800	1.04335300

H	-8.05519100	-1.89859200	-0.90775400
C	-8.84708800	-1.75460000	2.40346100
H	-7.60644500	-0.84802800	3.90886500
H	-9.85498400	-2.55637900	0.67442000
H	-9.62807100	-2.04121400	3.09940300
H	3.03023900	0.12961900	0.60459200
N	-3.74926600	-1.86093500	-1.72844800
H	-4.53209500	-2.03620500	-2.33747300
C	-3.26076000	-4.17880300	-2.26493100
H	-2.46201500	-4.37128700	-2.98547000
H	-3.28090300	-5.01720200	-1.56700000
H	-4.21081500	-4.13763600	-2.80139900
N	0.98372100	3.86872400	0.34574200
H	1.15180100	4.35315900	1.21174000
C	0.72104200	6.12082300	-0.56087700
H	1.53039400	6.62117900	-1.09682600
H	-0.22015800	6.46367000	-0.99506200
H	0.75373200	6.42006200	0.48896700
C	0.84155300	4.62366200	-0.78331400
O	0.79390900	4.13829100	-1.90554700
C	-2.92238600	-2.91252900	-1.49837700
O	-1.94788300	-2.88212200	-0.74566300
C	5.14127900	-0.32283500	-0.74803700
C	6.02949500	0.39224300	0.28736500
H	5.76326400	-0.46371900	-1.64406000
C	5.81769000	-2.49496000	-0.01329500
C	7.19856900	-0.51990300	0.66478600
H	6.41346000	1.30906600	-0.17568300
C	6.69768400	-1.89422700	1.09334700
H	6.41837700	-2.62276000	-0.92796100
H	7.86155700	-0.64011600	-0.20337700
H	6.09618100	-1.79200600	1.99989200
O	4.72680000	-1.60393700	-0.27533200
O	7.86054400	-2.68287500	1.36045800
O	7.89913600	0.13161900	1.71949400
O	5.28574800	0.72669200	1.46039900
C	5.21394700	-3.84435400	0.35401700
H	4.54344800	-4.16245800	-0.45264600
H	6.00555000	-4.59087200	0.44138200
O	4.54211200	-3.82983400	1.60606800
H	3.78503700	-3.23401700	1.54399500
H	7.63648200	-3.38121300	1.98633200
H	8.54295400	-0.49303500	2.07952300
H	5.93194100	0.92136500	2.15611600

J1

C	2.47745300	1.77122700	-0.56199000
C	1.11423500	1.87057700	0.16589400
C	0.17453700	0.75440100	-0.30653500
C	1.96694800	-0.67551900	-1.02440200
C	3.04368700	0.33886600	-0.60891900
H	1.27582000	1.74499300	1.24217200
H	2.33153100	2.09673500	-1.60206700
H	1.68697400	-0.49451500	-2.07254200
H	-0.09361400	0.90987400	-1.36420800
O	0.80324400	-0.51373600	-0.18491500
O	3.45951300	2.59646500	0.05014300
C	2.37243400	-2.13691800	-0.88184000
H	3.26823700	-2.32400600	-1.46751600
H	1.56164700	-2.77013300	-1.26362300
H	3.03345300	3.37339300	0.47805300
O	2.69048300	-2.49497100	0.45843400
H	1.92339900	-2.34854300	1.02528000
C	-2.49167000	-1.11112600	-0.07192200
C	-3.90621100	-1.41611400	-0.60196900
C	-2.21205600	0.39070300	-0.14115800
H	-2.41381700	-1.43018000	0.97264700
C	-4.98000300	-0.44977300	-0.06394400
H	-3.88009000	-1.26808400	-1.68456800
C	-3.33485500	1.15933900	0.56087600
H	-2.18307000	0.70346800	-1.19014000
H	-5.22238300	-0.67562000	0.97867100
H	-3.44123200	0.80624900	1.59760800
O	-4.53834400	0.90566700	-0.16611300
O	-6.09925300	-0.59498600	-0.88291500
O	-0.97013100	0.75248500	0.48435800
C	-3.11175400	2.66383500	0.60201300
H	-4.03771600	3.15426000	0.91836800
H	-2.33409000	2.89269800	1.32936600
O	-2.65471000	3.20528100	-0.64065400
H	-3.35749800	3.13313800	-1.29846900
O	-1.59841300	-1.85826900	-0.88701500
H	-0.69458600	-1.70129500	-0.56868100
O	4.07137200	0.32592300	-1.61270000
C	-7.34411600	-0.21279300	-0.42293400
C	-7.55588100	0.59883300	0.69123500
C	-8.42717000	-0.68487700	-1.16698300
C	-8.86311900	0.91596800	1.06505200
H	-6.72193600	1.00068600	1.25162500
C	-9.72307600	-0.35230600	-0.78911100
H	-8.23566500	-1.31073300	-2.03159400
C	-9.94958200	0.44676300	0.33276100
H	-9.02471000	1.54497600	1.93463500
H	-10.56002900	-0.72443600	-1.37118500
H	-10.96120300	0.70238400	0.62876500
H	3.44441800	0.05712900	0.36473300
N	-4.28123700	-2.80798600	-0.38536200
H	-4.30143500	-3.40504500	-1.19521300
C	-4.69017600	-4.87355300	0.85319900
H	-3.85389300	-5.38543400	1.33484600
H	-5.57875700	-5.06145000	1.45820200
H	-4.83939800	-5.30233300	-0.13982800
N	0.41961200	3.14695000	-0.07644700
H	-0.51865700	3.09834200	-0.45999700
C	-0.16377400	5.49137700	0.28272900
H	-0.37792100	5.91152600	1.26787400
H	0.32950000	6.27448300	-0.29743100
H	-1.09851400	5.21360100	-0.20375400
C	0.79909500	4.33821100	0.45432700
O	1.87523800	4.49648300	1.03567000
C	-4.40968200	-3.38344800	0.84682200
O	-4.31020700	-2.74124100	1.88475300
C	5.37818900	-0.09846600	-1.29389200
C	6.19352400	0.88360600	-0.42621500
H	5.87240100	-0.15550500	-2.27566200
C	6.54790700	-2.02388100	-0.42345900
C	7.55743200	0.25852500	-0.12240600
H	6.33122000	1.80255100	-1.01131300
C	7.39394400	-1.13563200	0.48835000
H	7.10429300	-2.19797400	-1.35845400
H	8.14229100	0.16569600	-1.04356400
H	6.89518800	-1.05451600	1.46038100
O	5.29674400	-1.38559500	-0.72396000
O	8.66574500	-1.76069600	0.63084800
O	8.32136000	1.09415900	0.74019300
O	5.61372300	1.18622300	0.83012100
C	6.19460500	-3.37596300	0.19510400
H	5.70573700	-3.99455500	-0.57109200
H	7.11802700	-3.87336600	0.49449100
O	5.38618100	-3.26208900	1.34743300
H	4.51137000	-2.93086200	1.08610300
H	9.23848700	-1.13914500	1.09970600
H	7.70080100	1.44998200	1.39506900
H	4.88124500	1.81882300	0.69144600

J2

C	2.58821100	2.01442500	-0.39547300
C	1.19293100	1.99894300	0.26630100
C	0.23075400	1.00645200	-0.39598800
C	2.09878500	-0.29905100	-1.25622000
C	3.14454900	0.59474300	-0.56217900
H	1.31305400	1.66693500	1.30160200
H	2.49435700	2.45315600	-1.39296100
H	1.91619500	0.08775000	-2.26883900
H	-0.05376900	1.33565100	-1.39800200
O	0.87568100	-0.28120700	-0.49657700
O	3.43482500	2.86667100	0.36042900
C	2.47041700	-1.77345000	-1.36225300
H	3.42643500	-1.88246100	-1.86589100
H	1.70105800	-2.29124000	-1.94774500
H	4.10367400	2.33433000	0.82506100
O	2.61026300	-2.39764800	-0.08533900
H	1.79404300	-2.26752800	0.41420900
C	-2.37923100	-0.87403900	-0.47129300
C	-3.82734800	-1.14324100	-0.91328200
C	-2.15536400	0.61832700	-0.20116500
H	-2.17993500	-1.41822800	0.45855600
C	-4.84574000	-0.46298400	0.01733600
H	-3.96304800	-0.69173200	-1.89957400
C	-3.22556500	1.13928400	0.76571400
H	-2.20915800	1.18419600	-1.13682700
H	-4.87850400	-0.95781800	0.99302400
H	-3.14156400	0.59953500	1.72123400
O	-4.51377700	0.91113600	0.18428800
O	-6.08933900	-0.52770400	-0.61603500
O	-0.88256200	0.85055200	0.42248600
C	-3.14401700	2.63334200	1.05086200
H	-3.91039300	2.88779500	1.79434200
H	-2.16449200	2.86892300	1.46499200
O	-3.29123700	3.42758000	-0.11633100
H	-4.13973700	3.21113700	-0.52445800
O	-1.54342200	-1.36309800	-1.51276400
H	-0.62110200	-1.26126600	-1.22531000
O	4.31634400	0.68316300	-1.40506400
C	-7.24382100	-0.36609600	0.12034100
C	-7.28636100	0.14850600	1.41634200
C	-8.42350000	-0.74463500	-0.52474900
C	-8.51683900	0.26073600	2.06608200
H	-6.38283300	0.48163000	1.90950100
C	-9.64270300	-0.61781000	0.13093900
H	-8.36474100	-1.13659200	-1.53415100
C	-9.69707100	-0.11834000	1.43335400
H	-8.54506700	0.65927700	3.07530200
H	-10.55451100	-0.91545100	-0.37707500
H	-10.64869300	-0.02282900	1.94507400
H	3.40215600	0.15481400	0.40133000
N	-4.09798500	-2.56783300	-1.07281000
H	-4.17540300	-2.90809300	-2.01664700
C	-4.22781400	-4.93107900	-0.47659000
H	-3.31200800	-5.48432400	-0.25606100
H	-5.02489500	-5.36543600	0.12927400
H	-4.47259800	-5.06386600	-1.53248000
N	0.61238000	3.33710000	0.32525300
H	0.72103400	3.83287700	1.19453300
C	-0.29810900	5.44858800	-0.48916900
H	-0.00223200	6.11622200	-1.29809400
H	-1.39015100	5.39277100	-0.47332100
H	0.04295200	5.86163900	0.46335600
C	0.23620500	4.06006500	-0.77518300
O	0.27197700	3.59860100	-1.90842800
C	-4.03411900	-3.48353300	-0.06384400
O	-3.84114000	-3.16968400	1.10420900
C	5.53436500	0.15126900	-0.97915400
C	6.24377400	0.92502600	0.15221500
H	6.18599600	0.23238900	-1.86172700
C	6.49918500	-1.95678800	-0.28198300
C	7.48578300	0.15398200	0.60666800
H	6.54291000	1.90005400	-0.25099300
C	7.13264500	-1.28984000	0.94424600
H	7.22953800	-1.96671500	-1.10733100
H	8.23528700	0.15593300	-0.19709600
H	6.41019200	-1.31313700	1.76430800
O	5.33817900	-1.21411600	-0.66272400
O	8.35757300	-1.91333400	1.34368000
O	7.99819900	0.84568200	1.74205900
O	5.38449200	1.12633200	1.27567700
C	6.01557400	-3.38430100	-0.03235500
H	5.60993300	-3.78167600	-0.97185200
H	6.86525700	-4.01434900	0.24459000
O	5.07103000	-3.46451400	1.01220200
H	4.23197500	-3.07448900	0.70568900
H	8.16260600	-2.67377100	1.90307300
H	8.65720400	0.27462000	2.15989900
H	5.95809000	1.33616500	2.02911300

K1

C	-2.57441700	1.12165600	-0.05161500
C	-1.09069100	1.56905200	-0.28443700
C	-0.09292200	0.41820500	-0.11666300
C	-1.68691000	-1.27988500	-0.28852300
C	-2.88439400	-0.34291500	-0.46451300
H	-1.01503900	1.90698500	-1.32342000
H	-2.77986800	1.19604100	1.02544200
H	-1.51438800	-1.44123600	0.78600700
H	-0.00147400	0.12719400	0.94208800
O	-0.52968000	-0.67904800	-0.86638400
O	-3.44582300	1.96574700	-0.78607800
C	-1.80596200	-2.64083300	-0.95791600
H	-1.89822800	-2.49563500	-2.04459600
H	-2.69615700	-3.16187100	-0.60182000
H	-3.15385400	2.89049900	-0.64671800
O	-0.69145500	-3.45362700	-0.64457700
H	0.11218100	-2.90627700	-0.65815500
C	2.76532500	-1.05458000	-0.34889000
C	4.19474800	-1.34679500	0.17300400
C	2.32351400	0.35940100	0.06580200
H	2.76843200	-1.10912000	-1.44643600
C	5.14705700	-0.18074400	-0.12489600
H	4.15138500	-1.45555800	1.26209400
C	3.39889800	1.40429700	-0.24990200
H	2.14625800	0.35481200	1.14632800
H	5.30020900	-0.07087600	-1.21257600
H	3.55451400	1.45220100	-1.33895400
O	4.62366000	1.02605300	0.38878900
O	6.36005800	-0.45518000	0.50417800
O	1.15422400	0.82450600	-0.60494800
C	3.02906100	2.80457000	0.22612300
H	3.91152400	3.44909900	0.15430400
H	2.25440500	3.20631600	-0.42464900
O	2.48480300	2.83574500	1.54492800
H	3.17715600	2.62921100	2.18478900
O	1.83190000	-1.99635400	0.16114600
H	2.30167100	-2.74893800	0.57452500
O	-3.92764700	-0.90926600	0.35314800
C	7.50759500	0.21827600	0.11773400
C	7.51872400	1.35928000	-0.68340500
C	8.70198700	-0.32327200	0.59581200
C	8.74286200	1.94156900	-1.01961000
H	6.59581300	1.81462500	-1.01728300
C	9.91200500	0.27387200	0.26125800
H	8.66379200	-1.20441400	1.22671000
C	9.93986600	1.40701300	-0.55344400
H	8.75024500	2.82998200	-1.64273900
H	10.83735200	-0.15028200	0.63696600
H	10.88473100	1.87062300	-0.81480400
H	-3.20382500	-0.33248900	-1.51170600
N	4.76203200	-2.57611100	-0.38835800
H	5.59618900	-2.48277600	-0.94836900
C	5.35936300	-4.94978200	-0.33919100
H	5.93525800	-5.32227500	0.51146800
H	4.71940900	-5.76469000	-0.68026300
H	6.04989100	-4.67762600	-1.13957200
N	-0.66820800	2.65541300	0.61415300
H	0.20567700	2.52588700	1.10908200
C	-0.47018100	4.93156500	1.47263700
H	-0.28082600	5.83287000	0.88704900
H	-1.14751800	5.21195700	2.28323600
H	0.46691200	4.57069000	1.89794100
C	-1.16721900	3.91968400	0.58759400
O	-2.14307000	4.23975700	-0.09048000
C	4.48881300	-3.80714300	0.13009200
O	3.57662400	-3.98022900	0.93469800
C	-5.21232400	-0.98836100	-0.19158600
C	-6.03832500	-1.93909200	0.68789700
H	-5.17059200	-1.38765000	-1.22078400
C	-7.07926300	0.36827200	-0.76340100
C	-7.51507200	-1.86563700	0.28356800
H	-5.67003000	-2.96470900	0.54887200
C	-8.01699500	-0.41850700	0.15021300
H	-7.07915000	-0.07976600	-1.76896300
H	-7.64319800	-2.35239200	-0.68897000
H	-8.01568700	0.05241700	1.14235900
O	-5.76179600	0.29895700	-0.20759200
O	-9.32454800	-0.40151900	-0.40590700
O	-8.33792000	-2.59848300	1.18651700
O	-5.97170800	-1.58409900	2.06393800
C	-7.36264900	1.85916800	-0.89047600
H	-8.32360200	2.01803400	-1.38045900
H	-7.40975900	2.30062500	0.11576800
O	-6.37144100	2.48822300	-1.67968900
H	-5.49808100	2.25028600	-1.32334900
H	-9.86230600	-1.02961700	0.09524500
H	-8.02515900	-2.41094800	2.08341000
H	-5.05545300	-1.37578700	2.28724600

K2

C	-2.58486500	1.12865000	-0.09390000
C	-1.12139800	1.54061000	-0.46067300
C	-0.11428700	0.43505900	-0.13205800
C	-1.69433800	-1.29396500	-0.05696700
C	-2.89830300	-0.37538600	-0.30308000
H	-1.08702700	1.70306400	-1.54355500
H	-2.73178700	1.33818100	0.97543100
H	-1.50892500	-1.35359400	1.02564500
H	-0.02799600	0.30258500	0.95838700
O	-0.54016600	-0.78089100	-0.70817700
O	-3.50849700	1.85623200	-0.88050600
C	-1.88795200	-2.70303800	-0.58323800
H	-1.97053700	-2.67623100	-1.67641800
H	-2.80231200	-3.13250100	-0.16159800
H	-3.25856800	2.80354600	-0.82291700
O	-0.74109700	-3.45384700	-0.17949000
H	-0.75291100	-4.32297900	-0.59479600
C	2.67294700	-1.10741800	-0.14556900
C	4.08014800	-1.37592600	0.43672700
C	2.28940100	0.36418500	0.05507200
H	2.66692500	-1.32900800	-1.21640200
C	5.11479900	-0.27960400	0.12418200
H	3.97017000	-1.38031200	1.52411200
C	3.41691300	1.29273200	-0.40328500
H	2.11664600	0.53574500	1.12326700
H	5.44067300	-0.32676200	-0.91954600
H	3.64361800	1.11193300	-1.46486100
O	4.56873500	1.01040600	0.39302900
O	6.19066700	-0.48195000	0.99169400
O	1.12259700	0.76740900	-0.67271600
C	3.07715200	2.76956800	-0.24422900
H	3.98615600	3.36273600	-0.38779000
H	2.35332700	3.05657000	-1.00531900
O	2.46593700	3.08166500	1.01030400
H	3.10720000	2.94376900	1.71858000
O	1.80859900	-1.98621400	0.56176200
H	1.03065900	-2.22521800	0.03398900
O	-3.91272300	-0.82398600	0.61910000
C	7.42757900	0.04866900	0.68655000
C	7.63586300	1.07490500	-0.23492100
C	8.50507300	-0.50024300	1.38500700
C	8.93482100	1.53135100	-0.46528400
H	6.80119100	1.53252800	-0.74940100
C	9.79208900	-0.02838300	1.15322800
H	8.31593300	-1.29426200	2.09896300
C	10.01581000	0.98756600	0.22245600
H	9.09396400	2.32837100	-1.18465200
H	10.62492600	-0.46078000	1.69851400
H	11.02081800	1.35241100	0.03990700
H	-3.25807800	-0.49254600	-1.33019600
N	4.57237300	-2.69846700	0.07003100
H	4.58876500	-3.39528600	0.79570400
C	5.27915200	-4.53235500	-1.37893600
H	4.60811400	-5.01622200	-2.09144400
H	6.28140900	-4.55197700	-1.81195300
H	5.28555400	-5.10749200	-0.45076800
N	-0.66123500	2.75196700	0.23943900
H	0.28259000	2.72129800	0.60977600
C	-0.45735300	5.11749300	0.79337400
H	-0.35915800	5.93216900	0.07345000
H	-1.04842700	5.49848000	1.62985900
H	0.53068800	4.82869800	1.15210300
C	-1.22105600	3.98677100	0.13684500
O	-2.29420700	4.19965800	-0.42860700
C	4.84995900	-3.08693300	-1.20784400
O	4.77265300	-2.32203700	-2.16143300
C	-5.19322300	-1.06001500	0.10444000
C	-6.00852900	-1.78396300	1.18471100
H	-5.13437700	-1.69862400	-0.79627900
C	-7.07640400	0.08307200	-0.79180100
C	-7.47910400	-1.85084600	0.75721400
H	-5.61601700	-2.80299800	1.30850500
C	-8.01282200	-0.49350700	0.26934500
H	-7.05665400	-0.58128000	-1.66933400
H	-7.57899400	-2.56320200	-0.06850200
H	-8.04976200	0.20107500	1.11942400
O	-5.76372900	0.17075600	-0.22719300
O	-9.30515200	-0.65040800	-0.29954200
O	-8.29925500	-2.36444300	1.80205100
O	-5.97102300	-1.09814500	2.42960300
C	-7.38644400	1.50228000	-1.25323000
H	-8.33595800	1.52276800	-1.78848600
H	-7.47140600	2.15393700	-0.37179700
O	-6.38731300	1.96848400	-2.13916600
H	-5.52386600	1.88021000	-1.70101700
H	-9.84585300	-1.14217700	0.33332500
H	-8.01696100	-1.94503700	2.62781700
H	-5.07369100	-0.77556800	2.58345600

K3

C	2.62237200	1.04201600	0.24809600
C	1.14989100	1.44552300	0.61035200
C	0.10311600	0.42342100	0.17207500
C	1.67014100	-1.27759600	-0.29645100
C	2.89532100	-0.48150000	0.16440500
H	1.09683700	1.51527100	1.70126300
H	2.83790600	1.44933300	-0.74924100
H	1.47053200	-1.04749600	-1.35266000
H	-0.06062500	0.47828600	-0.91565000
O	0.53937000	-0.88585800	0.49070700
O	3.50431200	1.59076000	1.21248100
C	1.79404500	-2.78929700	-0.16467000
H	1.83525800	-3.05955100	0.89967200
H	2.71668200	-3.12193300	-0.63953300
H	3.24231000	2.52339200	1.35575300
O	0.74386900	-3.47056000	-0.83101000
H	-0.10836700	-3.23430600	-0.43951800
C	-2.83897400	-1.05707500	0.83762100
C	-4.15107600	-1.46039100	0.13939300
C	-2.28653300	0.21394900	0.19235700
H	-3.03351200	-0.85287500	1.89555300
C	-5.11811400	-0.27160300	-0.06697300
H	-3.88833400	-1.81510200	-0.86052300
C	-3.35991700	1.30383400	0.26152500
H	-2.06366600	0.02092000	-0.86155900
H	-5.61149300	-0.00545800	0.87248900
H	-3.72093400	1.41061500	1.29577100
O	-4.43257400	0.87410100	-0.58034300
O	-6.04857600	-0.67714300	-1.02346100
O	-1.09179400	0.66748800	0.84583800
C	-2.88358200	2.67555500	-0.19298300
H	-3.75232300	3.32371900	-0.34549000
H	-2.26562700	3.11036800	0.59182700
O	-2.05956400	2.65151500	-1.36223400
H	-2.59855600	2.44533100	-2.13546700
O	-1.94762800	-2.16027600	0.71707600
H	-1.08475000	-1.87134200	1.05379300
O	3.91770500	-0.77072300	-0.80835500
C	-7.27509000	-0.04623100	-1.09916800
C	-7.55288700	1.19822000	-0.53433600
C	-8.26012300	-0.73359200	-1.81079800
C	-8.83322500	1.73784100	-0.67044100
H	-6.78324400	1.75500900	-0.01610200
C	-9.52766400	-0.17886100	-1.94767900
H	-8.01715300	-1.69748700	-2.24410100
C	-9.82374500	1.05848200	-1.37349400
H	-9.04778300	2.70505200	-0.22723900
H	-10.28912800	-0.71913000	-2.50090100
H	-10.81414700	1.48822300	-1.47755500
H	3.21714800	-0.82304600	1.15345300
N	-4.81332800	-2.57229700	0.80998800
H	-4.80260200	-3.46101000	0.33778600
C	-5.83287100	-3.82784600	2.63988800
H	-5.18974300	-4.16452100	3.45650200
H	-6.82277200	-3.64251700	3.05961500
H	-5.90403000	-4.62618400	1.89836200
N	0.76182600	2.73365200	0.02284300
H	-0.03978900	2.74302900	-0.59840800
C	0.65675500	5.16102800	-0.19962800
H	0.27148300	5.78602200	0.60927600
H	1.42938700	5.73833500	-0.71107100
H	-0.15162800	4.94225900	-0.89889000
C	1.28907700	3.92530700	0.40404200
O	2.23167600	4.01257700	1.19093500
C	-5.28206900	-2.52557400	2.09247000
O	-5.26162700	-1.50051100	2.76169100
C	5.19622400	-1.08437100	-0.33028800
C	6.02073300	-1.62006900	-1.51036000
H	5.13364000	-1.86153500	0.45305000
C	7.06957700	-0.09878600	0.75165500
C	7.49086400	-1.74584100	-1.09367600
H	5.63479300	-2.60732600	-1.79928000
C	8.01088800	-0.48133500	-0.38901300
H	7.04859200	-0.90363300	1.50198700
H	7.59359700	-2.58222100	-0.39444600
H	8.03592300	0.34430800	-1.11283400
O	5.75863600	0.07820400	0.20279200
O	9.30644600	-0.71417800	0.14465200
O	8.31899200	-2.07540400	-2.20398400
O	5.98565300	-0.74046400	-2.62733400
C	7.37331400	1.22112200	1.44969800
H	8.32311500	1.15319200	1.98045700
H	7.45565400	2.01478000	0.69301100
O	6.37290200	1.52479300	2.40226100
H	5.50822600	1.49789600	1.95829100
H	9.84783000	-1.09799900	-0.55848200
H	8.03269300	-1.53372600	-2.95366000
H	5.07804000	-0.43895900	-2.76052200

K4

C	2.55215500	1.33321100	-0.04648300
C	1.12317600	1.96737200	0.06171300
C	0.02817600	0.98929200	-0.37033500
C	1.32994500	-0.93096800	-0.13155200
C	2.61214500	-0.18965700	0.26165300
H	0.94929700	2.20407400	1.11681400
H	2.89514500	1.45950700	-1.08288000
H	1.28892200	-0.99025600	-1.22945600
H	0.08234200	0.77570400	-1.44777800
O	0.20360600	-0.21119100	0.34626000
O	3.43309500	1.97850900	0.85518700
C	1.19572800	-2.34705500	0.41131500
H	1.12926400	-2.32347700	1.50370200
H	2.06770300	-2.93883700	0.12559600
H	3.33212700	2.94457500	0.72570300
O	0.03652700	-2.96227900	-0.15499800
H	-0.67256400	-3.06563700	0.49985700
C	-2.48001400	-0.53712000	-0.64056400
C	-3.91582600	-1.01406700	-0.93543600
C	-2.39701300	0.99744100	-0.67619700
H	-2.18164500	-0.86916300	0.35090100
C	-4.95563900	-0.25641500	-0.10137700
H	-4.12894200	-0.77036800	-1.98097600
C	-3.54310400	1.64902100	0.11384100
H	-2.42787400	1.33367500	-1.71838700
H	-4.85318200	-0.48323400	0.97018000
H	-3.39578100	1.42720400	1.18144000
O	-4.80728700	1.13600800	-0.31697100
O	-6.21879500	-0.64105400	-0.56066200
O	-1.21402100	1.53257900	-0.04560300
C	-3.61475700	3.16230700	-0.05860400
H	-4.44110700	3.54527900	0.55263000
H	-2.68815700	3.61223700	0.29560600
O	-3.76340200	3.55560000	-1.41521200
H	-4.60724900	3.21709700	-1.74061000
O	-1.62251000	-1.08938100	-1.62847700
H	-1.05282300	-1.76620600	-1.21887300
O	3.66499200	-0.83083300	-0.48596400
C	-7.33312100	-0.42950200	0.22946300
C	-7.36568800	0.43397700	1.32412500
C	-8.47547500	-1.14101800	-0.14202500
C	-8.54804500	0.56160900	2.05567100
H	-6.49853400	1.02363900	1.59073600
C	-9.64879700	-0.99579500	0.58947800
H	-8.42760800	-1.79950900	-1.00244700
C	-9.69063000	-0.14725900	1.69689300
H	-8.57065600	1.23382600	2.90738900
H	-10.53301400	-1.55171600	0.29493200
H	-10.60566400	-0.03670600	2.26847500
H	2.80227100	-0.30036800	1.33406900
N	-4.09076900	-2.46284400	-0.81540200
H	-4.80821800	-2.84359500	-1.41121500
C	-3.90889400	-4.78654900	-0.12445300
H	-3.05082800	-5.36947800	-0.46740000
H	-4.19289900	-5.17089200	0.85659500
H	-4.73580500	-4.93993600	-0.82034400
N	0.95926400	3.18173200	-0.75255900
H	0.07920400	3.24454700	-1.24414100
C	1.12715600	5.53445200	-1.37881300
H	0.80660000	6.30491300	-0.67371000
H	1.94732300	5.95350300	-1.96407000
H	0.29610100	5.29902600	-2.04581100
C	1.64455000	4.34630600	-0.59499000
O	2.63503700	4.45227800	0.12566300
C	-3.48012500	-3.33879100	0.02537900
O	-2.62230300	-3.02343200	0.85065500
C	4.83912300	-1.16466200	0.19775300
C	5.65529700	-2.10357300	-0.70280300
H	4.59966600	-1.68425000	1.14332600
C	6.75947200	-0.17645300	1.19169900
C	7.05454100	-2.30022400	-0.10785200
H	5.14316500	-3.07367800	-0.76566800
C	7.71166800	-0.97126200	0.29969200
H	6.55784200	-0.74086500	2.11503100
H	6.97636500	-2.92142900	0.79052900
H	7.91650300	-0.38338300	-0.60511700
O	5.53662200	0.01548000	0.47185400
O	8.91404700	-1.21706100	1.01533100
O	7.89924700	-3.02358700	-0.99755700
O	5.83526300	-1.56673900	-2.00749700
C	7.20922700	1.23091800	1.56271700
H	8.09825400	1.18627000	2.19214400
H	7.46235000	1.77927900	0.64381900
O	6.20058400	1.89926300	2.29566400
H	5.37235100	1.85547600	1.78798200
H	9.43847100	-1.84363400	0.49841700
H	7.75280900	-2.67348800	-1.88832600
H	5.00202400	-1.17843900	-2.30377800

K5

C	-2.58229300	1.10371800	-0.12524600
C	-1.09800900	1.54968200	-0.35445300
C	-0.10157800	0.39956900	-0.18332300
C	-1.68639900	-1.30249400	-0.36052500
C	-2.89668600	-0.37325300	-0.49851300
H	-1.01727300	1.88498800	-1.39375200
H	-2.80298200	1.20599400	0.94492100
H	-1.50164100	-1.48066300	0.70839000
H	-0.01832000	0.10275500	0.87436700
O	-0.53573900	-0.69057800	-0.94281800
O	-3.44178300	1.93888700	-0.89292400
C	-1.81475000	-2.65090500	-1.05200500
H	-1.93196500	-2.48664600	-2.13373600
H	-2.69540900	-3.17974000	-0.68378700
H	-3.13618300	2.86264000	-0.77892400
O	-0.69067200	-3.46737700	-0.78154300
H	0.10805500	-2.91336600	-0.78389100
C	2.77240600	-1.06081100	-0.40033000
C	4.19164900	-1.35138700	0.14911300
C	2.31187000	0.34384900	0.02595300
H	2.80115200	-1.09719700	-1.49841700
C	5.14308400	-0.17546300	-0.11033400
H	4.12475000	-1.47683700	1.23519400
C	3.38628300	1.39940000	-0.25578100
H	2.11578900	0.32294000	1.10310400
H	5.32324600	-0.05172900	-1.19245500
H	3.56439300	1.46068500	-1.34072500
O	4.60060000	1.02200600	0.40425900
O	6.34205300	-0.45078000	0.54593600
O	1.15152900	0.80757800	-0.65924000
C	2.99858800	2.79170400	0.22836500
H	3.87701500	3.44383300	0.17601600
H	2.23014900	3.19341300	-0.42970300
O	2.43502900	2.80575800	1.53915400
H	3.11696300	2.58640500	2.18593400
O	1.83407900	-2.01594500	0.07210900
H	2.29710200	-2.77130600	0.48686500
O	-3.88160900	-0.93598600	0.38399000
C	7.49418600	0.23536900	0.19988100
C	7.51926400	1.38816300	-0.58405000
C	8.67925600	-0.30523900	0.70204400
C	8.74795000	1.98364000	-0.87783900
H	6.60252700	1.84217100	-0.93615500
C	9.89365400	0.30488700	0.40964700

H	8.63002200	-1.19587100	1.31869300
C	9.93566400	1.45035600	-0.38705300
H	8.76595300	2.88131600	-1.48736600
H	10.81141100	-0.11866900	0.80424000
H	10.88397000	1.92427400	-0.61549900
H	-3.26479500	-0.38232000	-1.53068700
N	4.77986900	-2.56862600	-0.41714600
H	5.62236900	-2.46170800	-0.96193500
C	5.39309300	-4.93856000	-0.39156300
H	5.95493100	-5.32015800	0.46443400
H	4.76543900	-5.75233000	-0.75735000
H	6.09713700	-4.64975900	-1.17420400
N	-0.68300200	2.64219100	0.53927100
H	0.16638100	2.50588000	1.07327300
C	-0.50328300	4.92453600	1.38355900
H	-0.30106300	5.82452300	0.80031900
H	-1.20212200	5.20483100	2.17581600
H	0.42301400	4.56706500	1.83480600
C	-1.17393200	3.90699600	0.48457800
O	-2.12454800	4.22448700	-0.23100800
C	4.50547400	-3.80893500	0.07810000
O	3.58007300	-4.00040600	0.86275600
C	-5.18616100	-1.00367500	-0.07713300
C	-6.02629700	-1.90743600	0.83280300
H	-5.21121900	-1.42642700	-1.09939700
C	-7.02174800	0.37653700	-0.69230900
C	-7.48533600	-1.84162500	0.35481100
H	-5.66761200	-2.94124600	0.72827700
C	-7.97926000	-0.39984700	0.22025300
H	-7.01942100	-0.08101500	-1.69427500
H	-7.56347300	-2.31782800	-0.63363100
H	-7.99003600	0.06712700	1.21138800
O	-5.71903400	0.30095900	-0.12029300
O	-9.31283200	-0.49991700	-0.29921600
O	-8.26320600	-2.57182500	1.29795400
O	-5.91504500	-1.47939400	2.17489000
C	-7.28325900	1.87111300	-0.83881800
H	-8.24226100	2.04717700	-1.33395000
H	-7.32006700	2.32600400	0.16326100
O	-6.29243500	2.47491400	-1.63977900
H	-5.41516600	2.21505100	-1.30154400
H	-9.79159500	0.32051900	-0.13824300
H	-9.19439300	-2.41957100	1.08920800
H	-6.60513000	-1.93855800	2.67344300

K6

C	2.61615300	0.74408300	0.29628300
C	1.20716600	1.17276100	0.77863700
C	0.15392300	0.16639400	0.30636800
C	1.69369300	-1.62034100	0.19382400
C	2.88739400	-0.74831900	0.61076500
H	1.19360200	1.16848300	1.87416400
H	2.67060400	0.87016800	-0.79267400
H	1.60282400	-1.59191200	-0.90131900
H	0.10966900	0.14096800	-0.79475200
O	0.49666500	-1.11880500	0.78282200
O	3.62549900	1.55133000	0.89531300
C	1.81089300	-3.06992300	0.62149000
H	1.85602500	-3.12916300	1.71656700
H	2.72166000	-3.49769100	0.19580600
H	3.21288600	2.26521100	1.42519000
O	0.64488700	-3.73512000	0.12833700
H	0.66120200	-4.66138600	0.39190300
C	-2.73660700	-1.21603700	0.14306800
C	-4.11850900	-1.35876400	-0.53443600
C	-2.23338900	0.22781700	0.01876900
H	-2.81729200	-1.46740900	1.20495900
C	-5.08661100	-0.19957200	-0.23766300
H	-3.94118900	-1.33110700	-1.61255900
C	-3.31434100	1.22441000	0.44353600
H	-1.98336600	0.41961400	-1.03029000
H	-5.47394000	-0.25864100	0.78406300
H	-3.61857400	1.02689000	1.48277200
O	-4.43406400	1.05467000	-0.42718000
O	-6.12309300	-0.28918100	-1.17005700
O	-1.08578200	0.50818400	0.82867800
C	-2.85493100	2.67514100	0.36501900
H	-3.72538100	3.33145900	0.46735600
H	-2.17119400	2.87630100	1.18811000
O	-2.12738100	2.98743700	-0.82613300
H	-2.72249900	2.94309200	-1.58470700
O	-1.90117500	-2.13987200	-0.53798100
H	-1.12137500	-2.37576700	-0.01012700
O	3.98698700	-1.31491800	-0.10263300
C	-7.32670100	0.33653800	-0.92128700
C	-7.51665200	1.32042300	0.04924400
C	-8.39055700	-0.06698000	-1.73137200
C	-8.78442200	1.88109400	0.21372900
H	-6.68932200	1.66708900	0.65430300
C	-9.64521200	0.50763400	-1.56337900
H	-8.21631400	-0.83066000	-2.48131800
C	-9.85163300	1.48263000	-0.58587500
H	-8.92878900	2.64452800	0.97166500
H	-10.46701700	0.18776300	-2.19608400
H	-10.83193400	1.92753100	-0.45321600
H	3.06091700	-0.84156900	1.69180000
N	-4.72573200	-2.65445300	-0.25324800
H	-4.70661700	-3.33264200	-0.99644500
C	-5.61575800	-4.49661400	1.07922800
H	-4.98224200	-5.05045900	1.77541800
H	-6.62458900	-4.48509000	1.49625200
H	-5.63480900	-5.02353500	0.12304500
N	0.83247200	2.51395300	0.31869000
H	0.02567800	2.59422800	-0.29056900
C	0.59934500	4.93694000	0.54673200
H	0.26335900	5.46977300	1.43823300
H	1.36144500	5.55766300	0.06914400
H	-0.23861500	4.80714800	-0.13885800
C	1.23718500	3.63489000	0.97360500
O	2.09817700	3.59968000	1.85495600
C	-5.10889700	-3.06917700	0.98855500
O	-5.06164600	-2.34073500	1.97222500
C	5.27223900	-0.85851500	0.16675100
C	6.29140300	-1.97375400	-0.10502100
H	5.35724000	-0.54795800	1.22056900
C	6.78231800	0.88081500	-0.37396900
C	7.70657800	-1.39109700	0.03056000
H	6.16207000	-2.75527700	0.65837900
C	7.88511800	-0.10541400	-0.77938000
H	6.85972800	1.08469300	0.70543300
H	7.89538000	-1.14674600	1.08611700
H	7.79286500	-0.33334400	-1.84723300
O	5.53060400	0.25785300	-0.66032500
O	9.21074500	0.34897600	-0.47682100
O	8.61209600	-2.40624800	-0.39147300
O	6.08590300	-2.51201800	-1.39552700
C	6.74227800	2.21458200	-1.10158600
H	7.69571200	2.74033900	-0.99079100
H	6.56896800	2.03211200	-2.17284200
O	5.74179300	3.05419700	-0.56032500
H	4.99440900	2.50727300	-0.25581800
H	9.50150200	0.98511200	-1.13937900
H	9.49293000	-2.01176500	-0.44091100
H	6.87671600	-3.02514600	-1.61362600

K7

C	2.63108200	1.00174200	0.29765500
C	1.15683200	1.41571500	0.63745500
C	0.10840200	0.39773400	0.19715300
C	1.66372600	-1.31984000	-0.23785400
C	2.89859700	-0.52456800	0.20210000
H	1.08934100	1.49305500	1.72698900
H	2.87467000	1.41565700	-0.68873100
H	1.46889000	-1.10864000	-1.29810000
H	-0.04202200	0.43974700	-0.89286700
O	0.53097800	-0.90904600	0.53973200
O	3.49267000	1.54333600	1.29142300
C	1.78614900	-2.82823100	-0.07607500
H	1.82978600	-3.07742400	0.99353800
H	2.70805900	-3.16720700	-0.54748700
H	3.21871000	2.47183000	1.44070500
O	0.73576300	-3.52678700	-0.72521300
H	-0.11720800	-3.27315100	-0.34701800
C	-2.85191700	-1.04938300	0.84992600
C	-4.15878600	-1.45223500	0.14204600
C	-2.28325900	0.20881300	0.19355600
H	-3.05759600	-0.82856200	1.90258200
C	-5.11605800	-0.25919200	-0.08219200
H	-3.88836500	-1.81610300	-0.85251700
C	-3.34870900	1.30760600	0.23771100
H	-2.05093500	0.00017800	-0.85542500
H	-5.61147000	0.02177600	0.85190000
H	-3.71696300	1.43235700	1.26747600
O	-4.41915000	0.87459200	-0.60584800
O	-6.04642100	-0.66819000	-1.03785000
O	-1.09318500	0.66148700	0.85397800
C	-2.85762400	2.66892000	-0.23266300
H	-3.72030300	3.32124000	-0.40202900
H	-2.24346800	3.10991400	0.55166700
O	-2.02300200	2.62269000	-1.39312800
H	-2.55487200	2.40472000	-2.16806200
O	-1.96710800	-2.15957600	0.75570600
H	-1.10000000	-1.86543100	1.07832100
O	3.88243000	-0.81770000	-0.80136500
C	-7.26687100	-0.02817700	-1.12529700
C	-7.53658200	1.22491900	-0.57560700
C	-8.25539000	-0.71532200	-1.83248800
C	-8.81195600	1.77339200	-0.72217200
H	-6.76412200	1.78122100	-0.06111800
C	-9.51788200	-0.15188400	-1.97990600
H	-8.01886400	-1.68607000	-2.25389500
C	-9.80579700	1.09426300	-1.42077800
H	-9.01993500	2.74737400	-0.29075200
H	-10.28191400	-0.69222600	-2.52955500
H	-10.79229800	1.53090500	-1.53309100
H	3.24040500	-0.87043800	1.18439400
N	-4.83291800	-2.55618400	0.81443300
H	-4.81263600	-3.45022400	0.35268800
C	-5.87208500	-3.79645200	2.64326000
H	-5.23630600	-4.12695700	3.46813200
H	-6.86575200	-3.60779600	3.05246600
H	-5.93657100	-4.60044800	1.90722000
N	0.78491300	2.70401100	0.04049200
H	0.00508200	2.71466100	-0.60765700
C	0.70494400	5.13134400	-0.18983700
H	0.30879200	5.76624900	0.60592800
H	1.49629300	5.69543900	-0.68734900
H	-0.08928700	4.91784900	-0.90676800
C	1.30796500	3.89165900	0.43537000
O	2.22596200	3.97313400	1.25245600
C	-5.31593900	-2.49828500	2.09086500
O	-5.30469800	-1.46767400	2.75175800
C	5.17925900	-1.06413800	-0.37196500
C	6.01809800	-1.63671600	-1.52055000
H	5.17575900	-1.80160300	0.45321000
C	7.02637400	-0.00615200	0.68456000
C	7.46946600	-1.76531900	-1.03178200
H	5.63986800	-2.63920300	-1.76695700
C	7.98659300	-0.46067800	-0.42145000
H	6.99797700	-0.76661800	1.48065600
H	7.52171300	-2.54317200	-0.25584400
H	8.02671600	0.30729500	-1.20208600
O	5.73142500	0.13906800	0.10582800
O	9.30635800	-0.75934200	0.05332000
O	8.24906800	-2.15922500	-2.15555200
O	5.93697700	-0.78769300	-2.64656200
C	7.31215500	1.35178200	1.31562500
H	8.26457600	1.33255400	1.85223100
H	7.37662500	2.10867300	0.51864500
O	6.31782300	1.68351600	2.25830400
H	5.44362400	1.58324000	1.83808800
H	9.80527600	0.05551900	0.17767800
H	9.17957900	-2.10809900	-1.89967200
H	6.63337500	-1.06550000	-3.25791600

K8			
C	2.55883700	1.29784000	-0.01541100
C	1.13091600	1.93992200	0.05244200
C	0.03296600	0.96820000	-0.38375100
C	1.32125600	-0.95712800	-0.11944400
C	2.61078700	-0.23008800	0.27955700
H	0.93490100	2.19439100	1.09917600
H	2.93990300	1.43032600	-1.03610700
H	1.29274900	-1.02040100	-1.21685700
H	0.09356000	0.74437200	-1.45905800
O	0.19502800	-0.22653300	0.34446900
O	3.40708200	1.94854300	0.92255500
C	1.17333200	-2.36945200	0.42884600
H	1.11846600	-2.34362600	1.52202600
H	2.03459900	-2.97114300	0.13325900
H	3.24638400	2.91264900	0.85107200
O	0.00012600	-2.97520800	-0.12148900
H	-0.70710300	-3.04951500	0.53876800
C	-2.48139600	-0.54547600	-0.65332600
C	-3.91955400	-1.01888600	-0.94383900
C	-2.39034200	0.98833000	-0.70225700
H	-2.18566200	-0.86937100	0.34156500
C	-4.95458700	-0.25156300	-0.11332900
H	-4.13313900	-0.78158800	-1.99065300
C	-3.53507600	1.65332000	0.07890400
H	-2.41628200	1.31486400	-1.74781400
H	-4.84990200	-0.46961800	0.95972000
H	-3.38630700	1.44664400	1.14922900
O	-4.80243700	1.13829400	-0.34173900
O	-6.22123700	-0.63527600	-0.56526600
O	-1.20780500	1.52310100	-0.07197000
C	-3.60517700	3.16389400	-0.11682400
H	-4.41965800	3.55983500	0.50235600
H	-2.67081400	3.61653100	0.21248000
O	-3.77829600	3.53371500	-1.47711400
H	-4.62076700	3.17521700	-1.78436100
O	-1.62677500	-1.11156500	-1.63513100
H	-1.05769700	-1.78447300	-1.21767700
O	3.63925400	-0.88044000	-0.48339200
C	-7.33115500	-0.41354400	0.22712200
C	-7.35763800	0.46180800	1.31264300
C	-8.47667400	-1.12685900	-0.13145800
C	-8.53651900	0.59963100	2.04787200
H	-6.48805300	1.05250400	1.56874700
C	-9.64651500	-0.97146800	0.60348500
H	-8.43354800	-1.79496200	-0.98468600
C	-9.68205400	-0.11088100	1.70182500
H	-8.55408900	1.28110000	2.89235700
H	-10.53301400	-1.52900300	0.31888700
H	-10.59440300	0.00754900	2.27616700
H	2.80017900	-0.35266900	1.35226600
N	-4.09691700	-2.46638100	-0.81527400
H	-4.79381500	-2.85656300	-1.42886600
C	-3.91615100	-4.78357400	-0.10189000
H	-3.04665300	-5.36016400	-0.42641900
H	-4.21494700	-5.16579100	0.87554200
H	-4.72853200	-4.94742000	-0.81244600
N	1.00503200	3.15009000	-0.77274200
H	0.19874300	3.18371500	-1.37927200
C	1.18171300	5.51611400	-1.34979200
H	0.79878700	6.28612100	-0.67639800
H	2.04866800	5.93662800	-1.86252300
H	0.41291600	5.27751100	-2.08679700
C	1.63145100	4.32976400	-0.52351000
O	2.52454500	4.44602300	0.31459400
C	-3.50275700	-3.33125900	0.04839600
O	-2.66975500	-3.00253100	0.89337200
C	4.84036100	-1.14972500	0.15508100
C	5.67155300	-2.13759900	-0.67227400
H	4.65350500	-1.60452900	1.14653600
C	6.73754600	-0.07343000	1.09765800
C	7.03869100	-2.29974800	0.01026100
H	5.16085200	-3.11138400	-0.67202200
C	7.70345100	-0.95078400	0.29140100
H	6.52126000	-0.55843400	2.06262700
H	6.90317700	-2.81403400	0.97309900
H	7.92939300	-0.45758300	-0.66062700
O	5.53609000	0.06034700	0.34191400
O	8.91573200	-1.26262700	0.99160100
O	7.83711900	-3.10452600	-0.85111400
O	5.81910500	-1.65833000	-1.99268000
C	7.17876600	1.36116100	1.36193900
H	8.07036200	1.37876000	1.99475000
H	7.42344100	1.84157200	0.40192000
O	6.17430800	2.07181300	2.05007400
H	5.32738700	1.94782100	1.58247500
H	9.52347100	-0.51621900	0.94813800
H	8.74029200	-3.09463900	-0.50740600
H	6.51173800	-2.19047900	-2.40848900

L1

C	2.64142300	-1.00074900	-0.30643400
C	1.19846400	-1.53154300	-0.50357200
C	0.12280900	-0.50835800	-0.11396200
C	1.60312600	1.31616100	-0.30718300
C	2.80074500	0.47165300	-0.75343600
H	1.06781800	-1.70436300	-1.57509400
H	2.89384100	-1.05148200	0.75676700
H	1.57392100	1.34728100	0.79165300
H	0.09561100	-0.32752100	0.96307900
O	0.41903300	0.69440600	-0.79904900
O	3.45887300	-1.88318400	-1.06372600
C	1.55707100	2.74429000	-0.82853200
H	1.49369500	2.71811200	-1.92713600
H	2.46583700	3.28076300	-0.55049800
H	4.34340400	-1.95610900	-0.67209500
O	0.46284900	3.44956800	-0.27344900
H	-0.28962400	2.84098100	-0.16450300
C	-2.67272400	0.88596900	-0.00433700
C	-4.12113800	1.13028500	0.47259000
C	-2.28315000	-0.59802000	0.16506200
H	-2.60440200	1.13227700	-1.07238700
C	-5.06933100	0.11599500	-0.17434600
H	-4.17105800	0.97322100	1.55524500
C	-3.36900300	-1.53342800	-0.38926000
H	-2.12754200	-0.80523900	1.22933800
H	-5.08445100	0.23769700	-1.27134500
H	-3.38743400	-1.43304500	-1.48539300
O	-4.65664200	-1.19273700	0.14383000
O	-6.34751700	0.32940600	0.34582800
O	-1.11653400	-0.96324300	-0.57302100
C	-3.14431800	-3.00188100	-0.04646800
H	-3.92096700	-3.59942000	-0.54124800
H	-2.17196900	-3.31140900	-0.42656700
O	-3.12994300	-3.24904800	1.35026400
H	-3.98774200	-2.99772800	1.71585800
O	-1.75550400	1.71747900	0.69661500
H	-2.22752200	2.28255600	1.33879400
O	3.95850200	1.10870500	-0.17391600
C	-7.45243100	-0.18561000	-0.30833800
C	-7.39348100	-1.15253200	-1.31169300
C	-8.68261700	0.32738100	0.10782600
C	-8.57902300	-1.58464000	-1.91011900
H	-6.44944500	-1.59238400	-1.60451300
C	-9.85493900	-0.12017300	-0.49044200
H	-8.70108800	1.06933500	0.89848300
C	-9.80976300	-1.07540000	-1.50744500
H	-8.53124300	-2.33784600	-2.68997100
H	-10.80788600	0.28168200	-0.16191000
H	-10.72510000	-1.42240000	-1.97430200
H	2.88948400	0.50101800	-1.84676200
N	-4.58681500	2.48932000	0.18793100
H	-5.34874000	2.58928700	-0.46563400
C	-5.10232000	4.80813200	0.78016900
H	-5.74731200	5.00826200	1.63904500
H	-4.39169800	5.63279800	0.70739900
H	-5.71469700	4.78370200	-0.12317800
N	1.01212200	-2.82586200	0.14454400
H	1.06629400	-3.63772100	-0.44781300
C	0.79866000	-4.45455000	1.94858100
H	1.43263500	-4.64444600	2.81499400
H	-0.24165000	-4.57603800	2.26287100
H	1.01347200	-5.19507200	1.17433700
C	0.99730300	-3.01935900	1.49624600
O	1.09074400	-2.09856900	2.29754500
C	-4.33946300	3.52805700	1.03496200
O	-3.53056200	3.43229000	1.95464600
C	5.21728300	0.80622900	-0.67758000
C	6.13971100	1.98766000	-0.33849600
H	5.17964500	0.66085200	-1.76976800
C	6.98120300	-0.78776600	-0.47523700
C	7.59925200	1.63020000	-0.62343900
H	5.84343900	2.84496600	-0.95822600
C	7.98094900	0.27444900	-0.01595600
H	7.01622500	-0.87412100	-1.57185800
H	7.75103500	1.56803600	-1.70624400
H	7.93588500	0.34941300	1.07846600
O	5.67024700	-0.39237500	-0.06493300
O	9.28305100	-0.10986500	-0.43513500
O	8.48773400	2.64498700	-0.16949500
O	6.07016900	2.33626900	1.03774200
C	7.22837400	-2.15768500	0.12006800
H	8.23689900	-2.48829700	-0.14428300
H	7.14081200	-2.10419400	1.21199100
O	6.23399500	-3.02853300	-0.42696700
H	6.29929500	-3.89838000	-0.01846000
H	9.86565400	0.65116800	-0.30537800
H	8.16204600	2.95694900	0.68759500
H	5.14348100	2.34958400	1.31171000

L2

C	2.70082100	-0.77629400	-0.56334700
C	1.25707100	-1.22802600	-0.89305500
C	0.18534400	-0.30893900	-0.29304700
C	1.68694100	1.51801500	-0.12071700
C	2.87854400	0.75077600	-0.70984800
H	1.13909100	-1.16317000	-1.97777900
H	2.92865700	-1.04568000	0.46978900
H	1.67020100	1.36881600	0.96868400
H	0.16847800	-0.36583700	0.79942300
O	0.48126500	1.02981700	-0.69214000
O	3.55137100	-1.46914600	-1.46773500
C	1.71552600	3.00880800	-0.40147700
H	1.62760100	3.17595100	-1.48209300
H	2.65738700	3.43744400	-0.04722600
H	4.29333300	-1.87515600	-0.99239000
O	0.60230600	3.57699000	0.29235400
H	0.48401900	4.49574700	0.02850500
C	-2.64092900	1.01378900	0.09190700
C	-4.04720800	1.12852200	0.71758300
C	-2.20568700	-0.45852600	-0.00193300
H	-2.65750600	1.43270800	-0.91881700
C	-5.05621200	0.13665100	0.12050900
H	-3.95092800	0.86203500	1.77344100
C	-3.31213200	-1.31123700	-0.63619800
H	-1.99299200	-0.83775900	1.00325000
H	-5.32878800	0.41289100	-0.90279300
H	-3.48837800	-0.96465300	-1.66601500
O	-4.50896300	-1.17626300	0.13644500
O	-6.18074000	0.14254300	0.95192100
O	-1.05079500	-0.64719300	-0.83038400
C	-3.00276500	-2.80273700	-0.68103600
H	-3.83211100	-3.31476500	-1.18558100
H	-2.09259700	-2.96756700	-1.25582500
O	-2.77690400	-3.36136100	0.60578900
H	-3.56066200	-3.19779300	1.14654000
O	-1.79326800	1.77602100	0.93925600
H	-1.04347700	2.14070600	0.44392500
O	4.03173800	1.26609400	-0.01249200
C	-7.38934500	-0.31548200	0.47443500
C	-7.53749100	-1.10393700	-0.66718100
C	-8.50836700	0.04723200	1.22803500
C	-8.81604800	-1.50575400	-1.05779600
H	-6.67278300	-1.42449700	-1.23304300
C	-9.77443500	-0.36913700	0.83294800
H	-8.36631000	0.65658300	2.11376200
C	-9.93727100	-1.14482600	-0.31614900
H	-8.92727800	-2.11703100	-1.94785400
H	-10.63869900	-0.08143200	1.42323500
H	-10.92594800	-1.46654500	-0.62544700
H	2.97982300	0.98371000	-1.77800200
N	-4.54352700	2.50008300	0.68638200
H	-4.51096800	3.00789200	1.55444500
C	-5.24241000	4.64124800	-0.25203500
H	-4.60803100	5.27703600	-0.87250200
H	-6.26912500	4.76413700	-0.60397600
H	-5.18408300	4.98268200	0.78361700
N	1.04033600	-2.62516000	-0.53623000
H	0.88175600	-3.27244600	-1.29012700
C	0.63574300	-4.56506300	0.88153300
H	1.13079700	-4.97999100	1.75905500
H	-0.44563800	-4.62396700	1.03691800
H	0.89052000	-5.16460800	0.00363100
C	1.01367400	-3.10339700	0.74301500
O	1.23751900	-2.39206500	1.71489500
C	-4.82855100	3.19390900	-0.45152000
O	-4.77877800	2.69067200	-1.56736100
C	5.30115500	0.87614700	-0.43297900
C	6.29564800	1.94348000	0.04965800
H	5.34140800	0.78922100	-1.53024700
C	6.87670300	-0.88616000	-0.21793000
C	7.73492500	1.45731200	-0.14585400
H	6.13625800	2.86050700	-0.53415000
C	7.94163400	0.03052600	0.38388600
H	6.97653300	-0.88998900	-1.31427000
H	7.97005300	1.45254000	-1.21547100
H	7.82694600	0.03406000	1.47585000
O	5.59282900	-0.38125500	0.14816600
O	9.22672700	-0.45289400	0.01987700
O	8.67390800	2.34714900	0.44697700
O	6.14420800	2.22090500	1.43638500
C	6.93114000	-2.31950100	0.26415100
H	7.91981900	-2.73720900	0.05541700
H	6.74170800	-2.35398400	1.34317900
O	5.91064000	-3.02769700	-0.45192200
H	5.82187600	-3.92035700	-0.10115700
H	9.87138700	0.23110400	0.24760800
H	8.31730900	2.62094800	1.30467800
H	5.20306100	2.23031400	1.65517600

L3

C	2.67197400	0.74407000	0.55357800
C	1.21961500	1.10063700	0.96441400
C	0.16378700	0.44853900	0.06475800
C	1.67174800	-1.26872300	-0.65290700
C	2.83824900	-0.75111800	0.19596100
H	1.05816800	0.67941300	1.95990700
H	2.94705700	1.32934900	-0.32757700
H	1.68266700	-0.77462100	-1.63359100
H	0.17680600	0.84656100	-0.95556200
O	0.45277800	-0.95295200	0.02169700
O	3.46384100	1.10729500	1.67479300
C	1.66233600	-2.77893500	-0.86268500
H	1.60833800	-3.27905500	0.11543700
H	2.58650200	-3.08725700	-1.35027800
H	4.33751400	1.42479000	1.39610700
O	0.60523200	-3.19433100	-1.70867900
H	-0.24325900	-2.97237200	-1.29985500
C	-2.80664900	-1.09952500	0.54494600
C	-4.18408900	-1.46094100	-0.03212400
C	-2.22708000	0.15946400	-0.11208700
H	-2.91323500	-0.89571200	1.61612300
C	-5.11155800	-0.23581100	-0.06864800
H	-4.04537000	-1.77115900	-1.07114900
C	-3.25768200	1.29518200	-0.12491400
H	-1.92188100	-0.05872700	-1.13954900
H	-5.39327600	0.07785400	0.94136500
H	-3.45970200	1.60999600	0.90981600
O	-4.46240600	0.83358600	-0.74656700
O	-6.23902800	-0.59109700	-0.81223800
O	-1.08700900	0.60152300	0.65523400
C	-2.81727000	2.50685000	-0.94045800
H	-3.57060100	3.29732900	-0.82897500
H	-1.86593100	2.88325800	-0.56435800
O	-2.61632100	2.18785000	-2.30723000
H	-3.44847000	1.84404600	-2.65785200
O	-1.97292000	-2.23936500	0.35493300
H	-1.07239200	-1.98192500	0.60784300
O	4.01477200	-1.01645400	-0.59197300
C	-7.39920600	0.14472900	-0.69028400
C	-7.46852600	1.41539900	-0.11862100
C	-8.54957100	-0.46033000	-1.20151300
C	-8.70253900	2.06377000	-0.04420100
H	-6.57601100	1.90891900	0.24249000
C	-9.76987700	0.20169100	-1.12980900
H	-8.46862200	-1.44652300	-1.64513600
C	-9.85527400	1.46692400	-0.54617700
H	-8.75262200	3.05146400	0.40311900
H	-10.65958900	-0.27593400	-1.52769000
H	-10.80857600	1.98115800	-0.48839300
H	2.89880300	-1.32477400	1.13007200
N	-4.78757200	-2.59446500	0.65927100
H	-4.79693900	-3.47058400	0.16424400
C	-5.62706200	-3.92737600	2.52324800
H	-4.91912700	-4.28033400	3.27689500
H	-6.58428100	-3.76997100	3.02292700
H	-5.74591200	-4.70277100	1.76358300
N	1.04660000	2.54227400	1.10118000
H	1.02150700	2.90281500	2.04062400
C	1.11102600	4.89827400	0.46031700
H	2.08680200	5.35561900	0.27895100
H	0.39315000	5.40039200	-0.19013600
H	0.83113400	5.07133200	1.50166400
C	1.18017400	3.43141000	0.07434500
O	1.34018500	3.07885900	-1.08644200
C	-5.13599500	-2.59892100	1.97848100
O	-5.06567000	-1.60108800	2.68479100
C	5.26075100	-0.98352000	0.02767900
C	6.21670100	-1.84287400	-0.81384900
H	5.19888800	-1.38584700	1.05211600
C	6.98322900	0.52172500	0.68644300
C	7.65799800	-1.66010200	-0.33413700
H	5.92436300	-2.89618100	-0.70442100
C	8.02696900	-0.17766900	-0.18515300
H	6.97919700	0.06567700	1.68801800
H	7.77400800	-2.13455600	0.64607900
H	8.02941500	0.29105500	-1.17802000
O	5.69737200	0.36367200	0.08097500
O	9.29912900	-0.04154800	0.43206100
O	8.58448500	-2.31832800	-1.18941500
O	6.19816900	-1.47200300	-2.18559600
C	7.20832500	2.01040500	0.84176700
H	8.19616500	2.18192100	1.27838300
H	7.15804200	2.49529900	-0.14049900
O	6.16877000	2.49338800	1.69914500
H	6.23745500	3.44965100	1.79203400
H	9.91073900	-0.63428000	-0.02615600
H	8.29942300	-2.16833000	-2.10271000
H	5.28238400	-1.36305700	-2.47316800

L4

C	-2.59378200	1.04548900	-0.39207900
C	-1.20088200	1.71021000	-0.55575800
C	-0.07368900	0.88638100	0.07758800
C	-1.32301900	-1.11900200	0.01805900
C	-2.55953100	-0.48496500	-0.62789300
H	-0.98507000	1.73979500	-1.62671500
H	-2.94664300	1.21729900	0.62750000
H	-1.39889500	-1.00946700	1.11027900
H	-0.14792900	0.84681500	1.16704000
O	-0.17769300	-0.43151000	-0.45584700
O	-3.43681800	1.69334900	-1.33596700
C	-1.09390100	-2.59286700	-0.29429500
H	-0.95027900	-2.72552500	-1.37193600
H	-1.96035000	-3.17991500	0.01553300
H	-4.31315800	1.86046200	-0.95523900
O	0.04450100	-3.06104300	0.43055700
H	0.80435300	-3.21111000	-0.15461100
C	2.49338300	-0.46709800	0.61834700
C	3.94208300	-0.82200000	1.00116900
C	2.32540400	1.05296200	0.42846300
H	2.23650700	-0.95104100	-0.32084100
C	4.95751400	-0.14048700	0.07877100
H	4.12074100	-0.41821600	2.00272100
C	3.46802100	1.65733100	-0.40425700
H	2.28143900	1.53556600	1.41089100
H	4.87793900	-0.51645100	-0.95235000
H	3.35594000	1.30728300	-1.44134700
O	4.74737200	1.25767000	0.10205200
O	6.23306700	-0.40176000	0.59378900
O	1.15276800	1.40554500	-0.32722400
C	3.48241300	3.18254000	-0.40553400
H	4.26214200	3.52459600	-1.09866200
H	2.52013900	3.55418700	-0.75526800
O	3.68415300	3.72900200	0.88809900
H	4.52870900	3.40270200	1.22465500
O	1.65053500	-0.92841700	1.66280400
H	1.06938400	-1.63266000	1.32543400
O	-3.68859100	-1.17492000	-0.05244000
C	7.34800000	-0.21709700	-0.19865900
C	7.34528500	0.46832100	-1.41376800
C	8.53325600	-0.76423200	0.29791300
C	8.53550100	0.58223100	-2.13467400
H	6.44264700	0.93476500	-1.78536800
C	9.71277700	-0.63426000	-0.42621300
H	8.51240100	-1.28506200	1.24898900
C	9.72034600	0.03588800	-1.65064800
H	8.52977300	1.11661600	-3.07933900
H	10.62948400	-1.06198300	-0.03313600
H	10.64070500	0.13520400	-2.21584000
H	-2.54848400	-0.66724700	-1.71028100
N	4.19160400	-2.26225500	1.09493100
H	4.91868000	-2.51272300	1.74550800
C	4.11131700	-4.67171400	0.79482400
H	3.27966400	-5.22114700	1.24241800
H	4.40482500	-5.20469600	-0.11099300
H	4.94784500	-4.67291000	1.49636800
N	-1.21921100	3.09704200	-0.10400000
H	-1.16746600	3.80744300	-0.81447500
C	-1.37452500	4.99036300	1.42712500
H	-2.21022900	5.26102900	2.07394600
H	-0.45352400	5.24232700	1.95882900
H	-1.42003900	5.58620500	0.51273000
C	-1.40234500	3.49081700	1.18869500
O	-1.55503300	2.69740500	2.10860900
C	3.62186500	-3.28711300	0.40962900
O	2.75778200	-3.15138700	-0.45714100
C	-4.94563600	-0.99816200	-0.62139400
C	-5.79845600	-2.21840400	-0.23966300
H	-4.87278900	-0.91749300	-1.71804600
C	-6.81310300	0.47671600	-0.60243400
C	-7.26559700	-1.98007200	-0.60460300
H	-5.42050600	-3.09213100	-0.78801400
C	-7.76526600	-0.61450700	-0.11281000
H	-6.79694000	0.48039700	-1.70314800
H	-7.37413100	-2.00310300	-1.69409700
H	-7.77079500	-0.61016200	0.98508500
O	-5.50566300	0.19559800	-0.10127400
O	-9.06657300	-0.35018000	-0.61813600
O	-8.10442000	-3.02035100	-0.11521400
O	-5.76434800	-2.47198100	1.15898400
C	-7.16701800	1.87009300	-0.12921900
H	-8.18076600	2.11770000	-0.45614300
H	-7.11879600	1.91099600	0.96530900
O	-6.20370100	2.75521900	-0.71263900
H	-6.32685900	3.64690900	-0.36995300
H	-9.60550100	-1.13762700	-0.46053100
H	-7.80338300	-3.24375400	0.77768200
H	-4.85870800	-2.35945300	1.47668600

L5

C	2.62968400	-0.98829100	-0.18303100
C	1.19425400	-1.49610100	-0.46113000
C	0.11921800	-0.46570500	-0.08636500
C	1.63591100	1.33754600	-0.18886600
C	2.82861700	0.48000100	-0.62375600
H	1.10844100	-1.63401600	-1.54251800
H	2.82348000	-1.03985600	0.89290600
H	1.57641700	1.34333800	0.90857700
H	0.05743000	-0.31201300	0.99331300
O	0.45375900	0.74800400	-0.73044300
O	3.47811000	-1.88482100	-0.89101700
C	1.63283600	2.77769500	-0.67580900
H	1.63351100	2.78181300	-1.77693200
H	2.52809000	3.29328800	-0.32613500
H	4.35232200	-1.92329500	-0.47405100
O	0.51127500	3.47969800	-0.16965200
H	-0.24642200	2.87203200	-0.11096300
C	-2.69392800	0.92310800	-0.01360900
C	-4.14753900	1.14498600	0.45824700
C	-2.29192000	-0.55991300	0.13000800
H	-2.62159400	1.18885000	-1.07708000
C	-5.08418300	0.13230300	-0.20806900
H	-4.20131200	0.97245800	1.53827000
C	-3.36262900	-1.48965600	-0.46063700
H	-2.15128100	-0.78931000	1.19180900
H	-5.10436800	0.27829600	-1.30217100
H	-3.37837100	-1.35357300	-1.55306900
O	-4.65598700	-1.17877500	0.07832500
O	-6.36376100	0.31832100	0.32028300
O	-1.11084200	-0.89436000	-0.59705800
C	-3.12463100	-2.96665500	-0.16818300
H	-3.89516200	-3.55374400	-0.68484500
H	-2.14887900	-3.25274900	-0.55768000
O	-3.10966400	-3.26385000	1.21893300
H	-3.96533000	-3.02035900	1.59472900
O	-1.78787000	1.74821500	0.70685900
H	-2.26639000	2.30147500	1.35352100
O	3.98515000	1.09121000	-0.02937000
C	-7.46382200	-0.19729300	-0.34018000
C	-7.39664400	-1.14392400	-1.36231300
C	-8.69908900	0.29188900	0.08985900
C	-8.57885800	-1.57974000	-1.96457800
H	-6.44807700	-1.56585100	-1.66650900
C	-9.86787800	-0.15916100	-0.51260500

H	-8.72380300	1.01831700	0.89463300
C	-9.81451900	-1.09425600	-1.54779000
H	-8.52441800	-2.31725800	-2.75889300
H	-10.82460800	0.22411200	-0.17295700
H	-10.72717300	-1.44418000	-2.01776200
H	2.92446300	0.51032500	-1.71759800
N	-4.62482000	2.50338900	0.19121800
H	-5.37150400	2.60992100	-0.47853800
C	-5.16203000	4.81117700	0.80610100
H	-5.81566500	4.99991000	1.66095500
H	-4.45439100	5.63961800	0.74798200
H	-5.76603800	4.79319400	-0.10308400
N	0.95739600	-2.80721300	0.13556600
H	1.04842200	-3.60074000	-0.47679300
C	0.58731200	-4.49102900	1.85957600
H	1.11593100	-4.71305300	2.78669900
H	-0.48390400	-4.60842400	2.04689300
H	0.88421300	-5.21040200	1.09216700
C	0.84612300	-3.04514500	1.47576400
O	0.89650400	-2.15530200	2.31444800
C	-4.39538500	3.53193300	1.05538800
O	-3.59947600	3.42859000	1.98506800
C	5.22635000	0.79970200	-0.55274400
C	6.18778100	1.96863000	-0.30996400
H	5.16030500	0.61822800	-1.63928100
C	6.97924300	-0.82773400	-0.43478800
C	7.58412300	1.56712700	-0.79697600
H	5.83848500	2.82390000	-0.90497600
C	8.03474900	0.25539200	-0.15729100
H	6.91567300	-0.99506800	-1.52132600
H	7.56251000	1.42377200	-1.88766600
H	8.13039100	0.40443400	0.92324500
O	5.71917600	-0.39316400	0.06734600
O	9.31087200	-0.04841800	-0.73862700
O	8.46536200	2.63441000	-0.47200800
O	6.20648300	2.29378200	1.06479100
C	7.26599300	-2.15686000	0.23627700
H	8.25992300	-2.51583500	-0.05863200
H	7.23656400	-2.02686500	1.32506600
O	6.25753400	-3.06748400	-0.19652200
H	6.26561100	-3.85777600	0.35422600
H	9.84492100	-0.55825300	-0.12056100
H	9.36479300	2.34881500	-0.67922100
H	6.89092100	2.96472100	1.19070900

L6

C	2.70130600	-0.74063500	-0.48577300
C	1.26712200	-1.18904700	-0.85808400
C	0.18368800	-0.29748000	-0.23531800
C	1.68585300	1.52118500	0.00456200
C	2.86822300	0.78500700	-0.63953300
H	1.16608600	-1.07940800	-1.94114300
H	2.90050200	-1.00331000	0.55466600
H	1.68608000	1.31523900	1.08429600
H	0.15485000	-0.40148900	0.85419300
O	0.47147100	1.05635700	-0.57507500
O	3.58904800	-1.43365500	-1.35718800
C	1.71246500	3.02388600	-0.19216800
H	1.64544400	3.25619600	-1.26276100
H	2.64470500	3.42768000	0.21029200
H	4.25167800	-1.91152200	-0.83542400
O	0.57867900	3.54551500	0.50864800
H	0.50684800	4.49357700	0.35567000
C	-2.64431300	1.01308500	0.15633900
C	-4.06407200	1.11095500	0.75517800
C	-2.21245600	-0.45737100	0.01615300
H	-2.63622900	1.47019600	-0.83793700
C	-5.06403200	0.14610300	0.10248100
H	-3.99095700	0.80578400	1.80240300
C	-3.31088300	-1.28248800	-0.66690800
H	-2.01821300	-0.87207000	1.01123700
H	-5.31246200	0.45765700	-0.91678800
H	-3.46604300	-0.90000500	-1.68736800
O	-4.52207700	-1.16904100	0.08658700
O	-6.20717100	0.12871400	0.90857700
O	-1.04554600	-0.62380100	-0.79931900
C	-3.00798300	-2.77319200	-0.75705700
H	-3.83112500	-3.26363100	-1.29233900
H	-2.08919700	-2.92375000	-1.32194100
O	-2.80712100	-3.37601200	0.51404800
H	-3.59720700	-3.22067200	1.04802600
O	-1.81536600	1.73870400	1.05151600
H	-1.05310600	2.12186500	0.58906600
O	4.03071400	1.32426300	0.00760600
C	-7.40354900	-0.31821200	0.39264000
C	-7.52675300	-1.06209600	-0.78149500
C	-8.53812900	0.00785400	1.13992900
C	-8.79542100	-1.45706100	-1.20937900
H	-6.65017700	-1.35377700	-1.34466600
C	-9.79416400	-0.40124700	0.70714200
H	-8.41544900	0.58376700	2.05056300
C	-9.93174500	-1.13291200	-0.47377400
H	-8.88704300	-2.03387100	-2.12435700
H	-10.67049400	-0.14234600	1.29299000
H	-10.91264100	-1.44900900	-0.81235200
H	2.91244200	1.02102500	-1.71223300
N	-4.55292000	2.48565600	0.76458000
H	-4.51134800	2.96739200	1.64710300
C	-5.19089900	4.67188400	-0.11150600
H	-4.46826700	5.31285200	-0.62127100
H	-6.16714100	4.85954800	-0.56278300
H	-5.22997700	4.95257600	0.94309700
N	1.04629600	-2.60136300	-0.56708700
H	0.82795000	-3.19859700	-1.34696700
C	0.59388300	-4.60136300	0.74592100
H	1.06611600	-5.07622600	1.60544000
H	-0.49058500	-4.63437700	0.88804400
H	0.83938300	-5.16300400	-0.15939100
C	1.01939200	-3.14669300	0.68470000
O	1.28134700	-2.50035900	1.69170500
C	-4.81220700	3.22127000	-0.35308700
O	-4.76085000	2.75380900	-1.48427900
C	5.27530300	0.87793400	-0.39797100
C	6.34227200	1.94194600	-0.11519700
H	5.27619400	0.65006600	-1.47574600
C	6.78570800	-0.93185200	-0.10473200
C	7.72126200	1.34963600	-0.43975000
H	6.16356300	2.79628300	-0.78376700
C	7.94719900	0.00654300	0.25788900
H	6.78053900	-1.08372900	-1.19607700
H	7.79430700	1.17873100	-1.52400800
H	7.96510600	0.16393700	1.34155400
O	5.57404000	-0.32001900	0.31283200
O	9.22255700	-0.45950100	-0.20440500
O	8.69026000	2.31197500	-0.04189000
O	6.27205500	2.34877700	1.23625900
C	6.81622700	-2.29605500	0.55451400
H	7.77593300	-2.79111100	0.36486500
H	6.67109200	-2.18366400	1.63462600
O	5.75120700	-3.05801500	-0.02472200
H	5.50302100	-3.78021100	0.56226600
H	9.62436900	-1.03713900	0.45308300
H	9.56435600	1.91476000	-0.15025700
H	7.05773100	2.88455400	1.41214400

L7

C	2.65844400	0.79718200	0.45560500
C	1.21229700	1.12299300	0.90612800
C	0.15172800	0.47839100	0.00557300
C	1.67682200	-1.20298300	-0.76361800
C	2.83654400	-0.69552900	0.10078900
H	1.07445100	0.67169700	1.89225800
H	2.89883400	1.38363600	-0.43525000
H	1.68251500	-0.68191400	-1.72957700
H	0.14562800	0.90335500	-1.00391100
O	0.45270800	-0.91747100	-0.07760700
O	3.47806600	1.18223900	1.55158700
C	1.69043900	-2.70564700	-1.01673400
H	1.66845800	-3.23629900	-0.05315000
H	2.60926000	-2.97440100	-1.53573600
H	4.34071800	1.49431100	1.23795500
O	0.62063500	-3.11877000	-1.84962300
H	-0.22111000	-2.91194100	-1.42036000
C	-2.79543600	-1.10928800	0.51071200
C	-4.17801500	-1.47601700	-0.05032500
C	-2.23883300	0.16435100	-0.13758400
H	-2.88774900	-0.92067900	1.58619000
C	-5.11844700	-0.26054800	-0.04869400
H	-4.05306100	-1.76576600	-1.09694700
C	-3.28043000	1.29000100	-0.11727800
H	-1.94745300	-0.03693700	-1.17250100
H	-5.38397900	0.03373600	0.97149600
H	-3.46557100	1.58956300	0.92510700
O	-4.49330600	0.82585500	-0.72171200
O	-6.25729000	-0.61454100	-0.77611300
O	-1.09228300	0.60540100	0.61899000
C	-2.86770000	2.51635200	-0.92549400
H	-3.62340600	3.30000000	-0.78430100
H	-1.90984700	2.89383800	-0.56754300
O	-2.69855700	2.21850400	-2.30112900
H	-3.53397800	1.86435100	-2.63325700
O	-1.95409100	-2.23744400	0.29220400
H	-1.04774100	-1.96947600	0.51307400
O	4.01514400	-0.96098900	-0.67205700
C	-7.41962500	0.11108300	-0.62295900
C	-7.48889000	1.36949000	-0.02453300
C	-8.57376100	-0.49097000	-1.12954500
C	-8.72577000	2.00791400	0.08113600
H	-6.59438200	1.86160500	0.33341400
C	-9.79709400	0.16124600	-1.02646300
H	-8.49299400	-1.46729800	-1.59451400
C	-9.88204400	1.41374300	-0.41588200
H	-8.77549000	2.98597800	0.54923500
H	-10.68948200	-0.31415500	-1.42106000
H	-10.83767200	1.92032800	-0.33362900
H	2.87770000	-1.27105600	1.03618800
N	-4.75693400	-2.62927200	0.62988100
H	-4.74869000	-3.49936400	0.12421300
C	-5.54157100	-4.00810300	2.48391900
H	-4.80813700	-4.36442600	3.21125700
H	-6.48761500	-3.87648500	3.01140300
H	-5.66771500	-4.76999400	1.71184800
N	1.01738900	2.55712800	1.08906000
H	1.00767600	2.88775300	2.03967700
C	1.03254300	4.93349300	0.52795100
H	1.99782100	5.41235900	0.34568500
H	0.29562900	5.44608400	-0.09233500
H	0.76859800	5.06681200	1.57931400
C	1.11695700	3.48131500	0.09030600
O	1.25813700	3.17237700	-1.08494400
C	-5.08611900	-2.66105300	1.95348200
O	-5.02688700	-1.67325300	2.67483600
C	5.24133300	-0.94152400	-0.03645500
C	6.22564500	-1.86038800	-0.76900000
H	5.14585800	-1.28638300	1.00715900
C	6.96182200	0.55890400	0.66854100
C	7.60748100	-1.71782500	-0.12052800
H	5.88341800	-2.89715000	-0.64234000
C	8.04510700	-0.25506400	-0.05745600
H	6.86830300	0.18894300	1.70158400
H	7.56719000	-2.10358600	0.90906600
H	8.16472200	0.12343300	-1.07788600
O	5.72487800	0.40220100	-0.02023100
O	9.30251700	-0.25049400	0.63234200
O	8.51155800	-2.49917200	-0.88998900
O	6.27044200	-1.51556400	-2.13797600
C	7.22908400	2.04997400	0.72035200
H	8.20221800	2.23809500	1.18996200
H	7.23309700	2.45557400	-0.29866400
O	6.18049200	2.63050400	1.49553600
H	6.20086400	3.59020800	1.41451900
H	9.84238500	0.49002200	0.33662900
H	9.40277800	-2.34142000	-0.55184100
H	6.98240000	-2.03196700	-2.53957200

L8

C	2.59708200	1.00930300	0.34567900
C	1.21536700	1.67771100	0.57353400
C	0.07454900	0.88563500	-0.07747700
C	1.31343800	-1.12607800	-0.09314900
C	2.55090700	-0.52010800	0.57708000
H	1.02175000	1.65698600	1.64916400
H	2.90993400	1.18412300	-0.68594600
H	1.39219200	-0.97464100	-1.17960300
H	0.14148100	0.88639400	-1.16871200
O	0.16918100	-0.44985600	0.40595600
O	3.49465600	1.63704400	1.25621900
C	1.08309400	-2.61060800	0.15736300
H	0.97464000	-2.79785100	1.23121200
H	1.93413700	-3.18188100	-0.21471100
H	4.31293900	1.88343000	0.79955700
O	-0.08616000	-3.03775100	-0.54887800
H	-0.83091200	-3.18328300	0.05524200
C	-2.49572600	-0.43313700	-0.65554800
C	-3.94810700	-0.77178700	-1.04240700
C	-2.32699000	1.07710200	-0.40107200
H	-2.23346200	-0.95606900	0.26086000
C	-4.95520600	-0.13914500	-0.07754600
H	-4.13741400	-0.31965600	-2.02088400
C	-3.46041100	1.63743900	0.47418200
H	-2.29841800	1.60325800	-1.36163700
H	-4.86506000	-0.56571000	0.93250000
H	-3.33339900	1.23819400	1.49156800
O	-4.74588000	1.25946700	-0.03355100
O	-6.23657800	-0.37365200	-0.59119200
O	-1.14550800	1.40188300	0.35344500
C	-3.47924600	3.16072600	0.54880200
H	-4.24804900	3.46649600	1.27062000
H	-2.51214100	3.51907000	0.89911200
O	-3.70578500	3.76725900	-0.71379500
H	-4.54942400	3.44335600	-1.05512700
O	-1.66147100	-0.85239400	-1.72359300
H	-1.07990600	-1.57133100	-1.41776000
O	3.66773700	-1.22880100	0.01933200
C	-7.34144500	-0.23202900	0.22263600
C	-7.32523300	0.39076900	1.47107600
C	-8.53233900	-0.75508800	-0.28660800
C	-8.50678200	0.46623400	2.21095700
H	-6.41853200	0.83867200	1.85532400
C	-9.70324400	-0.66359300	0.45709700
H	-8.52215900	-1.22717400	-1.26294900
C	-9.69698800	-0.05638900	1.71398900
H	-8.49001000	0.95155300	3.18164800
H	-10.62416400	-1.07236100	0.05371700
H	-10.61064700	0.01261500	2.29441700
H	2.51218700	-0.70305900	1.66009900
N	-4.18817200	-2.20634400	-1.20838600
H	-4.86808800	-2.43719000	-1.91442800
C	-4.07524800	-4.62602300	-1.01334400
H	-3.21578300	-5.12821400	-1.46419100
H	-4.38094400	-5.21470900	-0.14709000
H	-4.89000200	-4.61017400	-1.74005000
N	1.22761600	3.08563600	0.19055000
H	1.12952400	3.75839200	0.93201400
C	1.33161700	5.06078100	-1.23579200
H	2.13860400	5.38543200	-1.89391400
H	0.38767400	5.31885600	-1.72280600
H	1.39445200	5.60860600	-0.29265300
C	1.40049300	3.55097300	-1.07872900
O	1.57635300	2.81399100	-2.04031600
C	-3.63531600	-3.25210400	-0.54044500
O	-2.82001100	-3.13848800	0.37486300
C	4.92004100	-1.00697100	0.55788100
C	5.81025300	-2.23948800	0.35926400
H	4.84838000	-0.79114700	1.63658000
C	6.75302800	0.50943100	0.44910600
C	7.22849900	-1.90347800	0.83963200
H	5.41282000	-3.05347600	0.98207400
C	7.75669400	-0.62466700	0.18815500
H	6.67107900	0.66916000	1.53612000
H	7.21428300	-1.74498300	1.92823400
H	7.85097300	-0.78723900	-0.89071000
O	5.49361300	0.12909500	-0.08779400
O	9.04367800	-0.38974000	0.77706000
O	8.05009000	-3.02236600	0.53077700
O	5.81406700	-2.61479200	-1.00288600
C	7.10034800	1.83641400	-0.19514900
H	8.10724000	2.15134500	0.10402300
H	7.05935400	1.73431200	-1.28564900
O	6.13185100	2.78357400	0.26382000
H	6.12404200	3.55503800	-0.31283300
H	9.60567500	0.09700400	0.16503800
H	8.96395900	-2.78331000	0.73387100
H	6.48763400	-3.30062000	-1.10721700

M1

C	2.59664300	-1.12382900	0.38079100
C	1.19245500	-1.64127200	0.00783600
C	0.12538100	-0.54926900	0.20340200
C	1.70245700	1.20936000	0.08603500
C	2.88489900	0.27813200	-0.18846100
H	1.19680400	-1.87662500	-1.05986200
H	2.65884500	-1.04706900	1.47233300
H	1.55760600	1.30753800	1.17160300
H	-0.01327800	-0.30749400	1.25951500
O	0.55295100	0.60311200	-0.50217900
O	3.52585100	-2.09487700	-0.09008400
C	1.77377300	2.60420000	-0.51553100
H	1.83399700	2.51717100	-1.61125700
H	2.66477600	3.12755400	-0.16124700
H	4.41825900	-1.74479900	0.05590600
O	0.65323800	3.37439000	-0.12774500
H	-0.13307000	2.80072300	-0.09279900
C	-2.64195000	0.91376400	-0.04600700
C	-4.11405700	1.20726900	0.31974800
C	-2.29218400	-0.56014700	0.24426300
H	-2.50284900	1.08829100	-1.12149500
C	-5.04152500	0.15974600	-0.30626600
H	-4.23191300	1.13416200	1.40619500
C	-3.34878800	-1.51035500	-0.33756700
H	-2.22782400	-0.70488500	1.32782700
H	-5.00717500	0.21464200	-1.40830800
H	-3.30299400	-1.45621900	-1.43637400
O	-4.65890500	-1.13223100	0.10660700
O	-6.33798900	0.41591200	0.14293400
O	-1.07231600	-0.97872700	-0.36956400
C	-3.16234500	-2.96603300	0.07550800
H	-3.92878900	-3.57139900	-0.42524300
H	-2.18162200	-3.30673100	-0.25262900
O	-3.20619700	-3.15615400	1.48069800
H	-4.07604900	-2.88652800	1.80209500
O	-1.75570400	1.77420500	0.65976900
H	-2.25892700	2.40401400	1.21229000
O	4.06853500	0.80714300	0.45517600
C	-7.42057200	-0.12423100	-0.52908700
C	-7.33182800	-1.15479600	-1.46445800
C	-8.65930600	0.43243500	-0.20498000
C	-8.49589100	-1.60777300	-2.08914500
H	-6.38294200	-1.62602600	-1.68383800
C	-9.81039200	-0.03617600	-0.82787200
H	-8.70124200	1.22455600	0.53443800
C	-9.73478900	-1.05583300	-1.77832800
H	-8.42546600	-2.41093200	-2.81557900
H	-10.77038000	0.39964400	-0.57106800
H	-10.63353500	-1.41938000	-2.26439100
H	3.05939000	0.21069600	-1.26766700
N	-4.54236400	2.54470200	-0.09655900
H	-5.26633900	2.59980100	-0.79705000
C	-5.06127100	4.90691400	0.27980200
H	-5.75841500	5.17265400	1.07804600
H	-4.34080500	5.72144600	0.19229100
H	-5.61497100	4.81731600	-0.65671600
N	0.86290300	-2.88008900	0.70404600
H	0.92773800	-3.73172500	0.17144000
C	0.30294000	-4.36624200	2.55422900
H	0.72329800	-4.50255800	3.55030000
H	-0.78615000	-4.43208800	2.63302600
H	0.65148200	-5.17052000	1.90173300
C	0.65909100	-2.98051400	2.05254700
O	0.70800100	-2.01172900	2.79924500
C	-4.32770700	3.64614400	0.67709400
O	-3.57024000	3.61826900	1.64390800
C	5.21689600	0.92440100	-0.32086900
C	6.25311200	1.70769700	0.49582800
H	5.00509100	1.45485100	-1.26410800
C	6.88385200	-0.40416400	-1.42371300
C	7.61751000	1.65743800	-0.19654200
H	5.91825000	2.75059200	0.57419800
C	8.00763600	0.22851300	-0.60265500
H	6.72007900	0.17927400	-2.34097400
H	7.57996900	2.26450600	-1.10711200
H	8.16236300	-0.37111200	0.30450600
O	5.68314000	-0.38239700	-0.63094800
O	9.18645500	0.24596900	-1.39350600
O	8.63400700	2.23996600	0.60969500
O	6.43618400	1.15349500	1.79122300
C	7.12313200	-1.85593600	-1.81081000
H	8.03719800	-1.92222600	-2.39976100
H	7.25615600	-2.45899800	-0.90189000
O	6.07773200	-2.37166800	-2.61987900
H	5.25289300	-2.36935900	-2.11587200
H	9.83813100	0.79385400	-0.93486000
H	8.48332400	1.95633600	1.52328500
H	5.57433500	1.05067700	2.21508600

M2

C	2.61479700	1.15207200	-0.21359500
C	1.20740400	1.55805700	0.26165800
C	0.13961600	0.54736400	-0.19161200
C	1.73811700	-1.20044400	-0.45023000
C	2.90612600	-0.33702000	0.04074000
H	1.20606100	1.54014200	1.35485100
H	2.67934300	1.31721700	-1.29465500
H	1.63095500	-1.08251900	-1.53837500
H	0.00707900	0.56830300	-1.27687600
O	0.55485200	-0.76322600	0.20651000
O	3.53557800	1.99721400	0.46449300
C	1.87886600	-2.67832300	-0.14037400
H	1.87699400	-2.82536200	0.94679600
H	2.81944300	-3.05675300	-0.55488600
H	4.43074300	1.70262500	0.23659000
O	0.76021500	-3.32959600	-0.74241400
H	0.71459900	-4.24641200	-0.45029100
C	-2.61519900	-0.95351300	-0.23958000
C	-4.07533900	-1.17906600	-0.68525900
C	-2.27414900	0.54585800	-0.22361900
H	-2.47897700	-1.34657800	0.77213000
C	-5.06938800	-0.24009000	0.01413500
H	-4.12780800	-0.93589000	-1.74972500
C	-3.35363000	1.34403700	0.51879600
H	-2.19979100	0.91361500	-1.25245700
H	-5.19085000	-0.50348500	1.06969300
H	-3.38019600	1.02107700	1.57072200
O	-4.62182900	1.10570500	-0.09856200
O	-6.28617900	-0.34720200	-0.66629100
O	-1.04811200	0.82939600	0.46674400
C	-3.14253600	2.85307300	0.48679400
H	-3.94332400	3.32852300	1.06732600
H	-2.18762600	3.09659700	0.95038500
O	-3.09796800	3.37871300	-0.83242100
H	-3.92882100	3.15834300	-1.27340100
O	-1.82205400	-1.67693300	-1.17095500
H	-1.00262800	-1.98830900	-0.75732800
O	4.09878900	-0.71923800	-0.68497700
C	-7.45493900	0.03380800	-0.04249500
C	-7.51266100	0.86143700	1.07916100
C	-8.63055400	-0.45194700	-0.61949600
C	-8.75560400	1.17940000	1.62918900
H	-6.60843500	1.27528600	1.50573600
C	-9.86201500	-0.11824100	-0.06740900
H	-8.55904300	-1.08927600	-1.49397300
C	-9.93231700	0.69577900	1.06440700
H	-8.79654200	1.82170000	2.50325000
H	-10.77090900	-0.50107400	-0.52067400
H	-10.89352400	0.95238300	1.49665700
H	3.06445800	-0.50235700	1.11224900
N	-4.46894100	-2.57857900	-0.56601500
H	-4.54331100	-3.09987500	-1.42338600
C	-4.88887000	-4.73685300	0.49244700
H	-4.09172700	-5.32019600	0.95810900
H	-5.80435500	-4.93151000	1.05469700
H	-5.02642200	-5.08168300	-0.53453500
N	0.86802800	2.91865100	-0.13357400
H	0.78465000	3.60304300	0.59942600
C	0.23717000	4.76570400	-1.59020900
H	0.60421700	5.14755000	-2.54214700
H	-0.85672100	4.77988300	-1.61096800
H	0.57543000	5.42031500	-0.78297500
C	0.68877100	3.32896700	-1.42539200
O	0.83740400	2.57461900	-2.37873100
C	-4.54865900	-3.26174300	0.61042000
O	-4.37300400	-2.73061100	1.70032400
C	5.25455500	-0.96343000	0.05328800
C	6.29733600	-1.56081700	-0.90126700
H	5.05557700	-1.66897200	0.87770400
C	6.90742600	0.14818700	1.38932400
C	7.66292300	-1.62855200	-0.21183900
H	5.97634700	-2.57378400	-1.17992300
C	8.03698600	-0.29934600	0.46116500
H	6.75285400	-0.60458500	2.17531800
H	7.63474200	-2.39913100	0.56564600
H	8.18138700	0.46515800	-0.31422600
O	5.70462800	0.26320600	0.60601900
O	9.21612800	-0.45332200	1.23550000
O	8.68264100	-2.03565200	-1.11507700
O	6.47069800	-0.76471100	-2.06441100
C	7.12643900	1.49957700	2.05292600
H	8.04655600	1.46465600	2.63509400
H	7.23889000	2.27267600	1.28031700
O	6.08288800	1.82410200	2.95728500
H	5.25486500	1.92961800	2.46978300
H	9.87816700	-0.88752500	0.68031000
H	8.53511800	-1.57500300	-1.95401300
H	5.60749900	-0.58227600	-2.45758900

M3

C	2.61532400	1.18986000	0.23542100
C	1.19678200	1.35905100	0.81827000
C	0.13074700	0.74323600	-0.10112900
C	1.72624500	-0.71113800	-1.16516200
C	2.88171500	-0.23501100	-0.27997400
H	1.15167200	0.79442400	1.75296800
H	2.72537100	1.86705200	-0.61801800
H	1.64798900	-0.07070400	-2.05378800
H	0.02905000	1.29450800	-1.04206800
O	0.52777900	-0.60379600	-0.39192500
O	3.51665900	1.56151600	1.27102300
C	1.81612400	-2.16542600	-1.61194600
H	1.86353200	-2.81574200	-0.72600800
H	2.72650200	-2.31413400	-2.19374400
H	4.41668500	1.36859400	0.96491200
O	0.74099100	-2.53044100	-2.45626400
H	-0.09271100	-2.44571100	-1.97211300
C	-2.66711200	-1.11790000	0.32414900
C	-4.05254600	-1.50579000	-0.21462200
C	-2.23567600	0.27058700	-0.16218100
H	-2.70713900	-1.08834900	1.41822000
C	-5.07154300	-0.37000000	-0.02360100
H	-3.96382500	-1.65955700	-1.29324300
C	-3.34665500	1.30183400	0.07043800
H	-1.99569100	0.23951100	-1.22862600
H	-5.32133700	-0.23629400	1.03356000
H	-3.50377200	1.42709800	1.15223200
O	-4.54910800	0.84166400	-0.55596000
O	-6.20505800	-0.70814300	-0.76557600
O	-1.07319200	0.68341500	0.58986100
C	-3.05445100	2.66286500	-0.55272500
H	-3.86469200	3.35258100	-0.28416000
H	-2.12027200	3.06034900	-0.15612800
O	-2.89841200	2.58689300	-1.95995800
H	-3.72075100	2.24442600	-2.33403100
O	-1.76630900	-2.13941900	-0.09642900
H	-0.87233200	-1.85105300	0.14390300
O	4.09352000	-0.23072200	-1.06850900
C	-7.41313600	-0.10126600	-0.49102100
C	-7.55748500	1.05510500	0.27560800
C	-8.53351900	-0.71668600	-1.05377500
C	-8.83460900	1.57663300	0.48959000
H	-6.69216700	1.56178400	0.68182300
C	-9.79813900	-0.18027300	-0.84066800

H	-8.39449500	-1.61166300	-1.65013600
C	-9.95755500	0.96800700	-0.06312700
H	-8.94303000	2.47551900	1.08821600
H	-10.66380500	-0.66505300	-1.28064800
H	-10.94506700	1.38410100	0.10477700
H	3.00278500	-0.91764600	0.56853600
N	-4.52345900	-2.76778800	0.34478400
H	-4.53891600	-3.56011100	-0.27534000
C	-5.14210700	-4.40282100	2.04839200
H	-4.35612800	-4.81259100	2.68720600
H	-6.06160600	-4.38074300	2.63545600
H	-5.28158600	-5.06847900	1.19405600
N	0.91652400	2.74798300	1.16358100
H	0.90631900	2.97326900	2.14485300
C	0.81266500	5.16943300	0.86690600
H	1.79173600	5.65550600	0.84325900
H	0.13895900	5.76001500	0.24549900
H	0.44950700	5.17315800	1.89685900
C	0.94393500	3.78143900	0.26855400
O	1.06971700	3.60431900	-0.93515500
C	-4.76983600	-2.98151800	1.66992500
O	-4.69967700	-2.09063300	2.50712900
C	5.21498500	-0.85593900	-0.52607600
C	6.29791900	-0.89263000	-1.61210700
H	4.97534200	-1.88194800	-0.20046800
C	6.80791900	-0.64509600	1.25682400
C	7.63030100	-1.34612000	-1.00813800
H	5.98711600	-1.60028900	-2.39228000
C	7.97700100	-0.57413500	0.27420900
H	6.61570600	-1.69212400	1.53035800
H	7.56453900	-2.40823200	-0.75007000
H	8.16144700	0.47821200	0.01922500
O	5.64116800	-0.10921600	0.60322300
O	9.11820800	-1.14224900	0.89731200
O	8.69000800	-1.24720200	-1.95030900
O	6.52724700	0.39085200	-2.17558400
C	7.00145800	0.16102400	2.53268300
H	7.88997800	-0.19973300	3.04946400
H	7.15913400	1.21796000	2.27712000
O	5.91504700	0.00631400	3.43184200
H	5.11489800	0.37569300	3.03520500
H	9.80221300	-1.24398100	0.22127900
H	8.57937000	-0.41498600	-2.43297600
H	5.68681600	0.76482300	-2.46974800

M4

C	2.55578300	1.35011300	-0.24167600
C	1.17934200	1.95845600	0.10610100
C	0.03473900	1.09021200	-0.44084400
C	1.38900700	-0.85634400	-0.56643500
C	2.62610700	-0.16806500	0.01654100
H	1.07664400	1.94960300	1.19431000
H	2.74110600	1.50193900	-1.31067700
H	1.37380500	-0.70603400	-1.65630400
H	0.00304800	1.09233700	-1.53294400
O	0.25552300	-0.24140400	0.02572700
O	3.51875000	2.06618600	0.52364800
C	1.25923700	-2.34914800	-0.29322000
H	1.19812900	-2.52590900	0.78608000
H	2.13278200	-2.87621500	-0.68495600
H	4.38215000	1.65142700	0.37274900
O	0.10092800	-2.85277200	-0.95568800
H	-0.60257500	-3.07922800	-0.32506400
C	-2.49674400	-0.38341300	-0.77969200
C	-3.95592800	-0.80798600	-1.02613600
C	-2.39232800	1.13554900	-0.54429400
H	-2.11848200	-0.88690100	0.10663600
C	-4.89936300	-0.22451200	0.03064600
H	-4.26392400	-0.37679400	-1.98388200
C	-3.47290500	1.64661800	0.42336300
H	-2.47335300	1.65470900	-1.50527300
H	-4.68129000	-0.63039100	1.02987400
H	-3.23056900	1.27731400	1.43096700
O	-4.77419600	1.18432500	0.04238800
O	-6.20778700	-0.54700600	-0.34805200
O	-1.16688400	1.52716800	0.10378100
C	-3.57345700	3.16811300	0.47132500
H	-4.28814000	3.44794600	1.25628000
H	-2.60039000	3.58861400	0.72216800
O	-3.95009000	3.72849400	-0.77619000
H	-4.80639900	3.35799900	-1.02610100
O	-1.74111100	-0.75402700	-1.92282400
H	-1.09662400	-1.44063700	-1.67877100
O	3.81053100	-0.70065700	-0.62429200
C	-7.22590200	-0.49030300	0.58339700
C	-7.13215600	0.17045700	1.80840900
C	-8.40687100	-1.14297100	0.22365700
C	-8.22408700	0.15139200	2.67811100
H	-6.23798400	0.71829700	2.07484100
C	-9.48988200	-1.14436200	1.09539400
H	-8.45906900	-1.64190700	-0.73790300
C	-9.40311500	-0.50173700	2.33144700
H	-8.14791100	0.66608000	3.63059800
H	-10.40427700	-1.65380000	0.80885100
H	-10.24769000	-0.50563000	3.01190200
H	2.68262200	-0.35673500	1.09436300
N	-4.13530100	-2.25597400	-1.15817500
H	-4.91703100	-2.52222000	-1.73501200
C	-3.88855000	-4.66652300	-0.98722800
H	-3.08869000	-5.14214800	-1.55998500
H	-4.03711800	-5.25671900	-0.08157600
H	-4.80363400	-4.68920700	-1.58209000
N	1.08522600	3.35392400	-0.30609600
H	1.10631100	4.04546600	0.42453400
C	1.08646100	5.28823500	-1.79598800
H	1.94369000	5.59836200	-2.39630800
H	0.18488800	5.53163100	-2.36238900
H	1.07951500	5.85901400	-0.86501100
C	1.14552600	3.78461400	-1.60011900
O	1.22456200	3.01572000	-2.54930700
C	-3.43515300	-3.27255400	-0.59311800
O	-2.48381200	-3.12109500	0.17498300
C	4.85856300	-1.10465300	0.19741200
C	5.88608300	-1.82879800	-0.68284400
H	4.50079000	-1.78262900	0.99052500
C	6.53454300	-0.23222200	1.67761900
C	7.17369300	-2.07927400	0.10689000
H	5.45691100	-2.78833600	-1.00137900
C	7.66411600	-0.81481900	0.82826800
H	6.22341100	-0.96627700	2.43444200
H	6.98577300	-2.84482500	0.86689100
H	7.96329500	-0.06941000	0.07917000
O	5.42196600	0.04792300	0.80778900
O	8.75302100	-1.12689600	1.68341200
O	8.19949400	-2.60903100	-0.72327100
O	6.25191600	-1.05512500	-1.81665200
C	6.87641600	1.07222400	2.38170300
H	7.73604100	0.91164500	3.03127600
H	7.14835600	1.83038200	1.63430600
O	5.81694800	1.52450400	3.21003400
H	5.04568400	1.72604100	2.66326600
H	9.38981800	-1.64292500	1.17020600
H	8.17179700	-2.13274300	-1.56599800
H	5.45197400	-0.75709700	-2.26873100

M5

C	2.59459600	-1.12255800	0.50655400
C	1.20532400	-1.63437700	0.07862200
C	0.13746400	-0.53919800	0.25515600
C	1.72865500	1.21119100	0.18962900
C	2.90978600	0.27262000	-0.06686900
H	1.24342600	-1.85297100	-0.99232000
H	2.61758800	-1.03607600	1.59854500
H	1.56081500	1.30265400	1.27208100
H	-0.02699900	-0.30633900	1.30908800
O	0.58678400	0.61684300	-0.42959500
O	3.54085700	-2.10271400	0.08394000
C	1.82356700	2.60916600	-0.39996500
H	1.91963100	2.53043100	-1.49394400
H	2.70384100	3.12549700	-0.01083300
H	4.42124300	-1.77049400	0.31413700
O	0.69195900	3.38131100	-0.04617700
H	-0.09328800	2.80611700	-0.02700800
C	-2.62512900	0.92647600	-0.02523500
C	-4.10170300	1.21308100	0.32684200
C	-2.27963800	-0.55200800	0.24601900
H	-2.47305900	1.11732200	-1.09632500
C	-5.02068900	0.17856400	-0.33248000
H	-4.23387700	1.11877000	1.40995000
C	-3.32501800	-1.49003200	-0.37415700
H	-2.23668600	-0.71647300	1.32789300
H	-4.97382100	0.25855100	-1.43260100
H	-3.26056100	-1.41184700	-1.47057200
O	-4.64353700	-1.12228800	0.05550600
O	-6.32301200	0.42404500	0.10727700
O	-1.04857200	-0.95847600	-0.35148300
C	-3.14522100	-2.95456000	0.00910500
H	-3.89751800	-3.54955900	-0.52481100
H	-2.15538600	-3.28474100	-0.30206000
O	-3.22383700	-3.17841200	1.40772100
H	-4.09758200	-2.90574400	1.71575800
O	-1.74762500	1.77659000	0.70233000
H	-2.25566000	2.39130900	1.26651500
O	4.08928900	0.78654300	0.58526700
C	-7.39706400	-0.10398000	-0.58642500
C	-7.29642900	-1.10943700	-1.54771500
C	-8.64117200	0.43815200	-0.25768700
C	-8.45351300	-1.55123400	-2.19303100
H	-6.34369200	-1.57049100	-1.77188300
C	-9.78516700	-0.01924900	-0.90150700
H	-8.69244800	1.21039900	0.50189500
C	-9.69747100	-1.01341400	-1.87763800
H	-8.37358800	-2.33477500	-2.93966600
H	-10.74917800	0.40527100	-0.64076700
H	-10.59073900	-1.36835700	-2.37993600
H	3.08663300	0.19523000	-1.14565900
N	-4.52424600	2.55869800	-0.06799000
H	-5.23166500	2.62973900	-0.78369000
C	-5.04741900	4.91354500	0.34631100
H	-5.75955600	5.16378800	1.13629300
H	-4.32448800	5.72860400	0.28942200
H	-5.58313600	4.84397200	-0.60233400
N	0.84961100	-2.88464300	0.74178600
H	0.98147900	-3.72952400	0.21063000
C	0.22850200	-4.40661200	2.54336400
H	0.61939400	-4.55179200	3.55027000
H	-0.86114900	-4.49124700	2.58683400
H	0.61145400	-5.19455900	1.89018300
C	0.57522300	-3.00593000	2.07641500
O	0.55483600	-2.04666600	2.83543800
C	-4.32269700	3.64376000	0.73181400
O	-3.58396900	3.59526800	1.71192200
C	5.21845600	0.90839800	-0.20160600
C	6.32169400	1.65612000	0.54812500
H	4.99177500	1.45385900	-1.13452400
C	6.76651100	-0.41511400	-1.47172600
C	7.58025300	1.66150200	-0.33111700
H	5.99467600	2.69307600	0.70497200
C	7.96508600	0.24969100	-0.77837900
H	6.50530400	0.15344900	-2.37658200
H	7.39073200	2.26276500	-1.23257100
H	8.24415400	-0.33577100	0.10440900
O	5.66189400	-0.40157300	-0.56287600
O	9.09246700	0.41311600	-1.64821000
O	8.61584100	2.26160400	0.43595000
O	6.56946800	1.01812700	1.78368300
C	6.96608000	-1.87680800	-1.85363200
H	7.78146000	-1.96558300	-2.57468600
H	7.23382800	-2.45242500	-0.95529900
O	5.82299600	-2.42072300	-2.48595200
H	5.06634800	-2.35790600	-1.88448900
H	9.61936200	-0.39313600	-1.66605900
H	9.44032700	2.17598600	-0.06055500
H	7.35913600	1.42734000	2.16308200

M6

C	2.61980700	1.19794800	-0.35500400
C	1.22113000	1.57965300	0.16163400
C	0.15167400	0.57304200	-0.29965600
C	1.76818300	-1.14855300	-0.63979400
C	2.92889000	-0.29120300	-0.12160400
H	1.24190500	1.52743900	1.25382900
H	2.66173400	1.37641000	-1.43455000
H	1.65251300	-0.99082300	-1.72150300
H	-0.00801400	0.63160900	-1.37979300
O	0.58350600	-0.74735700	0.04238900
O	3.54964800	2.04762200	0.31023500
C	1.92739900	-2.63467500	-0.38696400
H	1.94900500	-2.82269200	0.69402400
H	2.86276800	-2.98544800	-0.83551100
H	4.43569300	1.79939200	0.00840900
O	0.80223900	-3.27527000	-0.98903900
H	0.78625800	-4.20815400	-0.74967300
C	-2.59662800	-0.95506000	-0.29957600
C	-4.06699600	-1.18101200	-0.70934800
C	-2.26343900	0.54604700	-0.26522700
H	-2.43100700	-1.36526000	0.70129000
C	-5.04668400	-0.26407900	0.03744400
H	-4.15071600	-0.91511600	-1.76636900
C	-3.32675500	1.32246100	0.52257900
H	-2.21918100	0.93331100	-1.28867400
H	-5.13596300	-0.54931400	1.09039900
H	-3.31977600	0.97941200	1.56842300
O	-4.61163200	1.08679500	-0.06109700
O	-6.28243700	-0.36641100	-0.60968600
O	-1.02181900	0.82356500	0.39806600
C	-3.12806400	2.83353700	0.51327100
H	-3.90619000	3.29064900	1.13803000
H	-2.15516200	3.07448300	0.93919500
O	-3.14563800	3.38606600	-0.79519200
H	-3.98701600	3.15304200	-1.20899000
O	-1.82876100	-1.65952400	-1.26478600
H	-0.97966700	-1.94180800	-0.89106100
O	4.13076300	-0.64422500	-0.83641500
C	-7.43456300	-0.00638900	0.05516500
C	-7.46511500	0.80150000	1.19225800
C	-8.62332700	-0.49252900	-0.49415400
C	-8.69316400	1.09855200	1.78559700
H	-6.55150900	1.21585600	1.59778100
C	-9.84001800	-0.17966100	0.10115500
H	-8.57308700	-1.11451200	-1.38107100
C	-9.88253100	0.61401400	1.24873300
H	-8.71269700	1.72552300	2.67147900
H	-10.75912500	-0.56299700	-0.33065700
H	-10.83220400	0.85431200	1.71461500
H	3.06738300	-0.47260500	0.95060200
N	-4.44753100	-2.58594800	-0.61066400
H	-4.51225400	-3.09425000	-1.47664900
C	-4.81356400	-4.77210200	0.40922600
H	-3.99307700	-5.35039300	0.83947400
H	-5.70967900	-4.99717600	0.99090100
H	-4.97214300	-5.09567000	-0.62161800
N	0.86035100	2.94929100	-0.18118200
H	0.81927500	3.61494300	0.57230800
C	0.18519000	4.84140400	-1.55953800
H	0.54048000	5.25613100	-2.50225900
H	-0.90878100	4.85765600	-1.56696200
H	0.53461300	5.46720100	-0.73436200
C	0.63567600	3.39827700	-1.45318100
O	0.74310500	2.67304200	-2.43329500
C	-4.49770400	-3.29370100	0.55276700
O	-4.31606700	-2.78229900	1.65108900
C	5.24706500	-0.92403100	-0.06739400
C	6.37875700	-1.47025000	-0.93990600
H	5.01070900	-1.67045000	0.71206200
C	6.74180300	0.09508100	1.50657600
C	7.62122000	-1.65233200	-0.05528600
H	6.07631300	-2.45205200	-1.33038200
C	7.96790300	-0.37219800	0.70891600
H	6.47360700	-0.67114500	2.24886400
H	7.42881300	-2.44464500	0.68336900
H	8.25000500	0.40401700	-0.01076500
O	5.65795200	0.27223600	0.58925400
O	9.08134100	-0.71119800	1.54416200
O	8.68172900	-2.04639000	-0.91545000
O	6.63691600	-0.57098100	-1.99675700
C	6.89543700	1.43283200	2.22065000
H	7.69112000	1.36837100	2.96589300
H	7.16975100	2.20571300	1.48763500
O	5.72408900	1.79135000	2.92776400
H	4.98605000	1.87136800	2.30524400
H	9.59108600	0.07782600	1.75792500
H	9.49697400	-2.05685500	-0.39671800
H	7.45247100	-0.86229800	-2.42722700

M7

C	2.61738700	1.29741100	0.12095200
C	1.20678900	1.43354300	0.72857700
C	0.13954100	0.80636800	-0.18312800
C	1.75686900	-0.60956000	-1.27415300
C	2.90421400	-0.12876000	-0.38058900
H	1.18557000	0.85662200	1.65699700
H	2.70141400	1.96728100	-0.74076900
H	1.66959200	0.04070300	-2.15421800
H	0.01274800	1.36818500	-1.11439000
O	0.55515300	-0.52944200	-0.49761300
O	3.53022500	1.70378800	1.13664200
C	1.87018500	-2.05574600	-1.73907800
H	1.93087900	-2.71694900	-0.86180400
H	2.78185000	-2.17806700	-2.32462300
H	4.42568000	1.58272000	0.78707300
O	0.80018700	-2.43194100	-2.58636100
H	-0.03475900	-2.35842100	-2.10304700
C	-2.61905400	-1.10817500	0.25359500
C	-4.00715900	-1.50712900	-0.26923300
C	-2.22202400	0.29880200	-0.20949900
H	-2.63995100	-1.10304100	1.34885400
C	-5.04185800	-0.39795100	-0.01779300
H	-3.93922000	-1.62335400	-1.35406600
C	-3.34524500	1.30454900	0.07247100
H	-2.00476000	0.29616200	-1.28137600
H	-5.26386300	-0.29894300	1.04935500
H	-3.47491900	1.40469000	1.16049600
O	-4.55713200	0.83744800	-0.53071600
O	-6.19049500	-0.73666500	-0.73648900
O	-1.05250100	0.71623800	0.52707300
C	-3.09425800	2.68320000	-0.52993700
H	-3.90368400	3.35517600	-0.21692000
H	-2.15162700	3.08490000	-0.15862500
O	-2.98939200	2.63985200	-1.94305800
H	-3.81654500	2.28343400	-2.29275900
O	-1.70838800	-2.10334600	-0.20471900
H	-0.81396400	-1.79530300	0.01038700
O	4.12360800	-0.12072200	-1.14772300
C	-7.40141500	-0.16577200	-0.40519400
C	-7.54501600	0.96797800	0.39476300
C	-8.52674300	-0.79340100	-0.94424500
C	-8.82538800	1.45340400	0.66638500
H	-6.67739400	1.48472500	0.78307400
C	-9.79500100	-0.29274800	-0.67363400
H	-8.38828900	-1.67016600	-1.56720500
C	-9.95303600	0.83198200	0.13791200
H	-8.93313600	2.33465400	1.29083900
H	-10.66436700	-0.78712200	-1.09531200
H	-10.94325200	1.22023600	0.35070400
H	3.00787300	-0.80520000	0.47586200
N	-4.44048500	-2.79690900	0.25655400
H	-4.43566600	-3.57093600	-0.38646400
C	-4.98541200	-4.50298200	1.91465800
H	-4.17809300	-4.90990600	2.52819500
H	-5.89596900	-4.52666600	2.51546600
H	-5.11905000	-5.14476400	1.04128300
N	0.90271400	2.81164300	1.09720100
H	0.93618900	3.02923600	2.07970400
C	0.74695300	5.23396300	0.83027300
H	1.71622200	5.73659800	0.77271900
H	0.03937100	5.81927800	0.24243600
H	0.42506500	5.22126600	1.87389700
C	0.87743700	3.85410200	0.21213600
O	0.95606700	3.69126100	-0.99683300
C	-4.65990000	-3.05973000	1.57734200
O	-4.60335200	-2.19500000	2.44249800
C	5.20891700	-0.77079500	-0.58393200
C	6.37183100	-0.83644700	-1.57520400
H	4.93894600	-1.79896500	-0.28501700
C	6.64517300	-0.68880600	1.33709100
C	7.58077500	-1.45448900	-0.85647700
H	6.08328000	-1.49070300	-2.40943600
C	7.89916000	-0.72666600	0.45207400
H	6.34841400	-1.71524100	1.59793000
H	7.36158700	-2.50498000	-0.61427700
H	8.21013100	0.29766000	0.21951300
O	5.59500800	-0.06324800	0.59217700
O	8.97850800	-1.45663900	1.04686800
O	8.67210300	-1.38923400	-1.76409100
O	6.66819900	0.46356400	-2.03944300
C	6.77610200	0.12447900	2.61963000
H	7.54022900	-0.31573600	3.26357300
H	7.08417000	1.15010900	2.36842000
O	5.57874500	0.12131800	3.37320300
H	4.86839100	0.51495600	2.84532600
H	9.48506800	-0.88534500	1.63429400
H	9.46612000	-1.68056400	-1.29684800
H	7.49284200	0.40705100	-2.54141800

M8

C	2.56528500	1.39881600	-0.34753900
C	1.19557700	1.98558800	0.05625000
C	0.04449000	1.13148300	-0.50127900
C	1.41295300	-0.79739400	-0.72915800
C	2.65009700	-0.12514300	-0.12743500
H	1.11531700	1.93075800	1.14525400
H	2.72414600	1.57861300	-1.41597800
H	1.38426300	-0.59981800	-1.81077400
H	-0.01109500	1.18017100	-1.59120500
O	0.28116100	-0.21656700	-0.09621200
O	3.54806700	2.10409000	0.40785800
C	1.29777400	-2.30111900	-0.51931400
H	1.27204000	-2.52824900	0.55217000
H	2.16055600	-2.80285600	-0.96372200
H	4.41123500	1.73223000	0.17388300
O	0.12029500	-2.78444900	-1.16543700
H	-0.56327700	-3.03147100	-0.52133000
C	-2.48390000	-0.34746200	-0.83890700
C	-3.94614300	-0.77557200	-1.06236000
C	-2.38547500	1.16158100	-0.54379100
H	-2.07803900	-0.88319200	0.01571500
C	-4.86304200	-0.24765500	0.04548000
H	-4.28557900	-0.30665900	-1.99122300
C	-3.44318100	1.62142700	0.47365900
H	-2.49719600	1.71848800	-1.48028000
H	-4.61096000	-0.69300500	1.01951700
H	-3.16750000	1.21422300	1.45778900
O	-4.75108600	1.16078600	0.11293100
O	-6.17996000	-0.56761900	-0.30629100
O	-1.14763800	1.53836400	0.08799000
C	-3.55755800	3.13846800	0.58756800
H	-4.24524700	3.37791200	1.40932200
H	-2.57994900	3.55960300	0.81891600
O	-3.98704200	3.74476900	-0.62083000
H	-4.84402900	3.36745700	-0.85797000
O	-1.75812000	-0.66633000	-2.01594900
H	-1.09408500	-1.34807600	-1.81389800
O	3.83745900	-0.62886100	-0.77466400
C	-7.16873300	-0.55831200	0.65704200
C	-7.04463600	0.05818300	1.90240200
C	-8.35325400	-1.21156000	0.30977300
C	-8.10898000	-0.00559600	2.80358700
H	-6.14863700	0.60679300	2.16091700
C	-9.40877500	-1.25775400	1.21343000
H	-8.42976400	-1.67528000	-0.66765600
C	-9.29105800	-0.65965800	2.46912900
H	-8.00894000	0.47472300	3.77172600
H	-10.32597800	-1.76739100	0.93624500
H	-10.11420700	-0.69856200	3.17433100
H	2.69542500	-0.33888500	0.94678000
N	-4.11382500	-2.21809800	-1.25352300
H	-4.89299000	-2.46910000	-1.84037100
C	-3.83217900	-4.63032000	-1.19053700
H	-3.03049900	-5.06673500	-1.79129900
H	-3.96337500	-5.26310000	-0.31135300
H	-4.75228400	-4.64016900	-1.77802000
N	1.08300100	3.39713500	-0.29282700
H	1.14688800	4.05654300	0.46454000
C	1.04524700	5.39534400	-1.69596600
H	1.90231800	5.72697000	-2.28532100
H	0.14331900	5.66914700	-2.24735600
H	1.04589000	5.92413700	-0.74035800
C	1.09645900	3.88360800	-1.56860000
O	1.13095400	3.15791200	-2.55290100
C	-3.39615900	-3.24860600	-0.73750200
O	-2.44278700	-3.11736600	0.03148100
C	4.84682100	-1.07439000	0.05867200
C	5.95051700	-1.76117200	-0.74753800
H	4.45477500	-1.79033800	0.80301200
C	6.35409500	-0.29636300	1.75561500
C	7.08868800	-2.12966900	0.21561800
H	5.54201000	-2.68391400	-1.18229700
C	7.55587000	-0.92347600	1.03408600
H	5.92829700	-1.02595700	2.46022300
H	6.73438000	-2.89797700	0.91876000
H	7.99812700	-0.18559700	0.35578100
O	5.37442400	0.04439600	0.77049500
O	8.54630400	-1.43062200	1.93719900
O	8.14348900	-2.65723800	-0.57779700
O	6.40581200	-0.89044500	-1.76139800
C	6.64475700	0.99611600	2.50871600
H	7.37198800	0.80998800	3.30196500
H	7.07269200	1.73257300	1.81268400
O	5.49100400	1.50740200	3.14835600
H	4.81153300	1.69403400	2.48348500
H	9.14978700	-0.72721800	2.19974000
H	8.90761500	-2.79431500	-0.00245600
H	7.19475200	-1.29189800	-2.15068300

N1

C	2.67387900	-0.78285700	-0.72457100
C	1.26783700	-1.41087800	-0.86917300
C	0.17285700	-0.48295500	-0.32688700
C	1.49137000	1.45098500	-0.64902300
C	2.70136900	0.68719900	-1.20050600
H	1.06881900	-1.51689000	-1.93998800
H	2.95959600	-0.79190300	0.32391600
H	1.57941800	1.49300000	0.44631400
H	0.26088700	-0.33047200	0.75722100
O	0.29847700	0.75049300	-1.00173100
O	3.57863900	-1.56795000	-1.48889400
C	1.29607600	2.86759500	-1.16583100
H	1.08827200	2.82469000	-2.24534200
H	2.20384700	3.45380300	-1.01583800
H	4.20482600	-2.00709100	-0.88634100
O	0.24655100	3.51914000	-0.47238700
H	-0.43031100	2.86421800	-0.22534200
C	-2.59035900	0.82729400	0.07075500
C	-4.01923900	1.06468600	0.60692100
C	-2.20375500	-0.66543500	0.16313400
H	-2.55301700	1.12118400	-0.98605800
C	-5.00033100	0.08279100	-0.03782900
H	-4.03239300	0.86791700	1.68406500
C	-3.32514500	-1.57683100	-0.36356700
H	-1.99291200	-0.91013300	1.20997900
H	-5.05308500	0.23401100	-1.12978000
H	-3.38767800	-1.45364100	-1.45552900
O	-4.58431500	-1.23773800	0.23051400
O	-6.25533800	0.29005300	0.53445300
O	-1.07784300	-1.02136400	-0.64745100
C	-3.10794100	-3.05435700	-0.05394800
H	-3.91810000	-3.63152600	-0.51738800
H	-2.16343800	-3.37867500	-0.48829500
O	-3.02658900	-3.31985300	1.33589400
H	-3.86693700	-3.07545000	1.74431300
O	-1.64795500	1.62328900	0.78170400
H	-2.09905900	2.15712400	1.46553600
O	3.84390600	1.42869900	-0.72298100
C	-7.38902000	-0.19578500	-0.09445200
C	-7.37543200	-1.14324400	-1.11746000
C	-8.59732500	0.32510200	0.37200600
C	-8.58557100	-1.54877800	-1.68449800
H	-6.44716800	-1.58864200	-1.44980000
C	-9.79449900	-0.09595900	-0.19562700
H	-8.58006500	1.05165200	1.17684900
C	-9.79535400	-1.03187400	-1.23136000
H	-8.57359800	-2.28700700	-2.47982300
H	-10.73070200	0.31151100	0.17169100
H	-10.73008600	-1.35809500	-1.67404600
H	2.70172700	0.70771800	-2.29792500
N	-4.47540500	2.43890000	0.39267600
H	-5.24767100	2.57898500	-0.24102500
C	-4.93225200	4.74010500	1.09475100
H	-5.51156900	4.94208700	1.99844900
H	-4.20481100	5.54649200	0.98730800
H	-5.60334800	4.74904800	0.23393500
N	1.18042100	-2.76280600	-0.31056900
H	0.55904600	-3.38016700	-0.80668700
C	1.47670900	-4.74243900	1.07111000
H	0.77445500	-5.19701500	0.36972800
H	2.40784700	-5.31286600	1.05686700
H	1.06329600	-4.81414700	2.07892100
C	1.79688000	-3.28772700	0.78044800
O	2.57450900	-2.66350100	1.50282200
C	-4.19000900	3.43659500	1.27664400
O	-3.35936500	3.28631900	2.16994700
C	5.11564500	0.86609900	-0.83876200
C	6.15094700	1.98605700	-0.66964700
H	5.24261000	0.37663900	-1.81526200
C	6.50412400	-0.78767200	0.13391500
C	7.55407200	1.37950900	-0.53706200
H	6.11706300	2.63956800	-1.55212900
C	7.60704800	0.21585700	0.46905000
H	6.66696100	-1.17501600	-0.88446200
H	7.86491500	0.98654300	-1.51083200
H	7.42991700	0.60821400	1.47934400
O	5.26097900	-0.09302900	0.18980700
O	8.86865800	-0.43486500	0.40285200
O	8.51954700	2.37039600	-0.19961900
O	5.92394100	2.75157500	0.50763200
C	6.36805100	-1.97895600	1.06955600
H	7.29299500	-2.55798600	1.06251500
H	6.18025100	-1.63310900	2.09101800
O	5.30151800	-2.81726000	0.61403700
H	4.52881800	-2.74325600	1.19691800
H	9.55132400	0.24852000	0.44810200
H	8.12114100	2.94989000	0.46613600
H	4.97646100	2.91760300	0.59668900

N2

C	-2.72194600	-0.62029100	0.76614800
C	-1.30524600	-1.15179000	1.07015500
C	-0.21092000	-0.26903400	0.45976900
C	-1.61138500	1.63317800	0.40658000
C	-2.82241700	0.90350500	1.00532700
H	-1.16831300	-1.10636600	2.15554500
H	-2.96059800	-0.80637000	-0.27783200
H	-1.64695200	1.52193500	-0.68730500
H	-0.23797100	-0.29775400	-0.63848500
O	-0.40855600	1.06359400	0.90560100
O	-3.63568500	-1.30091900	1.61219400
C	-1.54144000	3.11278400	0.72886300
H	-1.38249300	3.24521000	1.80548300
H	-2.47367500	3.60465100	0.43870600
H	-4.22275100	-1.85950900	1.07068300
O	-0.43546200	3.63656400	-0.01603500
H	-0.23628500	4.53206400	0.27738300
C	2.57377600	0.95168300	-0.11695100
C	3.95515700	1.03755600	-0.80515000
C	2.16332000	-0.51860800	0.07467100
H	2.62432100	1.42962500	0.86476900
C	4.99927000	0.08376800	-0.20421800
H	3.81461300	0.71626200	-1.84047200
C	3.30437300	-1.30777300	0.72974100
H	1.94109100	-0.96009200	-0.90317700
H	5.32363200	0.42059700	0.78563000
H	3.52708800	-0.87071000	1.71522300
O	4.45853900	-1.22988700	-0.11025800
O	6.07775600	0.04359900	-1.09230800
O	1.02737500	-0.69564800	0.93260300
C	3.01074300	-2.78904600	0.92840800
H	3.89527300	-3.26072100	1.37367800
H	2.17973600	-2.90348900	1.62365700
O	2.63264800	-3.45441000	-0.26918500
H	3.36907500	-3.40272200	-0.89198300
O	1.67610600	1.64882600	-0.97050200
H	1.04356100	2.16948800	-0.45152200
O	-3.96178600	1.51868200	0.36680500
C	7.31761700	-0.36425200	-0.64674900
C	7.53021600	-1.09849500	0.52026900
C	8.39547700	-0.00985100	-1.46118200
C	8.83266100	-1.45503000	0.87423800
H	6.69659400	-1.41276400	1.13435500
C	9.68615000	-0.38108100	-1.10173400
H	8.20348500	0.55710300	-2.36545600
C	9.91370300	-1.10217500	0.07157400
H	8.99455900	-2.02432500	1.78407900
H	10.51869300	-0.10050300	-1.73904700
H	10.92144300	-1.38833400	0.35283800
H	-2.88310300	1.08742800	2.08583500
N	4.44525100	2.40960100	-0.86659500
H	4.44073200	2.85210400	-1.77016100
C	5.20010800	4.59045600	-0.07286500
H	4.61548800	5.27481500	0.54433200
H	6.24649600	4.70175100	0.22071400
H	5.09853200	4.88021000	-1.12087800
N	-1.09911100	-2.55082300	0.68538800
H	-0.31271600	-2.98100000	1.14719500
C	-1.08204500	-4.68195800	-0.45712800
H	-1.49234400	-5.33160300	0.32202000
H	-1.37409100	-5.08426900	-1.42539100
H	0.00789400	-4.69079600	-0.37599200
C	-1.65612600	-3.28253100	-0.31700700
O	-2.56570600	-2.88510900	-1.04594800
C	4.75996600	3.16764100	0.22165300
O	4.71370600	2.73456200	1.36668600
C	-5.22498600	0.94029600	0.52867300
C	-6.28132000	1.98104500	0.13427300
H	-5.37681700	0.61889300	1.56913000
C	-6.52589200	-0.89829600	-0.19352600
C	-7.66111700	1.31419900	0.04684500
H	-6.30613100	2.77312600	0.89509100
C	-7.63779000	-0.00681700	-0.74382000
H	-6.72081000	-1.10278900	0.87100200
H	-8.00957000	1.08412300	1.05914100
H	-7.42435900	0.21055700	-1.79903300
O	-5.30017600	-0.17971100	-0.32673100
O	-8.88342400	-0.67756800	-0.61638500
O	-8.63272100	2.20156100	-0.49682900
O	-6.01914200	2.55036600	-1.14406200
C	-6.31850500	-2.23345600	-0.89002700
H	-7.23726300	-2.82021700	-0.83500900
H	-6.06995900	-2.07142600	-1.94377700
O	-5.27448900	-2.94837700	-0.22437700
H	-4.47839900	-3.00318200	-0.77760500
H	-9.58144500	-0.03750600	-0.81160400
H	-8.22249700	2.66227300	-1.24319300
H	-5.06703200	2.68004300	-1.24122700

N3			
C	2.73118000	0.31417400	0.82945900
C	1.30371300	0.70237600	1.27803600
C	0.24150400	0.12019300	0.34184300
C	1.65943500	-1.69408500	-0.31422600
C	2.84364600	-1.19767900	0.52764800
H	1.13275800	0.23526700	2.25301800
H	2.98187200	0.85301600	-0.08064500
H	1.72237700	-1.24287900	-1.31344100
H	0.32079500	0.52289100	-0.67830200
O	0.43253800	-1.29215000	0.30424400
O	3.61890300	0.66931500	1.87754500
C	1.57996700	-3.20813900	-0.46668500
H	1.45340600	-3.66541600	0.52508700
H	2.50920200	-3.58146700	-0.89514200
H	4.22237200	1.36781800	1.56457700
O	0.54416300	-3.60171700	-1.35024000
H	-0.31108200	-3.32610600	-0.99236200
C	-2.88539900	-1.16920900	0.62506800
C	-4.22783500	-1.40432800	-0.08560300
C	-2.14853400	0.03814400	0.03567400
H	-3.07353400	-0.96452300	1.68484700
C	-5.03954700	-0.10327500	-0.20839300
H	-4.01401200	-1.72999100	-1.10693500
C	-3.07392200	1.25879900	-0.03043500
H	-1.79740000	-0.19510600	-0.97332400
H	-5.40182400	0.22977600	0.76901100
H	-3.36474900	1.55146700	0.99018700
O	-4.23268300	0.91571500	-0.79479400
O	-6.10327200	-0.35985800	-1.07374000
O	-1.02124700	0.36379500	0.87680000
C	-2.44686200	2.46013200	-0.72860700
H	-3.16181200	3.29200800	-0.70777100
H	-1.54735600	2.76748800	-0.19459200
O	-2.04597900	2.16773500	-2.05781400
H	-2.83162700	1.91911600	-2.56209100
O	-2.13506600	-2.37258400	0.49371500
H	-1.23877000	-2.19271400	0.81740700
O	3.99831700	-1.54784400	-0.26144100
C	-7.22156700	0.44872800	-1.03963200
C	-7.24674500	1.72802600	-0.48437700
C	-8.36952900	-0.08756600	-1.62621800
C	-8.43746600	2.45636600	-0.50315100
H	-6.35046600	2.16566600	-0.06464000
C	-9.54572100	0.65344400	-1.64697500
H	-8.32268900	-1.08284300	-2.05410500
C	-9.58854800	1.92866600	-1.08085800
H	-8.45449100	3.45069400	-0.06833200
H	-10.43489300	0.22952700	-2.10258500
H	-10.50796500	2.50396400	-1.09455900
H	2.88921100	-1.74099300	1.48085300
N	-4.99539300	-2.47662900	0.53714000
H	-5.04550800	-3.34718100	0.03470700
C	-6.13217600	-3.71778900	2.30510700
H	-5.53156400	-4.13699700	3.11584000
H	-7.11053100	-3.46841800	2.71889400
H	-6.25626100	-4.47863700	1.53175900
N	1.12860800	2.14074200	1.48829900
H	0.48744100	2.37667600	2.22846400
C	1.36957900	4.55855300	1.39201500
H	2.26182100	5.00175100	1.84090100
H	1.07584100	5.19406600	0.55511000
H	0.57133200	4.55530000	2.13705100
C	1.73185700	3.18449000	0.85880200
O	2.52977800	3.06402100	-0.06941600
C	-5.46850700	-2.44390500	1.81699600
O	-5.37082500	-1.45434400	2.53170100
C	5.25219100	-1.06100600	0.11872400
C	6.32685700	-1.89832200	-0.58744100
H	5.38027700	-1.12117300	1.20936200
C	6.55105200	0.91708300	0.10744600
C	7.70155200	-1.24334600	-0.39706300
H	6.33877500	-2.90633900	-0.15082000
C	7.68488800	0.26913700	-0.68556600
H	6.72086100	0.74675200	1.18235000
H	8.01767700	-1.37627800	0.64291400
H	7.50051200	0.42685200	-1.75654700
O	5.33439100	0.28802000	-0.29088800
O	8.91874300	0.85814900	-0.30059200
O	8.69782600	-1.88701900	-1.18337300
O	6.10721900	-1.98089100	-1.99052600
C	6.34707700	2.40929300	-0.10001800
H	7.25115800	2.94662100	0.19107400
H	6.14105300	2.61665500	-1.15476300
O	5.26380600	2.85068100	0.72533800
H	4.48902300	3.08377200	0.18978600
H	9.62948700	0.32673600	-0.68465500
H	8.31094200	-2.07233300	-2.05150500
H	5.17007900	-2.14521000	-2.15572700

N4			
C	2.61604900	0.80781600	0.67552500
C	1.25634200	1.53381400	0.81345200
C	0.13079200	0.75765500	0.12138200
C	1.26625700	-1.30369200	0.29913700
C	2.50490600	-0.70644300	0.97867500
H	1.00433100	1.55029100	1.87840900
H	2.97204200	0.91004400	-0.34634700
H	1.40851200	-1.23726200	-0.79000900
H	0.27838000	0.69615500	-0.96440000
O	0.12595800	-0.54793000	0.67735500
O	3.52701000	1.41719900	1.57891900
C	0.94548700	-2.75259100	0.64003000
H	0.72241500	-2.84365600	1.70801400
H	1.80105000	-3.38915300	0.40857300
H	4.22663400	1.86848300	1.07344500
O	-0.16479500	-3.18534800	-0.15042100
H	-0.96461200	-3.29582700	0.38814200
C	-2.44881500	-0.48812800	-0.56841900
C	-3.88169100	-0.79343900	-1.04551200
C	-2.23746000	1.02661600	-0.38744300
H	-2.27710400	-0.96703000	0.39250400
C	-4.93053100	-0.06696500	-0.19742800
H	-3.97927700	-0.39346500	-2.05963300
C	-3.40520900	1.67658900	0.37354600
H	-2.12496300	1.49541500	-1.37114100
H	-4.93602900	-0.43700700	0.83861800
H	-3.37060300	1.32658500	1.41615000
O	-4.66225200	1.32321000	-0.21317300
O	-6.17642200	-0.28066400	-0.79621700
O	-1.09182900	1.35410400	0.42420600
C	-3.36548100	3.20136400	0.37109500
H	-4.18456800	3.57306500	1.00008000
H	-2.42282200	3.54270300	0.79731000
O	-3.44319500	3.74867600	-0.93494100
H	-4.27472700	3.46049900	-1.33309900
O	-1.55057600	-0.99587700	-1.54345500
H	-1.04726400	-1.73743000	-1.16195500
O	3.61051900	-1.48094100	0.46758800
C	-7.33357800	-0.06703800	-0.07258700
C	-7.39018400	0.64861300	1.12333400
C	-8.49502300	-0.61364000	-0.62227800
C	-8.61715800	0.79269700	1.77376700
H	-6.50347600	1.11434500	1.53252900
C	-9.71151500	-0.45329400	0.03139300
H	-8.42736000	-1.15863800	-1.55747100
C	-9.77926700	0.24666600	1.23706800
H	-8.65801800	1.34998500	2.70417400
H	-10.60987500	-0.88119000	-0.40169500
H	-10.72845400	0.36894500	1.74735400
H	2.45032800	-0.84569800	2.06612600
N	-4.17457400	-2.22436100	-1.14683000
H	-4.84037300	-2.46170100	-1.86434500
C	-4.21389200	-4.62872500	-0.79283800
H	-3.36207000	-5.22127800	-1.13493800
H	-4.62363700	-5.12503000	0.08841000
H	-4.97026600	-4.61818500	-1.57996100
N	1.29467000	2.93577800	0.38695900
H	0.61253600	3.52029400	0.84248700
C	1.79465800	5.02145700	-0.75658100
H	2.71516300	5.55865500	-0.51669900
H	1.56952100	5.21235400	-1.80729600
H	0.98502700	5.42086800	-0.14304300
C	2.04238700	3.53494100	-0.57538800
O	2.88416000	2.95635200	-1.26355700
C	-3.71375000	-3.25376700	-0.38966300
O	-2.93269000	-3.12546600	0.55373600
C	4.91428000	-1.05402600	0.73146400
C	5.86537400	-2.23278600	0.48566800
H	5.00805400	-0.70064800	1.76869100
C	6.49797000	0.57527000	0.06888300
C	7.31916000	-1.74233100	0.51451700
H	5.71743800	-2.98232400	1.27519900
C	7.53705000	-0.47229200	-0.32769000
H	6.61671000	0.81350800	1.13767100
H	7.59254300	-1.50227900	1.54735200
H	7.40050400	-0.71892300	-1.38912100
O	5.20987900	0.00594800	-0.15466500
O	8.83893800	0.05000400	-0.10192500
O	8.22135200	-2.76697900	0.11010200
O	5.66001900	-2.82928800	-0.78998400
C	6.52762200	1.88345400	-0.70585000
H	7.49863900	2.36421800	-0.57494200
H	6.37460500	1.69011900	-1.77247000
O	5.51331100	2.75393500	-0.19726900
H	4.78067400	2.84210900	-0.82841500
H	9.46661400	-0.67945800	-0.19660000
H	7.82807000	-3.21778900	-0.65131200
H	4.71123300	-2.88684400	-0.96119500

N5

C	2.66704800	-0.77206200	-0.66475800
C	1.26586800	-1.40841800	-0.80132900
C	0.15978700	-0.48262900	-0.27833600
C	1.47008200	1.45409200	-0.58931200
C	2.68815500	0.69958600	-1.13658100
H	1.07246400	-1.53296700	-1.87142600
H	2.96514100	-0.78115000	0.38009700
H	1.55064400	1.49002300	0.50621500
H	0.23173600	-0.32675400	0.80654900
O	0.28124000	0.74697900	-0.95494900
O	3.56844000	-1.55175300	-1.44297600
C	1.27959700	2.87321900	-1.09805600
H	1.10580000	2.84143100	-2.18419200
H	2.17970600	3.45844600	-0.90754500
H	4.19805600	-1.99681100	-0.85110700
O	0.19940300	3.51060100	-0.43514900
H	-0.45895300	2.84236500	-0.17742300
C	-2.60015500	0.80809600	0.10387300
C	-4.03631000	1.04571100	0.61941800
C	-2.22460400	-0.68799100	0.17722600
H	-2.54143000	1.11979900	-0.94684700
C	-5.01477300	0.08300100	-0.05686300
H	-4.06882900	0.83092300	1.69253600
C	-3.34155300	-1.57822300	-0.39297600
H	-2.04090800	-0.95337000	1.22430600
H	-5.05476300	0.25675200	-1.14605000
H	-3.38396500	-1.42590700	-1.48222800
O	-4.60879800	-1.24580700	0.18810500
O	-6.27515200	0.28393000	0.50591000
O	-1.08277900	-1.03849500	-0.61228300
C	-3.14081600	-3.06518100	-0.12030600
H	-3.94948300	-3.62256100	-0.60991800
H	-2.19379500	-3.38621800	-0.55175300
O	-3.08029600	-3.36976400	1.26270700
H	-3.92212400	-3.12394400	1.66722700
O	-1.66638600	1.58652700	0.84384300
H	-2.12271300	2.10218400	1.53722100
O	3.81171100	1.44928100	-0.64635700
C	-7.40382100	-0.18101800	-0.14606700
C	-7.38439000	-1.10914100	-1.18664200
C	-8.61430900	0.33981400	0.31508700
C	-8.59014400	-1.49490600	-1.77632800
H	-6.45500500	-1.55474400	-1.51554500
C	-9.80721200	-0.06161600	-0.27523300
H	-8.60171000	1.05119600	1.13344200
C	-9.80183700	-0.97779800	-1.32848000
H	-8.57342900	-2.21792800	-2.58544700
H	-10.74501900	0.34591600	0.08798000
H	-10.73328200	-1.28858400	-1.78889400
H	2.69028000	0.72010500	-2.23496500
N	-4.47518600	2.42748900	0.42502900
H	-5.20806100	2.59787100	-0.24660200
C	-4.91331700	4.72366400	1.15454500
H	-5.50753100	4.92825800	2.04781900
H	-4.16633300	5.51507100	1.07172900
H	-5.56504100	4.75658500	0.27949500
N	1.17419100	-2.75169400	-0.21786200
H	0.48741200	-3.34313700	-0.65655200
C	1.45047900	-4.72460300	1.17452000
H	0.68599200	-5.15181700	0.52292200
H	2.34986000	-5.34121600	1.11447000
H	1.09458700	-4.76367500	2.20580500
C	1.82468800	-3.29060700	0.84484100
O	2.67168800	-2.70055200	1.51740300
C	-4.20285600	3.40263600	1.33748600
O	-3.40729300	3.21897000	2.25595600
C	5.08423700	0.92264000	-0.78640600
C	6.13760400	2.02687500	-0.64344100
H	5.20413000	0.43899800	-1.76828300
C	6.49981500	-0.76867900	0.09146900
C	7.52878200	1.37261000	-0.65693500
H	6.05838400	2.69865400	-1.51012400
C	7.63932000	0.22642100	0.35262600
H	6.60135800	-1.16238700	-0.93289400
H	7.72045600	0.95526400	-1.65653900
H	7.54317100	0.63157200	1.36592500
O	5.27511400	-0.06322000	0.22408700
O	8.94776800	-0.33058300	0.15764200
O	8.47381800	2.40086300	-0.38145100
O	5.92768100	2.74195600	0.55695900
C	6.40458200	-1.95735400	1.03783900
H	7.33532200	-2.53252900	1.01233300
H	6.23739400	-1.60519100	2.06160000
O	5.35122300	-2.82086700	0.61828500
H	4.57399000	-2.74124300	1.19677200
H	9.22889200	-0.79926800	0.95079100
H	9.33792400	1.98323100	-0.26944800
H	6.70391800	3.30281900	0.69120900

N6

C	-2.70032900	-0.64119100	0.63563500
C	-1.28111800	-1.18459200	0.89981100
C	-0.17706700	-0.28456300	0.33085700
C	-1.58100600	1.59885600	0.24809400
C	-2.78856000	0.88473300	0.86746500
H	-1.14120800	-1.19168000	1.98617600
H	-2.98268100	-0.83075200	-0.39689100
H	-1.61035100	1.45012100	-0.84103300
H	-0.16919800	-0.30819900	-0.76800100
O	-0.38198600	1.04178600	0.77568800
O	-3.58693500	-1.30874600	1.52412500
C	-1.52010300	3.08872700	0.51215800
H	-1.39978700	3.27073100	1.58702300
H	-2.44057600	3.56043700	0.16078800
H	-4.20483300	-1.86149300	1.01646900
O	-0.38267100	3.57864600	-0.21206400
H	-0.24845300	4.51235000	-0.01820500
C	2.61311700	0.93866900	-0.16787100
C	4.01873100	1.02600300	-0.80757300
C	2.21198300	-0.53369300	0.02716600
H	2.62288200	1.43026300	0.80835100
C	5.05066800	0.08216000	-0.16993800
H	3.91327700	0.69853400	-1.84510500
C	3.33730600	-1.29585900	0.73853100
H	2.03749200	-0.98951800	-0.95406800
H	5.34977000	0.43190900	0.82320500
H	3.53139000	-0.82638000	1.71511100
O	4.51329900	-1.23298300	-0.07216200
O	6.15100700	0.03624800	-1.03129800
O	1.04622100	-0.72234300	0.83885900
C	3.04126600	-2.76967200	0.98178600
H	3.92905700	-3.23214600	1.42973900
H	2.21818700	-2.86075700	1.68979900
O	2.64410700	-3.47039500	-0.19088300
H	3.37503600	-3.45069200	-0.82171000
O	1.74358600	1.61511700	-1.06457800
H	1.09569600	2.15217900	-0.58088200
O	-3.92088500	1.50217500	0.23673900
C	7.38153100	-0.35770400	-0.55003300
C	7.56929100	-1.07671200	0.63075000
C	8.47750500	-0.00570000	-1.34114100
C	8.86453900	-1.42069300	1.02149300
H	6.72208000	-1.38868600	1.22714500
C	9.76098200	-0.36433800	-0.94501500
H	8.30470200	0.54986100	-2.25628600
C	9.96330000	-1.07026400	0.24208800
H	9.00707000	-1.97811000	1.94188800
H	10.60766600	-0.08551100	-1.56425200
H	10.96542000	-1.34635500	0.55212800
H	-2.82643600	1.07369600	1.94897500
N	4.50217500	2.40098800	-0.86102200
H	4.50186200	2.84543400	-1.76368300
C	5.20281700	4.59469800	-0.05175900
H	4.56998800	5.26984800	0.52720200
H	6.22903500	4.73372800	0.29565400
H	5.14919600	4.87340700	-1.10627400
N	-1.05729100	-2.55948800	0.43135900
H	-0.16821100	-2.93251800	0.72915700
C	-1.11858100	-4.68799300	-0.72214700
H	-1.65681200	-5.43944000	-0.13710000
H	-1.25402300	-4.93343200	-1.77558600
H	-0.05706500	-4.74284900	-0.47333900
C	-1.73897300	-3.32694000	-0.45835000
O	-2.79090100	-3.00048700	-1.01075900
C	4.78113800	3.16438800	0.23344500
O	4.71738400	2.73107000	1.37746800
C	-5.18540700	0.98467700	0.47470200
C	-6.25432300	2.03227400	0.14302600
H	-5.29175600	0.68476300	1.52890000
C	-6.57783300	-0.85351200	-0.07797400
C	-7.63699200	1.37208800	0.26063800
H	-6.18998400	2.84107000	0.88529200
C	-7.72560600	0.06222300	-0.52669800
H	-6.67869100	-1.04529000	1.00217100
H	-7.83520300	1.13880900	1.31703300
H	-7.62265600	0.27781800	-1.59607000
O	-5.35982300	-0.16832200	-0.33939900
O	-9.03061900	-0.46211800	-0.24420600
O	-8.58936200	2.32327800	-0.20330100
O	-6.04604300	2.54274400	-1.15853300
C	-6.46393800	-2.20290600	-0.77303000
H	-7.40468000	-2.75427600	-0.67356600
H	-6.25525700	-2.05589700	-1.83846000
O	-5.44046200	-2.97071900	-0.15010100
H	-4.65299900	-3.05042800	-0.71436700
H	-9.29178600	-1.08735300	-0.92885500
H	-9.44711200	1.88079700	-0.25107900
H	-6.84761900	3.02692500	-1.40122700

N7

C	2.72512100	0.32615900	0.78940000
C	1.29871600	0.71443100	1.23676400
C	0.23304300	0.12777000	0.30688200
C	1.65423200	-1.67261400	-0.36370500
C	2.83827100	-1.18435300	0.48373300
H	1.13080500	0.25200700	2.21464700
H	2.98117600	0.86598000	-0.11845500
H	1.71409100	-1.20548100	-1.35500100
H	0.30389200	0.53330000	-0.71289100
O	0.42535600	-1.28148500	0.26549400
O	3.61040000	0.67790500	1.84387800
C	1.58767500	-3.18335400	-0.54371200
H	1.49048900	-3.66381400	0.44079000
H	2.51163500	-3.52844600	-1.00482100
H	4.20939900	1.38120600	1.53899100
O	0.53346600	-3.57265300	-1.40923600
H	-0.31461300	-3.30623500	-1.02904500
C	-2.88637500	-1.16861000	0.61433500
C	-4.23319700	-1.40796000	-0.08697400
C	-2.16058200	0.04427100	0.02194600
H	-3.06749900	-0.96629000	1.67606300
C	-5.05408800	-0.11171200	-0.19442900
H	-4.02543700	-1.72550000	-1.11209400
C	-3.09374400	1.25997000	-0.02831200
H	-1.81879300	-0.18324600	-0.99163000
H	-5.40916200	0.21357500	0.78811900
H	-3.37585500	1.54457600	0.99711300
O	-4.25877600	0.91539600	-0.78256700
O	-6.12461600	-0.36905500	-1.05155600
O	-1.02704700	0.37339900	0.85218300
C	-2.48255400	2.47035400	-0.72477200
H	-3.20183500	3.29808800	-0.68666700
H	-1.57745800	2.77776700	-0.20027000
O	-2.09815400	2.19262500	-2.06165300
H	-2.88746200	1.93330500	-2.55478900
O	-2.13323900	-2.36817200	0.47493900
H	-1.22849700	-2.18153600	0.77177100
O	3.98129200	-1.53704300	-0.30912300
C	-7.24533500	0.43472400	-1.00518900
C	-7.27362100	1.70884400	-0.43808300
C	-8.39390100	-0.10084500	-1.59155100
C	-8.46751100	2.43218300	-0.44476800
H	-6.37745900	2.14652900	-0.01822500
C	-9.57330000	0.63521400	-1.60011000
H	-8.34466900	-1.09192200	-2.02880900
C	-9.61903800	1.90500400	-1.02206600
H	-8.48672800	3.42238400	-0.00065600
H	-10.46270900	0.21165100	-2.05563600
H	-10.54094900	2.47648400	-1.02629400
H	2.87580100	-1.72958700	1.43731900
N	-4.98774000	-2.49038500	0.53459500
H	-5.01683500	-3.36360500	0.03500300
C	-6.09081300	-3.75573600	2.30629000
H	-5.47116400	-4.17209600	3.10415200
H	-7.06602400	-3.52384000	2.73717300
H	-6.21563700	-4.51259100	1.52914800
N	1.11572100	2.15439000	1.43873900
H	0.43669300	2.38668500	2.14574500
C	1.32651100	4.57383400	1.34232600
H	2.19587000	5.03715500	1.81501300
H	1.04725100	5.19726700	0.49139800
H	0.50526200	4.56145300	2.06183500
C	1.73041400	3.20261500	0.83092000
O	2.56825700	3.09365300	-0.06387700
C	-5.45345300	-2.46868200	1.81729600
O	-5.36963500	-1.47928900	2.53407400
C	5.23270100	-1.07266300	0.06614400
C	6.33049300	-1.90547700	-0.60452300
H	5.35834600	-1.12483100	1.15871400
C	6.53686500	0.90985100	0.11374400
C	7.68935700	-1.25834600	-0.29113400
H	6.31628100	-2.91558300	-0.17076600
C	7.70786600	0.23811200	-0.61709300
H	6.66082600	0.75412400	1.19754200
H	7.90125300	-1.36768900	0.78270800
H	7.58672000	0.37033500	-1.69776200
O	5.33998700	0.28864400	-0.33396400
O	8.99606400	0.70638600	-0.19405700
O	8.66676300	-1.96654500	-1.04441300
O	6.10711000	-1.96298600	-1.99831900
C	6.34797900	2.40187400	-0.11872400
H	7.25407400	2.94408600	0.16869300
H	6.14550100	2.59197400	-1.17817300
O	5.27820600	2.87446000	0.69746400
H	4.49179400	3.08740000	0.16826100
H	9.22813900	1.51190900	-0.66850500
H	9.50804400	-1.50124700	-0.94742800
H	6.90570400	-2.33480400	-2.39753900

N8

C	2.61097700	0.80658700	0.62721100
C	1.25410300	1.53496500	0.76757500
C	0.11961300	0.75877400	0.09005100
C	1.25478400	-1.29659500	0.24022700
C	2.49887600	-0.71013400	0.91878000
H	1.01099300	1.56081200	1.83463100
H	2.97415700	0.91403300	-0.39149100
H	1.38828700	-1.21048200	-0.84806800
H	0.25447400	0.70052200	-0.99764900
O	0.11680900	-0.54520000	0.64178500
O	3.51916600	1.40784100	1.54227700
C	0.94561000	-2.75330900	0.55423300
H	0.77043600	-2.87894400	1.62783500
H	1.78912100	-3.37813700	0.25958800
H	4.21783500	1.86795600	1.04695600
O	-0.20374900	-3.16402700	-0.19604100
H	-0.98198800	-3.25960800	0.37456800
C	-2.46127000	-0.47693400	-0.58719400
C	-3.89835300	-0.78207100	-1.05457600
C	-2.25489400	1.03615600	-0.38969200
H	-2.28038700	-0.96619400	0.36675900
C	-4.94258900	-0.07380900	-0.18613100
H	-4.00802100	-0.36810500	-2.06165300
C	-3.41560200	1.66509000	0.39909600
H	-2.16274000	1.51888000	-1.36887700
H	-4.93920400	-0.46128200	0.84328900
H	-3.36635000	1.29339400	1.43357400
O	-4.67803200	1.31791600	-0.17985000
O	-6.19301300	-0.27893900	-0.77877500
O	-1.09814100	1.36261100	0.40642100
C	-3.38412100	3.18960700	0.42939300
H	-4.19915100	3.54281600	1.07415300
H	-2.43958000	3.52725600	0.85439200
O	-3.47797000	3.76555700	-0.86354600
H	-4.30945200	3.47668800	-1.26138100
O	-1.57144000	-0.97187800	-1.57543600
H	-1.06475500	-1.71834200	-1.20604600
O	3.58615100	-1.48650800	0.39074400
C	-7.34423000	-0.07794300	-0.04346300
C	-7.39227200	0.61508500	1.16623600
C	-8.51029300	-0.61280200	-0.59531200
C	-8.61475700	0.74872900	1.82722200
H	-6.50212700	1.07138900	1.57852700
C	-9.72224700	-0.46297200	0.06912700

H	-8.44910900	-1.14088600	-1.54058500
C	-9.78125800	0.21461600	1.28803300
H	-8.64859900	1.28832200	2.76831200
H	-10.62400300	-0.88175100	-0.36589800
H	-10.72693600	0.32860700	1.80674200
H	2.44515400	-0.85459400	2.00658900
N	-4.17861500	-2.21335300	-1.17560800
H	-4.80447900	-2.45668800	-1.92599800
C	-4.18447700	-4.62135300	-0.83904800
H	-3.30927700	-5.18952900	-1.16320600
H	-4.60736700	-5.13715000	0.02454400
H	-4.91802700	-4.61892800	-1.64764100
N	1.28034400	2.93381300	0.32365900
H	0.54165800	3.49310700	0.71957100
C	1.75099900	5.02344000	-0.82159000
H	2.63153100	5.60673500	-0.54250200
H	1.57477800	5.18775400	-1.88611200
H	0.89157500	5.39393700	-0.25984000
C	2.05617900	3.55231000	-0.60186400
O	2.96318500	3.00766200	-1.23371700
C	-3.72825300	-3.23980900	-0.40733400
O	-2.98737300	-3.10111400	0.56589400
C	4.88642000	-1.09420800	0.66615000
C	5.85509200	-2.26043900	0.44008700
H	4.97897700	-0.74920100	1.70782800
C	6.48491900	0.56283500	0.09224300
C	7.29065200	-1.73610600	0.60496700
H	5.67042900	-3.02137400	1.21198400
C	7.55370200	-0.48739600	-0.24139000
H	6.55309600	0.81006200	1.16388900
H	7.45535300	-1.46425900	1.65798100
H	7.48491300	-0.75199100	-1.30236000
O	5.21798200	-0.01131900	-0.19590000
O	8.88919600	-0.07815500	0.08747400
O	8.16442400	-2.80089300	0.24600100
O	5.66146700	-2.80821700	-0.84813800
C	6.54446100	1.86860900	-0.68756200
H	7.52237500	2.34148500	-0.55199000
H	6.39507000	1.67144300	-1.75476500
O	5.55556000	2.76546800	-0.19110900
H	4.81211900	2.85732800	-0.81111500
H	9.25107900	0.46812200	-0.61854500
H	9.06399600	-2.44862900	0.22775400
H	6.40764600	-3.39978800	-1.01763200

O1

C	2.53455400	-1.15287600	0.14381900
C	1.11288200	-1.66175400	-0.17855500
C	0.05165100	-0.57571000	0.06146200
C	1.61713000	1.18657100	-0.09179900
C	2.80203100	0.26623100	-0.39505500
H	1.07978800	-1.88819600	-1.24755600
H	2.64810300	-1.10719000	1.23336800
H	1.49887000	1.27825600	0.99766600
H	-0.05244500	-0.33861800	1.12232100
O	0.45599700	0.58014500	-0.65312500
O	3.42526200	-2.11208900	-0.40706600
C	1.66918200	2.58510500	-0.68710700
H	1.69946200	2.50343900	-1.78453800
H	2.56873900	3.10827400	-0.35542800
H	4.33536000	-1.79928200	-0.27959300
O	0.55840000	3.35151900	-0.26498700
H	-0.22470200	2.77542500	-0.20541400
C	-2.72181200	0.88735700	-0.08708400
C	-4.18170800	1.17783500	0.32630200
C	-2.36381900	-0.58884900	0.18118300
H	-2.61603000	1.06990500	-1.16501300
C	-5.12627600	0.13718500	-0.28522300
H	-4.26741100	1.09145000	1.41480100
C	-3.43779000	-1.53512200	-0.37517200
H	-2.26576200	-0.74184100	1.26111500
H	-5.11923000	0.20203800	-1.38723000
H	-3.42227300	-1.47542600	-1.47452500
O	-4.73524800	-1.15836200	0.10665200
O	-6.41107000	0.39070800	0.19846800
O	-1.16438500	-1.00286200	-0.47404500
C	-3.24148600	-2.99293600	0.02549000
H	-4.01960800	-3.59570600	-0.46031900
H	-2.26868800	-3.33151700	-0.32750800
O	-3.25230900	-3.19051000	1.43019500
H	-4.11403100	-2.92174500	1.77344000
O	-1.81326300	1.74277200	0.59611100
H	-2.29776900	2.36392900	1.17438200
O	3.97193300	0.81912600	0.25296700
C	-7.51025200	-0.14753400	-0.44734600
C	-7.44432300	-1.16526900	-1.39858600
C	-8.74175700	0.39737800	-0.07859500
C	-8.62428200	-1.61695400	-1.99366200
H	-6.50013200	-1.62833100	-1.65272200
C	-9.90858600	-0.06986500	-0.67251400
H	-8.76568000	1.17936200	0.67232800
C	-9.85626200	-1.07657800	-1.63822000
H	-8.57142300	-2.41013200	-2.73245500
H	-10.86273500	0.35681500	-0.38093200
H	-10.76729800	-1.43913300	-2.10162400
H	2.97415300	0.22505100	-1.47693400
N	-4.62193900	2.52013400	-0.06115300
H	-5.37027500	2.58259300	-0.73490800
C	-5.12851700	4.87751400	0.35957800
H	-5.79757300	5.13570400	1.18389700
H	-4.41048600	5.69229900	0.25568000
H	-5.71395500	4.79799400	-0.55836100
N	0.81273000	-2.90852000	0.51741200
H	0.90703300	-3.75682200	-0.01630300
C	0.33726000	-4.41487900	2.37536900
H	0.80571400	-4.55189800	3.34978700
H	-0.74552000	-4.49973100	2.50538100
H	0.66773600	-5.20833900	1.70068800
C	0.64598000	-3.01885300	1.87008500
O	0.68810600	-2.05251300	2.62048500
C	-4.38307000	3.61219300	0.71867700
O	-3.59572100	3.57323700	1.66088600
C	5.17521600	0.84123400	-0.43694100
C	6.08081200	1.85395200	0.28196200
H	5.02754500	1.15121000	-1.48451600
C	7.01930400	-0.55199300	-1.07344300
C	7.51658500	1.76465500	-0.23694000
H	5.68097800	2.85916100	0.09291500
C	7.99455300	0.31207500	-0.26923800
H	6.94344200	-0.17052900	-2.10209300
H	7.56189400	2.16397500	-1.25538300
H	8.03157500	-0.07771800	0.75588800
O	5.73610900	-0.47328800	-0.43165600
O	9.28540900	0.22337400	-0.86993300
O	8.40593100	2.56597200	0.53133000
O	6.14429300	1.60865000	1.67943200
C	7.42739900	-2.02563400	-1.13984500
H	7.63866200	-2.39448900	-0.12612200
H	6.59730500	-2.60886400	-1.54320900
O	8.52519300	-2.23131800	-2.00876800
H	9.23868700	-1.63198600	-1.74484700
H	9.85343700	0.88806100	-0.45675600
H	8.14607500	2.48481100	1.46091200
H	5.24822800	1.48308300	2.01872600

02

C	-2.55780700	1.09038000	-0.08478000
C	-1.12976700	1.50670100	-0.48988500
C	-0.07064900	0.50652300	-0.00113500
C	-1.65313500	-1.25840000	0.19278500
C	-2.82303500	-0.40802300	-0.31811000
H	-1.08089600	1.49665900	-1.58196200
H	-2.68799900	1.28118300	0.98607000
H	-1.57535300	-1.14732900	1.28421900
H	0.01859800	0.51743500	1.08844300
O	-0.45866800	-0.80362900	-0.42794800
O	-3.43481400	1.90407800	-0.84753700
C	-1.76845600	-2.73579000	-0.13174200
H	-1.73707400	-2.87306000	-1.21981800
H	-2.71475400	-3.12872100	0.25420700
H	-4.34774200	1.69200400	-0.59530700
O	-0.65776500	-3.37896600	0.49354600
H	-0.59458700	-4.29314500	0.19653600
C	2.70721100	-0.95727200	0.14017700
C	4.14962400	-1.16429900	0.64757500
C	2.33851500	0.53544100	0.13263800
H	2.62409900	-1.33476900	-0.88324900
C	5.15647000	-0.19453300	0.01135700
H	4.14935700	-0.94063700	1.71759700
C	3.43502800	1.36545300	-0.54682700
H	2.21017600	0.88484300	1.16252300
H	5.33301600	-0.43804400	-1.04118100
H	3.51587100	1.06019300	-1.60134300
O	4.67821800	1.14034500	0.12421500
O	6.34157100	-0.28983600	0.74735300
O	1.13999800	0.80579400	-0.60858900
C	3.19285800	2.86946600	-0.50224100
H	4.00831000	3.36897400	-1.04074100
H	2.25383200	3.10071400	-1.00305200
O	3.08031000	3.37517100	0.82072300
H	3.89609900	3.16740100	1.29466800
O	1.88737900	-1.71180700	1.02248600
H	1.08693400	-2.02090400	0.57147900
O	-4.00266800	-0.83210800	0.40404100
C	7.53024900	0.12872200	0.18935800
C	7.62527000	0.96704400	-0.92188800
C	8.68644300	-0.32955000	0.82530900
C	8.88677300	1.32374600	-1.40153300
H	6.73471500	1.36000800	-1.39443000
C	9.93603800	0.04246400	0.34295200
H	8.58576400	-0.97571600	1.69037800
C	10.04448600	0.86792100	-0.77752300
H	8.95668900	1.97433700	-2.26759700
H	10.82949400	-0.31908200	0.84207400
H	11.02001500	1.15477000	-1.15525600
H	-2.97390800	-0.58734500	-1.38967200
N	4.57479900	-2.55410100	0.52078500
H	4.61270700	-3.09179900	1.37042900
C	5.06913200	-4.68702200	-0.55664100
H	4.28497700	-5.27178200	-1.04256200
H	5.99563500	-4.86714500	-1.10518900
H	5.19131200	-5.04277300	0.46856200
N	-0.82397000	2.86868500	-0.07175100
H	-0.76803300	3.56833800	-0.79285900
C	-0.29990800	4.71656900	1.42701500
H	-0.73214200	5.08133200	2.35821000
H	0.78988900	4.76091400	1.51237000
H	-0.60860600	5.36757100	0.60520400
C	-0.69898000	3.26837600	1.22944200
O	-0.85116200	2.49759200	2.16917800
C	4.71761200	-3.21366300	-0.66316700
O	4.58527500	-2.66421200	-1.75013400
C	-5.22692100	-0.88001300	-0.25256700
C	-6.12415900	-1.83063800	0.55686800
H	-5.10900100	-1.26072000	-1.28040100
C	-7.07476700	0.49074800	-0.92670400
C	-7.57368300	-1.75835400	0.07426000
H	-5.74235100	-2.85210600	0.42295000
C	-8.03577800	-0.30508300	-0.03946300
H	-7.03354200	0.04195500	-1.92966900
H	-7.65336000	-2.22295400	-0.91395200
H	-8.03704600	0.15103800	0.95854700
O	-5.77252200	0.43761600	-0.31793700
O	-9.34154900	-0.23730000	-0.60827300
O	-8.44721700	-2.49808500	0.91815900
O	-6.14315100	-1.49436700	1.93613900
C	-7.46936500	1.96229000	-1.07789200
H	-7.65227300	2.39776700	-0.08551100
H	-6.64468500	2.51018300	-1.53809900
O	-8.58509800	2.12285400	-1.93245600
H	-9.30134900	1.55742900	-1.60832600
H	-9.90802800	-0.86202400	-0.13510000
H	-8.17101100	-2.34702300	1.83414300
H	-5.24043600	-1.32041100	2.23388200

O3

C	2.55376200	1.08596500	0.35883300
C	1.12571400	1.33431800	0.89393300
C	0.06664400	0.66149200	0.00998400
C	1.63364800	-0.93398200	-0.86645000
C	2.79965500	-0.38010800	-0.04149300
H	1.05117400	0.85313700	1.87242000
H	2.70102400	1.69385400	-0.54061500
H	1.56987500	-0.39945200	-1.82380900
H	0.01299800	1.10995700	-0.98762300
O	0.43485700	-0.71883400	-0.12014400
O	3.42890000	1.50686600	1.39311000
C	1.70028500	-2.43193800	-1.14181200
H	1.73036500	-2.97491700	-0.18560300
H	2.61158000	-2.66356400	-1.69398500
H	4.34335200	1.32912400	1.11871400
O	0.62400500	-2.87442300	-1.94655400
H	-0.21131700	-2.71953600	-1.48269700
C	-2.79475600	-1.07658300	0.53445400
C	-4.16454200	-1.49419500	-0.02082900
C	-2.30234000	0.23205000	-0.09423900
H	-2.88263900	-0.91616800	1.61433300
C	-5.15802800	-0.32079700	-0.00435000
H	-4.03343800	-1.76974200	-1.07051000
C	-3.39069500	1.31142400	-0.04593900
H	-2.01483300	0.06269500	-1.13571300
H	-5.43976300	-0.05494400	1.01938900
H	-3.58568800	1.58300600	1.00218200
O	-4.58233700	0.80456900	-0.65756900
O	-6.27529500	-0.71711300	-0.74215800
O	-1.16223100	0.70495800	0.65682600
C	-3.03330400	2.56839100	-0.83346900
H	-3.82630000	3.31255300	-0.68563700
H	-2.09686500	2.98704000	-0.46514100
O	-2.84294100	2.29760800	-2.21225800
H	-3.66399600	1.92334300	-2.55782000
O	-1.90418400	-2.16113300	0.28174300
H	-1.01377600	-1.86441900	0.52505700
O	3.98553900	-0.47514200	-0.86127900
C	-7.47359800	-0.05247700	-0.58293600
C	-7.60794800	1.19034400	0.03616800
C	-8.59324400	-0.70253300	-1.10685700
C	-8.87558500	1.76494000	0.14453600
H	-6.74075400	1.71992700	0.40778100
C	-9.84790100	-0.11337100	-1.00095000
H	-8.46167800	-1.66531400	-1.58821100
C	-9.99805800	1.12271000	-0.36992100
H	-8.97645500	2.73108200	0.62876500
H	-10.71340000	-0.62532300	-1.40936000
H	-10.97803100	1.57991500	-0.28538900
H	2.93550400	-0.98654800	0.86231900
N	-4.69279000	-2.67488000	0.65304800
H	-4.69529400	-3.53282500	0.12723100
C	-5.42799300	-4.09443100	2.49765800
H	-4.69547600	-4.43475200	3.23326100
H	-6.38422600	-3.99105500	3.01323700
H	-5.52411600	-4.85523500	1.72026500
N	0.87699400	2.75388400	1.11692000
H	0.91928900	3.06778300	2.07283200
C	0.83753300	5.14039100	0.60330000
H	1.83143200	5.58928600	0.52424400
H	0.17388600	5.69540300	-0.06022700
H	0.49058500	5.24834400	1.63320500
C	0.91388500	3.70044800	0.13141700
O	1.00337600	3.41100400	-1.05383600
C	-5.00209300	-2.73283100	1.98081600
O	-4.94758300	-1.75462800	2.71542400
C	5.17573000	-0.89993300	-0.28219300
C	6.09262500	-1.35113700	-1.42981200
H	4.99982300	-1.74172300	0.40765200
C	7.01119100	-0.13383800	1.05621000
C	7.51222000	-1.60386800	-0.92027000
H	5.67959300	-2.27837800	-1.84929700
C	8.00023000	-0.42569800	-0.07657200
H	6.90804100	-1.02004100	1.69878400
H	7.52180500	-2.50289200	-0.29566100
H	8.06718100	0.46694900	-0.71128700
O	5.74065700	0.18117000	0.45909500
O	9.27340600	-0.71203400	0.49772400
O	8.41652700	-1.86598300	-1.98564900
O	6.20229300	-0.36194500	-2.44249800
C	7.42960200	1.04736800	1.93528500
H	7.67753400	1.90916000	1.29969700
H	6.59221000	1.33166600	2.57567500
O	8.49887300	0.70983900	2.79730500
H	9.21967200	0.34742600	2.26171300
H	9.84999700	-1.04770300	-0.20229700
H	8.19554200	-1.26830600	-2.71508800
H	5.31942600	-0.05282100	-2.68384100

04

C	2.48435600	1.33433400	-0.04330200
C	1.09441500	1.95047400	0.23795200
C	-0.03697600	1.07689500	-0.32313500
C	1.30686900	-0.87736200	-0.36468600
C	2.54004700	-0.18404200	0.22169300
H	0.95645900	1.96711300	1.32206900
H	2.72308000	1.48378100	-1.10226600
H	1.31515400	-0.75614700	-1.45831400
H	-0.03636400	1.05014500	-1.41505800
O	0.16453000	-0.24274700	0.18546000
O	3.39692600	2.04842400	0.77715400
C	1.16343400	-2.36195200	-0.05401100
H	1.07629300	-2.50764200	1.02817500
H	2.04295700	-2.90398700	-0.40930800
H	4.28666500	1.68703800	0.63533600
O	0.01862500	-2.87940900	-0.72935700
H	-0.70223800	-3.08191100	-0.11042800
C	-2.56676100	-0.39456200	-0.69878000
C	-4.02035600	-0.81732500	-0.97812900
C	-2.45903900	1.13006300	-0.50343400
H	-2.21891000	-0.87447000	0.21274100
C	-4.99332100	-0.19689800	0.02956500
H	-4.29481600	-0.41267400	-1.95743100
C	-3.56677500	1.67576600	0.41327400
H	-2.50480200	1.62200500	-1.48100700
H	-4.81177000	-0.57438900	1.04708800
H	-3.35982000	1.33466500	1.43853900
O	-4.85865000	1.21052300	0.00407300
O	-6.29075200	-0.52266700	-0.38360600
O	-1.25305600	1.53180200	0.17335600
C	-3.65826000	3.19857600	0.41413000
H	-4.39470100	3.50538800	1.16836200
H	-2.69038000	3.62019100	0.68248000
O	-3.99296900	3.72473000	-0.85996100
H	-4.84351900	3.35176100	-1.12537500
O	-1.77995800	-0.80267500	-1.80756700
H	-1.14598700	-1.48421500	-1.52461600
O	3.70988100	-0.75747800	-0.40924400
C	-7.34069600	-0.42705900	0.50812900
C	-7.28257100	0.26517900	1.71789400
C	-8.51688800	-1.07376700	0.12261300
C	-8.40605600	0.28442300	2.54645900
H	-6.39115000	0.80795900	2.00316100
C	-9.63119800	-1.03696100	0.95308300

H	-8.54062900	-1.59813200	-0.82646400
C	-9.58084400	-0.36198100	2.17382100
H	-8.35767200	0.82398000	3.48693600
H	-10.54162300	-1.54176100	0.64638400
H	-10.44994900	-0.33572700	2.82214800
H	2.59051800	-0.36456600	1.30229600
N	-4.20633100	-2.26744300	-1.07301400
H	-4.97440800	-2.54504000	-1.66272300
C	-3.98314200	-4.67346200	-0.82604800
H	-3.17433900	-5.16704200	-1.37045600
H	-4.15284600	-5.23973300	0.09102300
H	-4.88613700	-4.70705700	-1.43867700
N	1.02363500	3.33719300	-0.20761700
H	1.07687600	4.04390800	0.50681600
C	1.08617200	5.23498100	-1.74231500
H	1.95007600	5.51063200	-2.34976300
H	0.19017200	5.48592900	-2.31438700
H	1.09245300	5.82741900	-0.82497500
C	1.10957400	3.73532500	-1.51063800
O	1.17989000	2.94285600	-2.44102000
C	-3.53079500	-3.27182000	-0.45841000
O	-2.60114000	-3.10503600	0.33261900
C	4.84266500	-0.99532700	0.35706800
C	5.68973000	-2.02531200	-0.40660900
H	4.57347800	-1.39730800	1.34776500
C	6.76666700	0.09002900	1.28748100
C	7.07260400	-2.16946400	0.22973400
H	5.16342200	-2.98874500	-0.36973600
C	7.70689300	-0.79803600	0.46744100
H	6.55736900	-0.38353800	2.25762600
H	6.97913800	-2.67662300	1.19554400
H	7.88215300	-0.30969200	-0.49959400
O	5.54385200	0.23519100	0.54542900
O	8.93810800	-0.93182700	1.17497600
O	7.93143500	-2.98882700	-0.55377400
O	5.90763900	-1.63938000	-1.75560600
C	7.33135900	1.48831100	1.55118200
H	7.67231200	1.93182300	0.60503300
H	6.54058100	2.12422100	1.95406800
O	8.36310900	1.46644700	2.51896100
H	9.02588900	0.81677700	2.24242200
H	9.46526400	-1.61122300	0.73267100
H	7.77591500	-2.77274900	-1.48505600
H	5.06830100	-1.36219500	-2.14590900

P1

C	2.38362700	1.22712600	-0.96539800
C	0.85859200	1.46911300	-0.89752500
C	0.12788000	0.61143100	-1.95328300
C	1.85766400	-1.06528200	-1.88305900
C	2.66420900	-0.27378300	-0.84255400
H	0.53234700	1.14546500	0.09712200
H	2.76677300	1.56455600	-1.93502100
H	2.23306500	-0.80594400	-2.88554400
H	0.39741100	0.96554100	-2.95692200
O	0.46496200	-0.74917700	-1.79967400
O	3.03765400	1.92458100	0.07341900
C	1.95292100	-2.57937100	-1.70175900
H	2.99465700	-2.88634300	-1.80595200
H	1.38350200	-3.06140300	-2.50762600
H	3.14804500	2.83310800	-0.25671600
O	1.51236600	-3.01265700	-0.43172000
H	0.54526700	-2.92067800	-0.38349100
C	-2.91562500	1.36844100	-0.31350300
C	-3.95558100	0.86613100	0.71345900
C	-2.01763200	0.21626000	-0.80912300
H	-3.44592100	1.77728000	-1.18651100
C	-4.67404700	-0.37188800	0.15219400
H	-3.42101500	0.55938000	1.61979300
C	-2.86332500	-0.99053500	-1.25602700
H	-1.34955000	-0.09524900	-0.00378100
H	-5.26902200	-0.09235900	-0.73218800
H	-3.45023800	-0.69671600	-2.13991200
O	-3.75137500	-1.37256900	-0.19160200
O	-5.51717600	-0.85607800	1.15967700
O	-1.28010500	0.71070900	-1.93462800
C	-2.09246700	-2.25756600	-1.59981800
H	-2.80174100	-3.00349900	-1.97710900
H	-1.35571500	-2.04566200	-2.36850000
O	-1.37785900	-2.78252800	-0.48489500
H	-2.00423200	-3.01133300	0.21303000
O	-2.05720500	2.34545700	0.23891600
H	-2.62534500	3.08855300	0.54537900
O	4.04918800	-0.55657900	-1.10219000
C	-6.54617200	-1.73050200	0.84241000
C	-6.68479400	-2.36512900	-0.39078900
C	-7.47157700	-1.95418900	1.86284700
C	-7.77516900	-3.21212500	-0.59965700
H	-5.94745600	-2.23439700	-1.17153200
C	-8.54722900	-2.80718900	1.64267000
H	-7.33247500	-1.46058400	2.81853900
C	-8.70814300	-3.43724900	0.40774400
H	-7.88159600	-3.70651800	-1.55973700
H	-9.26282800	-2.97904600	2.43994300
H	-9.54812200	-4.10161000	0.23736500
H	2.40265900	-0.61638200	0.16153900
N	-5.02566300	1.81506400	1.07274800
H	-5.83923200	1.34376000	1.44272100
C	-6.28861700	3.77551700	1.75808100
H	-6.07582400	4.23272500	2.72718100
H	-6.60421000	4.57497000	1.08595400
H	-7.10809900	3.06443500	1.87495300
N	0.43892300	2.86202500	-1.04792600
H	-0.53166800	2.98337100	-0.77594100
C	0.34370100	5.25513300	-1.42036700
H	0.76081900	5.97176800	-0.70982000
H	0.45775200	5.68563500	-2.41764000
H	-0.71902300	5.12253000	-1.21107200
C	1.15415600	3.97268200	-1.35173500
O	2.36812200	3.99797400	-1.56516700
C	-5.00941100	3.16621700	1.22096200
O	-4.05379900	3.89127100	0.94459100
C	4.77686000	-1.09080600	-0.03086000
C	6.11133800	-1.61019800	-0.57746100
H	4.22308400	-1.92357600	0.43437600
C	5.69468400	-0.45798400	2.07853200
C	7.02324900	-1.99499100	0.59164700
H	5.92166700	-2.49479700	-1.20062700
C	7.09895800	-0.89124000	1.65764700
H	5.17780200	-1.29451100	2.57268300
H	6.63199800	-2.89967000	1.06828800
H	7.61495500	-0.02280600	1.22629000
O	4.97233000	-0.06738900	0.90902400
O	7.79510000	-1.35854200	2.80592600
O	8.33132300	-2.33736900	0.14036400
O	6.80043500	-0.62467400	-1.33818100
C	5.65216600	0.77329400	2.98096700
H	6.04419300	0.52866300	3.96817200
H	6.28479400	1.55770200	2.54200100
O	4.32442200	1.23507500	3.15400400
H	3.94676000	1.39039400	2.27355300
H	8.62200200	-1.75723300	2.50252200
H	8.58738800	-1.68591900	-0.52863300
H	6.15519300	-0.10115700	-1.83127000

P2

C	2.37961600	1.10672000	-0.96374900
C	0.85560500	1.35829400	-0.89777400
C	0.11529000	0.45045200	-1.90234400
C	1.82401400	-1.23643200	-1.73544100
C	2.66362100	-0.38881200	-0.76550200
H	0.53457200	1.08627600	0.11422200
H	2.76121100	1.38939200	-1.95038000
H	2.19291400	-1.05220900	-2.75593300
H	0.39147700	0.73982000	-2.92479200
O	0.43452200	-0.90016600	-1.66323600
O	3.03171600	1.87459700	0.03062700
C	1.90391500	-2.73511900	-1.45009500
H	2.94326700	-3.05595600	-1.52726500
H	1.33299400	-3.26673600	-2.22321000
H	3.14667400	2.75605700	-0.36670200
O	1.45219500	-3.07790600	-0.15496100
H	0.48697500	-2.96813400	-0.11751600
C	-2.93233100	1.33678300	-0.33073700
C	-4.00953500	0.90867900	0.69305800
C	-2.04562600	0.14667900	-0.75262500
H	-3.43466200	1.71400700	-1.23416100
C	-4.73176800	-0.34605900	0.17679900
H	-3.50757300	0.64417700	1.63083100
C	-2.89943500	-1.07401900	-1.14298200
H	-1.38928400	-0.12750000	0.07561000
H	-5.28901700	-0.10633000	-0.74325400
H	-3.46309500	-0.82774400	-2.05604600
O	-3.81665800	-1.37844200	-0.07764000
O	-5.62101700	-0.75924700	1.17700000
O	-1.29262800	0.56992600	-1.89632700
C	-2.13788400	-2.36840200	-1.39148400
H	-2.84947800	-3.13001500	-1.73142900
H	-1.38670900	-2.21360200	-2.15983800
O	-1.44585200	-2.82686700	-0.23404100
H	-2.08529300	-3.01293900	0.46459300
O	-2.06422000	2.32037700	0.19427700
H	-2.62001600	3.08051100	0.48003300
O	4.02626500	-0.70872800	-1.07374300
C	-6.65070500	-1.63552400	0.86820000
C	-6.75268200	-2.33506700	-0.33318000
C	-7.61854200	-1.78955800	1.86198200
C	-7.84819800	-3.17623500	-0.53838200
H	-5.98384000	-2.25883200	-1.09035300
C	-8.69907000	-2.63758400	1.64637400

H	-7.50821900	-1.24618500	2.79414500
C	-8.82313300	-3.33197800	0.44207800
H	-7.92562400	-3.72111600	-1.47360500
H	-9.44753200	-2.75482600	2.42314600
H	-9.66693000	-3.99234800	0.27500800
H	2.43239800	-0.67586000	0.26486100
N	-5.07352500	1.89335800	0.96362500
H	-5.91751500	1.45450700	1.30426800
C	-6.30309100	3.90456800	1.55837600
H	-6.12485000	4.37043400	2.53033000
H	-6.55762500	4.70257300	0.85921000
H	-7.15041700	3.22212900	1.64349000
N	0.43900200	2.74388500	-1.11578900
H	-0.53003200	2.88093200	-0.84625400
C	0.33695400	5.11589800	-1.60071600
H	0.74968000	5.86284600	-0.91929300
H	0.45352400	5.50439500	-2.61472800
H	-0.72614400	4.98931900	-1.38928000
C	1.15120300	3.83956300	-1.47527400
O	2.36396700	3.85968600	-1.69463300
C	-5.01902200	3.24519900	1.09696500
O	-4.02592700	3.93318300	0.86213900
C	4.83147000	-1.06013900	0.00206400
C	6.10113800	-1.77363500	-0.47419200
H	4.28840500	-1.74693400	0.67597100
C	5.87371700	-0.10721400	1.92100800
C	6.99618500	-2.01183200	0.75334500
H	5.81862100	-2.74970100	-0.89426800
C	7.22745900	-0.72761300	1.55330700
H	5.31751900	-0.79680600	2.57569700
H	6.50990300	-2.74136900	1.41727700
H	7.78866200	-0.01983200	0.93286900
O	5.15416400	0.11203600	0.71534100
O	8.00897500	-1.11989500	2.69154100
O	8.22439300	-2.55030700	0.27300300
O	6.76826400	-0.99314700	-1.44445900
C	5.92196200	1.27278500	2.57331100
H	6.34929100	1.21237700	3.57774900
H	6.55735000	1.93217100	1.96227100
O	4.62290700	1.80794400	2.71324600
H	4.17258200	1.74954800	1.85156900
H	8.44765900	-0.35012200	3.07001800
H	8.84412600	-2.56906900	1.01429900
H	7.65041200	-1.37475500	-1.55342200

Q1

C	-2.34712200	0.65834300	0.49894800
C	-0.86788000	1.03860100	0.72213300
C	-0.14366500	0.13291000	1.73150700
C	-1.71284700	-1.65927300	1.36877100
C	-2.49294900	-0.86206900	0.31566500
H	-0.37857800	0.92228400	-0.24668100
H	-2.94033200	0.96527400	1.36245400
H	-2.18432500	-1.50481700	2.35160600
H	-0.48536000	0.34652800	2.74808500
O	-0.35211200	-1.23046100	1.41461700
O	-2.75243600	1.35742700	-0.67045100
C	-1.65910700	-3.15962400	1.07457700
H	-2.67462400	-3.55463700	1.02899100
H	-1.14097600	-3.65472600	1.90717300
H	-3.66395100	1.67491900	-0.58119100
O	-1.04181600	-3.45790500	-0.16061000
H	-0.09080300	-3.26509000	-0.09689400
C	2.78660100	1.31710900	0.19365700
C	3.94953300	0.99982000	-0.77882000
C	2.05451000	0.03366200	0.64079800
H	3.19930700	1.79603800	1.09401200
C	4.80187900	-0.14909200	-0.21860100
H	3.51710800	0.66069100	-1.72710300
C	3.03774200	-1.07488100	1.05771500
H	1.44304700	-0.33269400	-0.18596600
H	5.29558900	0.16770900	0.71430200
H	3.54453500	-0.76127000	1.98315400
O	4.01257000	-1.28298900	0.02248500
O	5.76857400	-0.46150900	-1.18483900
O	1.25168400	0.34898500	1.78313700
C	2.41070500	-2.44147600	1.29938500
H	3.18441700	-3.11940700	1.67879800
H	1.61696100	-2.35807400	2.03599500
O	1.81684400	-2.98702100	0.12483400
H	2.49498800	-3.08820300	-0.55461400
O	1.82000800	2.15299500	-0.41169400
H	2.28303200	2.97626300	-0.68133000
O	-3.85528200	-1.31650000	0.46430300
C	6.88783200	-1.19850800	-0.83122400
C	7.03598100	-1.86862000	0.38220400
C	7.89975100	-1.24121900	-1.79178600
C	8.21971400	-2.56570100	0.63297200
H	6.23832500	-1.88060200	1.11283400
C	9.06916400	-1.94661300	-1.53097100
H	7.75353900	-0.72452300	-2.73415400
C	9.23821500	-2.60902700	-0.31413700
H	8.33305900	-3.08797300	1.57741400
H	9.85154600	-1.97744500	-2.28216300
H	10.15097100	-3.15852700	-0.11167900
H	-2.13563000	-1.14081000	-0.68124700
N	4.89377800	2.10359900	-1.03499400
H	5.79619500	1.76726000	-1.34039800
C	5.89482100	4.24211100	-1.61137500
H	5.70921100	4.65252900	-2.60683300
H	6.01084700	5.08612600	-0.92995100
H	6.82459500	3.67105800	-1.63270500
N	-0.69691500	2.44001200	1.07644100
H	0.04270700	2.90261700	0.56928000
C	-0.82842400	4.49038800	2.38026800
H	-1.73209000	5.10383200	2.38436300
H	-0.36232300	4.59371900	3.36254600
H	-0.14620900	4.87450600	1.61929400
C	-1.22574300	3.03781500	2.17547300
O	-1.98447900	2.47057900	2.95688400
C	4.68198100	3.43784600	-1.18621100
O	3.60620600	4.00408100	-0.99651400
C	-4.77139400	-1.00990100	-0.53604200
C	-5.85658400	-2.09745400	-0.52227800
H	-4.28151500	-0.97816000	-1.52119300
C	-6.29871700	0.68918600	-1.20295000
C	-7.03623900	-1.68881700	-1.40654000
H	-5.41805000	-3.02999300	-0.90181900
C	-7.50015800	-0.25315700	-1.12313800
H	-5.86820200	0.64828700	-2.21518300
H	-6.73222700	-1.74052100	-2.45733100
H	-7.91632800	-0.20580700	-0.10822200
O	-5.32833900	0.26303800	-0.24711100
O	-8.47268000	0.15132500	-2.07743700
O	-8.12746200	-2.59518600	-1.27878600
O	-6.37941400	-2.30585100	0.78530200
C	-6.61577700	2.13428700	-0.88157000
H	-7.39520100	2.49496700	-1.55905400
H	-6.97119400	2.21033900	0.15290800
O	-5.39827100	2.86681300	-1.05517000
H	-5.50723900	3.76766300	-0.73234800
H	-9.12577200	-0.55892600	-2.14356200
H	-8.20800300	-2.82348800	-0.34118100
H	-5.64876500	-2.28464200	1.41727900

Q2

C	-2.36537500	0.65590100	0.55497900
C	-0.87960900	1.03876600	0.71401300
C	-0.13900000	0.15741400	1.73415100
C	-1.70392800	-1.64825900	1.41665200
C	-2.50107100	-0.86406200	0.36624900
H	-0.41906200	0.88155100	-0.26382600
H	-2.92137500	0.95301600	1.44588100
H	-2.16925000	-1.48783800	2.40048900
H	-0.47085900	0.39393700	2.74976500
O	-0.34208000	-1.21147700	1.44964500
O	-2.83600000	1.35953600	-0.58929600
C	-1.65315700	-3.14981700	1.13583600
H	-2.67127600	-3.53716100	1.10167700
H	-1.12960100	-3.63960900	1.96826000
H	-3.72850900	1.69964400	-0.42906500
O	-1.04250200	-3.46075100	-0.10204900
H	-0.09213000	-3.26597800	-0.04508300
C	2.78173500	1.31545200	0.15001500
C	3.94977000	0.98201700	-0.81099100
C	2.05358700	0.04115200	0.63007700
H	3.18947000	1.82018700	1.03849600
C	4.80322500	-0.15166500	-0.22226200
H	3.52343400	0.62217700	-1.75435600
C	3.03964000	-1.05675700	1.06851400
H	1.43813100	-0.34421800	-0.18498600
H	5.29072600	0.18684100	0.70643800
H	3.54493900	-0.72402100	1.98804400
O	4.01709600	-1.28229700	0.03834300
O	5.77770200	-0.48006000	-1.17598400
O	1.25694300	0.38356700	1.76863000
C	2.41640100	-2.42048600	1.33502200
H	3.19175800	-3.08849400	1.72879800
H	1.62067800	-2.32553300	2.06804600
O	1.82678200	-2.98929000	0.16993000
H	2.50665800	-3.10139200	-0.50600800
O	1.81184300	2.13041200	-0.47962500
H	2.27266300	2.94026300	-0.79071300
O	-3.84599400	-1.34202700	0.53197300
C	6.89773500	-1.20389800	-0.79949400
C	7.03806000	-1.85362400	0.42597500
C	7.91941500	-1.25598100	-1.74929600
C	8.22334700	-2.53941800	0.69937300
H	6.23319400	-1.85913700	1.14871400
C	9.09026400	-1.95021500	-1.46594300
H	7.77933100	-0.75547500	-2.70129600
C	9.25130600	-2.59197100	-0.23701900
H	8.33039000	-3.04578000	1.65317900
H	9.88009400	-1.98848200	-2.20898500
H	10.16521800	-3.13271700	-0.01689600
H	-2.14310100	-1.13479200	-0.63388900
N	4.89226400	2.08382100	-1.08313500
H	5.80476800	1.74518700	-1.35408200
C	5.88220200	4.20726900	-1.73128900
H	5.71508700	4.57535900	-2.74634200
H	5.97364800	5.07915800	-1.08191200
H	6.81828600	3.64662000	-1.70854500
N	-0.67876000	2.44961400	1.01149500
H	0.08181100	2.86586000	0.49417300
C	-0.75337000	4.56355400	2.21027900
H	-1.63400400	5.20885800	2.17799600
H	-0.29019000	4.69675400	3.19042000
H	-0.05261700	4.88670500	1.43781200
C	-1.20153900	3.11681800	2.07179400
O	-1.99097000	2.61980800	2.87025200
C	4.66856800	3.40654600	-1.30159800
O	3.58101300	3.96581200	-1.16621000
C	-4.77443900	-1.02585700	-0.43816700
C	-5.83300800	-2.12893700	-0.54745000
H	-4.28869800	-0.90399900	-1.41931900
C	-6.29957000	0.69651600	-1.06147400
C	-6.91384000	-1.67349600	-1.53636900
H	-5.34732400	-3.02974300	-0.94736400
C	-7.46827700	-0.29563100	-1.17283300
H	-5.80207200	0.77418700	-2.04145500
H	-6.47651700	-1.60573200	-2.54340600
H	-7.98195400	-0.36348800	-0.20779300
O	-5.38848400	0.21501800	-0.08433100
O	-8.39872700	0.03611800	-2.21427300
O	-7.93701000	-2.66260300	-1.52591800
O	-6.39192600	-2.39133500	0.72514700
C	-6.68810800	2.09367100	-0.61834300
H	-7.46058800	2.50075900	-1.28260100
H	-7.07495200	2.05468200	0.40662000
O	-5.50698700	2.89411500	-0.68772400
H	-5.59846100	3.67076400	-0.12566600
H	-9.06256300	0.65007300	-1.88300600
H	-8.66926400	-2.33371300	-2.06362700
H	-7.14180000	-2.98570900	0.58641700

R1

C	2.37669000	1.27350400	-0.90039500
C	0.87279100	1.58228800	-0.93522700
C	0.13812500	0.81955700	-2.05671700
C	1.87588600	-0.86466800	-2.14366200
C	2.60291800	-0.23982000	-0.94934300
H	0.46569400	1.25535500	0.02265700
H	2.86500400	1.72273000	-1.77096500
H	2.29437600	-0.45971600	-3.07796000
H	0.36655900	1.26978000	-3.02598600
O	0.48332400	-0.55657000	-2.05820400
O	2.88846000	1.85094700	0.29518500
C	1.96687200	-2.38927900	-2.18066200
H	3.01184900	-2.68416800	-2.29226300
H	1.42434000	-2.74683000	-3.06559000
H	3.78953700	1.51769700	0.42332400
O	1.48431600	-2.99998500	-1.00078800
H	0.51618000	-2.90612600	-0.96630200
C	-2.69942200	1.37630100	-0.07387000
C	-3.71528000	0.75301200	0.91443300
C	-1.92323700	0.28546900	-0.84062700
H	-3.24512700	1.98002200	-0.81387000
C	-4.53818100	-0.33616300	0.20831000
H	-3.15054300	0.27044500	1.72044700
C	-2.85788000	-0.80219500	-1.39738500
H	-1.20486500	-0.18314400	-0.16533200
H	-5.16377500	0.11449600	-0.57880300
H	-3.48038000	-0.35548900	-2.18758500
O	-3.69932100	-1.31294800	-0.35050400
O	-5.35118700	-0.93630700	1.17916000
O	-1.26606800	0.89920400	-1.95541300
C	-2.15788000	-2.02400100	-1.97890100
H	-2.91361600	-2.67989600	-2.42638700
H	-1.45329200	-1.71664700	-2.74617400
O	-1.40673100	-2.74190900	-1.00266000
H	-2.00354500	-3.05054600	-0.30951700
O	-1.74355100	2.16981900	0.60092800
H	-2.24295200	2.88526100	1.05271200
O	4.01742400	-0.50816600	-1.09325100
C	-6.44263700	-1.70031400	0.79642300
C	-6.69764000	-2.10665200	-0.51227300
C	-7.30792300	-2.05935400	1.83115200
C	-7.84264300	-2.86048700	-0.77837900
H	-6.00850800	-1.87270400	-1.31268900
C	-8.43930100	-2.81754300	1.55184400
H	-7.07877900	-1.74360700	2.84314900
C	-8.71620800	-3.21823100	0.24390200
H	-8.03935300	-3.17736400	-1.79740800
H	-9.10754100	-3.09529200	2.36034100
H	-9.59944500	-3.80910500	0.02755200
H	2.24142200	-0.70628400	-0.02921300
N	-4.70285100	1.68068000	1.49658700
H	-5.51961900	1.19598100	1.84102900
C	-5.80863100	3.56432200	2.56282500
H	-5.51538700	3.85583100	3.57396900
H	-6.11752900	4.47183100	2.04168400
H	-6.65843500	2.88229800	2.62351500
N	0.58436800	3.00595900	-1.01284800
H	-0.12923500	3.31759800	-0.37160000
C	0.53030200	5.27157900	-1.90224400
H	1.40485200	5.91013500	-1.75683000
H	0.06460400	5.56666100	-2.84437400
H	-0.17008600	5.44863400	-1.08362700
C	0.99601200	3.83060800	-2.01286900
O	1.71042700	3.45981200	-2.93955200
C	-4.59795300	2.98758200	1.85592400
O	-3.61825100	3.69978600	1.63856400
C	4.65413300	-1.09788300	-0.00181700
C	6.04761600	-1.54561700	-0.45934400
H	4.08124000	-1.96242000	0.36725000
C	5.39172000	-0.59090800	2.22417000
C	6.89027100	-1.96081700	0.74986700
H	5.93381500	-2.40148100	-1.13809000
C	6.84430300	-0.91580200	1.87462700
H	4.88362900	-1.49750500	2.58239600
H	6.50107100	-2.90196900	1.15154400
H	7.33674300	0.00281700	1.52789000
O	4.74058800	-0.12515500	1.03045000
O	7.48807800	-1.41508700	3.03782700
O	8.23729400	-2.23199500	0.37835500
O	6.75711700	-0.49852700	-1.10926300
C	5.22063400	0.50624700	3.26471600
H	5.71133600	0.20277300	4.18895900
H	5.70299200	1.42609600	2.90573500
O	3.85665000	0.73284000	3.58056500
H	3.39353300	1.04343900	2.79048800
H	8.34525300	-1.77133400	2.76680600
H	8.51024000	-1.55018000	-0.25269600
H	6.17781200	-0.08502700	-1.76241700

R2

C	2.40788600	1.35563400	-0.95095900
C	0.90076700	1.64482100	-0.93522600
C	0.15417100	0.91160000	-2.06904500
C	1.90845800	-0.75276700	-2.23341900
C	2.64254400	-0.15651000	-1.02887900
H	0.52018100	1.27403400	0.01790300
H	2.86945800	1.82306900	-1.82602600
H	2.31438400	-0.31736200	-3.15886000
H	0.35920300	1.39937500	-3.02522300
O	0.51164900	-0.45934500	-2.12580300
O	2.95267200	1.92068500	0.23850600
C	2.01361300	-2.27379500	-2.31679700
H	3.06112200	-2.55187900	-2.44379600
H	1.46841800	-2.61160800	-3.20790600
H	3.88017200	1.64658300	0.28735100
O	1.54557700	-2.92698900	-1.15213900
H	0.57766600	-2.83970500	-1.10595800
C	-2.65193800	1.36401400	-0.01421200
C	-3.65996900	0.69663600	0.95375100
C	-1.88242400	0.31091400	-0.83964800
H	-3.20462000	2.00192300	-0.71960300
C	-4.48611800	-0.36088900	0.20579900
H	-3.08886500	0.17947200	1.73335500
C	-2.81796400	-0.75946000	-1.42804800
H	-1.14906200	-0.17926300	-0.19633700
H	-5.11389100	0.12264900	-0.55982100
H	-3.44881200	-0.28552100	-2.19539800
O	-3.64993800	-1.31356700	-0.39505200
O	-5.29791900	-1.00016100	1.15349800
O	-1.24992500	0.97427300	-1.93946400
C	-2.11689900	-1.95419500	-2.06140700
H	-2.87290000	-2.59710900	-2.52718400
H	-1.42042500	-1.61383800	-2.82223900
O	-1.35313800	-2.70419400	-1.11979100
H	-1.94162900	-3.04115700	-0.43281600
O	-1.68899800	2.12447700	0.68810500
H	-2.18214500	2.81616900	1.18112900
O	4.05216500	-0.41724700	-1.18324600
C	-6.39151600	-1.74510600	0.74201500
C	-6.64680400	-2.10214500	-0.58103400
C	-7.25941500	-2.13895300	1.76189400
C	-7.79421400	-2.84155500	-0.87566900
H	-5.95574100	-1.84137700	-1.37140100
C	-8.39322700	-2.88227700	1.45394300

H	-7.03021900	-1.86144700	2.78504700
C	-8.67023500	-3.23356200	0.13187800
H	-7.99091300	-3.12000900	-1.90587900
H	-9.06330700	-3.18715500	2.25109400
H	-9.55537500	-3.81283100	-0.10699200
H	2.27648600	-0.63883100	-0.11815000
N	-4.64389500	1.59725800	1.58291900
H	-5.46139000	1.09857400	1.90480200
C	-5.73953000	3.42755600	2.74783100
H	-5.44275800	3.66771300	3.77136600
H	-6.04719000	4.36064000	2.27315600
H	-6.59123700	2.74577400	2.77691200
N	0.58548800	3.06489600	-0.94889200
H	-0.11168900	3.33869400	-0.27352300
C	0.45909600	5.36254900	-1.74218600
H	1.32685900	6.01267800	-1.60802500
H	-0.05113300	5.68400600	-2.65213200
H	-0.20976900	5.49368200	-0.88909700
C	0.94735700	3.93619700	-1.92841400
O	1.63407200	3.61775900	-2.89370000
C	-4.53230200	2.88313800	2.00959000
O	-3.55026000	3.60172800	1.82739300
C	4.66230600	-1.04362200	-0.10882300
C	6.07648400	-1.49748100	-0.47233300
H	4.07857000	-1.92107800	0.21408500
C	5.20550500	-0.66093900	2.19616200
C	6.73890600	-2.05908400	0.79497500
H	6.00363200	-2.30206000	-1.21717700
C	6.66646800	-1.07132200	1.96176300
H	4.61502600	-1.54542800	2.47716700
H	6.21908000	-2.97982100	1.09703700
H	7.25447400	-0.18197800	1.70875300
O	4.70338500	-0.11584000	0.97550200
O	7.24814700	-1.75342200	3.08091600
O	8.08769500	-2.35946600	0.45587600
O	6.81346800	-0.40761500	-0.98778700
C	4.99182000	0.42639000	3.24334700
H	5.31898100	0.07208000	4.22353300
H	5.59278000	1.30819200	2.97562000
O	3.62567700	0.76385400	3.38391000
H	3.28556700	1.08080800	2.53317900
H	7.56183800	-1.11583700	3.73136600
H	8.54974900	-2.59961400	1.26987100
H	7.73666800	-0.69046000	-1.04226900

S1

C	-2.51425800	0.42301100	-1.08182300
C	-1.29347600	1.04144100	-1.78802700
C	0.01592100	0.39713200	-1.31851800
C	-1.06910800	-1.67178600	-0.81383100
C	-2.46537200	-1.11529700	-1.14105000
H	-1.37572700	0.80905600	-2.85412400
H	-2.51492000	0.72070300	-0.03613900
H	-0.88289800	-1.52462900	0.26027700
H	0.16957100	0.55439800	-0.24195300
O	-0.05489500	-1.00217400	-1.57210200
O	-3.67893400	0.90580500	-1.73519900
C	-0.92229200	-3.15571200	-1.13736700
H	-1.67745300	-3.72571400	-0.59711900
H	0.06807200	-3.49159300	-0.80368400
H	-4.20565700	1.43163300	-1.10690600
O	-1.11613700	-3.43739500	-2.51415200
H	-0.45950600	-2.94399500	-3.02176100
C	2.56992400	1.66715700	-0.29229400
C	4.02009500	1.61686800	0.20956900
C	2.41221800	0.89823000	-1.60619100
H	1.93690700	1.22581600	0.48336300
C	4.57980300	0.18303200	0.16870900
H	4.63270400	2.20858100	-0.47509700
C	3.00457300	-0.51538300	-1.51265000
H	2.95492000	1.43060300	-2.38971700
H	4.10856900	-0.44532300	0.93147700
H	2.46015300	-1.12162400	-0.77697400
O	4.37833900	-0.38539900	-1.11877800
O	5.95601500	0.27998100	0.38417200
O	1.04829300	0.94181900	-2.07943600
C	3.03142900	-1.25368500	-2.84902600
H	3.39205100	-2.27620300	-2.67737700
H	2.02425300	-1.30339600	-3.25885600
O	3.83227300	-0.59096200	-3.81334500
H	4.72930100	-0.52391200	-3.46078400
O	2.23468600	3.04178700	-0.46399400
H	1.33730500	3.08750700	-0.81950300
O	-3.31426500	-1.75388800	-0.16236500
C	6.66495500	-0.82531600	0.80915100
C	6.19187200	-2.13579000	0.74410700
C	7.93993800	-0.56101000	1.31376600
C	6.99882900	-3.17579500	1.20919400
H	5.22210600	-2.35393300	0.31659300
C	8.73674200	-1.60782000	1.76311800

H	8.28673100	0.46588800	1.34832300
C	8.26900500	-2.92224700	1.71834900
H	6.62707400	-4.19424800	1.15857500
H	9.72633500	-1.39452200	2.15439600
H	8.89047800	-3.73788800	2.07178200
H	-2.77149000	-1.44371300	-2.14008000
N	4.16381100	2.22159300	1.52883400
H	4.68467200	3.08082000	1.58243200
C	3.76553900	2.59101500	3.90994700
H	2.80200300	2.97243700	4.25502100
H	4.15684500	1.93833600	4.69242300
H	4.44898000	3.43187000	3.77515600
N	-1.24859900	2.50670500	-1.70304200
H	-0.96077200	2.96093200	-2.55530800
C	-1.73262300	4.80395100	-1.05924700
H	-2.76516900	5.13375200	-1.20100500
H	-1.32812600	5.35301700	-0.20803800
H	-1.16252700	5.05889800	-1.95501500
C	-1.74528700	3.32237900	-0.72957000
O	-2.18158600	2.92114800	0.34695100
C	3.54161000	1.76888200	2.65563600
O	2.84580500	0.76021200	2.66374900
C	-4.65031300	-1.35792400	-0.07829000
C	-5.43317900	-2.44719600	0.66585600
H	-5.07644900	-1.20299100	-1.08000000
C	-6.00774700	0.40418100	0.73587300
C	-6.84317500	-1.93850900	0.99363000
H	-5.50436400	-3.33647700	0.02528400
C	-6.83818800	-0.52995700	1.61506300
H	-6.45955100	0.45095600	-0.26759500
H	-7.42621800	-1.88787900	0.06801800
H	-6.37522600	-0.57968300	2.60973500
O	-4.69464500	-0.14347800	0.64542300
O	-8.16348300	-0.02728900	1.70806700
O	-7.54358700	-2.84797600	1.83536400
O	-4.82497100	-2.79109700	1.90590600
C	-5.84093000	1.82866800	1.24143700
H	-6.81865000	2.30628100	1.32202900
H	-5.37203000	1.82243600	2.23037500
O	-5.04547300	2.56847000	0.30798500
H	-4.16754900	2.75936300	0.67241300
H	-8.70767800	-0.71240100	2.11992000
H	-6.92273100	-3.16174300	2.50892200
H	-3.86933800	-2.85113900	1.78089700

S2

C	-2.50744700	0.41443800	-1.06381500
C	-1.28673400	1.01780400	-1.78069000
C	0.02183300	0.36963400	-1.31240100
C	-1.07261200	-1.68112500	-0.77373300
C	-2.46840100	-1.12430900	-1.10322800
H	-1.37618000	0.77588900	-2.84420800
H	-2.50607100	0.72331300	-0.02152500
H	-0.88357900	-1.51504600	0.29625800
H	0.18388400	0.54089100	-0.23888900
O	-0.05631000	-1.02886700	-1.54936000
O	-3.67244100	0.89576600	-1.72249400
C	-0.94002100	-3.17180200	-1.06419300
H	-1.69998400	-3.71676500	-0.50617500
H	0.04826200	-3.51020500	-0.72674100
H	-4.19147100	1.43604400	-1.10286700
O	-1.13941300	-3.48299500	-2.43585500
H	-0.48137500	-3.00389000	-2.95498600
C	2.59240300	1.65644200	-0.32740200
C	4.04884200	1.61272100	0.15623200
C	2.41821200	0.85789000	-1.62187600
H	1.96704000	1.23509100	0.46560700
C	4.59283700	0.17226400	0.15546200
H	4.65691600	2.17419000	-0.55744400
C	2.99939200	-0.55811800	-1.49709800
H	2.96119900	1.36642300	-2.42103700
H	4.11946100	-0.42753700	0.93956600
H	2.45399600	-1.14203700	-0.74458200
O	4.37693200	-0.43046000	-1.11360300
O	5.97172900	0.25931900	0.35969700
O	1.05245900	0.90405100	-2.08669500
C	3.01409500	-1.32836100	-2.81546400
H	3.36604500	-2.34969000	-2.62071500
H	2.00500100	-1.37856500	-3.22006000
O	3.81817300	-0.69609200	-3.79800700
H	4.71621400	-0.62761500	-3.44833000
O	2.25809300	3.02775900	-0.52715800
H	1.35593100	3.06527100	-0.87127600
O	-3.29795600	-1.74836000	-0.10843800
C	6.67026500	-0.84197100	0.81080800
C	6.18129600	-2.14802900	0.78560100
C	7.95163700	-0.57918900	1.29996100
C	6.97888600	-3.18426200	1.27451600
H	5.20586600	-2.36644600	0.37138200
C	8.73889100	-1.62256400	1.77352600
H	8.31069200	0.44408000	1.30388400
C	8.25537700	-2.93203400	1.76835000
H	6.59435600	-4.19902700	1.25507200
H	9.73343600	-1.41031200	2.15272600
H	8.86937200	-3.74494200	2.14070400
H	-2.78012700	-1.46189900	-2.09841500
N	4.21770500	2.26277800	1.45054100
H	4.74239700	3.12118500	1.46435800
C	3.86017400	2.72092700	3.82283800
H	2.90340600	3.11285900	4.17476300
H	4.26913600	2.09629500	4.61912100
H	4.53919800	3.55734200	3.64454500
N	-1.22728900	2.48449100	-1.71056100
H	-0.90334100	2.92280300	-2.55825800
C	-1.67115000	4.79979900	-1.10845900
H	-2.69210300	5.16143100	-1.25531600
H	-1.25034700	5.34468800	-0.26224200
H	-1.09114300	5.02701400	-2.00532400
C	-1.73182600	3.32257800	-0.76224300
O	-2.20898300	2.94896500	0.30752500
C	3.61110200	1.85499600	2.60285700
O	2.91066300	0.85161500	2.65921300
C	-4.62610300	-1.36934100	-0.01579200
C	-5.43684200	-2.43603400	0.72705000
H	-5.06215400	-1.21985700	-1.01557900
C	-5.99226400	0.42442800	0.73059100
C	-6.86216700	-1.90037000	0.93927300
H	-5.49099200	-3.33417600	0.09581900
C	-6.87072000	-0.50797700	1.57715000
H	-6.41237900	0.47860200	-0.28675100
H	-7.36555300	-1.82333400	-0.03571900
H	-6.45843800	-0.57601600	2.58985400
O	-4.68694300	-0.13278600	0.69019100
O	-8.24976300	-0.11461900	1.62206400
O	-7.54277500	-2.84818500	1.75371500
O	-4.82064200	-2.73726400	1.96312200
C	-5.82405400	1.84867700	1.24130000
H	-6.80015400	2.33624800	1.32311800
H	-5.35267600	1.83681300	2.22986300
O	-5.04040000	2.59765400	0.31339000
H	-4.14911800	2.77049800	0.65692000
H	-8.38297300	0.54883400	2.30756000
H	-8.40400200	-2.47538600	1.98359000
H	-5.44932300	-3.27064400	2.46868200

T1

C	2.26580400	1.24572200	-0.84567100
C	0.75817100	1.54385700	-0.90571800
C	0.03079000	0.73822600	-1.99963300
C	1.77051400	-0.93738900	-2.03058000
C	2.51716000	-0.26401900	-0.87464000
H	0.34870300	1.25245100	0.06249300
H	2.76052300	1.68812100	-1.71644800
H	2.17236900	-0.56527600	-2.98570700
H	0.26667400	1.14479200	-2.98620000
O	0.37850300	-0.63571400	-1.93672800
O	2.74347000	1.84083600	0.35118700
C	1.86965000	-2.46209200	-2.01132200
H	2.91454200	-2.75735300	-2.12291500
H	1.31958600	-2.85525200	-2.87632300
H	3.63393200	1.50403400	0.53667100
O	1.40200900	-3.03019900	-0.80439100
H	0.43459700	-2.93333900	-0.76024400
C	-2.83456200	1.37774800	-0.07813800
C	-3.86670200	0.80239300	0.92185100
C	-2.04032700	0.25577300	-0.77843800
H	-3.36778000	1.94235600	-0.85696800
C	-4.66647200	-0.33349800	0.26468100
H	-3.31563700	0.37311100	1.76645000
C	-2.95961900	-0.86689800	-1.28933900
H	-1.32645600	-0.17218800	-0.07226600
H	-5.28392200	0.06474300	-0.55643700
H	-3.57805400	-0.46705500	-2.10744700
O	-3.80784900	-1.32991500	-0.22557900
O	-5.48974600	-0.88760600	1.25425200
O	-1.37454900	0.82010200	-1.91359900
C	-2.24500400	-2.11094300	-1.80177000
H	-2.99196800	-2.79533800	-2.22050600
H	-1.53725800	-1.83661100	-2.57841400
O	-1.49529700	-2.77133000	-0.78465700
H	-2.09505700	-3.04731300	-0.08040300
O	-1.89438100	2.20740100	0.57510700
H	-2.40449900	2.94757300	0.97067600
O	3.93019100	-0.50754100	-1.07454900
C	-6.56697800	-1.68304700	0.89622500
C	-6.79155200	-2.16900300	-0.39075800
C	-7.45058800	-1.98811700	1.93283100
C	-7.92463100	-2.94790800	-0.63453600
H	-6.08803800	-1.97649600	-1.18965000
C	-8.56977000	-2.77209300	1.67641400
H	-7.24517000	-1.61056400	2.92862900
C	-8.81618000	-3.25253300	0.38942600
H	-8.09779300	-3.32627400	-1.63673100
H	-9.25249900	-3.00725600	2.48631900
H	-9.68998400	-3.86335500	0.19089800
H	2.20396900	-0.71879400	0.06927000
N	-4.87319100	1.75433200	1.42742900
H	-5.69501100	1.28506100	1.78100600
C	-6.01645400	3.69044700	2.35043700
H	-5.74966500	4.04593000	3.34833300
H	-6.31910600	4.56165400	1.76729700
H	-6.86286100	3.00645600	2.43287800
N	0.46845100	2.96353300	-1.03827400
H	-0.24116200	3.30155400	-0.40630800
C	0.41029200	5.19279600	-2.01478600
H	1.28556100	5.83586600	-1.89578800
H	-0.05751400	5.45196500	-2.96638300
H	-0.28803500	5.40155400	-1.20190000
C	0.87503800	3.74824400	-2.07149800
O	1.58450500	3.34202300	-2.98733200
C	-4.78519400	3.08147900	1.70894200
O	-3.80491300	3.78658200	1.47250900
C	4.65910100	-1.02707800	-0.01066500
C	5.97163200	-1.57229400	-0.59383400
H	4.10606500	-1.83600300	0.49093900
C	5.68806600	-0.37736300	2.05232600
C	6.95931600	-1.91383600	0.52218400
H	5.73773300	-2.47765200	-1.16978500
C	7.07203200	-0.76107900	1.52105200
H	5.22533300	-1.24296800	2.54801400
H	6.61117500	-2.80245600	1.05831600
H	7.49638900	0.11399300	1.01264000
O	4.88808500	0.02285500	0.92942800
O	7.89936300	-1.13189600	2.62289900
O	8.24180700	-2.25289600	0.00564100
O	6.61845000	-0.61912500	-1.42688000
C	5.72101900	0.78288900	3.04972500
H	6.29940600	1.61605000	2.62587600
H	4.70213600	1.13446600	3.22103800
O	6.23123800	0.38324200	4.30859000
H	7.08722600	-0.04657800	4.16643300
H	8.71068200	-1.52165600	2.26948500
H	8.43073100	-1.64966000	-0.72814500
H	5.95893100	-0.20420100	-1.99856000

Table S3. Cartesian coordinates of optimized configurations for Manβ(1,3) Manβ(1,6)Man

A1

C	2.60356000	-3.57830300	0.78615300
C	1.17315800	-4.10005500	0.63405000
C	0.29184000	-3.06369100	-0.07677600
C	2.17636500	-1.97851200	-1.07540000
C	3.16025600	-3.01901700	-0.52721600
H	2.60383900	-2.76951500	1.53088800
H	2.08760000	-1.17282300	-0.33989700
H	3.27790300	-3.83433100	-1.24573000
O	0.88228400	-2.58292500	-1.26259800
C	-1.16469900	-1.25938900	0.68220300
C	-1.44695600	-0.68637800	-0.71460300
C	-1.03542100	-0.12271700	1.69155000
C	-2.77331500	0.08149000	-0.62695500
H	-1.52153800	-1.47624500	-1.46025400
C	-2.04947900	1.03359300	1.52010100
H	-3.04267400	0.49233800	-1.60295500
H	-2.86671600	0.86020900	2.22680500
O	-2.60177100	1.20808400	0.19831200
C	3.59466700	1.12866400	1.44831700
C	2.44965000	1.82491800	0.70669800
C	2.95018800	2.68579900	-0.46578200
C	0.71367300	3.42326700	1.20856100
C	1.80506000	3.49403300	-1.06963700
H	3.70420800	3.38269600	-0.09000700
C	1.08867400	4.30647100	0.01117600
H	0.44464300	4.06526700	2.04999800
H	1.08455300	2.81152100	-1.54191900
O	1.78673100	2.64057400	1.68353200
O	3.61124600	1.90915400	-1.46846000
H	2.98348400	1.27279100	-1.88665300
O	2.28435800	4.42339600	-2.03715800
H	2.94353300	3.97492000	-2.58450500
O	1.92383700	5.34134000	0.50255100
H	2.32524600	5.77132300	-0.26581300
O	-1.19745900	-0.57342800	3.03324300
H	-0.48145300	-1.18967600	3.23226500
O	-0.44398600	0.21593800	-1.18250500
H	-0.54286300	1.07302500	-0.73288500
O	1.14014200	-5.28099600	-0.14892500
H	1.87328600	-5.83820500	0.14864800
O	3.39598300	-4.66426800	1.25862300
H	4.32454100	-4.42286400	1.13454200
O	4.46589500	-2.49500100	-0.31237000
H	4.41058400	-1.64900600	0.17233200
H	-0.64036600	-3.54160400	-0.39027200
C	2.57528600	-1.35520700	-2.40296500
H	3.65635400	-1.22322900	-2.44534300
C	-1.37207500	2.36013200	1.83778700
O	1.99842300	-0.05321400	-2.57734600
H	1.06736600	-0.06819900	-2.28806200
H	2.27370800	-2.01358900	-3.22423000
H	3.19651900	0.55804400	2.28853300
H	4.28169100	1.88291700	1.84951000
O	4.29435900	0.20750700	0.61011600
H	4.50634700	0.66717700	-0.22072400
H	-0.90152200	2.29257300	2.82306900
H	-2.10511700	3.17298200	1.84021200
O	-0.38962100	2.61841300	0.82216000
H	0.74896300	-4.29173200	1.62816400
H	-0.02580700	0.27929200	1.56838000
H	-2.00975500	-1.89952400	0.95338400
O	-3.76924900	-0.81086700	-0.17925800
C	-5.10173600	-0.49127600	-0.37392100
C	-5.56276200	0.79569000	-0.65276300
C	-5.99805200	-1.55535700	-0.25783800
C	-6.93216900	1.00118400	-0.83466200
H	-4.87717000	1.63157300	-0.70309500
C	-7.35921600	-1.33227800	-0.43150000
H	-5.61332800	-2.54500200	-0.03778100
C	-7.83409600	-0.05286000	-0.72537100
H	-7.28985400	2.00232800	-1.05286300
H	-8.05113000	-2.16344200	-0.34194900
H	-8.89612500	0.11880300	-0.86325100
H	0.16380700	4.72615800	-0.40303200
O	0.04827700	-2.00904200	0.83515300
H	1.75724900	1.06452100	0.32666000

A2

C	-2.41630900	3.79123600	0.78856900
C	-1.01808200	4.29288600	0.41295000
C	-0.19231300	3.15264100	-0.19389800
C	-2.14977500	2.00118900	-0.97081000
C	-3.08904400	3.05368600	-0.37735800
H	-2.33279800	3.09068000	1.62333800
H	-1.96380100	1.23669900	-0.21257300
H	-3.36893300	3.77798800	-1.15607800
O	-0.89014500	2.62555900	-1.31534400
C	1.20904200	1.36319600	0.69311100
C	1.44263900	0.67990100	-0.66075300
C	1.03032100	0.31331800	1.78479900
C	2.73621300	-0.13741500	-0.53144100
H	1.55523200	1.41217600	-1.45988600
C	1.97460700	-0.91078300	1.68187700
H	2.96796000	-0.63370900	-1.47669100
H	2.78494400	-0.75838400	2.40053900
O	2.54913000	-1.18091800	0.38549600
C	-3.98706000	-1.34206700	1.11014800
C	-2.69857500	-1.91628800	0.50007100
C	-2.92490800	-2.90999800	-0.65281800
C	-0.85960300	-3.27109800	1.34370100
C	-1.61497100	-3.61695900	-1.01719500
H	-3.64251000	-3.66450600	-0.31829700
C	-0.97163700	-4.27944300	0.19519700
H	-0.62833900	-3.80403300	2.26854000
H	-0.91561200	-2.87815800	-1.43381000
O	-2.04695800	-2.57961400	1.60781000
O	-3.47880700	-2.34356700	-1.82904100
H	-3.02082200	-1.51090000	-2.05507700
O	-1.85645600	-4.63578000	-1.98260500
H	-2.49657900	-4.27459600	-2.61378400
O	-1.74874500	-5.37140100	0.65809900
H	-2.04259400	-5.85801100	-0.12538600
O	1.24298400	0.85011000	3.08758500
H	0.52332500	1.46077200	3.28838100
O	0.38650100	-0.18592400	-1.05976200
H	0.38479500	-0.99416000	-0.51438300
O	-1.11058500	5.38174400	-0.50809300
H	-1.22890700	5.02776600	-1.40101300
O	-3.24810100	4.84979900	1.24703900
H	-3.06466600	5.62685300	0.69819100
O	-4.26139600	2.38319100	0.08302000
H	-4.80376400	3.01811400	0.57054400
H	0.74690100	3.54347600	-0.59748200
C	-2.68126800	1.32526400	-2.22613800
H	-3.74665300	1.12549600	-2.10955000
C	1.20298000	-2.17688800	2.03180500
O	-2.05595600	0.06989200	-2.47612800
H	-1.11965600	0.09924700	-2.21157000
H	-2.54423000	1.99690300	-3.08321700
H	-3.71685000	-0.83568600	2.04500600
H	-4.65020300	-2.17170700	1.37002200
O	-4.69984000	-0.48902300	0.23573000
H	-4.45897000	0.44024600	0.36190700
H	0.74307700	-2.06008800	3.01905000
H	1.87900300	-3.03837300	2.04940000
O	0.20320800	-2.36438400	1.02593500
H	-0.51758500	4.69374400	1.29593000
H	-0.00171700	-0.04011800	1.70347000
H	2.08224300	1.98320800	0.91412500
O	3.77499100	0.75614200	-0.18310200
C	5.08950500	0.37807100	-0.39298900
C	5.50168200	-0.94259300	-0.57391000
C	6.02130100	1.41780500	-0.40067500
C	6.85665800	-1.20789900	-0.78361000
H	4.79032000	-1.75701400	-0.52853500
C	7.36799200	1.13609300	-0.60047200
H	5.67533500	2.43513200	-0.25450100
C	7.79330500	-0.17874000	-0.79761400
H	7.17579500	-2.23536700	-0.92579800
H	8.08725800	1.94866200	-0.60742600
H	8.84386200	-0.39659900	-0.95636700
H	0.04106400	-4.61726500	-0.06125000
O	0.02933200	2.18561600	0.79428500
H	-2.06537300	-1.09073000	0.15156000

A3

C	-2.98489400	-3.34976200	-0.87763300
C	-1.62248900	-4.03894900	-0.77964500
C	-0.62579300	-3.14390800	-0.03052900
C	-2.36766900	-1.95290200	1.09977900
C	-3.46496300	-2.82504900	0.47855700
H	-2.89502600	-2.49554400	-1.56352500
H	-2.20374400	-1.09292000	0.44237800
H	-3.68625100	-3.67144100	1.13374800
O	-1.14137700	-2.69587400	1.20534000
C	0.95192400	-1.40171800	-0.72149200
C	1.30145600	-0.93077600	0.69909200
C	0.85236600	-0.19281800	-1.64199400
C	2.65020400	-0.19738800	0.61139300
H	1.42148100	-1.78396300	1.37155700
C	2.02860000	0.79135600	-1.52819600
H	2.92334300	0.19076200	1.59456200
H	2.80658800	0.45376600	-2.22391300
O	2.58623300	0.92875400	-0.21663200
C	-3.37242200	1.50693400	-1.20034200
C	-2.11427900	2.13718800	-0.59795500
C	-2.40898900	3.04838600	0.59736200
C	-0.30501100	3.58191100	-1.32218000
C	-1.15983500	3.81327200	1.03304300
H	-3.18990300	3.76736400	0.31139600
C	-0.54656600	4.55503200	-0.16071600
H	-0.04504900	4.13216500	-2.23213900
H	-0.43140800	3.10544600	1.43471300
O	-1.53556000	2.92477800	-1.65652000
O	-2.91700700	2.25320700	1.68767900
H	-2.97230900	2.83808600	2.45833400
O	-1.49638400	4.70376200	2.09336900
H	-1.81722700	5.52625600	1.69375500
O	-1.39506900	5.63577100	-0.56098200
H	-2.06731800	5.30156800	-1.17063800
O	0.79850200	-0.57044000	-3.01740200
H	0.04549800	-1.16414800	-3.12909700
O	0.36616900	-0.00174500	1.20884200
H	-0.23037500	-0.40870700	1.86293400
O	-1.71516000	-5.25993700	-0.06570900
H	-2.49604000	-5.72417800	-0.39844100
O	-3.90002100	-4.30211200	-1.41425000
H	-4.79403300	-3.96424100	-1.26594300
O	-4.69733100	-2.12427200	0.31597900
H	-4.52577200	-1.25000800	-0.07887500
H	0.26592100	-3.72337500	0.22369500
C	-2.69488800	-1.43078900	2.49112400
H	-3.74586800	-1.13360400	2.53879800
C	1.58452300	2.19169700	-1.93815200
O	-1.84168700	-0.34094700	2.85676200
H	-2.18148000	0.48232900	2.47017600
H	-2.52581900	-2.21767300	3.22817200
H	-3.09927700	0.91176900	-2.07216500
H	-4.05625300	2.29750800	-1.53431800
O	-4.03393000	0.62613500	-0.29144400
H	-4.14578600	1.07978400	0.55729400
H	1.09016900	2.13741100	-2.91238600
H	2.45104000	2.85723400	-2.00662200
O	0.69405400	2.70064900	-0.93477000
H	-1.23360000	-4.21811400	-1.79032500
H	-0.06774900	0.32563000	-1.36906100
H	1.74083600	-2.07703000	-1.06872900
O	3.61346800	-1.15199700	0.17913400
C	4.95400500	-0.89324600	0.36874800
C	5.47460700	0.36315300	0.68640600
C	5.80789900	-1.98849900	0.21136600
C	6.85252100	0.50441200	0.86395300
H	4.82613400	1.22519600	0.76969200
C	7.17824700	-1.82924200	0.38176700
H	5.38048700	-2.95368200	-0.03738000
C	7.70971700	-0.58155600	0.71335500
H	7.25311300	1.48221600	1.11246200
H	7.83349700	-2.68588600	0.25987500
H	8.77900400	-0.45923900	0.84873000
H	0.39820200	5.01180600	0.13620000
O	-0.31738900	-2.06107000	-0.88171500
H	-1.41471900	1.35971400	-0.27904100

A4

C	3.08270100	-3.38054900	0.92468900
C	1.75342900	-4.09592400	0.66246400
C	0.76701400	-3.14505900	-0.02567500
C	2.52790800	-1.83753800	-0.99231900
C	3.61883900	-2.68637600	-0.33533700
H	2.93053000	-2.61475600	1.68946600
H	2.25322000	-1.03453600	-0.30875100
H	3.97631000	-3.44442200	-1.04701800
O	1.36326600	-2.66522200	-1.22593400
C	-0.83748700	-1.45971200	0.71405800
C	-1.19373800	-0.96969800	-0.69850000
C	-0.76512000	-0.26429000	1.65469300
C	-2.57452300	-0.29799300	-0.59428400
H	-1.27858300	-1.80896300	-1.39502600
C	-1.99348900	0.65868900	1.57260300
H	-2.86180200	0.10555900	-1.56708100
H	-2.74904600	0.25223600	2.25660100
O	-2.56163600	0.80305900	0.26600500
C	3.28961900	1.64823200	0.86182400
C	1.96243200	2.25292600	0.40015200
C	2.08208900	3.25037500	-0.76170200
C	0.19158600	3.56379000	1.43476800
C	0.78091400	4.04381100	-0.94445600
H	2.90516600	3.94907400	-0.54810100
C	0.30783300	4.66109300	0.37210500
H	-0.00598300	3.99927400	2.41950400
H	0.01270300	3.36279400	-1.31605700
O	1.45726600	2.92382200	1.58036300
O	2.37734100	2.55209300	-1.97273800
H	2.19818200	3.16231800	-2.70219100
O	0.96018400	5.03253000	-1.95669300
H	1.38449200	5.79908100	-1.54374400
O	1.20157700	5.69397500	0.79849000
H	1.94987000	5.28642300	1.25663200
O	-0.68327000	-0.66536000	3.02238000
H	0.13357200	-1.16546100	3.13830800
O	-0.30339600	0.01355300	-1.17419300
H	0.35928100	-0.33652000	-1.80104900
O	1.96886900	-5.25533800	-0.14626300
H	2.00077200	-4.98334100	-1.07468700
O	4.06231200	-4.27003900	1.45003000
H	3.95974200	-5.11859900	0.99358200
O	4.71184900	-1.83070300	0.01763100
H	5.33649300	-2.35267300	0.53976700
H	-0.12300100	-3.69499800	-0.34666400
C	2.91488500	-1.21062200	-2.32546400
H	3.92303500	-0.79888300	-2.25533600
C	-1.63663300	2.07101300	2.03058000
O	1.98413200	-0.20435600	-2.70580000
H	2.27258900	0.66712300	-2.37303200
H	2.90254800	-1.97575200	-3.10721900
H	3.08540900	1.02030100	1.73711600
H	3.95949100	2.45647900	1.18624900
O	3.87843700	0.90400600	-0.19093200
H	4.33741500	0.11917300	0.14422500
H	-1.11601000	2.01084900	2.99095200
H	-2.54979500	2.66270100	2.15360200
O	-0.82046700	2.69262300	1.03399100
H	1.33622500	-4.46220600	1.60200200
H	0.12609500	0.30321100	1.38208600
H	-1.60899400	-2.15853900	1.05145100
O	-3.49508800	-1.30975700	-0.19570100
C	-4.84566700	-1.10579500	-0.38216800
C	-5.42222000	0.13697200	-0.65330200
C	-5.64906400	-2.24407300	-0.27110600
C	-6.80465700	0.22181400	-0.83122100
H	-4.81394900	1.03051700	-0.70002000
C	-7.02492800	-2.14113000	-0.44109500
H	-5.17908800	-3.19780600	-0.05708800
C	-7.61185200	-0.90708300	-0.72648500
H	-7.24877300	1.18928600	-1.04333600
H	-7.64070800	-3.03077800	-0.35481100
H	-8.68526700	-0.82831800	-0.86141200
H	-0.65932400	5.14499500	0.22860600
O	0.44710100	-2.10933800	0.85753500
H	1.27012300	1.46353300	0.09817500

B1

C	2.56735300	-3.66049800	0.70678900
C	1.18478300	-4.22631200	0.37935800
C	0.31349000	-3.14224800	-0.26854600
C	2.20430300	-1.90236700	-1.05062400
C	3.19770100	-2.92303200	-0.47916000
H	2.46118700	-2.94646400	1.53512100
H	2.02551900	-1.13714800	-0.28915000
H	3.46887100	-3.64874200	-1.25022100
O	0.95234100	-2.54021300	-1.36818000
C	-1.16786900	-1.40186500	0.63468500
C	-1.43992700	-0.72330100	-0.71595400
C	-0.99281400	-0.34236800	1.71868200
C	-2.74337300	0.07503400	-0.56024600
H	-1.54861700	-1.45683900	-1.51385100
C	-1.96045400	0.86158400	1.63749500
H	-3.00563000	0.56284800	-1.50192700
H	-2.76631800	0.68659100	2.35652300
O	-2.53902800	1.13264800	0.34364200
C	3.94190100	1.25434200	0.77069100
C	2.63541400	1.90453400	0.34754800
C	2.85933200	2.95629000	-0.74958300
C	0.86764900	3.22293200	1.34962500
C	1.56069300	3.71615600	-1.02088200
H	3.61287200	3.67321300	-0.41160400
C	0.98348200	4.30337400	0.26654400
H	0.67957700	3.70042900	2.31342800
H	0.82639900	3.02687100	-1.46082600
O	2.05598700	2.49100900	1.53172900
O	3.36822500	2.39372800	-1.94944200
H	2.84331500	1.60780800	-2.21583900
O	1.78724800	4.80342200	-1.91238700
H	2.36648500	4.48444100	-2.61890000
O	1.81650400	5.33027800	0.77843300
H	2.06295300	5.89348000	0.03101500
O	-1.15726400	-0.87984200	3.02854200
H	-0.49150000	-1.56794300	3.15397700
O	-0.41592400	0.17549900	-1.13417400
H	-0.44834900	0.98692100	-0.59777200
O	1.27024400	-5.30371100	-0.53619000
H	2.00011400	-5.86763700	-0.24375700
O	3.37989600	-4.75723600	1.11990000
H	4.30074800	-4.46191200	1.11210500
O	4.42087300	-2.31605900	-0.06922700
H	4.23164500	-1.60518300	0.57085400
H	-0.59659600	-3.59509600	-0.67153000
C	2.68407500	-1.22066200	-2.32210000
H	3.74570700	-0.98634400	-2.23834500
C	-1.21461200	2.13890800	2.00421100
O	2.00630300	0.01715300	-2.56425700
H	1.07306700	-0.06064300	-2.29709600
H	2.54346000	-1.89824700	-3.17154600
H	4.53682000	1.96723600	1.35126100
H	4.50894200	0.97172300	-0.11488500
O	3.74823600	0.04565200	1.51714400
H	3.32839800	0.25768600	2.35942700
H	-0.75234400	2.01577200	2.98921600
H	-1.90605100	2.98737000	2.03339500
O	-0.21556400	2.36592700	1.00122800
H	0.69882400	-4.55368000	1.30774800
H	0.03049200	0.02845200	1.60879100
H	-2.03147000	-2.03289200	0.86561800
O	-3.76276200	-0.82098400	-0.17343000
C	-5.08602200	-0.46024600	-0.35595800
C	-5.51842600	0.85417200	-0.53500000
C	-6.00462200	-1.51145100	-0.33551200
C	-6.88116200	1.10180900	-0.71418100
H	-4.81549100	1.67688000	-0.51201900
C	-7.35893800	-1.24725800	-0.50502600
H	-5.64217000	-2.52341800	-0.19255000
C	-7.80499600	0.06113800	-0.69970100
H	-7.21639900	2.12433500	-0.85526100
H	-8.06803600	-2.06861200	-0.49070800
H	-8.86163700	0.26505400	-0.83502200
H	-0.02361500	4.69276800	0.06953000
O	0.02262600	-2.19458200	0.74121400
H	1.96353200	1.12741900	-0.03046200

B2

C	-3.08030600	-3.31414700	-0.82018400
C	-1.80223000	-4.09761400	-0.52157300
C	-0.78752100	-3.18050700	0.17178500
C	-2.48442100	-1.75240200	1.07074900
C	-3.60754500	-2.56043000	0.40452700
H	-2.85731400	-2.57871700	-1.60438400
H	-2.19538400	-0.94780000	0.38738700
H	-4.02207100	-3.27966100	1.11568800
O	-1.33309900	-2.57718300	1.32287400
C	0.86044000	-1.53973000	-0.65761700
C	1.25061100	-1.02684200	0.73844500
C	0.72354700	-0.34783900	-1.59506400
C	2.60613200	-0.31406800	0.58205400
H	1.38335400	-1.85744500	1.43711000
C	1.93340600	0.59784000	-1.57769500
H	2.90909000	0.11037600	1.54103500
H	2.67746600	0.19879800	-2.27826200
O	2.53324900	0.77941000	-0.28968900
C	-3.44508700	1.67032500	-0.55083000
C	-2.06166100	2.23313000	-0.26638000
C	-2.10414400	3.26241400	0.86679800
C	-0.31505100	3.46484600	-1.41948600
C	-0.77539800	4.01166200	0.98559300
H	-2.90622800	3.98924000	0.66858200
C	-0.35547300	4.58507400	-0.37275200
H	-0.15421700	3.87545500	-2.42104200
H	-0.00987800	3.31555600	1.33445200
O	-1.60883700	2.84959300	-1.49427000
O	-2.41080600	2.57108200	2.08238600
H	-2.23892400	3.17987900	2.81484700
O	-0.88253400	5.02727100	1.97845600
H	-1.27864400	5.80654900	1.56093800
O	-1.23851600	5.63811300	-0.76982400
H	-2.01185900	5.25353700	-1.20489600
O	0.56179100	-0.75281600	-2.95507900
H	-0.15276300	-1.40200500	-2.97929700
O	0.35332800	-0.06247900	1.24739200
H	-0.27460100	-0.44342600	1.88847500
O	-2.05478000	-5.19154800	0.34206700
H	-2.84729100	-5.63677700	0.01130600
O	-4.04336800	-4.25166200	-1.30031700
H	-4.90909000	-3.82158300	-1.28778100
O	-4.70123100	-1.72642700	0.01641600
H	-4.37312100	-1.04582600	-0.59803700
H	0.06438600	-3.76625100	0.52744500
C	-2.87971700	-1.14486300	2.40973800
H	-3.85991000	-0.66838800	2.32188300
C	1.52165600	1.98992900	-2.04639200
O	-1.89495300	-0.21777300	2.87069700
H	-2.12248000	0.68719600	2.59893700
H	-2.94727400	-1.93655600	3.15955900
H	-4.07694700	2.45386600	-0.98664800
H	-3.90563300	1.34524700	0.38155600
O	-3.42580400	0.52232500	-1.40076500
H	-3.03972000	0.76385600	-2.25187800
H	0.99401100	1.90225900	-3.00087400
H	2.40726300	2.61994300	-2.17831300
O	0.68434600	2.57653700	-1.04296800
H	-1.36533000	-4.44963000	-1.46512800
H	-0.16126600	0.20157700	-1.26892100
H	1.64746300	-2.21476100	-1.00962900
O	3.54759900	-1.29618800	0.16482500
C	4.89494700	-1.04734700	0.31710200
C	5.43730100	0.21514400	0.56711400
C	5.73176900	-2.15991000	0.19343000
C	6.82007800	0.34585700	0.71152600
H	4.80134500	1.08860600	0.62412000
C	7.10713700	-2.01140900	0.33001900
H	5.28748300	-3.12964100	-0.00252200
C	7.66051400	-0.75718200	0.59403000
H	7.23768600	1.32840100	0.90786300
H	7.74914900	-2.88136600	0.23494600
H	8.73370700	-0.64309000	0.70313000
H	0.63190900	5.04116300	-0.28803300
O	-0.40357400	-2.21622000	-0.78445800
H	-1.37076200	1.43353400	0.01481200

C1

C	2.56854500	3.18662200	-0.83221000
C	1.19433500	3.85880400	-0.82614000
C	0.19080600	2.99487800	-0.05199400
C	1.90391500	1.95915300	1.26032600
C	3.01275900	2.76681900	0.57210500
H	2.51288400	2.28760000	-1.46155500
H	1.77497600	1.02230900	0.70704200
H	3.24664800	3.65967100	1.15684600
O	0.65889600	2.67683200	1.23959100
C	-1.26210600	1.11961700	-0.68869500
C	-1.64968200	0.70588000	0.73833700
C	-1.07075200	-0.10508600	-1.58765100
C	-2.93491600	-0.12815600	0.63355700
H	-1.82760100	1.58139900	1.36162700
C	-1.98798800	-1.32226500	-1.30045200
H	-3.27944400	-0.42300600	1.62757900
H	-2.74256000	-1.35904800	-2.09085000
O	-2.65837300	-1.33995400	-0.02179400
C	3.54113300	-2.43343700	-2.44261600
C	2.60462400	-2.25171500	-1.24739600
C	3.33688400	-2.13498100	0.09264700
C	0.80887800	-3.47747200	-0.17044400
C	2.31920100	-2.04075100	1.22097400
H	3.96228100	-3.02314100	0.23298600
C	1.43082000	-3.28921600	1.22008600
H	0.36756000	-4.47348300	-0.26235400
H	1.69709400	-1.16009100	1.04759700
O	1.74581100	-3.40406500	-1.22081500
O	4.18006200	-0.96726000	0.07899000
H	4.60608400	-0.93586500	0.94799000
O	2.99862000	-1.89605100	2.46284500
H	2.56778200	-1.15693300	2.93864900
O	2.17558300	-4.45323700	1.53406700
H	2.66742700	-4.26702200	2.34614600
O	-1.26023700	0.20559200	-2.96550900
H	-0.55244700	0.80038000	-3.24348600
O	-0.66273000	-0.06982700	1.41194300
H	-0.62417800	-0.96054700	1.01988500
O	1.24137200	5.12509300	-0.19291900
H	2.01757300	5.58777100	-0.53830600
O	3.48088600	4.11973600	-1.40540700
H	4.37805300	3.78877300	-1.26493900
O	4.23338400	2.02765500	0.49415500
H	4.06208800	1.15408800	0.10263500

H	-0.73529600	3.55449000	0.10647600
C	2.20598500	1.62260700	2.71488000
H	3.27700900	1.46451900	2.84307800
C	-1.15546400	-2.60022000	-1.31931300
O	1.56688700	0.41622000	3.14916300
H	0.66347100	0.38096800	2.78820700
H	1.89966100	2.46193800	3.34824400
H	2.94711900	-2.69023500	-3.32077700
H	4.23127800	-3.26538600	-2.24907800
O	4.23891500	-1.23918100	-2.75974900
H	4.73861800	-0.96305900	-1.97920200
H	-0.65777000	-2.70846000	-2.28735100
H	-1.78838400	-3.47489300	-1.13642400
O	-0.19113500	-2.47525100	-0.26579400
H	0.83963700	3.96151200	-1.85962000
H	-0.03566400	-0.41720300	-1.43132100
H	-2.07522200	1.73602200	-1.08374200
O	-3.91355600	0.65949000	-0.01100500
C	-5.24724500	0.31139100	0.10399500
C	-5.69467400	-0.94206600	0.52324200
C	-6.16074000	1.30817000	-0.24496900
C	-7.06840400	-1.17898700	0.60864800
H	-4.99349500	-1.73278500	0.75622000
C	-7.52487800	1.05335500	-0.16369800
H	-5.78689400	2.27252100	-0.57078900
C	-7.98726800	-0.19114800	0.26790200
H	-7.41485000	-2.15407100	0.93594900
H	-8.22961400	1.83298300	-0.43423000
H	-9.05197800	-0.38749500	0.33301000
H	0.61393800	-3.15827700	1.94102900
O	-0.02555500	1.83386800	-0.82783800
H	2.01097000	-1.34400700	-1.41329300

C2

C	-2.34622900	3.69251200	0.93878400
C	-0.85062800	4.03087100	0.98848900
C	-0.01465100	3.00782200	0.20647300
C	-1.90981200	2.36177100	-1.11998700
C	-2.81337300	3.42729400	-0.49897700
H	-2.53605000	2.78855400	1.52436800
H	-1.97141000	1.45727000	-0.50803900
H	-2.73811500	4.35481600	-1.08836700
O	-0.54712000	2.85377200	-1.10185700
C	1.20859900	0.98808400	0.74938600
C	1.60854300	0.67217400	-0.69889100
C	0.91348900	-0.29826200	1.51781000
C	2.87263400	-0.19648300	-0.63662100
H	1.82395600	1.58368400	-1.25546900
C	1.84684700	-1.49743900	1.19160100
H	3.21987600	-0.43561100	-1.64460200
H	2.56857700	-1.58059500	2.00858800
O	2.56993500	-1.43697100	-0.05790800
C	-3.45591900	-3.27898500	2.33142900
C	-2.59994200	-2.77582100	1.16820000
C	-3.42621800	-2.34856700	-0.04998200
C	-0.84280200	-3.58915400	-0.26846100
C	-2.49544500	-1.93956100	-1.18270800
H	-4.04695600	-3.19233700	-0.37409500
C	-1.56108000	-3.10484900	-1.53486800
H	-0.32938900	-4.53817000	-0.44688000
H	-1.89949700	-1.08625900	-0.84655500
O	-1.70764200	-3.84330400	0.80815700
O	-4.25786400	-1.25864500	0.35025100
H	-4.66372400	-0.88728600	-0.44488500
O	-3.27350900	-1.55292800	-2.31158200
H	-2.82828800	-0.78389400	-2.72036600
O	-2.27870300	-4.19373100	-2.08603200
H	-2.87321300	-3.83005400	-2.75767700
O	1.00460100	-0.11579200	2.92752700
H	0.24786800	0.40600700	3.22142000
O	0.60987500	-0.01920300	-1.44128500
H	0.53079800	-0.93526800	-1.11800600
O	-0.62883200	5.34370200	0.46091100
H	-0.61467000	5.29731400	-0.50520200
O	-3.12707500	4.71995000	1.53700000
H	-2.72702300	5.56893100	1.29613000
O	-4.15222900	2.95975300	-0.51694700
H	-4.66814200	3.52110700	0.07832100

H	0.99280900	3.40930000	0.05902600
C	-2.24729300	1.99635200	-2.55518300
H	-3.32977700	1.93409900	-2.66517200
C	1.02242200	-2.77572700	1.08635800
O	-1.72388200	0.72085200	-2.93020200
H	-0.81825600	0.61725500	-2.58524200
H	-1.87290400	2.77409600	-3.23177700
H	-2.80748100	-3.76115900	3.06497900
H	-4.16999200	-4.03131500	1.96909000
O	-4.11369000	-2.21738700	3.00282700
H	-4.58796500	-1.69019800	2.34323300
H	0.49640300	-2.96872400	2.02544000
H	1.66788500	-3.62932200	0.85361400
O	0.09487800	-2.55875300	0.01897800
H	-0.51294000	4.07340600	2.02501200
H	-0.10984500	-0.57523500	1.25484400
H	2.04569000	1.50999300	1.22172600
O	3.86306100	0.53525500	0.05826700
C	5.19052400	0.16931400	-0.07343700
C	5.61489500	-1.06986500	-0.55471300
C	6.12227200	1.12966400	0.32587600
C	6.98394000	-1.32836800	-0.65061800
H	4.89915300	-1.83447800	-0.82728200
C	7.48157300	0.85316500	0.23335900
H	5.76667100	2.08335700	0.70009100
C	7.92088400	-0.37665500	-0.25974600
H	7.31232300	-2.29233300	-1.02586000
H	8.20057000	1.60442800	0.54372400
H	8.98175200	-0.58998400	-0.33330300
H	-0.79810400	-2.76109600	-2.24621600
O	0.02261400	1.79285300	0.90566500
H	-2.02532500	-1.91140600	1.52378500

C3

C	2.32528600	4.26025200	-1.03484700
C	0.83087300	4.57948700	-0.92962500
C	0.08948900	3.47671200	-0.16200600
C	2.09166200	2.73725700	0.93646900
C	2.91953200	3.87015100	0.31944100
H	2.45261300	3.41423500	-1.72782600
H	2.13008000	1.86185700	0.27095400
H	2.90888800	4.73538200	0.98531500
O	0.72753800	3.16533200	1.06264200
C	-1.11099100	1.44817200	-0.82442800
C	-1.43210800	1.03762000	0.62348700
C	-0.73090700	0.21943500	-1.64403100
C	-2.56045000	-0.00515200	0.56900800
H	-1.78620600	1.89549500	1.20273100
C	-1.71737100	-0.95025200	-1.49415600
H	-2.78019600	-0.35955600	1.57792700
H	-2.58425400	-0.75495600	-2.13737900
O	-2.15734700	-1.14932800	-0.14185500
C	3.27584400	-5.24432500	-1.12338200
C	2.46811900	-4.00292800	-0.74325800
C	3.03676100	-3.26895200	0.48123200
C	0.22469800	-3.37238800	-0.17543800
C	2.11760500	-2.12486100	0.90236400
H	3.11137500	-3.98964200	1.30902000
C	0.68170800	-2.62713000	1.08459400
H	-0.74273800	-3.84987400	-0.00287500
H	2.14177800	-1.34557500	0.13911500
O	1.12786100	-4.43951600	-0.44270700
O	4.34797800	-2.79988600	0.15880700
H	4.68130000	-2.29857800	0.91447000
O	2.60705700	-1.51526600	2.11136500
H	2.25791800	-2.03892700	2.84999000
O	0.61139900	-3.47994200	2.23648300
H	0.87235000	-4.37542500	1.97962600
O	-0.68249800	0.51098500	-3.03933000
H	-0.02269800	1.20162400	-3.17601300
O	-0.35799300	0.39061500	1.26904100
H	0.18044700	0.99792500	1.80881900
O	0.62147400	5.78991900	-0.22352200
H	1.24039000	6.44113400	-0.58251200
O	2.95915400	5.41828300	-1.56997700
H	3.90921300	5.34902700	-1.40511600
O	4.30494700	3.53682200	0.17008600
H	4.38875400	2.74007800	-0.37225700
H	-0.89949700	3.84612700	0.11986500
C	2.54795600	2.31043500	2.32655600
H	3.62267000	2.10764700	2.31677700
C	-1.07203700	-2.26301800	-1.93186000
O	1.80940100	1.18429100	2.78764500
H	2.22452200	0.34526800	2.51697800
H	2.35956500	3.12228500	3.03281800
H	2.71332100	-5.81684200	-1.86229300
H	3.41348700	-5.88299900	-0.23811300
O	4.51797400	-4.91451100	-1.72020200
H	4.96738900	-4.26995900	-1.15482400
H	-0.85429400	-2.21172600	-2.99962800
H	-1.76062400	-3.09439100	-1.75161800
O	0.17261800	-2.49004100	-1.26703700
H	0.40463500	4.64628300	-1.93842800
H	0.25091400	-0.10767100	-1.29409400
H	-1.99865500	1.92339200	-1.25534200
O	-3.69755000	0.62643500	0.00691800
C	-4.94680500	0.07146000	0.19914500
C	-5.16447200	-1.24024700	0.62432700
C	-6.02830900	0.91200100	-0.07592000
C	-6.47511800	-1.69338200	0.78896800
H	-4.33294000	-1.90935700	0.80230800
C	-7.32706800	0.44288200	0.08371900
H	-5.83310500	1.92624700	-0.40641700
C	-7.55882600	-0.86250600	0.52106600
H	-6.64143000	-2.71313000	1.12147500
H	-8.16198800	1.10265100	-0.12954600
H	-8.57280500	-1.22620700	0.64785300
H	0.02285500	-1.78615900	1.28395300
O	0.00792200	2.34503200	-0.99508200
H	2.44704900	-3.32380000	-1.60047000

C4

C	2.70775100	3.22618800	-1.03083600
C	1.42371000	4.06504900	-0.98547000
C	0.33049700	3.30036200	-0.22805300
C	1.94307100	2.16281600	1.14312700
C	3.12636700	2.73797600	0.36387700
H	2.53379300	2.34717100	-1.65488600
H	1.66389600	1.22051600	0.66892000
H	3.56965900	3.57821800	0.91831300
O	0.80287800	3.04776700	1.09319800
C	-1.18196000	1.41924300	-0.79691500
C	-1.57091700	1.04173300	0.64396700
C	-0.94564000	0.17767600	-1.65685500
C	-2.76322200	0.07399600	0.56710200
H	-1.87456700	1.93229500	1.20243900
C	-1.92783100	-0.98731100	-1.43745600
H	-3.05195700	-0.23311000	1.57441300
H	-2.78444000	-0.84647700	-2.10659300
O	-2.39645300	-1.11223800	-0.08675200
C	3.87237100	-3.85635000	-1.49363500
C	2.70683800	-3.08490900	-0.87356100
C	3.05440800	-2.48699400	0.50711200
C	0.40959800	-3.40214500	-0.30291300
C	1.81072800	-1.88391300	1.16444400
H	3.43035700	-3.29848100	1.14182300
C	0.62047300	-2.85572300	1.11499400
H	-0.34156200	-4.19723900	-0.29418200
H	1.52062100	-0.96949900	0.64432400
O	1.61001800	-4.01507100	-0.74145400
O	4.14650500	-1.57057100	0.39979700
H	3.85645300	-0.64758100	0.33697000
O	2.06584900	-1.50416300	2.52130500
H	1.95472800	-2.29480600	3.07053800
O	0.83573800	-3.93024500	2.03920400
H	1.38668000	-4.60156600	1.61220700
O	-1.01035000	0.48003800	-3.05048000
H	-0.24189900	1.01555400	-3.28038700
O	-0.54949600	0.33848500	1.31627000
H	-0.15137700	0.85709700	2.04158500
O	1.67180800	5.32796600	-0.36526200
H	1.60140600	5.22002700	0.59411500
O	3.77486700	3.93873800	-1.64547400
H	3.73082000	4.85611600	-1.33737800
O	4.08776800	1.68224300	0.25800900
H	4.81607900	1.99257100	-0.29690900
H	-0.56382000	3.91814400	-0.09692500
C	2.24712200	1.90947100	2.61512700
H	3.24123400	1.46360800	2.70470100
C	-1.24546900	-2.31291800	-1.77843900
O	1.26410600	1.07294100	3.21845100
H	1.54944900	0.14242700	3.12920200
H	2.24278700	2.85924000	3.15667800
H	3.51559600	-4.38220900	-2.38070800
H	4.23652500	-4.61153600	-0.78078700
O	4.91835000	-2.99820500	-1.91393200
H	5.11856200	-2.39394200	-1.18277900
H	-1.11864200	-2.38562100	-2.86140800
H	-1.86896800	-3.14376800	-1.43633900
O	0.04985200	-2.36500400	-1.17523600
H	1.09625000	4.28999800	-2.00192300
H	0.05370300	-0.17410800	-1.40185600
H	-1.98471300	2.02486800	-1.22842400
O	-3.83108500	0.75737000	-0.06781900
C	-5.12026200	0.28474700	0.07494000
C	-5.43970800	-0.99214300	0.53994600
C	-6.13278600	1.17493300	-0.29152700
C	-6.78247000	-1.35900500	0.65307500
H	-4.66146900	-1.70176400	0.78823200
C	-7.46434100	0.79124900	-0.18204100
H	-5.85929100	2.16021800	-0.65270700
C	-7.79806400	-0.47753400	0.29525000
H	-7.02771800	-2.35182000	1.01674100
H	-8.24516700	1.48938100	-0.46627400
H	-8.83762000	-0.77436400	0.38282200
H	-0.27415300	-2.33390900	1.45262100
O	0.06833400	2.13238100	-0.94566200
H	2.41765800	-2.28408700	-1.56105300

D1

C	-2.59334500	3.19287700	0.95229500
C	-1.21162600	3.84823300	0.98206300
C	-0.21610400	3.01087000	0.16888400
C	-1.94007300	2.05057200	-1.18893300
C	-3.03918300	2.84538900	-0.47079800
H	-2.54899900	2.26407200	1.53802300
H	-1.82430600	1.08996400	-0.67579400
H	-3.25015400	3.76869200	-1.01596500
O	-0.68696600	2.75402400	-1.13520400
C	1.23108000	1.10825500	0.72212900
C	1.61301200	0.75531400	-0.72321600
C	1.04742000	-0.15082300	1.57405400
C	2.89312800	-0.09011700	-0.66297100
H	1.79418500	1.65713800	-1.30702000
C	1.95173900	-1.36124400	1.22211200
H	3.23089100	-0.34256800	-1.67083900
H	2.71281100	-1.43965800	2.00316500
O	2.61088100	-1.32897100	-0.06146400
C	-3.63515800	-2.42320200	2.28075900
C	-2.68710900	-2.20090400	1.11174600
C	-3.39764500	-2.03217600	-0.23153400
C	-0.88156200	-3.43494500	0.03523300
C	-2.35581900	-1.95264700	-1.34282200
H	-4.05635700	-2.89040200	-0.40397700
C	-1.49305900	-3.21713800	-1.35476700
H	-0.45805700	-4.43982600	0.11499100
H	-1.71789300	-1.08918500	-1.14307000
O	-1.83089900	-3.36269400	1.07531300
O	-4.18034600	-0.83127800	-0.20252500
H	-4.59842500	-0.76689300	-1.07310900
O	-3.00923000	-1.76976000	-2.59364200
H	-2.58241100	-1.00473700	-3.02949200
O	-2.25702100	-4.36432900	-1.68604500
H	-2.73993100	-4.16081800	-2.49924500
O	1.26507600	0.10101200	2.95967300
H	0.53997000	0.64516400	3.29070300
O	0.62009900	0.01706200	-1.42866000
H	0.57514200	-0.88917200	-1.07545400
O	-1.24260600	5.14441200	0.41118300
H	-2.01749300	5.59629200	0.77353200
O	-3.49929500	4.10558000	1.56723300
H	-4.39871400	3.80187900	1.38420200
O	-4.27573100	2.13246200	-0.42879800
H	-4.12788200	1.23412800	-0.08445100
H	0.71369800	3.57069500	0.03445500
C	-2.24092700	1.78015900	-2.65720600
H	-3.31152800	1.62499900	-2.79237900
C	1.10614500	-2.63030300	1.19451500
O	-1.59741800	0.59768100	-3.14687600
H	-0.69613600	0.54589000	-2.78284100
H	-1.93781500	2.64889700	-3.25143200
H	-4.23153600	-3.32641200	2.09642400
H	-4.31316600	-1.57439900	2.36746000
O	-2.93939500	-2.51567600	3.51468500
H	-2.34899600	-3.27800300	3.46597600
H	0.61652600	-2.77037100	2.16274600
H	1.72738200	-3.50372100	0.97007900
O	0.13357800	-2.45244100	0.15646800
H	-0.85773200	3.89679400	2.01979800
H	0.00804600	-0.45126700	1.42463900
H	2.04495500	1.70938600	1.13862900
O	3.88055200	0.66081700	0.01107500
C	5.21146700	0.31426200	-0.13570300
C	5.64840000	-0.92459900	-0.60640600
C	6.13332400	1.29501200	0.23577100
C	7.01984300	-1.16249300	-0.72030900
H	4.94065900	-1.70374500	-0.85804600
C	7.49528200	1.03914000	0.12543500
H	5.76761300	2.24815500	0.60136000
C	7.94696400	-0.19031100	-0.35747700
H	7.35815400	-2.12604000	-1.08781800
H	8.20662700	1.80645600	0.41330500
H	9.00993500	-0.38722900	-0.44533300
H	-0.67037900	-3.09221900	-2.07013500
O	-0.00582000	1.81504100	0.89188600
H	-2.07951800	-1.31056500	1.31537300

D2

C	-2.10750300	3.96139800	1.09834200
C	-0.58973800	4.18495200	1.11307300
C	0.14824300	3.10956100	0.30286800
C	-1.82308600	2.61250500	-0.97472200
C	-2.62918900	3.73901800	-0.32819400
H	-2.35110900	3.07123900	1.68534000
H	-1.93350200	1.71090500	-0.36503000
H	-2.49822000	4.66079500	-0.91703600
O	-0.42836600	3.00532800	-0.99086900
C	1.24002900	1.00431800	0.79477000
C	1.58367700	0.68600900	-0.66688400
C	0.87542500	-0.27131500	1.55199800
C	2.79100700	-0.26180600	-0.64812700
H	1.84434300	1.59022500	-1.21580500
C	1.71589100	-1.52640200	1.18119400
H	3.09565500	-0.50877300	-1.66785500
H	2.43893400	-1.68064600	1.98649500
O	2.42836800	-1.48870300	-0.07506200
C	-3.74928100	-3.58384400	2.03778500
C	-2.86972500	-2.87088700	1.02185500
C	-3.65517700	-2.29846400	-0.16145800
C	-1.01380900	-3.44907100	-0.42255200
C	-2.68421800	-1.75462200	-1.20408200
H	-4.26150100	-3.09283900	-0.61477900
C	-1.70846200	-2.85087100	-1.65104000
H	-0.45463500	-4.35170200	-0.68570700
H	-2.12286700	-0.93337400	-0.74785600
O	-1.91059500	-3.85509900	0.57643900
O	-4.49049200	-1.26400700	0.34191300
H	-4.86226200	-0.79811800	-0.41939400
O	-3.42768700	-1.26057300	-2.31414700
H	-2.95195600	-0.47764700	-2.65750000
O	-2.37897800	-3.89172600	-2.33743400
H	-2.97545000	-3.47051100	-2.97292000
O	1.02076000	-0.11845100	2.96090100
H	0.31619700	0.45650000	3.28362100
O	0.52561900	0.07248600	-1.39327900
H	0.37628800	-0.83287800	-1.06332600
O	-0.28247600	5.48178400	0.58849500
H	-0.29771100	5.44259800	-0.37794200
O	-2.79319300	5.04124500	1.72023500
H	-2.33618000	5.85910900	1.47313500
O	-3.99875900	3.37367200	-0.31368500
H	-4.45760500	3.97096100	0.29316100
H	1.17769100	3.43924000	0.13119900
C	-2.22275400	2.27793100	-2.40132700
H	-3.30986400	2.27929700	-2.47932500
C	0.80259800	-2.74101400	1.05580500
O	-1.78338200	0.97776000	-2.79728800
H	-0.88117000	0.81419400	-2.46575900
H	-1.82361200	3.03769800	-3.08438500
H	-4.29211600	-4.39792100	1.53927500
H	-4.47577900	-2.88117100	2.44411500
O	-2.99533300	-4.07726000	3.13632500
H	-2.34805600	-4.70436800	2.78992600
H	0.28404700	-2.92951000	2.00037200
H	1.38486900	-3.62663800	0.78009700
O	-0.13092900	-2.42602500	0.01821500
H	-0.22445300	4.19381700	2.14105400
H	-0.17265000	-0.47266000	1.31690900
H	2.12252100	1.45876300	1.25416900
O	3.84408500	0.39803800	0.02699600
C	5.14226500	-0.04545000	-0.15030300
C	5.47582000	-1.30318200	-0.65448000
C	6.14258400	0.85317900	0.22612800
C	6.82301000	-1.64269200	-0.79630800
H	4.70682500	-2.02050400	-0.90989700
C	7.47911800	0.49622000	0.08819100
H	5.85742900	1.82311900	0.61839800
C	7.82765200	-0.75292600	-0.42822600
H	7.08053600	-2.62100300	-1.18933400
H	8.25161800	1.19991000	0.38100600
H	8.87083400	-1.02893800	-0.53751500
H	-0.93617500	-2.41466800	-2.29922100
O	0.11791200	1.88823900	0.99239700
H	-2.33962000	-2.05344500	1.52522600

D3

C	2.64201500	3.27474400	-1.03391000
C	1.28706700	3.98469000	-1.04067300
C	0.26994400	3.16702800	-0.23432100
C	1.97178200	2.16588400	1.13054400
C	3.09150100	2.89894500	0.38028100
H	2.55090600	2.35290700	-1.62492300
H	1.82278300	1.18917400	0.65539800
H	3.37337100	3.80665200	0.91897600
O	0.73835000	2.89921800	1.07108200
C	-1.17632800	1.24197400	-0.78528900
C	-1.58197600	0.93439800	0.66576400
C	-0.90223100	-0.04951300	-1.55163200
C	-2.81251500	0.01381300	0.60394800
H	-1.86835500	1.85346300	1.18470700
C	-1.88812600	-1.20957500	-1.29509300
H	-3.12966500	-0.24541800	1.61600100
H	-2.65707300	-1.17489900	-2.07455500
O	-2.51935900	-1.21021000	-0.00708900
C	3.80117500	-3.01333200	-1.91645100
C	2.68258000	-2.59320100	-0.97442500
C	3.17928200	-2.16498700	0.41056200
C	0.66296900	-3.53678900	-0.02754600
C	2.00336200	-1.98536400	1.37486600
H	3.85256800	-2.94253400	0.79998900
C	1.11929000	-3.23484000	1.40842200
H	0.10621000	-4.47778600	-0.08412600
H	1.38761100	-1.14667700	1.05014600
O	1.81787200	-3.74719000	-0.84869600
O	3.91857500	-0.94608200	0.28071100
H	4.25573500	-0.72736900	1.16017500
O	2.49324800	-1.63267300	2.67782400
H	2.65519300	-2.44588500	3.17799300
O	1.80507900	-4.33958600	1.99947400
H	2.35808800	-4.76431300	1.32924000
O	-0.91250700	0.15489200	-2.96481200
H	-0.19447900	0.76135300	-3.18386200
O	-0.58984600	0.22999900	1.38373100
H	-0.13448400	0.79781800	2.03045800
O	1.37416500	5.26876700	-0.44881000
H	2.14850400	5.70630200	-0.82894500
O	3.57600600	4.15471000	-1.65622600
H	4.46573900	3.81461500	-1.49229900
O	4.28527300	2.10970300	0.31551300
H	4.07650300	1.24374700	-0.07109200
H	-0.64863000	3.74370300	-0.09514400
C	2.27742200	1.95299500	2.60860200
H	3.31314300	1.62355600	2.72641300
C	-1.13917900	-2.53921900	-1.37761100
O	1.37383800	1.02109300	3.20505200
H	1.76418000	0.13113300	3.20099400
H	2.15625200	2.90149900	3.13686700
H	4.35267300	-3.85496000	-1.47290800
H	4.49546600	-2.18424700	-2.05296500
O	3.31073300	-3.35038500	-3.20242700
H	2.65202300	-4.04880400	-3.09709800
H	-0.74091100	-2.68523900	-2.38696800
H	-1.80899000	-3.36745800	-1.12357900
O	-0.08707600	-2.44251200	-0.41708000
H	0.92781000	4.06358400	-2.07457800
H	0.09105600	-0.36682500	-1.23442400
H	-1.99325800	1.79373000	-1.26058500
O	-3.84159100	0.73758300	-0.05996500
C	-5.15229200	0.33508100	0.08023100
C	-5.54387800	-0.90731200	0.58368600
C	-6.11480700	1.26179000	-0.33004400
C	-6.90496400	-1.20102700	0.69037600
H	-4.80690400	-1.64712100	0.86712800
C	-7.46547000	0.95061900	-0.22616700
H	-5.78675000	2.21907700	-0.72002000
C	-7.87003800	-0.28242300	0.28873000
H	-7.20522100	-2.16706700	1.08402900
H	-8.20573100	1.67770500	-0.54459100
H	-8.92449900	-0.52290600	0.37167100
H	0.24586100	-3.04934100	2.03430400
O	0.04650600	1.98334500	-0.96450700
H	2.11962100	-1.77282400	-1.42914300

D4

C	3.67497200	-3.22332600	1.12621600
C	2.38940900	-4.04642900	0.97555100
C	1.31566700	-3.25792800	0.21357200
C	2.96718400	-1.89002100	-0.86950200
C	4.12906700	-2.63898600	-0.21810000
H	3.49506700	-2.39037700	1.81132700
H	2.66414300	-1.07044200	-0.21038200
H	4.44482900	-3.45873500	-0.88306600
O	1.85455400	-2.80309200	-1.02349300
C	-0.49255900	-1.73703200	0.82836800
C	-0.92183200	-1.48017700	-0.62697200
C	-0.55930800	-0.44298900	1.63327000
C	-2.33815500	-0.88227800	-0.59268800
H	-0.96021000	-2.41558200	-1.19384400
C	-1.88374400	0.31988400	1.45767900
H	-2.65534600	-0.63817600	-1.60828500
H	-2.63840200	-0.14934700	2.10086900
O	-2.35360200	0.33895000	0.10106200
C	1.28647100	6.08646400	0.97832600
C	1.00900500	4.62763700	0.65081600
C	1.82842800	4.10378700	-0.53530900
C	-0.87668900	3.23200800	0.09979500
C	1.34774400	2.70689100	-0.93760500
H	1.68994500	4.78576800	-1.38909900
C	-0.16979100	2.67641700	-1.14132000
H	-1.94783700	3.33666700	-0.08899200
H	1.62941800	1.99985000	-0.15559500
O	-0.40451300	4.55210000	0.34301100
O	3.19620500	4.09646000	-0.15174700
H	3.70024300	3.66021300	-0.85121500
O	2.03006500	2.27062300	-2.12801700
H	1.55076300	2.64996600	-2.88125600
O	-0.51987600	3.43676200	-2.30702400
H	-0.56824900	4.37228800	-2.06548000
O	-0.43752100	-0.68626000	3.03298400
H	0.44739300	-1.03015900	3.20358300
O	-0.12678800	-0.51615400	-1.27743400
H	0.59813200	-0.90012500	-1.80597800
O	2.66987200	-5.27496900	0.29665500
H	2.69509100	-5.10831800	-0.65606900
O	4.71808900	-3.99442100	1.71128500
H	4.65932500	-4.88967400	1.34544000
O	5.20657200	-1.73333000	-0.03597100
H	5.85245900	-2.15742200	0.54577700
H	0.49791500	-3.93039500	-0.06259100
C	3.27750300	-1.32162600	-2.24628500
H	4.19781200	-0.73501600	-2.19032200
C	-1.72790300	1.77977100	1.87651300
O	2.19168400	-0.54295000	-2.73985900
H	2.28551000	0.39582000	-2.49273700
H	3.43173800	-2.13854300	-2.95656700
H	1.07099900	6.70646700	0.09553900
H	2.33878200	6.20779700	1.23336900
O	0.53689500	6.52782000	2.09854000
H	-0.39265400	6.32728000	1.92889500
H	-1.51386000	1.82053500	2.94543500
H	-2.65861700	2.32221500	1.68216600
O	-0.63165300	2.41038300	1.21221100
H	2.01391400	-4.33450200	1.95867900
H	0.25763800	0.19526200	1.28960500
H	-1.16615400	-2.48136300	1.26577500
O	-3.19824500	-1.85536900	-0.02516200
C	-4.55967200	-1.76091400	-0.23388200
C	-5.20350300	-0.61287600	-0.69886000
C	-5.29608900	-2.91018500	0.06388100
C	-6.58774200	-0.63623700	-0.88235500
H	-4.64659400	0.29409100	-0.89432500
C	-6.67466900	-2.91424000	-0.11471900
H	-4.77350400	-3.78839900	0.42679500
C	-7.32920200	-1.77784400	-0.59344800
H	-7.08521100	0.25703300	-1.24657000
H	-7.23937200	-3.81177400	0.11639900
H	-8.40451400	-1.78346700	-0.73531000
H	-0.49243700	1.65647400	-1.33252600
O	0.86619100	-2.20018500	1.01040100
H	1.21085900	4.01452000	1.53337200

E1

C	-5.04310200	-0.33412200	1.03437100
C	-4.78089200	1.12883800	0.64822200
C	-3.44466600	1.27863300	-0.09413000
C	-3.46943400	-1.01740600	-0.77626800
C	-4.83948900	-1.28399400	-0.15666200
H	-4.34459600	-0.63260400	1.82102200
H	-2.69929000	-1.19091000	-0.01920600
H	-5.61909600	-1.09683500	-0.91206400
O	-3.42085200	0.37089400	-1.18637900
C	-1.11895800	1.71301000	0.61536700
C	-0.62795100	1.88665000	-0.83228200
C	-0.14179200	0.86997100	1.44018500
C	0.78569600	2.48677700	-0.77078400
H	-1.28575000	2.54713900	-1.39832500
C	1.34196500	1.20993600	1.21487100
H	1.18207000	2.62195900	-1.78067500
H	1.59525100	2.08059000	1.82816600
O	1.63626200	1.52621600	-0.16973800
C	5.16275400	-3.50631700	-0.02025100
C	3.70703400	-3.30323800	0.37043100
C	2.79653700	-4.45301800	-0.05110400
C	1.95733700	-1.69095400	-0.06334200
C	1.34796400	-4.12676300	0.31586200
H	2.86939800	-4.57960200	-1.14087400
C	0.89990100	-2.79478000	-0.30116400
H	1.81095500	-0.87811200	-0.77696600
H	1.27616400	-4.06658700	1.40520000
O	3.27938600	-2.10099300	-0.30588300
O	3.22616000	-5.63431900	0.61239000
H	2.52406900	-6.29187100	0.51144700
O	0.48563200	-5.18713700	-0.07972900
H	0.29558200	-5.04681700	-1.02056600
O	0.67714200	-3.00213800	-1.69167900
H	-0.18205600	-2.61578200	-1.95036200
O	-0.36364000	1.02984900	2.83644500
H	-1.19794300	0.60418200	3.06690300
O	-0.52788200	0.67547800	-1.57728800
H	0.34516900	0.29138900	-1.41337800
O	-5.84646900	1.61622300	-0.17381000
H	-5.70411600	1.31351900	-1.08180400
O	-6.34443400	-0.49523200	1.58475600
H	-6.94607600	0.07802500	1.08655200
O	-4.89471400	-2.63715600	0.26189500
H	-5.67927600	-2.74484000	0.81701800
H	-3.39225500	2.27400000	-0.54352800
C	-3.12683200	-1.85621400	-1.99407900
H	-3.27501300	-2.91059500	-1.76286700
C	2.26041800	0.04865500	1.62078800
O	-1.75785600	-1.68921800	-2.36253400
H	-1.54036000	-0.74115300	-2.32448100
H	-3.77946000	-1.58748600	-2.83412600
H	5.22909700	-3.67542900	-1.10366900
H	5.55661900	-4.38413700	0.49022400
O	5.97328200	-2.40488700	0.36748400
H	5.60919400	-1.61488400	-0.05143500
H	2.33999300	0.03581500	2.70769100
H	3.25736400	0.21205900	1.20167000
O	1.76267200	-1.24123900	1.26784900
H	-4.78940900	1.75669200	1.54051300
H	-0.30354000	-0.17386200	1.16032000
H	-1.19054100	2.71342200	1.05313100
O	0.73105600	3.71121900	-0.08875600
C	1.77240100	4.61606300	-0.23770400
C	3.04813500	4.26505400	-0.67793100
C	1.47568200	5.93555300	0.10530900
C	4.02325000	5.25878700	-0.78884900
H	3.29315000	3.23602200	-0.90734600
C	2.46048600	6.91131700	-0.00146600
H	0.47600100	6.17906400	0.44754000
C	3.73874100	6.57886900	-0.45316800
H	5.01618000	4.98633500	-1.13137900
H	2.22603200	7.93664100	0.26522200
H	4.50485200	7.34184900	-0.53758400
H	-0.02993400	-2.47741200	0.18021600
O	-2.39090500	1.05305100	0.80205900
H	3.64173200	-3.15248600	1.45381600

F1

C	-3.80706900	-3.29624000	1.07120400
C	-4.49459300	-1.97610200	0.69280300
C	-3.53724100	-1.04493300	-0.06603400
C	-2.16763300	-2.88256800	-0.76061200
C	-3.08508900	-3.92774700	-0.12969500
H	-3.05980100	-3.10691500	1.84686800
H	-1.44469600	-2.54982400	-0.01038200
H	-3.82855400	-4.25241300	-0.87456800
O	-2.97937000	-1.75295500	-1.16396800
C	-1.95882800	0.72525400	0.62162500
C	-1.69022000	1.15993300	-0.82988800
C	-0.66271100	0.66189100	1.43461900
C	-0.94018900	2.50001200	-0.77919300
H	-2.61982100	1.27573000	-1.38829200
C	0.30360100	1.83523500	1.19382400
H	-0.72075300	2.84878400	-1.79178600
H	-0.01767200	2.68109100	1.80986900
O	0.32697900	2.26244500	-0.19183900
C	6.22001600	0.42343900	-0.01312100
C	4.91555300	-0.29895300	0.32689200
C	4.88705800	-1.75170700	-0.14688600
C	2.55820200	-0.08199000	-0.11253700
C	3.55607700	-2.39844500	0.22545800
H	4.99894400	-1.76035600	-1.23993500
C	2.38321700	-1.60052700	-0.36369200
H	1.94965200	0.48071800	-0.82287400
H	3.47372600	-2.42403600	1.31533300
O	3.85583900	0.40490000	-0.34287000
O	5.97509600	-2.45404200	0.45497400
H	5.82968100	-3.39973400	0.31443100
O	3.52442400	-3.75345400	-0.20801300
H	3.26372100	-3.73288300	-1.14243700
O	2.31181200	-1.88516200	-1.75673300
H	1.39386400	-2.11506900	-2.00021200
O	-0.92275900	0.66013900	2.83369800
H	-1.31610900	-0.18735300	3.07316700
O	-0.87575600	0.26093600	-1.57799000
H	0.05111900	0.48771100	-1.41623600
O	-5.65073800	-2.23831200	-0.10913200
H	-5.37067700	-2.39290100	-1.02224700
O	-4.73364000	-4.21514800	1.63633700
H	-5.56771800	-4.12538800	1.15148300
O	-2.30091500	-5.03604000	0.27804700
H	-2.85022900	-5.59691300	0.84289600
H	-4.10945100	-0.22581600	-0.51005800
C	-1.39606200	-3.33744800	-1.98590100
H	-0.86312600	-4.26022600	-1.75879700
C	1.74448400	1.47245000	1.58050600
O	-0.42241000	-2.36525600	-2.36707000
H	-0.83069500	-1.48251800	-2.32318600
H	-2.08525100	-3.52743700	-2.81818000
H	6.08763200	1.49328100	0.15843100
H	6.45339700	0.28105000	-1.07783400
O	7.29247700	0.00641700	0.81371200
H	7.33920000	-0.96030600	0.78096100
H	1.83017100	1.51131200	2.66648900
H	2.43216300	2.20546600	1.14924700
O	2.12523500	0.14455300	1.22035600
H	-4.86845000	-1.48106900	1.59035100
H	-0.15599500	-0.26503600	1.15475800
H	-2.62779400	1.46990800	1.06390600
O	-1.72622900	3.43477500	-0.08921700
C	-1.45428400	4.78759300	-0.23521600
C	-0.23609200	5.28904900	-0.69241000
C	-2.48862700	5.65075200	0.12789200
C	-0.07135800	6.67175000	-0.80080100
H	0.58191100	4.62384800	-0.93703500
C	-2.30518000	7.02504700	0.02307800
H	-3.42372800	5.23256300	0.48358200
C	-1.09670500	7.54294100	-0.44593100
H	0.87620900	7.06265000	-1.15685600
H	-3.11210200	7.69358200	0.30476500
H	-0.95645000	8.61518500	-0.52867400
H	1.45773800	-1.91633800	0.12647900
O	-2.55190900	-0.57803100	0.81449800
H	4.76690400	-0.25859700	1.41234600

G1

C	-2.10750800	-4.67770900	0.35754300
C	-1.49598600	-3.85987000	1.50084700
C	-0.98045400	-2.50530700	0.99673500
C	-2.49546700	-2.53680800	-0.87636600
C	-3.13173200	-3.85426200	-0.43331400
H	-1.31681000	-4.99019500	-0.32917400
H	-1.64892900	-2.75645400	-1.53461900
H	-3.99458600	-3.63360600	0.21555400
O	-2.02064800	-1.83503700	0.29230600
C	1.13241400	-1.70863300	0.02023000
C	2.00304700	-1.52041600	1.27500600
C	0.59316300	-0.36140900	-0.49658200
C	3.13923000	-0.54236800	0.96488100
H	2.40960500	-2.48451200	1.58417700
C	1.76605300	0.64488900	-0.60313400
H	3.71850600	-0.33353000	1.86781800
H	2.38941000	0.32301500	-1.44514800
O	2.57850900	0.70036300	0.58682400
C	-3.03289600	2.96873700	-2.16556400
C	-2.18095400	2.99415200	-0.89844000
C	-2.92066400	3.54563900	0.32216200
C	-0.07337900	3.80561200	-0.09813900
C	-1.99130400	3.56796400	1.53628100
H	-3.24703200	4.57193600	0.09564900
C	-0.69477900	4.32120500	1.20652700
H	0.70930400	4.49337700	-0.43742900
H	-1.75583500	2.53724400	1.81353500
O	-1.04223500	3.83559900	-1.14933800
O	-4.07336200	2.73979100	0.56648400
H	-4.43569100	2.98506700	1.42898700
O	-2.65817700	4.13929300	2.65614600
H	-2.53657000	5.09965800	2.60942700
O	-0.95674700	5.72512200	1.11384600
H	-1.27928300	5.93035200	0.22540900
O	0.07217500	-0.47608100	-1.80451600
H	-0.90537300	-0.48063000	-1.81437500
O	1.28268700	-1.01551700	2.40141200
H	1.19757500	-0.05614400	2.30450000
O	-2.47137700	-3.66269700	2.53242200
H	-3.01645900	-2.89794600	2.30130700
O	-2.70994100	-5.87519700	0.84157300
H	-3.14228600	-5.65909200	1.68138600
O	-3.57363300	-4.56609100	-1.57952300
H	-3.76407300	-5.47257900	-1.30083900
H	-0.74509300	-1.86102500	1.84206700
C	-3.44522900	-1.60533900	-1.59995800
H	-3.93879400	-2.15477700	-2.40668400
C	1.31065300	2.05523800	-0.90864900
O	-2.69160200	-0.51374300	-2.12672300
H	-3.24485400	0.28797600	-2.18890900
H	-4.20900000	-1.24387100	-0.90051400
H	-2.40259000	2.73593100	-3.02424200
H	-3.50075900	3.94499700	-2.34078600
O	-4.02773900	1.94461900	-2.09473100
H	-4.51880200	2.06226200	-1.26598900
H	0.78909800	2.05498400	-1.86760900
H	2.18283300	2.71613400	-0.97339700
O	0.43946200	2.52968100	0.13049700
H	-0.68292300	-4.41798800	1.96660900
H	-0.16191300	0.03285600	0.18931400
H	1.77468200	-2.11580900	-0.76382500
O	3.95991100	-1.11389600	-0.02830500
C	5.23851900	-0.62499700	-0.21832700
C	5.67540400	0.61912100	0.23894500
C	6.10130800	-1.45934400	-0.93231900
C	6.99302000	1.00970400	-0.01005400
H	4.99939800	1.28617800	0.75800400
C	7.40695200	-1.05166200	-1.18018600
H	5.73523300	-2.41969300	-1.27806500
C	7.86213500	0.18422000	-0.71702300
H	7.33216100	1.97677400	0.34748500
H	8.07338600	-1.70556500	-1.73333400
H	8.88205100	0.49939700	-0.90916000
H	0.01897800	4.20789300	2.02341400
O	0.14936200	-2.74197300	0.19446900
H	-1.84700200	1.97334100	-0.68990800

H1			
C	5.97879900	0.69384600	0.54304600
C	4.90191200	0.38757900	1.59341200
C	3.60211100	-0.08016900	0.92734600
C	4.78624200	-0.86360800	-1.01256100
C	6.14692900	-0.47243700	-0.43998500
H	5.69060200	1.58234000	-0.02389600
H	4.37200100	-0.01359800	-1.56160600
H	6.56660400	-1.33676500	0.09949500
O	3.89871300	-1.19549400	0.07560400
C	1.66641700	1.07899600	-0.07446000
C	0.82969000	1.38212400	1.17845800
C	1.10379200	-0.11741300	-0.85814400
C	-0.61591400	1.62806300	0.74809100
H	1.22801600	2.27989500	1.65709800
C	-0.42800600	0.03330400	-1.05743900
H	-1.25233700	1.78230300	1.62361800
H	-0.60006900	0.76284600	-1.85449700
O	-1.11561100	0.46287100	0.12909400
C	-2.70089800	-1.86695000	2.59129500
C	-3.26443300	-1.58848400	1.20934200
C	-4.78489500	-1.50034700	1.12835500
C	-3.06665200	-2.43678100	-1.03335500
C	-5.20192700	-1.27561300	-0.33295300
H	-5.22724900	-2.44082500	1.49422200
C	-4.57585600	-2.31693900	-1.27136100
H	-2.67943200	-3.33307800	-1.53189300
H	-4.86768300	-0.27874100	-0.63124600
O	-2.82140200	-2.67267000	0.35318800
O	-5.21896000	-0.42282000	1.94611600
H	-6.15164200	-0.26229200	1.74665000
O	-6.62108900	-1.26819200	-0.44413600
H	-6.90886200	-2.18657800	-0.55632800
O	-5.20391400	-3.58963700	-1.08262300
H	-4.81463500	-4.01839100	-0.30781100
O	1.66853600	-0.20389400	-2.15578000
H	2.29093900	-0.94882700	-2.22500000
O	0.86854400	0.34373800	2.15409400
H	0.09158100	-0.24032400	2.10269100
O	5.38146200	-0.60649700	2.50589200
H	5.22397500	-1.48102600	2.12454900
O	7.22742100	0.99954500	1.15622000
H	7.32387100	0.41237500	1.92100700
O	7.00721300	-0.12226500	-1.51311700
H	7.78591800	0.30918400	-1.13499000
H	2.90363600	-0.46229200	1.66936400
C	4.81683500	-2.07745800	-1.92735800
H	5.34859600	-1.83626400	-2.84611300
C	-1.01213500	-1.32181400	-1.44194500
O	3.48481500	-2.46242400	-2.30229400
H	3.11014100	-2.96349100	-1.56850700
H	5.32514200	-2.91914000	-1.44363000
H	-3.07784200	-2.82126700	2.98041200
H	-3.00179000	-1.07484800	3.27544300
O	-1.26902000	-1.87416200	2.57557200
H	-0.99192500	-2.61095100	2.01689700
H	-0.71207400	-2.05918800	-0.69268100
H	-0.61177800	-1.61932200	-2.41490600
O	-2.44260600	-1.27779300	-1.50753400
H	4.70936800	1.27506600	2.19740200
H	1.29729300	-1.03579500	-0.29248400
H	1.60344500	1.95111100	-0.72801000
O	-0.64074200	2.76223700	-0.08752000
C	-1.83058100	3.43711300	-0.29153600
C	-3.08626000	2.93575600	0.05166600
C	-1.71031600	4.69336300	-0.88970600
C	-4.21912400	3.71513900	-0.19184900
H	-3.19708300	1.95012100	0.48378200
C	-2.84852000	5.45289300	-1.13456600
H	-0.72285300	5.05846200	-1.14951000
C	-4.11040500	4.97001500	-0.78313000
H	-5.19402700	3.32287500	0.07901700
H	-2.74771400	6.42920600	-1.59764100
H	-4.99721900	5.56516800	-0.97236900
H	-4.76912800	-2.04379900	-2.30945300
O	3.07432100	1.00549900	0.21511800
H	-2.82807200	-0.65955800	0.84380100

I1

C	1.89641800	4.67555600	0.06871600
C	1.29260800	3.98476500	1.29733400
C	0.86123200	2.55296100	0.95847100
C	2.47222400	2.40224100	-0.82445000
C	2.99641500	3.81532300	-0.56591400
H	1.11481700	4.84081800	-0.67674800
H	1.65452400	2.45471200	-1.55015700
H	3.84660800	3.75471900	0.13245100
O	1.97624900	1.84599200	0.40869000
C	-1.18457100	1.58058300	-0.02481500
C	-2.12469800	1.55522200	1.19269400
C	-0.61241800	0.18209400	-0.31137700
C	-3.23351200	0.52641900	0.95570500
H	-2.55532500	2.54652500	1.34193600
C	-1.77896500	-0.83212600	-0.38687100
H	-3.85472000	0.42526200	1.84920200
H	-2.36617100	-0.60331000	-1.28320500
O	-2.64230900	-0.74454900	0.76690400
C	2.59336300	-3.33904000	-2.41165400
C	2.01102100	-3.03526200	-1.02943500
C	3.05100700	-3.13316600	0.09698600
C	0.27066100	-3.81198900	0.41099500
C	2.39196400	-2.96577700	1.45915900
H	3.54468500	-4.11043000	0.05402200
C	1.20368700	-3.91815600	1.62191100
H	-0.46150600	-4.62528300	0.42299700
H	2.03332900	-1.93288200	1.54783900
O	0.95699600	-3.97573200	-0.81560200
O	4.03869900	-2.08967500	-0.01064100
H	4.50991800	-2.24153000	-0.84717200
O	3.30096300	-3.25530900	2.51984500
H	4.11794400	-2.76500900	2.36202000
O	1.63095200	-5.26590700	1.71602200
H	2.35573700	-5.29298000	2.35569600
O	0.04826000	0.12999500	-1.56572900
H	1.01075400	0.21002900	-1.45541400
O	-1.46179500	1.22267100	2.41380800
H	-1.37669600	0.25966900	2.46574600
O	2.23895900	3.98420900	2.37207100
H	2.83315500	3.22857500	2.26634100
O	2.41212800	5.96230500	0.39630200
H	2.82194600	5.89525300	1.27178400
O	3.43507300	4.36851700	-1.79733400
H	3.57502400	5.31547100	-1.65845400
H	0.59551400	2.01100600	1.86383700
C	3.53824900	1.45151000	-1.34279100
H	3.89373400	1.80489400	-2.31072700
C	-1.31593800	-2.27133600	-0.50588900
O	3.00258400	0.12969400	-1.51599900
H	3.27325600	-0.42791100	-0.76467100
H	4.38834500	1.41767900	-0.65352700
H	1.80821600	-3.24249600	-3.16845500
H	2.96762000	-4.36391200	-2.44169000
O	3.70944200	-2.49673800	-2.71485400
H	3.41002900	-1.57723800	-2.79957800
H	-0.87599700	-2.42009600	-1.49342200
H	-2.17925600	-2.93951100	-0.40157500
O	-0.35542600	-2.55306100	0.51195000
H	0.43524300	4.55323200	1.65961300
H	0.06654800	-0.12242600	0.48887900
H	-1.77958800	1.87222400	-0.89309500
O	-4.00912200	0.95530200	-0.14000700
C	-5.27554500	0.42962500	-0.32152000
C	-5.72105100	-0.75337000	0.26937900
C	-6.11350900	1.15740700	-1.16877700
C	-7.02405100	-1.18895300	0.01826200
H	-5.06224800	-1.34231700	0.89416700
C	-7.40450300	0.70494700	-1.41640800
H	-5.74137400	2.07216300	-1.61668000
C	-7.86920100	-0.46903100	-0.82072400
H	-7.36981100	-2.10844400	0.47963700
H	-8.05225000	1.27577500	-2.07388700
H	-8.87776300	-0.81860300	-1.01309500
H	0.63798500	-3.63149400	2.51671700
O	-0.21453700	2.63818700	0.06085500
H	1.59553000	-2.02202000	-1.03550200

J1

C	2.36427500	-4.05874400	-0.65435100
C	1.82343700	-3.02748300	-1.65277100
C	1.14940300	-1.85891600	-0.92311500
C	2.45441500	-2.24106300	1.06504800
C	3.22717300	-3.39624200	0.42905700
H	1.52631400	-4.55773100	-0.16127600
H	1.53884100	-2.63232600	1.51702500
H	4.14683900	-2.99959700	-0.03122300
O	2.08827100	-1.30345800	0.01831000
C	-1.12681900	-1.46474600	-0.05536100
C	-1.95248800	-1.27153600	-1.33429300
C	-0.75737100	-0.12622000	0.59941500
C	-3.18594600	-0.42702700	-1.00854600
H	-2.24878300	-2.24545800	-1.72705300
C	-1.95262300	0.86550600	0.61383100
H	-3.77712800	-0.24494500	-1.90982100
H	-2.58437500	0.60457500	1.46861400
O	-2.75214000	0.84849700	-0.58709500
C	2.83295400	2.95446300	2.26859500
C	1.98579100	2.68147700	1.02334100
C	2.84035800	2.59119600	-0.25490800
C	0.20670100	3.75430200	-0.18101300
C	2.00194000	2.65912700	-1.53024500
H	3.55325900	3.41922300	-0.27251900
C	1.01000100	3.82464900	-1.48339500
H	-0.41574600	4.64579900	-0.05879800
H	1.43513300	1.72575100	-1.63956700
O	1.04175400	3.75144300	0.95893600
O	3.65217100	1.40110300	-0.24632700
H	3.08316900	0.61266700	-0.17718500
O	2.82446400	2.86190500	-2.67697400
H	3.56353200	2.24078800	-2.64302200
O	1.67608900	5.07345000	-1.51676100
H	2.29650200	5.04941900	-2.25801500
O	-0.38914000	-0.27694000	1.95767200
H	0.57210700	-0.39473300	2.08185200
O	-1.22582500	-0.62168200	-2.37797100
H	-1.28694900	0.33557500	-2.24067600
O	2.88367700	-2.55100700	-2.48931400
H	3.39092000	-1.88573600	-2.00465700
O	3.10328400	-5.08119100	-1.31365400
H	3.60141800	-4.66300100	-2.03174100
O	3.57144800	-4.32910500	1.44037700
H	3.86472000	-5.13985300	1.00175300
H	0.93045200	-1.05778500	-1.62659000
C	3.21884300	-1.48071200	2.12661500
H	3.57716800	-2.18998300	2.87726400
C	-1.47387900	2.29873200	0.78064800
O	2.34135100	-0.52618400	2.72085900
H	2.82489800	0.30920300	2.86677000
H	4.09089700	-0.98406700	1.68315800
H	2.18268500	3.06066800	3.13699900
H	3.38781200	3.89063000	2.14253600
O	3.72906300	1.87665700	2.55849400
H	4.24760100	1.69467600	1.75965500
H	-0.97455700	2.40227200	1.74615100
H	-2.32653900	2.98588100	0.74180900
O	-0.56956100	2.58483200	-0.29022600
H	1.10968300	-3.49952700	-2.32852200
H	0.05093300	0.33364800	0.02833100
H	-1.73495700	-2.02714500	0.65451000
O	-3.94973500	-1.11423800	-0.04479900
C	-5.27249400	-0.76125600	0.15211400
C	-5.82800400	0.44855800	-0.26584000
C	-6.05147800	-1.70230600	0.82826800
C	-7.17980900	0.69663200	-0.01717000
H	-5.21930400	1.19835900	-0.75431800
C	-7.39305700	-1.43593400	1.07664800
H	-5.59351700	-2.63282500	1.14478000
C	-7.96663800	-0.23626400	0.65137700
H	-7.61128600	1.63736600	-0.34393800
H	-7.99423900	-2.17191700	1.60063500
H	-9.01429200	-0.03168600	0.84373200
H	0.31300200	3.73617300	-2.32558600
O	-0.00167700	-2.34067600	-0.29242100
H	1.45773100	1.73123000	1.16237000

J2

C	2.40966500	-3.95089300	-0.73852200
C	1.85713000	-2.88269100	-1.68582800
C	1.15686100	-1.77207700	-0.89751900
C	2.42276100	-2.23321200	1.09169400
C	3.23138900	-3.33435000	0.39580400
H	1.56131400	-4.49756600	-0.30145300
H	1.51643200	-2.67084900	1.53109800
H	4.14951100	-2.90974100	-0.01579400
O	2.02331200	-1.25085000	0.11652100
C	-1.14830500	-1.45686100	-0.08261000
C	-1.96944000	-1.24284000	-1.36133600
C	-0.79165400	-0.13166000	0.60511900
C	-3.20454800	-0.40492100	-1.02644400
H	-2.26380800	-2.21109400	-1.76937000
C	-1.97482400	0.87451400	0.61297300
H	-3.79555800	-0.21224300	-1.92559500
H	-2.61042800	0.62980400	1.46957600
O	-2.77569100	0.86501700	-0.58732500
C	2.80058300	2.88924800	2.30768800
C	1.98648600	2.62895000	1.03875900
C	2.87508800	2.53552800	-0.21561700
C	0.24325500	3.71105400	-0.20781600
C	2.05975200	2.57518800	-1.50675400
H	3.57088500	3.37804800	-0.22595100
C	1.06716000	3.74230500	-1.49940100
H	-0.36167200	4.61790200	-0.11285900
H	1.49506800	1.63941200	-1.60746300
O	1.05687500	3.71037700	0.94685700
O	3.70965000	1.36479800	-0.17147500
H	3.16190700	0.55810000	-0.19042200
O	2.89923500	2.75642300	-2.64353300
H	3.61147500	2.10435900	-2.61045600
O	1.73586500	4.98942600	-1.55619500
H	2.37833500	4.93829500	-2.27717500
O	-0.44799000	-0.31163100	1.96822500
H	0.51050600	-0.43452200	2.10157000
O	-1.23987700	-0.57988400	-2.39264100
H	-1.28440500	0.37485500	-2.23317000
O	2.90465000	-2.26775700	-2.42113900
H	3.45689100	-2.97689900	-2.77835100
O	3.20120700	-4.83495600	-1.52810000
H	3.74090500	-5.37334100	-0.93381700
O	3.67248500	-4.35645000	1.29646000
H	2.90864200	-4.73334000	1.75517500
H	0.95121500	-0.93733100	-1.56346200
C	3.17697600	-1.50849200	2.18921500
H	3.56335600	-2.23864400	2.90642500
C	-1.47069500	2.30142400	0.76504200
O	2.28860900	-0.60331200	2.83702600
H	2.74676100	0.25126400	2.95748500
H	4.03393300	-0.97650100	1.76022200
H	2.12810600	3.00647600	3.15785900
H	3.37497400	3.81578500	2.19810500
O	3.67051100	1.79578400	2.61764200
H	4.18465600	1.59485600	1.81892100
H	-0.97066500	2.40765200	1.73001800
H	-2.31038000	3.00384900	0.71712400
O	-0.55967900	2.55746900	-0.30781400
H	1.12808400	-3.33916600	-2.36499600
H	0.03052100	0.33378300	0.06174800
H	-1.75602000	-2.04000300	0.61095500
O	-3.96934400	-1.10748600	-0.07239900
C	-5.29042200	-0.75558900	0.13204300
C	-5.84667600	0.45916700	-0.27085300
C	-6.06884500	-1.70318800	0.80010400
C	-7.19745300	0.70560800	-0.01527500
H	-5.23874300	1.21382600	-0.75264300
C	-7.40940400	-1.43851900	1.05559500
H	-5.61079100	-2.63755900	1.10501800
C	-7.98329800	-0.23376600	0.64537500
H	-7.62896400	1.65032500	-0.33033700
H	-8.00966300	-2.17972900	1.57333300
H	-9.03015800	-0.03031800	0.84325900
H	0.38200500	3.63427600	-2.34905000
O	-0.01342400	-2.31214600	-0.34015300
H	1.44534500	1.68383900	1.16021600

K1

C	2.48860100	-3.97435300	-0.61987400
C	1.89761400	-2.99472000	-1.64161300
C	1.16162300	-1.84320600	-0.94468200
C	2.46153300	-2.13957600	1.07250000
C	3.31255600	-3.24827700	0.45377300
H	1.67658400	-4.50670800	-0.11789600
H	1.56481400	-2.58728400	1.51056000
H	4.20614200	-2.80009900	-0.01024000
O	2.05541800	-1.23681800	0.01097600
C	-1.13233600	-1.50242200	-0.09624200
C	-1.90699700	-1.19610600	-1.38472800
C	-0.79284500	-0.22831600	0.68925900
C	-3.15035900	-0.37500300	-1.03020600
H	-2.19096000	-2.12975200	-1.87293200
C	-1.95794500	0.79916200	0.70855500
H	-3.71736100	-0.12885900	-1.93175200
H	-2.62036200	0.52512500	1.53509900
O	-2.72779700	0.86739400	-0.50987500
C	3.30492100	2.59434300	1.92764800
C	2.25061600	2.43201200	0.84223100
C	2.89556900	2.46914900	-0.55382700
C	0.30948500	3.63937000	0.04898000
C	1.87140200	2.71086300	-1.66331200
H	3.63978500	3.26816300	-0.59961400
C	0.94224900	3.87676400	-1.32558400
H	-0.26314900	4.51553900	0.36755000
H	1.26419600	1.80627900	-1.79077100
O	1.28377900	3.47027100	1.06087100
O	3.61833300	1.25701800	-0.79241500
H	3.06168200	0.49442400	-0.55327100
O	2.51483300	3.03729000	-2.89282700
H	3.19943900	2.37772100	-3.06527600
O	1.64749500	5.10511800	-1.27652900
H	2.16278200	5.17265500	-2.09191500
O	-0.52236000	-0.51109500	2.04861700
H	0.43617500	-0.52886500	2.23661400
O	-1.14887500	-0.45573700	-2.34259300
H	-1.20884900	0.48383800	-2.11201600
O	2.93295000	-2.49151400	-2.49343900
H	3.41658200	-1.79214700	-2.03310900
O	3.28172300	-4.97010600	-1.25699700
H	3.75460700	-4.54195500	-1.98634100
O	3.71145600	-4.14782000	1.47489100
H	4.04543600	-4.94722600	1.04451100

H	0.93272400	-1.05707300	-1.66178500
C	3.14714700	-1.32200900	2.14791100
H	3.60746500	-2.00271400	2.86887200
C	-1.42157900	2.20168300	0.95007100
O	2.15139300	-0.52024200	2.78410600
H	2.50271900	0.33432700	3.09419800
H	3.93382300	-0.69791100	1.70770300
H	3.62277600	3.64097800	1.97019900
H	4.17240400	1.97728100	1.69507500
O	2.85072700	2.16065200	3.21502000
H	2.12270000	2.72746300	3.49921400
H	-0.90970700	2.22611400	1.91540300
H	-2.24317700	2.92672800	0.95861900
O	-0.51572700	2.51088000	-0.11295600
H	1.20943200	-3.52035000	-2.30425300
H	0.06536400	0.25015800	0.21746400
H	-1.77018500	-2.12161900	0.53554300
O	-3.93932500	-1.12577700	-0.13695600
C	-5.26257000	-0.77647300	0.06060400
C	-5.80641400	0.45800400	-0.29631800
C	-6.05591600	-1.74955500	0.67187300
C	-7.16055000	0.69912400	-0.05349200
H	-5.18696400	1.23122400	-0.73176100
C	-7.39959600	-1.49005400	0.91586200
H	-5.60684600	-2.69863300	0.94270600
C	-7.96145300	-0.26533200	0.55042300
H	-7.58273100	1.65910200	-0.33307500
H	-8.01162000	-2.25077400	1.38959600
H	-9.01079800	-0.06606600	0.73917700
H	0.13784200	3.92392900	-2.06945200
O	0.00793700	-2.35858200	-0.34370400
H	1.76298800	1.45908700	0.96896300

K2

C	2.48333700	-3.91162100	-0.71778400
C	1.88889900	-2.88618400	-1.68625600
C	1.13840900	-1.78837500	-0.92666100
C	2.40359700	-2.17806600	1.08544800
C	3.27436300	-3.24437700	0.41069100
H	1.65708200	-4.48565900	-0.27299100
H	1.51597900	-2.66153700	1.51611000
H	4.17427300	-2.78252400	-0.00085600
O	1.97170100	-1.22961300	0.09392200
C	-1.17256800	-1.50021100	-0.12387600
C	-1.93987200	-1.15430500	-1.40710700
C	-0.82773700	-0.25086600	0.69839900
C	-3.17450200	-0.32714000	-1.03894800
H	-2.23336000	-2.07407300	-1.91564300
C	-1.96886700	0.80297300	0.72348400
H	-3.73643500	-0.05598600	-1.93641300
H	-2.63598900	0.54529400	1.55148900
O	-2.74164700	0.89875100	-0.49142500
C	3.32504900	2.46766500	1.98341400
C	2.31253000	2.35038300	0.85526500
C	2.99608400	2.47098700	-0.51543800
C	0.37484700	3.58024900	0.07095600
C	1.97866400	2.68716100	-1.63502100
H	3.68421100	3.31992400	-0.51107200
C	1.01444200	3.82607900	-1.29939300
H	-0.18135400	4.46517600	0.39477200
H	1.39878300	1.76541600	-1.77494300
O	1.33843600	3.38206300	1.08828700
O	3.81175300	1.32982100	-0.78287900
H	3.28344600	0.51619900	-0.71970400
O	2.62678100	3.03855800	-2.85407200
H	3.34878400	2.41342900	-3.00374000
O	1.68780500	5.07258200	-1.24663300
H	2.22398600	5.14079300	-2.04864100
O	-0.58012000	-0.57256200	2.05547800
H	0.37524300	-0.60867800	2.25448900
O	-1.17178600	-0.40519800	-2.34828800
H	-1.20247100	0.52849200	-2.08954000
O	2.91367800	-2.23856400	-2.42661800
H	3.50400100	-2.92717000	-2.76244000
O	3.31376000	-4.77574600	-1.48942400
H	3.87581800	-5.27750800	-0.88390100
O	3.75680800	-4.23554000	1.32491800
H	3.00865300	-4.63972100	1.78636000
H	0.92493000	-0.96599100	-1.60532400
C	3.08926300	-1.39741000	2.19122700
H	3.59067400	-2.09062800	2.87297400
C	-1.39176900	2.18964200	0.96302300
O	2.09194200	-0.65436800	2.89049000
H	2.41675900	0.22787900	3.15174100
H	3.84712200	-0.73462200	1.75935200
H	3.66639600	3.50493600	2.06061400
H	4.18623000	1.83359200	1.77655100
O	2.80160600	2.02702800	3.24313300
H	2.06901200	2.60390700	3.49370900
H	-0.87924800	2.20088900	1.92837400
H	-2.19117200	2.93918300	0.97033100
O	-0.47611300	2.47198000	-0.09986200
H	1.18670200	-3.38845400	-2.36139200
H	0.04649900	0.22224300	0.25483300
H	-1.81818800	-2.13235000	0.48728400
O	-3.97670000	-1.08793800	-0.16367700
C	-5.29639100	-0.72781000	0.03270900
C	-5.82216400	0.52256400	-0.29567200
C	-6.10758000	-1.70615000	0.61173200
C	-7.17501700	0.77462100	-0.05726500
H	-5.18906200	1.29870700	-0.70553500
C	-7.44987500	-1.43595100	0.85191600
H	-5.67291500	-2.66788100	0.86067200
C	-7.99323700	-0.19495200	0.51453400
H	-7.58262600	1.74702900	-0.31472600
H	-8.07547900	-2.20104700	1.30029700
H	-9.04155000	0.01275900	0.70001900
H	0.21433200	3.85406400	-2.04881000
O	-0.03574300	-2.35013300	-0.39550100
H	1.82421800	1.37150900	0.91663500

L1

C	5.85471900	0.69632100	0.63742400
C	4.74791000	0.36977900	1.65011200
C	3.46857900	-0.08951300	0.93887300
C	4.71291200	-0.83182800	-0.98109500
C	6.05524300	-0.45191700	-0.36005200
H	5.58054100	1.59373500	0.07765500
H	4.31480100	0.02769300	-1.52717400
H	6.45835500	-1.32595400	0.17647500
O	3.79330400	-1.18444300	0.07329400
C	1.56036800	1.08498700	-0.10647500
C	0.65291100	1.28784000	1.11381700
C	1.04869000	-0.07398600	-0.97743700
C	-0.76155100	1.58350300	0.60571700
H	1.01040700	2.14725900	1.69301500
C	-0.46950200	0.07508100	-1.27076000
H	-1.46149300	1.68205700	1.44181700
H	-0.58527200	0.80543300	-2.07760300
O	-1.24861800	0.50593900	-0.14567800
C	-2.06844600	-2.21371700	2.33427700
C	-2.84068600	-1.88468300	1.05990300
C	-4.34958600	-1.76785700	1.23509600
C	-3.02020600	-2.51234200	-1.23369500
C	-4.99548900	-1.39489700	-0.10392700
H	-4.75448900	-2.72997400	1.58468400
C	-4.54576800	-2.34858300	-1.22467900
H	-2.73114000	-3.35268000	-1.87557600
H	-4.69237800	-0.37684000	-0.35945500
O	-2.57757500	-2.89956800	0.06789600
O	-4.59419500	-0.77053600	2.22862800
H	-5.54246600	-0.58385300	2.24595400
O	-6.41364900	-1.37086300	0.01709700
H	-6.73322900	-2.27166900	-0.14267200
O	-5.17570500	-3.62349100	-1.06143200
H	-4.66712400	-4.14414500	-0.42409700
O	1.69275800	-0.09904600	-2.24166200
H	2.28714300	-0.86564600	-2.31718300
O	0.65292700	0.11086900	1.91578000
H	-0.19049900	-0.02695600	2.38367300
O	5.20274500	-0.63918400	2.55933800
H	5.05559900	-1.50727900	2.15949300
O	7.08381200	0.99521600	1.29270600
H	7.16142100	0.39258700	2.04753400
O	6.94911000	-0.08143300	-1.39837600
H	7.71300100	0.34609700	-0.98701200

H	2.75282100	-0.49428100	1.65181900
C	4.77141700	-2.02941400	-1.91562300
H	5.33827400	-1.77541600	-2.80965800
C	-1.02198300	-1.29166400	-1.69692600
O	3.45320100	-2.40066100	-2.34730500
H	3.04105300	-2.89519600	-1.62898800
H	5.25710200	-2.88203800	-1.42765200
H	-1.08688800	-2.60585700	2.06692100
H	-2.58251800	-2.96468600	2.94615900
O	-1.83342300	-1.02551600	3.09815400
H	-2.69434200	-0.63135400	3.30224700
H	-0.65117300	-2.05426400	-1.00849700
H	-0.66784600	-1.52317900	-2.70519900
O	-2.45565300	-1.30764200	-1.67507200
H	4.53627100	1.24666900	2.26322800
H	1.21478900	-1.01133400	-0.43991400
H	1.51410900	1.99600800	-0.70476300
O	-0.70651200	2.80055900	-0.11256000
C	-1.86588400	3.52526800	-0.31062400
C	-3.14868500	2.98234700	-0.22750600
C	-1.68875300	4.87400100	-0.62687600
C	-4.25288300	3.80884200	-0.44537100
H	-3.28960800	1.92885800	-0.02464600
C	-2.79792000	5.68182900	-0.85077500
H	-0.68138100	5.27072500	-0.68794200
C	-4.08736500	5.15514200	-0.75683000
H	-5.24964700	3.38409700	-0.38119500
H	-2.65347100	6.72945200	-1.09461700
H	-4.95144600	5.78757200	-0.92979200
H	-4.88715600	-1.97000900	-2.18894700
O	2.95561600	1.01238800	0.23977000
H	-2.45552300	-0.93357900	0.69785700

M1

C	5.97879900	0.69384600	0.54304600
C	4.90191200	0.38757900	1.59341200
C	3.60211100	-0.08016900	0.92734600
C	4.78624200	-0.86360800	-1.01256100
C	6.14692900	-0.47243700	-0.43998500
H	5.69060200	1.58234000	-0.02389600
H	4.37200100	-0.01359800	-1.56160600
H	6.56660400	-1.33676500	0.09949500
O	3.89871300	-1.19549400	0.07560400
C	1.66641700	1.07899600	-0.07446000
C	0.82969000	1.38212400	1.17845800
C	1.10379200	-0.11741300	-0.85814400
C	-0.61591400	1.62806300	0.74809100
H	1.22801600	2.27989500	1.65709800
C	-0.42800600	0.03330400	-1.05743900
H	-1.25233700	1.78230300	1.62361800
H	-0.60006900	0.76284600	-1.85449700
O	-1.11561100	0.46287100	0.12909400
C	-2.70089800	-1.86695000	2.59129500
C	-3.26443300	-1.58848400	1.20934200
C	-4.78489500	-1.50034700	1.12835500
C	-3.06665200	-2.43678100	-1.03335500
C	-5.20192700	-1.27561300	-0.33295300
H	-5.22724900	-2.44082500	1.49422200
C	-4.57585600	-2.31693900	-1.27136100
H	-2.67943200	-3.33307800	-1.53189300
H	-4.86768300	-0.27874100	-0.63124600
O	-2.82140200	-2.67267000	0.35318800
O	-5.21896000	-0.42282000	1.94611600
H	-6.15164200	-0.26229200	1.74665000
O	-6.62108900	-1.26819200	-0.44413600
H	-6.90886200	-2.18657800	-0.55632800
O	-5.20391400	-3.58963700	-1.08262300
H	-4.81463500	-4.01839100	-0.30781100
O	1.66853600	-0.20389400	-2.15578000
H	2.29093900	-0.94882700	-2.22500000
O	0.86854400	0.34373800	2.15409400
H	0.09158100	-0.24032400	2.10269100
O	5.38146200	-0.60649700	2.50589200
H	5.22397500	-1.48102600	2.12454900
O	7.22742100	0.99954500	1.15622000
H	7.32387100	0.41237500	1.92100700
O	7.00721300	-0.12226500	-1.51311700
H	7.78591800	0.30918400	-1.13499000
H	2.90363600	-0.46229200	1.66936400
C	4.81683500	-2.07745800	-1.92735800
H	5.34859600	-1.83626400	-2.84611300
C	-1.01213500	-1.32181400	-1.44194500
O	3.48481500	-2.46242400	-2.30229400
H	3.11014100	-2.96349100	-1.56850700
H	5.32514200	-2.91914000	-1.44363000
H	-3.07784200	-2.82126700	2.98041200
H	-3.00179000	-1.07484800	3.27544300
O	-1.26902000	-1.87416200	2.57557200
H	-0.99192500	-2.61095100	2.01689700
H	-0.71207400	-2.05918800	-0.69268100
H	-0.61177800	-1.61932200	-2.41490600
O	-2.44260600	-1.27779300	-1.50753400
H	4.70936800	1.27506600	2.19740200
H	1.29729300	-1.03579500	-0.29248400
H	1.60344500	1.95111100	-0.72801000
O	-0.64074200	2.76223700	-0.08752000
C	-1.83058100	3.43711300	-0.29153600
C	-3.08626000	2.93575600	0.05166600
C	-1.71031600	4.69336300	-0.88970600
C	-4.21912400	3.71513900	-0.19184900
H	-3.19708300	1.95012100	0.48378200
C	-2.84852000	5.45289300	-1.13456600
H	-0.72285300	5.05846200	-1.14951000
C	-4.11040500	4.97001500	-0.78313000
H	-5.19402700	3.32287500	0.07901700
H	-2.74771400	6.42920600	-1.59764100
H	-4.99721900	5.56516800	-0.97236900
H	-4.76912800	-2.04379900	-2.30945300
O	3.07432100	1.00549900	0.21511800
H	-2.82807200	-0.65955800	0.84380100

Table S4. Cartesian coordinates of optimized configurations for core pentasaccharide

A1

C	0.22057700	−0.61295200	1.91446000
C	1.64330400	−0.03943100	1.89524300
C	2.63107600	−1.15486100	2.27124100
C	1.18429800	−2.84649500	1.40104900
C	0.16791900	−1.79931100	0.95715400
H	1.84847800	0.28026300	0.88045900
H	−0.02063100	−0.97112500	2.90829500
H	0.91372600	−3.20228500	2.39477500
H	2.46532300	−1.43271000	3.30732900
O	2.46577600	−2.25471000	1.44129300
O	−0.73038600	0.33067600	1.51863700
C	1.29184800	−4.04178500	0.46817100
H	0.32757300	−4.52818100	0.40589800
H	1.99259400	−4.75259700	0.90019200
H	−0.88097100	0.88813000	2.27891000
O	1.67090900	−3.68070600	−0.82092100
H	2.59186300	−3.43783800	−0.81005500
C	5.21680100	0.85409200	1.02957800
C	6.15703000	1.06324400	−0.16678600
C	4.62241400	−0.56027100	0.99891200
H	5.77584100	0.95903300	1.95471100
C	7.17665900	−0.07207900	−0.22554700
H	5.55953300	1.00554200	−1.07050600
C	5.73758900	−1.60173700	0.85835700
H	3.94197700	−0.64812600	0.16402800
H	7.83497300	−0.03013300	0.63966700
H	6.34926700	−1.56935300	1.75913700
O	6.52877900	−1.29425700	−0.27198200
O	7.90713900	0.09178100	−1.38584300
O	3.96608800	−0.77160900	2.22746900
C	5.28050800	−3.03303000	0.63795400
H	6.15839600	−3.67295600	0.64931800
H	4.61532300	−3.33977200	1.42513800
O	4.59224400	−3.17908400	−0.57424700
H	5.16824000	−2.92630700	−1.28468600
O	4.15286200	1.75301600	1.00210000
H	4.51394800	2.61412600	1.20847500
C	9.11670600	−0.52304900	−1.54964300
C	9.55938600	−1.58346200	−0.77814000
C	9.91068900	−0.01658400	−2.56928400
C	10.81541700	−2.12170400	−1.02659100
H	8.93770400	−2.00600200	−0.01450300
C	11.15382800	−0.56803000	−2.80966400
H	9.53946000	0.79897000	−3.16225400
C	11.61741700	−1.62257200	−2.03513300
H	11.15706000	−2.94521100	−0.42551200
H	11.76346700	−0.17178900	−3.60171400
H	12.58574500	−2.04944000	−2.22161300
H	0.39107900	−1.48110400	−0.04624700
N	6.91310900	2.31380500	−0.18805900
H	7.57971900	2.33569000	−0.92652200
C	7.77721700	4.53957700	0.17753500
H	7.23009200	5.30473800	−0.36238000
H	8.15437900	4.98327300	1.08868800
H	8.60608600	4.20410100	−0.43284800
N	1.88221200	1.10760500	2.75573600
H	2.74900100	1.55012100	2.54546100
C	1.69130600	2.90344100	4.35544600
H	1.14342200	3.78215700	4.03448600
H	1.54545900	2.79660300	5.42252000
H	2.74376100	3.04717000	4.14667800
C	1.09391700	1.69397000	3.66840800
O	−0.02792800	1.33255900	3.95097200
C	6.81071500	3.43588300	0.54769500
O	6.01365600	3.60717500	1.44181400
C	−8.44955200	−1.21122100	−0.33538800
C	−7.79909100	−2.53596600	0.04774400
C	−6.34131700	−2.32009300	0.44936700
C	−6.70151000	−0.04186900	0.97882000
C	−8.20088700	−0.14094900	0.72157300
H	−8.01994500	−0.86770000	−1.27455100
H	−6.21118200	0.25241100	0.06047400
H	−8.70848100	−0.42440100	1.63446500
O	−6.21624300	−1.30758900	1.38553000
C	−4.26327800	−2.35244100	−0.82866400
C	−3.35577200	−2.07199200	0.36581600
C	−3.79074200	−1.54946900	−2.03063900
C	−1.94857400	−2.47847000	−0.07527100
H	−3.63971400	−2.67049000	1.22474500
C	−2.27915000	−1.60774600	−2.26859000
H	−2.06662100	−2.49649200	−2.85749300
O	−1.52159900	−1.63708000	−1.08360900
C	−5.78361300	2.54968400	−2.30671900
C	−4.36294100	2.30983100	−1.80719000
C	−3.83139600	3.44621500	−0.93472700
C	−2.20616200	1.97241600	−2.79020700
C	−2.36228000	3.21513400	−0.61588800

H	-3.92099200	4.37791800	-1.47762100
C	-1.56888700	3.03969400	-1.90471000
H	-1.78092900	2.03493100	-3.78273200
H	-2.25724700	2.31737700	-0.01374900
O	-3.57223400	2.17912700	-2.96761600
O	-4.58432000	3.61406800	0.24434700
H	-4.56483900	2.82524000	0.79083800
O	-1.82109200	4.32180900	0.06059200
H	-2.43493100	4.58742300	0.73470400
O	-1.54946200	4.23178400	-2.63735300
H	-1.33499300	4.93388400	-2.03523800
O	-4.38537400	-2.00975600	-3.21529200
H	-5.32450600	-1.90478300	-3.13840700
O	-3.35929300	-0.71418600	0.69796800
H	-2.46889300	-0.42398400	0.89827500
O	-8.44544300	-3.10375500	1.14834600
H	-9.38087900	-3.04186300	0.99724300
O	-9.82279200	-1.44925000	-0.49646200
H	-10.26563000	-0.60897900	-0.51493700
O	-8.76821400	1.07498500	0.31054200
H	-8.22351400	1.48698600	-0.35630600
H	-5.96884800	-3.21664900	0.92480600
C	-6.30507200	0.95491900	2.05058400
H	-6.87266900	1.86700500	1.93594600
C	-1.82276300	-0.37822900	-3.03865300
O	-4.94492600	1.29627600	1.94332300
H	-4.44055500	0.50417500	1.76789200
H	-6.51969100	0.53777900	3.02924200
H	-6.06306800	1.74661900	-2.97458200
H	-5.81696400	3.47754500	-2.86991800
O	-6.72483400	2.56561700	-1.26114800
H	-6.43664500	3.19463400	-0.60788500
H	-2.43435500	-0.27674700	-3.92733800
H	-0.78463800	-0.49451400	-3.33216400
O	-1.94634300	0.74556600	-2.20465300
H	-7.82818700	-3.21309600	-0.80127700
H	-4.07132500	-0.52649400	-1.84444400
H	-4.19212900	-3.41527500	-1.05233200
O	-1.12356800	-2.35251900	1.03581300
H	-0.55949500	2.72178800	-1.66541100
O	-5.62917000	-2.02183700	-0.70756100
H	-4.34011200	1.39464700	-1.22776600
H	-1.93281500	-3.51237900	-0.41603500
A2			
C	0.21496600	-0.48220800	-1.30992700
C	1.47131600	-0.98963800	-0.58389700
C	2.61510300	-0.00029700	-0.80506600
C	1.14765800	1.85257200	-1.12026800
C	-0.09081800	0.97445100	-0.96197400
H	1.27705000	-1.04338800	0.48133200
H	0.39644700	-0.54623800	-2.38043200
H	1.40452200	1.91282600	-2.17642500
H	2.87927400	0.02913400	-1.86101100
O	2.22679400	1.27751100	-0.40126100
O	-0.92867900	-1.22494500	-0.99582600
C	0.99730200	3.26190300	-0.56676300
H	0.14221800	3.74297500	-1.01563400
H	1.88389900	3.83306900	-0.83691500
H	-0.70655100	-2.07303800	-0.60994500
O	0.80098500	3.27318400	0.81383600
H	1.51537200	2.80774600	1.22849800
C	5.57015500	1.17553800	-0.25651700
C	7.03803100	1.24792600	-0.69634300
C	4.99857800	-0.20078600	-0.57569300
H	5.49828000	1.33768600	0.81208100
C	7.85091500	0.02607800	-0.25734200
H	7.04556400	1.23343400	-1.77890700
C	5.89853300	-1.27459200	0.02412700
H	4.97732700	-0.33327000	-1.65086300
H	8.04646900	0.04487600	0.80559400
H	5.98397000	-1.12532900	1.09945100
O	7.15920300	-1.14834900	-0.58670100
O	9.02650300	0.04608900	-0.97441000
O	3.70011500	-0.38138900	-0.05284800
C	5.40748500	-2.68842000	-0.21984300
H	6.17340100	-3.38564000	0.10332800
H	4.51606700	-2.86576000	0.36116200
O	5.06467900	-2.91380000	-1.56427400
H	5.84440400	-2.83512300	-2.10021800
O	4.90041000	2.18701500	-0.95542800
H	4.01083800	2.24454500	-0.62804900
C	10.12630900	-0.62326900	-0.52878200
C	10.08060600	-1.69025400	0.35258600
C	11.34325400	-0.18255300	-1.03118400
C	11.26598700	-2.30066500	0.74040000
H	9.14276300	-2.05714900	0.71951900
C	12.51316600	-0.80623700	-0.64467600
H	11.35067900	0.64524500	-1.71566400
C	12.48311600	-1.86829500	0.24883100
H	11.22645700	-3.12674800	1.42787200

H	13.45212400	-0.45872800	-1.03722400
H	13.39471400	-2.34996400	0.55222300
H	-0.45142300	1.03157700	0.05150100
N	7.67114700	2.48885300	-0.30211000
H	7.83037800	3.15476800	-1.02121200
C	8.36483300	4.29261500	1.15858400
H	7.53947400	4.99459300	1.08907500
H	8.80790100	4.37693500	2.14000900
H	9.09728500	4.55207900	0.40285400
N	1.90726000	-2.29681100	-1.06365800
H	2.81439400	-2.36140700	-1.47404000
C	2.01511700	-4.72949700	-1.09697700
H	2.11196900	-5.36059700	-0.22241200
H	1.37318900	-5.24580000	-1.80150100
H	2.98654400	-4.57093900	-1.54376500
C	1.32561700	-3.45187600	-0.68304500
O	0.28760900	-3.48031700	-0.06152900
C	7.83467200	2.88713900	0.98110200
O	7.56673500	2.18225000	1.91923100
C	-8.56571400	0.06908400	-1.67187600
C	-7.80365300	0.77973700	-2.78546600
C	-6.29709500	0.62874600	-2.58302700
C	-6.55207900	-1.32029000	-1.26721300
C	-8.06170900	-1.35591100	-1.47188600
H	-8.41897800	0.61504200	-0.74191100
H	-6.33702500	-0.74879000	-0.37439600
H	-8.29650600	-1.93780600	-2.35374000
O	-5.94353100	-0.69474200	-2.38177800
C	-4.67462600	2.01847700	-1.39596100
C	-3.45612800	1.16194900	-1.72899000
C	-4.59559400	2.48426500	0.04990200
C	-2.24082800	2.06630000	-1.49897700
H	-3.46282700	0.86412900	-2.77381800
C	-3.23511000	3.05790900	0.45188000
H	-3.23755800	4.11707500	0.20785400
O	-2.14025600	2.43871800	-0.17848600
C	-6.57222600	-0.44964600	2.91966500
C	-5.06823300	-0.34131000	2.69075600
C	-4.30337600	-1.60036900	3.09871500
C	-3.29092000	1.06697200	3.46812400
C	-2.80557100	-1.35231600	2.99728000
H	-4.53991600	-1.83924800	4.12692900
C	-2.42360100	-0.13451600	3.83039700
H	-3.17522300	1.82808000	4.22756600
H	-2.53888500	-1.17146700	1.95876600
O	-4.64896000	0.75792600	3.46899500
O	-4.68807000	-2.72629200	2.34498900
H	-4.52281200	-2.60193500	1.40842100
O	-2.07872400	-2.43955200	3.50605500
H	-2.41402200	-3.23811300	3.11705800
O	-2.61725800	-0.39123500	5.19164000
H	-2.22944800	-1.23657000	5.38304700
O	-5.52930900	3.49972700	0.30538400
H	-6.39844200	3.14937500	0.16167000
O	-3.40726500	0.04796400	-0.89477700
H	-2.53841900	-0.35276300	-0.93671300
O	-8.10676200	0.21892100	-4.02833300
H	-9.04888900	0.10882500	-4.07353700
O	-9.92395400	0.07773900	-2.02251800
H	-10.37696500	-0.53773500	-1.45816800
O	-8.74292900	-1.95773000	-0.40275500
H	-8.39631400	-1.64649200	0.43040000
H	-5.78423800	0.94459900	-3.48071100
C	-5.89862300	-2.67866600	-1.11008000
H	-6.47982200	-3.29646200	-0.44116600
C	-3.00787800	2.89222000	1.94613500
O	-4.61409500	-2.55060200	-0.55122100
H	-4.19255100	-1.78566600	-0.93365600
H	-5.84820900	-3.17080200	-2.07610600
H	-7.03292700	0.50070500	2.68794100
H	-6.76437100	-0.66581200	3.96629700
O	-7.17451000	-1.41450100	2.09124300
H	-6.71433000	-2.23895300	2.20934100
H	-3.85184300	3.31677200	2.47697700
H	-2.10034000	3.41038500	2.23379200
O	-2.86802100	1.52159700	2.23032900
H	-8.05051500	1.83767800	-2.77517700
H	-4.80311000	1.62301900	0.66175600
H	-4.66148900	2.88357400	-2.05612700
O	-1.06677900	1.41669100	-1.88225100
H	-1.38808800	0.12581900	3.63597300
O	-5.94372700	1.41262800	-1.48946000
H	-4.88188300	-0.15437600	1.63990800
H	-2.32316200	2.94156800	-2.14141100

A3

C	0.37401300	-1.13591000	1.76533400
C	1.78543700	-0.54411100	1.87268000
C	2.80343100	-1.69406000	1.92213000
C	1.36848400	-3.13351800	0.66652600
C	0.31997700	-2.03391300	0.53329800

H	1.96060200	0.03739100	0.97529900	H	7.61118400	2.63847700	-0.31881400
H	0.16229800	-1.74702700	2.63461500	C	7.79017600	4.47700600	1.32512100
H	1.13167900	-3.74436900	1.53715500	H	7.21941800	5.34524800	1.01414800
H	2.66878800	-2.23960700	2.85080400	H	8.17665200	4.67274200	2.31590100
O	2.63839000	-2.53966200	0.83431100	H	8.61311300	4.33288800	0.63655900
O	-0.60165400	-0.14301500	1.64754700	N	2.02244800	0.34032700	3.00179400
C	1.47382300	-4.03833800	-0.55029800	H	2.87753500	0.84073800	2.90371200
H	0.51839600	-4.51758200	-0.71675800	C	1.84880600	1.61156900	5.04623000
H	2.20167700	-4.81785100	-0.33720500	H	1.20119300	2.47797000	5.10626100
H	-0.75093800	0.18516300	2.53136200	H	1.85862000	1.14251000	6.02242100
O	1.80727500	-3.34007200	-1.70687400	H	2.85151800	1.93094600	4.79258600
H	2.72298300	-3.08320600	-1.65352100	C	1.24888600	0.63537800	4.05666300
C	5.31887600	0.63629800	1.21081900	O	0.14054400	0.18557600	4.25145100
C	6.22510600	1.18009600	0.09624700	C	6.85058300	3.29373900	1.40362400
C	4.75001700	-0.73294800	0.81537400	O	6.06705800	3.20595800	2.32154800
H	5.89876600	0.50478000	2.11964300	C	-8.26078800	-1.20134500	-0.43598800
C	7.26232100	0.12599800	-0.28493000	C	-7.69125900	-2.60951000	-0.30982100
H	5.60625100	1.35291100	-0.77794900	C	-6.22619100	-2.53812800	0.11259400
C	5.87958700	-1.67171700	0.37876800	C	-6.49523300	-0.42838100	1.16283800
H	4.04997500	-0.61148500	0.00117400	C	-7.97874800	-0.37020900	0.81021200
H	7.94152200	-0.04960500	0.54668000	H	-7.79357500	-0.71082000	-1.28711400
H	6.51424100	-1.86607100	1.24251000	H	-5.93261700	0.07388000	0.38563600
O	6.63469600	-1.05409900	-0.64417400	H	-8.56471600	-0.76482200	1.62983100
O	7.96009100	0.61143300	-1.37304700	O	-6.07205300	-1.77379600	1.25619300
O	4.12932100	-1.27960500	1.95593700	C	-4.16343900	-2.22575000	-1.20071600
C	5.44280400	-3.00297000	-0.20698500	C	-3.22231500	-2.27160800	0.00076600
H	6.33224500	-3.60040500	-0.38657700	C	-3.76805500	-1.07668300	-2.11573800
H	4.80546700	-3.52553400	0.48360200	C	-1.81871600	-2.45152200	-0.58251000
O	4.72392300	-2.83693000	-1.39873700	H	-3.44697700	-3.11764900	0.64169600
H	5.27589900	-2.39082200	-2.02858300	C	-2.26616700	-0.96975600	-2.40066200
O	4.23759500	1.48315000	1.44449100	H	-2.02518100	-1.61540400	-3.24080900
H	4.58794400	2.26857700	1.86288700	O	-1.46842700	-1.31904000	-1.29335200
C	9.17547700	0.09117700	-1.71995200	C	-5.38016800	3.67583300	-3.21204700
C	9.65615700	-1.12599200	-1.26935100	C	-4.58238500	2.88237700	-2.18963400
C	9.93390700	0.87008500	-2.58287200	C	-4.87530100	3.29502900	-0.75030900
C	10.91444500	-1.54863600	-1.67820200	C	-2.24575400	2.61170200	-1.65986500
H	9.06188200	-1.75161500	-0.63405000	C	-3.87367100	2.67387400	0.20820000
C	11.17974100	0.43226800	-2.98720200	H	-4.81772500	4.37281900	-0.67897800
H	9.53344300	1.80538300	-2.92841100	C	-2.45567700	3.04083600	-0.21210600
C	11.68132900	-0.77958800	-2.53256000	H	-1.30854800	2.99193000	-2.04622500
H	11.28570200	-2.49444400	-1.32636800	H	-3.97624500	1.59912400	0.18221900
H	11.76175900	1.03984400	-3.65660700	O	-3.22333300	3.12293300	-2.50977100
H	12.65172800	-1.11823600	-2.84606000	O	-6.18461100	2.87028900	-0.42608700
H	0.50982900	-1.46084300	-0.35753400	H	-6.37614400	3.21720400	0.43676300
N	6.95995200	2.40717200	0.39715500	O	-4.14572200	3.14617400	1.50588200

H	-4.22998200	2.39471400	2.08787300
O	-2.24292600	4.42171900	-0.12698000
H	-2.53012100	4.70358600	0.73318000
O	-4.39905100	-1.17229300	-3.36553100
H	-5.33644800	-1.19471200	-3.22435800
O	-3.26675600	-1.08552100	0.73775400
H	-2.37900000	-0.77550300	0.91634900
O	-8.37274700	-3.33685200	0.66758800
H	-9.30338000	-3.22076000	0.51939900
O	-9.64139000	-1.32514300	-0.65373300
H	-10.03964400	-0.47077000	-0.53790900
O	-8.42054200	0.95061500	0.61049000
H	-7.83618500	1.40115300	0.00800900
H	-5.86764200	-3.52685100	0.36256100
C	-6.16583000	0.23055200	2.49031700
H	-6.68542200	1.17517600	2.56802400
C	-1.90953300	0.47195400	-2.73852300
O	-4.79372000	0.50177900	2.61132900
H	-4.30072200	-0.22664200	2.24108400
H	-6.50259500	-0.41315800	3.29711500
H	-6.43722100	3.56963900	-3.01326200
H	-5.17346300	3.27077300	-4.20014500
O	-5.08351000	5.03985200	-3.16151200
H	-4.14038800	5.13547900	-3.20971400
H	-2.46082700	0.78885000	-3.61551100
H	-0.84507100	0.55382700	-2.93614400
O	-2.25609800	1.23095200	-1.61468500
H	-7.75240800	-3.11098700	-1.27148700
H	-4.08082900	-0.18281300	-1.60396900
H	-4.06940600	-3.17225100	-1.72923300
O	-0.95564600	-2.62637500	0.49110700
H	-1.74059500	2.50321100	0.40193000
O	-5.53633200	-1.99364800	-0.96451500
H	-4.81082200	1.82825600	-2.30937600
H	-1.77419600	-3.32795300	-1.22682100

A4

C	-0.37887700	-0.37011700	-0.31247300
C	-1.63139200	0.36733700	0.17784200
C	-2.82596700	-0.56241500	-0.02935100
C	-1.44753600	-2.51680500	0.31219500
C	-0.22216400	-1.63337000	0.53255000
H	-1.54649000	0.55470700	1.24089200
H	-0.52719500	-0.66986800	-1.34547800
H	-1.49531700	-2.82049300	-0.73199600
H	-2.95469400	-0.77439600	-1.08872900
O	-2.60848200	-1.77304400	0.65011700
O	0.76768200	0.41843200	-0.34166600
C	-1.46836100	-3.75822900	1.18654600
H	-0.61060400	-4.37249300	0.95467100
H	-2.36422400	-4.33223800	0.96076700
H	0.89814500	0.87272600	0.48399200
O	-1.39788600	-3.44982100	2.54691700
H	-2.14392700	-2.91143700	2.77772400
C	-5.95255500	-1.36682400	0.22917000
C	-7.32865100	-1.39082000	-0.44633500
C	-5.17700700	-0.13509700	-0.21935100
H	-6.07230300	-1.32461400	1.30494700
C	-8.04578300	-0.03887000	-0.36781000
H	-7.16311200	-1.57678800	-1.50003300
C	-6.01749200	1.11294400	0.01816700
H	-4.97540900	-0.21115100	-1.28092400
H	-8.40198300	0.15858900	0.63367600
H	-6.28334200	1.18397800	1.07194400
O	-7.17757100	0.98621300	-0.76834200
O	-9.09314800	-0.08683800	-1.26129600
O	-3.96338900	0.00856400	0.48386500
C	-5.32320200	2.40111200	-0.38098900
H	-6.03498900	3.21736900	-0.31143300
H	-4.51071500	2.59535300	0.30091200
O	-4.76301200	2.33818800	-1.66818900
H	-5.46096800	2.25056900	-2.30553200
O	-5.30112900	-2.54779500	-0.14782000
H	-4.47936500	-2.60771300	0.32512900
C	-10.15842300	0.75222500	-1.13200800
C	-10.11836700	1.95771400	-0.45176900
C	-11.32942700	0.33778800	-1.75228300
C	-11.26623700	2.73600500	-0.38305700
H	-9.20948400	2.30012900	0.00146300
C	-12.46033600	1.12741300	-1.68441300
H	-11.33293300	-0.60000900	-2.27608300
C	-12.43800900	2.33172100	-0.99425200
H	-11.23174400	3.66999400	0.14900700
H	-13.36387000	0.79937200	-2.16664900
H	-13.32000700	2.94333700	-0.93858300
H	-0.16717200	-1.38044500	1.57866400
N	-8.16195300	-2.47443100	0.03117600
H	-8.29448900	-3.23942700	-0.58771500
C	-9.29602000	-3.90749500	1.62041600
H	-8.56430100	-4.68796900	1.80558400
H	-9.88952700	-3.77155400	2.51253400

H	-9.93258100	-4.22482200	0.80242600
N	-1.83012300	1.63497500	-0.48251900
H	-2.54121300	1.72748600	-1.17509300
C	-1.51939000	4.02693100	-0.77365300
H	-1.55337500	4.84349100	-0.06547500
H	-0.70501100	4.21885000	-1.46448600
H	-2.44520500	3.97986300	-1.33266200
C	-1.21419400	2.74958100	-0.02688800
O	-0.44638600	2.72556300	0.90348300
C	-8.57342900	-2.61456700	1.31260300
O	-8.37081900	-1.78005700	2.15601500
C	8.31934300	-1.85595500	-1.05486100
C	7.55955100	-3.17473600	-0.97870200
C	6.05740500	-2.90585800	-0.97256800
C	6.28718900	-0.83753200	-2.11007000
C	7.80496500	-0.97812900	-2.18987300
H	8.18149000	-1.31929600	-0.11860900
H	6.03612100	-0.27408200	-1.22021500
H	8.08076200	-1.43298000	-3.13213300
O	5.68443600	-2.11506900	-2.04487100
C	4.50682300	-2.33766900	0.85604500
C	3.27044500	-2.26642400	-0.03917800
C	4.51940000	-1.13699000	1.78969600
C	2.07406500	-2.29755500	0.91259800
H	3.21416200	-3.12477200	-0.69932000
C	3.18932100	-0.86939200	2.49877300
H	3.14863900	-1.47874800	3.39726400
O	2.06713200	-1.14832200	1.68974500
C	6.92897500	3.22397800	2.37631100
C	5.78890100	2.58373400	1.60033500
C	5.75749200	2.99927300	0.13288900
C	3.37204200	2.70097600	1.70372200
C	4.45678900	2.57085200	-0.52340000
H	5.85039600	4.07488600	0.07335800
C	3.27838600	3.14902200	0.24940200
H	2.64605800	3.21990900	2.31426000
H	4.38995800	1.49270900	-0.51137700
O	4.60834800	2.99825100	2.26717200
O	6.85527100	2.39511800	-0.52251800
H	6.86186300	2.73898300	-1.40766600
O	4.45486600	3.03001300	-1.85380000
H	4.26178600	2.29152000	-2.42588600
O	3.30214600	4.54842100	0.22854400
H	3.38256800	4.82177800	-0.67710200
O	5.47972400	-1.27723300	2.80134600

H	6.32379500	-1.42142100	2.39368000
O	3.24292200	-1.09055300	-0.79319300
H	2.43463100	-0.60396600	-0.63307400
O	7.83403600	-3.97331100	-2.09004900
H	8.77541400	-3.98559600	-2.21362800
O	9.67590600	-2.16420200	-1.23449400
H	10.13447400	-1.36885300	-1.47764400
O	8.44979100	0.27158800	-2.15199400
H	8.13497200	0.77470000	-1.40701200
H	5.51885700	-3.83649500	-1.08402300
C	5.68648400	-0.12592800	-3.30920100
H	6.28474000	0.73960800	-3.55634400
C	3.08162200	0.60377800	2.87477400
O	4.38514200	0.33275300	-3.04899300
H	3.91848800	-0.32631300	-2.54078900
H	5.69300300	-0.80108500	-4.15949400
H	7.87217800	2.97296900	1.91186800
H	6.92598500	2.82272800	3.38724500
O	6.83267600	4.61680300	2.39231200
H	5.96070300	4.84765000	2.68802700
H	3.90636000	0.87219800	3.52168800
H	2.14699900	0.78730500	3.39586600
O	3.11738900	1.33305900	1.67595700
H	7.82482500	-3.69183100	-0.06099700
H	4.74997700	-0.29083500	1.16522500
H	4.44927700	-3.25948900	1.43152300
O	0.92554300	-2.33339700	0.12957700
H	2.34701300	2.77969700	-0.16277300
O	5.77573800	-2.28727900	0.24131200
H	5.88947600	1.50531000	1.66108300
H	2.10325600	-3.17839700	1.55056300

A5

C	-0.58603900	3.32076000	-1.00739000
C	-1.95613500	3.00666700	-0.39566500
C	-2.94200100	2.71363600	-1.53165400
C	-1.20460000	1.86045600	-2.92952300
C	-0.12578500	2.15234500	-1.88039000
H	-1.84821600	2.11024500	0.20393300
H	-0.65802800	4.19342000	-1.64367000
H	-1.25606700	2.72193100	-3.59481400
H	-3.06970800	3.61141700	-2.12914500
O	-2.46076100	1.67335200	-2.30828700
O	0.36298000	3.55703400	-0.00828600
C	-0.97129700	0.61366800	-3.77049500
H	0.00422100	0.66905000	-4.22623300

H	-1.71641100	0.60395500	-4.56412300
H	0.26109500	4.46740800	0.25825200
O	-1.02754200	-0.55779300	-3.02226700
H	-1.93213300	-0.72090000	-2.77592800
C	-5.17162500	1.42523500	0.84092400
C	-5.74682000	0.13660000	1.44576500
C	-4.51717000	1.15234100	-0.51807100
H	-5.99492900	2.11710400	0.67690800
C	-6.66407900	-0.52181700	0.41914600
H	-4.94060600	-0.55724600	1.65649300
C	-5.49843100	0.40277000	-1.42394300
H	-3.62473500	0.55831800	-0.38216600
H	-7.52179900	0.11907400	0.21394700
H	-6.33444200	1.06569200	-1.64371100
O	-5.96717700	-0.75267200	-0.75327300
O	-7.08956400	-1.72191900	0.94351600
O	-4.22772900	2.39736000	-1.11071900
C	-4.92773800	-0.10451600	-2.73610800
H	-5.74035500	-0.53067100	-3.31815200
H	-4.49187600	0.70833100	-3.29007800
O	-3.92313800	-1.05868300	-2.53468300
H	-4.28632000	-1.79418500	-2.05777700
O	-4.23606300	2.04332300	1.67430900
H	-4.18121100	1.60986600	2.52460600
C	-8.18383000	-2.36316600	0.43452100
C	-8.74017800	-2.09074500	-0.80319500
C	-8.73332400	-3.34100600	1.25258400
C	-9.86562600	-2.79731200	-1.20816300
H	-8.29908200	-1.36614300	-1.45785600
C	-9.84678900	-4.04096800	0.83195600
H	-8.27485600	-3.53980100	2.20358500
C	-10.42455600	-3.76942500	-0.40099700
H	-10.29568500	-2.58437400	-2.17040600
H	-10.26660300	-4.79853700	1.46903200
H	-11.29213400	-4.31362900	-0.72633900
H	0.06342800	1.27160500	-1.28623000
N	-6.51207800	0.37217900	2.66375300
H	-7.45378400	0.04941700	2.65666700
C	-6.84324400	0.33912800	5.07045700
H	-6.89066100	-0.70803700	5.35257100
H	-6.42967700	0.89650100	5.89813900
H	-7.84834000	0.68469500	4.86034400
N	-2.50630400	4.03222100	0.47670000
H	-3.27255700	3.68983000	1.01475700
C	-2.96447200	6.06927600	1.68136800
H	-2.33052900	6.44820100	2.47335200
H	-3.37819400	6.92233800	1.15645000
H	-3.77105600	5.48934600	2.11099600
C	-2.10316700	5.28760800	0.71153200
O	-1.13059200	5.81458900	0.21317300
C	-5.92659900	0.47839600	3.87904300
O	-4.74133500	0.66351600	4.00852600
C	7.21950700	2.12500500	1.73909300
C	5.70975000	1.97475100	1.86657700
C	5.17507000	1.02407200	0.80302600
C	7.26855200	-0.11061100	0.61781600
C	7.89559900	0.76072400	1.70315500
H	7.44163800	2.65171800	0.81288800
H	7.45860000	0.33347600	-0.35603800
H	7.76788100	0.28423500	2.66520100
O	5.87471300	-0.19203200	0.82668500
C	4.39962000	1.37591200	-1.47999800
C	3.10340400	2.17007700	-1.32320500
C	4.13555000	-0.11608800	-1.68291100
C	2.21636500	1.84671700	-2.52576800
H	3.33729400	3.23178600	-1.33884300
C	3.02908200	-0.36007500	-2.71908600
H	3.46043000	-0.20854400	-3.70789500
O	1.92883100	0.49471100	-2.55484700
C	4.97480500	-4.90308400	-0.27976400
C	3.94773700	-3.78544900	-0.13325400
C	3.47397200	-3.61280200	1.31378100
C	1.76618800	-3.30846600	-0.95589300
C	2.23293400	-2.73615400	1.41829400
H	3.22656600	-4.58396300	1.72114400
C	1.16289800	-3.20297900	0.43869200
H	1.06192800	-3.75797200	-1.64314900
H	2.49243400	-1.70778900	1.18642000
O	2.87818800	-4.14945000	-0.96860300
O	4.50928100	-3.10903400	2.13763100
H	4.78526600	-2.24914500	1.83152900
O	1.68146800	-2.81589700	2.70781900
H	2.36375600	-2.64170900	3.34407900
O	0.67754300	-4.46444200	0.79606700
H	0.45236100	-4.43581300	1.71769800
O	5.26646700	-0.76814500	-2.18355100
H	5.82111200	-1.11242400	-1.49086300
O	2.50358900	1.81373500	-0.11778500
H	1.80858800	2.43475700	0.08683800
O	5.37942900	1.42467100	3.11031500

H	5.82398600	1.93161600	3.77845000
O	7.66459400	2.87401900	2.83949900
H	8.60776400	2.78726800	2.90300000
O	9.28707200	0.91254400	1.53518400
H	9.47034200	1.25492000	0.66887400
H	4.15529800	0.77961900	1.02926700
C	7.79658300	-1.52977200	0.59929000
H	8.88010100	-1.51664900	0.56434500
C	2.50383600	-1.78324100	-2.64101700
O	7.27452300	-2.17363800	-0.52920500
H	7.07024700	-3.08236100	-0.32422600
H	7.49382400	-2.03169600	1.51367500
H	5.22808700	-5.01531300	-1.32411000
H	4.54301500	-5.83808100	0.06360200
O	6.17371100	-4.62771100	0.40583400
H	5.96800700	-4.41714500	1.30979700
H	3.28962300	-2.47195900	-2.92152200
H	1.66219800	-1.89391400	-3.31492000
O	2.08664900	-2.01755100	-1.31967800
H	5.23270300	2.93984200	1.73459700
H	3.81310300	-0.54570900	-0.75000800
H	4.91159900	1.73515200	-2.36490100
O	1.03320000	2.57019000	-2.56907700
H	0.36001000	-2.47404300	0.40921200
O	5.30489500	1.65298000	-0.42658200
H	4.39319500	-2.85840300	-0.47560100
H	2.74545900	2.13796800	-3.43201600

A6

C	-0.73498900	2.19278800	-0.87155300
C	-1.94212900	1.97440500	0.05628500
C	-2.95563000	1.06288500	-0.63127300
C	-1.30376400	0.01864000	-1.99120200
C	-0.17347800	0.87185200	-1.41179500
H	-1.61621400	1.48396800	0.96637900
H	-1.07106800	2.77680300	-1.72526000
H	-1.69726100	0.52390600	-2.87171300
H	-3.36227600	1.55969800	-1.51102500
O	-2.33634100	-0.12034300	-1.02678900
O	0.30319000	2.88552000	-0.23651000
C	-0.92026200	-1.40108800	-2.37770700
H	-0.08949300	-1.38092200	-3.06240600
H	-1.77120600	-1.85340500	-2.88519600
H	0.00427600	3.29807400	0.57375400
O	-0.53432300	-2.16892800	-1.27902500
H	-1.23161000	-2.15942800	-0.63694900
C	-5.60426800	-0.78748200	-0.69483100
C	-7.09406700	-0.90294100	-1.04197500
C	-5.28476700	0.62963900	-0.23396600
H	-5.35964400	-1.47251600	0.10801000
C	-8.00315500	-0.28479500	0.02461300
H	-7.25603200	-0.32059500	-1.94022200
C	-6.23590400	1.02359600	0.88970800
H	-5.43329900	1.31164500	-1.06272600
H	-8.04314400	-0.89978300	0.91250500
H	-6.14761600	0.31564400	1.71246600
O	-7.53891200	0.99510500	0.36055300
O	-9.25297100	-0.15235200	-0.53890100
O	-3.96517400	0.75505000	0.24959600
C	-5.99089400	2.41675200	1.43604500
H	-6.79436500	2.67097500	2.11952300
H	-5.06022100	2.43127800	1.98115700
O	-5.87875200	3.38127800	0.41966500
H	-6.70654200	3.44240300	-0.04123600
O	-4.90406000	-1.12165700	-1.85992500
H	-3.97640200	-1.15632600	-1.65889900
C	-10.36306800	-0.04993800	0.24384400
C	-10.34662100	0.37557700	1.56147800
C	-11.56330400	-0.38723600	-0.36722400
C	-11.54052500	0.44323100	2.26724400
H	-9.42840200	0.66855700	2.03033300
C	-12.74412500	-0.30487900	0.34404500
H	-11.54868800	-0.71173600	-1.39108700
C	-12.74053500	0.10711600	1.66974900
H	-11.52288600	0.77068400	3.29149600
H	-13.66971800	-0.56884600	-0.13567100
H	-13.65971800	0.16646300	2.22353300
H	0.33408000	0.32907500	-0.62938100
N	-7.48109900	-2.26080500	-1.36286800
H	-7.62362800	-2.46572200	-2.32398600
C	-7.68594600	-4.65974600	-1.09478600
H	-6.78074300	-5.03548700	-1.56196700
H	-7.97204900	-5.33629500	-0.30292900
H	-8.46703500	-4.62354400	-1.84548400
N	-2.61842500	3.22224900	0.39684300
H	-3.56478900	3.33192700	0.10066400
C	-3.09058100	5.20335300	1.73146000
H	-3.16533000	5.23460800	2.81115000
H	-2.62501300	6.12820700	1.41021900
H	-4.07769700	5.12825400	1.29730100
C	-2.17707100	4.06215600	1.35363800

O	-1.09158700	3.93705600	1.87428300
C	-7.40544200	-3.29885100	-0.49717500
O	-7.12368900	-3.15692000	0.66431600
C	7.51100800	3.36051600	-0.02996400
C	6.13915200	3.01231800	0.53138000
C	5.66391200	1.67009600	-0.01008700
C	7.89803500	0.91560300	-0.38833300
C	8.48477800	2.20748100	0.17481300
H	7.41405800	3.55300100	-1.09693200
H	7.78184500	1.01147300	-1.46496500
H	8.66503500	2.08884100	1.23416800
O	6.63439500	0.67850200	0.19535400
C	4.40278700	1.04860300	-1.99977200
C	3.00444400	1.61391700	-1.75645100
C	4.48299100	-0.43901900	-1.65746900
C	2.01299700	0.74012800	-2.52376200
H	2.95834500	2.62455100	-2.15453200
C	3.29529900	-1.21972200	-2.23885100
H	3.47934000	-1.35923200	-3.30335200
O	2.06613700	-0.56213200	-2.06304300
C	6.74303500	-4.19817500	0.85464500
C	5.51803600	-3.29267600	0.92571800
C	5.30321300	-2.70760600	2.32551500
C	3.16937400	-3.51895000	0.60670700
C	3.92719800	-2.07474400	2.48466500
H	5.38764700	-3.50112800	3.05579600
C	2.84022500	-3.03268100	2.01201900
H	2.48246000	-4.29762700	0.30296700
H	3.87335000	-1.16649300	1.89194800
O	4.43301200	-4.10325800	0.55023900
O	6.31922200	-1.77889200	2.65559500
H	6.30795300	-1.04686400	2.04477900
O	3.67002400	-1.78631100	3.83459200
H	4.39729800	-1.28827100	4.18639100
O	2.76517100	-4.15130200	2.84618700
H	2.71089100	-3.84142400	3.74175800
O	5.62136000	-1.02774100	-2.21520300
H	6.36816100	-1.00207900	-1.62517500
O	2.75104000	1.60893100	-0.38772000
H	1.96749000	2.12446800	-0.20956700
O	6.20031600	2.89068000	1.92396300
H	6.61864400	3.67130600	2.26553800
O	7.95749700	4.51529300	0.63058700
H	8.88732800	4.61676100	0.46850900
O	9.73663100	2.52209300	-0.39165700

H	9.66047800	2.56276800	-1.33708500
H	4.80017400	1.35383800	0.54210100
C	8.74790000	-0.30717200	-0.11373800
H	9.76645900	-0.12517400	-0.43729900
C	3.16916200	-2.58895900	-1.59355400
O	8.19398400	-1.38726800	-0.81204300
H	8.26779900	-2.18514700	-0.29480100
H	8.76130200	-0.49590900	0.95574400
H	6.80894600	-4.62599500	-0.13532900
H	6.63326000	-5.01133800	1.56537500
O	7.94439500	-3.49915100	1.07995500
H	7.87437200	-3.02594700	1.90150700
H	4.03827200	-3.18256100	-1.84322500
H	2.27574700	-3.08152600	-1.96070300
O	3.07358900	-2.40619700	-0.20445500
H	5.42093500	3.77221400	0.24306300
H	4.46160800	-0.55555200	-0.58751000
H	4.62975900	1.14855800	-3.05473000
O	0.70047900	1.19510400	-2.47144600
H	1.89011300	-2.50990900	1.97949700
O	5.39312700	1.83281800	-1.36157400
H	5.64444800	-2.48255600	0.21653000
H	2.26667300	0.77551700	-3.58201700

B1

C	1.28662600	-3.54311200	-1.62044300
C	2.66665700	-3.04009600	-1.17063100
C	3.24091700	-2.07977400	-2.22194100
C	1.06515400	-1.45115300	-2.96744800
C	0.40231000	-2.33821000	-1.91736300
H	2.50980800	-2.48773700	-0.25122000
H	1.37733700	-4.12987800	-2.52627900
H	1.17547600	-2.01821800	-3.89138500
H	3.46779500	-2.63856000	-3.12442500
O	2.33808900	-1.06244800	-2.49591800
O	0.67753000	-4.29359100	-0.61834300
C	0.30371400	-0.17308000	-3.27812200
H	-0.65314400	-0.42351000	-3.70520900
H	0.87248000	0.39439000	-4.01030400
H	1.31546378	-4.38177012	0.08243866
O	0.05903900	0.59696400	-2.13586400
H	0.88198000	0.96387800	-1.82460000
C	5.49359131	-0.14351186	-3.50591217
C	6.76877790	0.07464096	-4.33173775
C	5.54017408	-1.48076122	-2.75691022
H	5.43357446	0.64728950	-2.76148673

C	7.98013508	-0.10024706	-3.42170093
H	6.83259311	-0.68001450	-5.10760537
C	6.84525689	-1.58209399	-1.95977941
H	5.48960856	-2.29691599	-3.46351530
H	7.98926323	0.67098246	-2.65115237
H	6.82868689	-0.82516280	-1.17648544
O	7.94043531	-1.34886180	-2.82758523
O	9.11121265	0.00125811	-4.20076643
O	4.45130300	-1.49777100	-1.86296000
C	7.13214447	-2.92898828	-1.32054244
H	8.04819760	-2.84003361	-0.74305873
H	6.32931366	-3.20535573	-0.66115813
O	7.25881343	-3.94699434	-2.27556813
H	7.98484489	-3.74199268	-2.85099140
O	4.33468816	-0.10031788	-4.28460120
H	3.55062248	-0.23773716	-3.75602570
C	10.33195839	0.24771719	-3.63861949
C	10.62017541	0.07426853	-2.29598000
C	11.30971320	0.69902663	-4.51484024
C	11.89579295	0.37385867	-1.83461631
H	9.88350910	-0.31139238	-1.62012590
C	12.57558334	0.98394485	-4.04249182
H	11.06194484	0.81468254	-5.55376245
C	12.87595623	0.82817432	-2.69593662
H	12.11737668	0.23774104	-0.79126415
H	13.32907483	1.33134546	-4.72631986
H	13.86091668	1.05210292	-2.32930251
H	0.26513800	-1.77454200	-1.01171900
N	6.82956769	1.39640673	-4.94162269
H	6.53403443	1.45743843	-5.89018027
C	6.45487789	3.79661658	-4.98480687
H	5.37908787	3.93431152	-5.03046570
H	6.88375263	4.63685670	-4.45861498
H	6.84054437	3.76127239	-5.99663039
N	3.65150600	-4.07102300	-0.88304900
H	4.42761472	-4.03044998	-1.50729280
C	4.93968106	-5.85359932	0.10360700
H	5.47786938	-5.74866478	1.03758283
H	4.58533220	-6.87589648	0.04302570
H	5.61117780	-5.65913901	-0.72279487
C	3.73554572	-4.93445167	0.13993105
O	2.93907500	-5.02529009	1.04801900
C	6.74881885	2.53307866	-4.21260740
O	6.89215961	2.53998878	-3.01449119
C	-4.38265300	-1.57416200	4.19101400
C	-3.27674600	-0.91439300	3.37862400
C	-3.71845900	-0.72129900	1.93357700
C	-6.02802900	-0.66604900	2.53633900
C	-5.69836900	-0.82647900	4.01853100
H	-4.51034600	-2.59684200	3.84156200
H	-6.19630700	-1.64489800	2.09522600
H	-5.60380000	0.15014300	4.47263700
O	-4.94541000	-0.04162000	1.87833300
C	-3.61610700	-2.16331100	-0.03804300
C	-2.12722300	-2.18289400	-0.39636500
C	-4.37756200	-1.15770300	-0.90219300
C	-2.04856600	-2.51611700	-1.88704300
H	-1.62455900	-2.94453500	0.18461800
C	-3.97206200	-1.24309500	-2.38573800
H	-4.51229900	-2.07658400	-2.83010500
O	-2.59117800	-1.42632600	-2.57720900
C	-7.17406100	3.05767700	-1.13147500
C	-5.72278600	2.59201300	-1.17842800
C	-4.80588000	3.43621500	-0.28795600
C	-4.01656200	2.38842800	-2.82718400
C	-3.33135800	3.17895300	-0.56858900
H	-4.99973000	4.48354700	-0.47693000
C	-3.04175100	3.27488300	-2.06241300
H	-3.92308300	2.55205900	-3.89283400
H	-3.06506900	2.18379900	-0.22533400
O	-5.34115200	2.69404000	-2.52748300
O	-5.09513300	3.23689600	1.08258000
H	-4.93971800	2.32762600	1.32588800
O	-2.53326600	4.13872900	0.07401700
H	-2.78361700	4.18207600	0.98851200
O	-3.21350000	4.58539600	-2.51829400
H	-2.71540200	5.15739600	-1.94773500
O	-5.75603400	-1.38661100	-0.87108900
H	-6.19434100	-0.85008400	-0.21768800
O	-1.53695100	-0.94601900	-0.08741000
H	-1.43269300	-0.39781800	-0.86142300
O	-2.98960200	0.35672700	3.88954300
H	-2.84448900	0.26730300	4.82337100
O	-3.97896100	-1.56897500	5.53510800
H	-4.73063200	-1.77113000	6.07843600
O	-6.72546100	-1.47885100	4.73001600
H	-6.90745700	-2.32116100	4.33156500
H	-3.01121500	-0.09107400	1.42962900
C	-7.25407300	0.18549600	2.28194200
H	-8.08798900	-0.19382900	2.86113300

C	-4.35264200	0.03148100	-3.11789900
O	-7.54712400	0.14146200	0.91247500
H	-7.82835800	1.00218900	0.61355100
H	-7.05237900	1.20209000	2.60588600
H	-7.75540600	2.47274900	-1.82968200
H	-7.23116400	4.09797000	-1.43636400
O	-7.75527400	2.87756000	0.13786700
H	-7.20808700	3.30440200	0.78773800
H	-5.42530400	0.16639300	-3.08128600
H	-4.03697200	-0.03344000	-4.15455400
O	-3.68887800	1.09174700	-2.47915000
H	-2.39171900	-1.54120400	3.38745900
H	-4.14294200	-0.16622000	-0.55487800
H	-4.03131500	-3.14609900	-0.22483900
O	-0.81960400	-2.81089100	-2.43000600
H	-2.03429900	2.92263600	-2.25519800
O	-3.81160800	-1.97676200	1.35324800
H	-5.68172100	1.55858200	-0.85265600
H	-2.65625700	-3.39463500	-2.08059500
B2			
C	1.02268600	1.39900100	-0.49582800
C	2.54833500	1.56383100	-0.43292300
C	3.06711700	1.11593700	0.93685600
C	1.28737400	-0.43380200	1.21721600
C	0.63589400	-0.02509300	-0.10186100
H	2.96937800	0.90847500	-1.18708000
H	0.54261100	2.07900100	0.19567400
H	0.83608000	0.15613100	2.01339800
H	2.67716600	1.77974200	1.70224900
O	2.67428500	-0.19080800	1.17677200
O	0.57292200	1.62892700	-1.79703500
C	1.12186600	-1.91271300	1.53689500
H	0.06996600	-2.15784300	1.54530900
H	1.51906600	-2.09553700	2.53257600
H	0.45071000	2.57011900	-1.87584500
O	1.74423100	-2.72485500	0.59168700
H	2.68603700	-2.67027300	0.71998800
C	6.32687700	1.11224500	-0.36257300
C	7.45050700	0.20906600	-0.89252100
C	5.30514500	0.28737200	0.43200500
H	6.74543000	1.85684400	0.30841700
C	8.02563100	-0.62240100	0.25192500
H	7.01151500	-0.48254800	-1.60382500
C	6.01556100	-0.56180000	1.49167100
H	4.76227000	-0.35995800	-0.24167100
H	8.52244500	0.02440000	0.97214000
H	6.46570900	0.11012800	2.22144400
O	7.01958700	-1.34124300	0.87228700
O	8.93435700	-1.50497100	-0.29819300
O	4.44762200	1.19602800	1.08339400
C	5.14532300	-1.56288900	2.23106200
H	5.75104400	-2.02968500	3.00284000
H	4.31287700	-1.06543900	2.69528500
O	4.62483100	-2.53752300	1.36931300
H	5.34519700	-3.00616100	0.96722200
O	5.63868700	1.73063400	-1.40404300
H	6.23226800	2.37893600	-1.78079900
C	9.87513600	-2.12231800	0.47756300
C	9.81374400	-2.20023200	1.85807400
C	10.93590600	-2.69347800	-0.21175100
C	10.83640900	-2.84194800	2.54465100
H	8.97954100	-1.79639300	2.39578600
C	11.94096000	-3.33558700	0.48455600
H	10.95435000	-2.63050200	-1.28425700
C	11.90050300	-3.40957100	1.87009600
H	10.78631800	-2.90091400	3.61711300
H	12.75920800	-3.77700800	-0.05548400
H	12.68405500	-3.90764500	2.41103800
H	0.94327200	-0.70907600	-0.88510900
N	8.57931500	0.88354600	-1.53146600
H	9.32384800	0.25519600	-1.73411600
C	10.07448100	2.43827800	-2.61819500
H	9.91405700	2.51740500	-3.68789800
H	10.42942100	3.39690400	-2.26537000
H	10.82613700	1.68198300	-2.43080300
N	3.03631100	2.90693900	-0.70627500
H	4.02558100	2.91805300	-0.81857600
C	3.26628600	5.24750900	-1.23885700
H	3.04413200	5.62129600	-2.23116800
H	3.00687300	6.02512100	-0.53085400
H	4.32543400	5.03649500	-1.16498700
C	2.38102100	4.04733900	-0.97165200
O	1.17658900	4.17369600	-1.00652900
C	8.74403600	2.14888700	-1.95804500
O	7.91823900	3.02769700	-1.85760500
C	-7.29109900	-3.29306800	-1.91997500
C	-5.91054700	-3.65039300	-2.45778300
C	-4.82184000	-2.93819200	-1.65845300
C	-6.22592200	-2.66438600	0.23077900
C	-7.35811800	-3.46352300	-0.40679300

H	-7.50153000	-2.25135700	-2.15548700
H	-6.36123800	-1.61855900	-0.01093700
H	-7.24193200	-4.51221500	-0.16607600
O	-4.99017900	-3.11183300	-0.29362000
C	-3.68419400	-0.81387300	-1.93359900
C	-2.82337200	-0.95838500	-0.67770000
C	-4.16936800	0.62422900	-2.05965100
C	-1.64863700	-0.00073400	-0.87723500
H	-2.46233600	-1.96904500	-0.55585500
C	-3.09887200	1.68546400	-1.75597700
H	-2.58267700	1.91662200	-2.68308700
O	-2.16698200	1.29283700	-0.77972900
C	-8.22961700	1.11014800	0.19100000
C	-6.87627500	1.53443600	0.75104000
C	-6.88071500	1.69816700	2.27140500
C	-5.38395300	3.38455400	0.49041400
C	-5.55458900	2.27776000	2.73921600
H	-7.66893800	2.38630200	2.54648400
C	-5.25844900	3.57662700	1.99922900
H	-5.41260700	4.35476600	0.01535200
H	-4.76145900	1.56139300	2.53601300
O	-6.57861600	2.76656100	0.13399900
O	-7.16583300	0.49329100	2.94175600
H	-6.50177400	-0.17452300	2.75565600
O	-5.59257100	2.57651500	4.10902500
H	-5.99753200	1.84819500	4.56452800
O	-6.17597500	4.56628200	2.36402600
H	-6.22759300	4.57762300	3.31206200
O	-4.63732100	0.90071700	-3.35213300
H	-5.37547200	0.33353100	-3.53263800
O	-3.51634400	-0.60694300	0.49150600
H	-3.37979700	0.32150600	0.65488000
O	-5.67320200	-5.02144100	-2.33535700
H	-6.44771200	-5.47734800	-2.64194000
O	-8.21970600	-4.12603500	-2.56138000
H	-9.03833500	-4.07727300	-2.08184300
O	-8.62631200	-3.08611700	0.05963900
H	-8.71245400	-2.13553800	0.05378500
H	-3.86453600	-3.37899700	-1.89965900
C	-6.14644200	-2.78513500	1.74113700
H	-7.13921400	-2.75176700	2.16550700
C	-3.75998600	2.94249300	-1.21397800
O	-5.42403300	-1.71797400	2.30751300
H	-4.62879900	-1.56814500	1.80057200
H	-5.69289300	-3.73556600	2.00260700
H	-8.17378800	1.08727800	-0.88839600
H	-8.98181400	1.84174300	0.47019100
O	-8.60274600	-0.17803500	0.61424000
H	-8.56439800	-0.20580200	1.56457300
H	-4.54547500	3.25249700	-1.89281000
H	-3.02694100	3.73594800	-1.12375400
O	-4.28629000	2.65046200	0.06270000
H	-5.83432200	-3.34211900	-3.49661000
H	-4.97402700	0.73313100	-1.34815900
H	-3.06505700	-1.06368200	-2.79269300
O	-0.74542500	-0.16201600	0.14352600
H	-4.24437300	3.89587700	2.21724200
O	-4.86002500	-1.58988300	-1.99813800
H	-6.13561100	0.78931900	0.48299200
H	-1.17789300	-0.14916000	-1.84054400

B3

C	0.34475000	-2.64870200	-1.59033300
C	1.58271500	-2.57799000	-0.67529900
C	2.47713400	-1.38423500	-1.02987500
C	0.73035200	-0.20832000	-2.12062800
C	-0.29376400	-1.26987000	-1.74489200
H	1.22978600	-2.42728200	0.33974800
H	0.64421700	-2.99455800	-2.57634600
H	1.16614700	-0.44643500	-3.08975300
H	2.97858200	-1.56919000	-1.97639100
O	1.74094900	-0.20126200	-1.12826900
O	-0.64853100	-3.46258900	-1.05827900
C	0.16370900	1.19975400	-2.17386700
H	-0.60462100	1.25365300	-2.92709800
H	0.96110200	1.88907700	-2.44256500
H	-0.33891600	-4.36476200	-1.10949600
O	-0.42730900	1.57532900	-0.96098200
H	0.22300500	1.53542300	-0.27130400
C	4.85027900	0.66429400	-0.59033700
C	6.33065300	1.03922600	-0.73957500
C	4.71858400	-0.83139400	-0.32048500
H	4.41810300	1.20233000	0.24430300
C	7.20772300	0.45069400	0.36935900
H	6.68012700	0.59177700	-1.66152400
C	5.58798200	-1.19267700	0.88007100
H	5.07031900	-1.38261800	-1.18513600
H	7.05351100	0.96584200	1.30709400
H	5.28977600	-0.59277800	1.73865500
O	6.92128900	-0.91183700	0.52969700
O	8.51567200	0.56796000	-0.04653600

O	3.39969500	-1.22773700	-0.01632700
C	5.52174200	-2.65415500	1.27931300
H	6.25796400	-2.83558500	2.05583000
H	4.54459700	-2.87947900	1.67873000
O	5.72376800	-3.51449700	0.18939200
H	6.59316800	-3.36678200	-0.16225000
O	4.22789300	1.03565700	-1.78863800
H	3.28824100	1.00303000	-1.65803200
C	9.53743100	0.54936600	0.85428800
C	9.44946900	-0.01680100	2.11492200
C	10.72483100	1.13023100	0.42965600
C	10.55571100	0.01971500	2.95333900
H	8.54665000	-0.49600300	2.43754400
C	11.82008500	1.14972800	1.27057700
H	10.76739800	1.55969700	-0.55399800
C	11.74168300	0.59795700	2.54202600
H	10.48219600	-0.41812300	3.93287900
H	12.73617700	1.60223000	0.93509900
H	12.59349100	0.61764600	3.19710500
H	-0.75062200	-0.99650300	-0.80933800
N	6.52585200	2.46687000	-0.88121000
H	6.75613800	2.79582500	-1.78929800
C	6.31823900	4.82532000	-0.36829000
H	6.40454600	5.44257000	0.51386200
H	7.17535800	4.98528700	-1.01224300
H	5.42447300	5.11873800	-0.91041900
N	2.46277500	-3.74782000	-0.72926100
H	3.38991500	-3.57492200	-0.40711800
C	3.34009100	-6.00022900	-0.86434200
H	3.07257300	-6.75869800	-0.13829500
H	3.46739700	-6.49638300	-1.81908100
H	4.26847200	-5.53093300	-0.57196000
C	2.17677000	-5.04017500	-0.98636500
O	1.08977600	-5.45825100	-1.31171400
C	6.18553100	3.38004600	0.05793700
O	5.79173600	3.07088900	1.15256700
C	-7.16715900	-2.03484200	2.06834100
C	-6.55243900	-3.09718000	1.16442900
C	-5.23908700	-2.59867100	0.56574400
C	-4.91786300	-1.01439000	2.29692300
C	-6.14638300	-1.49411200	3.06294300
H	-7.51442900	-1.20703700	1.45258100
H	-5.21576900	-0.21427200	1.63213400
H	-5.86376300	-2.28742400	3.74255500
O	-4.39464300	-2.08677500	1.53744700
C	-4.71318200	-1.43765900	-1.50192500
C	-3.20529900	-1.37848600	-1.23913200
C	-5.17552700	-0.11309900	-2.09618400
C	-2.58968000	-1.15754900	-2.62767100
H	-2.83650400	-2.29325900	-0.80383300
C	-4.24713000	0.46443400	-3.17744900
H	-4.57378400	0.07179500	-4.13664700
O	-2.88343300	0.16306000	-2.98899100
C	-6.46770000	2.84127300	1.24876600
C	-5.11523900	2.89159500	0.54638800
C	-4.05670400	3.66464700	1.33300700
C	-4.25630400	3.69744800	-1.53356200
C	-2.78775100	3.79859800	0.50687100
H	-4.43503900	4.65503600	1.54939200
C	-3.10039500	4.42101200	-0.84780600
H	-4.61784200	4.31235000	-2.34565300
H	-2.35268900	2.81684100	0.35160800
O	-5.35030800	3.52341400	-0.69203400
O	-3.77844300	3.07272100	2.58094900
H	-3.41004600	2.19377900	2.46842500
O	-1.86229100	4.63992200	1.14684600
H	-1.83322300	4.40844900	2.06726700
O	-3.49289600	5.75513100	-0.69583400
H	-2.87360600	6.17180300	-0.10890000
O	-6.43840800	-0.22624500	-2.69474200
H	-7.06687000	-0.47122100	-2.02815500
O	-2.87766700	-0.32155300	-0.37080500
H	-2.65340200	0.46090600	-0.86793600
O	-6.25477300	-4.25144300	1.89202900
H	-7.00760000	-4.45393300	2.43419900
O	-8.25224100	-2.62576600	2.73208600
H	-8.49992700	-2.05938200	3.45341800
O	-6.73035000	-0.48749600	3.84660400
H	-6.83032900	0.31321200	3.33687000
H	-4.71438800	-3.43305400	0.12234800
C	-3.79299100	-0.49501200	3.17165100
H	-4.19517500	0.11511100	3.96719500
C	-4.35310100	1.98032700	-3.19223700
O	-2.90756900	0.31847500	2.43835000
H	-2.72716000	-0.09119300	1.59502900
H	-3.26459200	-1.33301300	3.61412600
H	-7.18292800	2.34900500	0.60454400
H	-6.82093700	3.85246300	1.42693100
O	-6.41790500	2.10956500	2.44875200
H	-5.72480000	2.47058900	2.99168000

H	-5.39756200	2.25822500	-3.26836100
H	-3.81447100	2.38067800	-4.04461400
O	-3.78301900	2.47936300	-2.00569100
H	-7.23816400	-3.32452100	0.35310900
H	-5.22028700	0.58381500	-1.27350200
H	-4.90339600	-2.24269400	-2.20738700
O	-1.23823000	-1.35531100	-2.78474200
H	-2.22641100	4.34780500	-1.48615800
O	-5.54848300	-1.62848900	-0.38364700
H	-4.76214400	1.87814900	0.39176200
H	-3.05327300	-1.83301600	-3.33761400
B4			
C	-0.80199100	-0.65629500	-1.85220100
C	-2.33642900	-0.74978000	-2.04210800
C	-3.09464100	-0.02031000	-0.93259700
C	-1.26014300	1.31548000	-0.29309100
C	-0.34282600	0.71813000	-1.34969300
H	-2.58934200	-0.26821000	-2.98001400
H	-0.50178600	-1.40131100	-1.12049600
H	-1.17582600	0.73937100	0.62616600
H	-2.99103600	-0.56041800	0.00689900
O	-2.59267800	1.26994200	-0.76419900
O	-0.13193600	-0.83798600	-3.06201900
C	-0.95477900	2.77412900	0.00943600
H	0.05916100	2.86145300	0.36787400
H	-1.62321300	3.11436300	0.79759000
H	-0.45445700	-1.63067500	-3.48710300
O	-1.06356200	3.58338500	-1.12405600
H	-1.94755100	3.52514400	-1.46222300
C	-5.73899300	1.33016100	0.33849300
C	-6.94430100	1.19621500	1.27807600
C	-5.41612400	-0.02026900	-0.29093800
H	-5.96771000	2.03170500	-0.45466100
C	-8.10398200	0.41780200	0.64992900
H	-6.62030300	0.60805100	2.12736400
C	-6.67381800	-0.58635500	-0.94083700
H	-5.09135800	-0.70348900	0.48496200
H	-8.60832000	1.00399100	-0.10535300
H	-7.04987000	0.11450600	-1.68469700
O	-7.62424800	-0.76990000	0.07966300
O	-8.96595000	0.08489000	1.67116500
O	-4.42117500	0.06872100	-1.28798600
C	-6.46306700	-1.92734700	-1.61679000
H	-7.42719000	-2.31974100	-1.92318000
H	-5.85130900	-1.79830500	-2.49612800

O	-5.79266700	-2.84371300	-0.78913000
H	-6.33113800	-3.02483000	-0.02838000
O	-4.68099300	1.80172400	1.12418000
H	-3.94624100	1.99738800	0.55534900
C	-10.26956500	-0.21479000	1.41309100
C	-10.73631200	-0.65245900	0.18507700
C	-11.14257700	-0.07393300	2.48344900
C	-12.08822800	-0.93074500	0.03344700
H	-10.06328100	-0.79493200	-0.63683600
C	-12.48274000	-0.36458400	2.32058400
H	-10.75431600	0.26445300	3.42607800
C	-12.96656700	-0.79119400	1.09128900
H	-12.44728200	-1.26710800	-0.92288200
H	-13.15310700	-0.25219200	3.15394000
H	-14.01056100	-1.01288100	0.96473700
H	-0.31170000	1.38582100	-2.20159400
N	-7.37387700	2.47311700	1.80845800
H	-7.14276600	2.66052500	2.75582100
C	-8.05702100	4.79686500	1.79317200
H	-8.74348300	5.40787000	1.22570300
H	-8.44964700	4.63500300	2.79047500
H	-7.11236300	5.32496500	1.88024300
N	-2.83051100	-2.12758300	-2.03288300
H	-3.62256700	-2.31411100	-1.45635600
C	-3.27643100	-4.38422200	-2.81308900
H	-3.64644000	-4.65076300	-3.79508600
H	-2.57642500	-5.15431600	-2.50904900
H	-4.09683200	-4.34761200	-2.11039900
C	-2.51856200	-3.08162300	-2.93213600
O	-1.66802900	-2.94692100	-3.78115000
C	-7.83140500	3.49784800	1.05177500
O	-8.03470700	3.39293100	-0.12998200
C	8.22937600	2.55402900	-0.84815200
C	7.12590900	3.14605200	-1.71695700
C	5.75670300	2.64704700	-1.26159700
C	6.53646800	2.14541400	0.91891400
C	7.91896000	2.72394600	0.63443100
H	8.31060900	1.48927500	-1.05972500
H	6.54727300	1.09007000	0.68091800
H	7.92777100	3.77866700	0.87648600
O	5.58207400	2.80062000	0.10487100
C	4.38052900	0.75931700	-1.91238500
C	3.25238200	1.06971500	-0.92693500
C	4.62010600	-0.74424300	-1.96018200
C	2.02848000	0.33321100	-1.46956300

H	3.05438700	2.12987200	-0.86908800	H	5.17201200	-0.98836800	-1.06481100
C	3.33824300	-1.59333200	-1.99307800	H	4.06936500	1.10759400	-2.89498400
H	3.05221300	-1.73512500	-3.03056400	O	0.92099000	0.67116400	-0.72906300
O	2.26478700	-1.02987800	-1.27957500	H	2.97302100	-3.93271000	2.05569300
C	7.77903800	-1.92868800	1.25905700	O	5.64714800	1.30987000	-1.63018000
C	6.27273000	-2.09694700	1.42680100	H	5.77329900	-1.23279700	1.00312100
C	5.84418000	-2.24526100	2.88712200	H	1.87049900	0.54693800	-2.51885500
C	4.60303500	-3.65332000	0.71112300	B5			
C	4.36084500	-2.57205500	2.96608100	C	0.68745800	-3.53779700	-1.50977100
H	6.39703600	-3.06173700	3.33235300	C	2.07610200	-3.06877400	-1.05325200
C	4.04855500	-3.80389600	2.12510400	C	2.73178900	-2.23553200	-2.16389700
H	4.58804800	-4.61833800	0.22485200	C	0.61666900	-1.55591800	-3.03287800
H	3.78982900	-1.72654700	2.58818600	C	-0.12444200	-2.32373300	-1.94356500
O	5.93720200	-3.26072000	0.70474400	H	1.92637400	-2.43387100	-0.18752800
O	6.14769100	-1.10594400	3.65600500	H	0.77830600	-4.20304400	-2.35992500
H	5.68715900	-0.33055600	3.32702900	H	0.71881700	-2.19507700	-3.90917900
O	3.97863200	-2.85870400	4.28453200	H	2.95025500	-2.88274000	-3.00737700
H	4.37372400	-2.21401900	4.85930900	O	1.89467600	-1.19926800	-2.55217400
O	4.64549200	-4.93902000	2.68045100	O	0.00065600	-4.17016500	-0.47612100
H	4.44585700	-4.94682600	3.60876500	C	-0.06880800	-0.26482500	-3.44880700
O	5.36081600	-1.11460200	-3.09081800	H	-1.04684000	-0.49316600	-3.84212900
H	6.20825200	-0.69113700	-3.04887300	H	0.51930800	0.19979800	-4.23616800
O	3.52965000	0.61510100	0.37176600	H	0.30779100	-5.07154900	-0.45648100
H	3.20913500	-0.27816800	0.45293200	O	-0.24158400	0.61119400	-2.37371600
O	7.10580800	4.53832300	-1.60804400	H	0.60837300	0.96226600	-2.12213400
H	8.00030600	4.84768300	-1.68515200	C	5.04550400	-1.07414200	0.20228200
O	9.42478600	3.20288400	-1.18964300	C	5.38399500	0.11035500	1.11927200
H	10.06604300	3.01355100	-0.51479700	C	4.05779100	-0.63786800	-0.88852000
O	8.92996100	2.13020900	1.40476800	H	5.94855100	-1.42424300	-0.28918600
H	8.84937000	1.17950000	1.38150100	C	5.83176000	1.30148800	0.27554300
H	4.99094800	3.24931200	-1.73039900	H	4.47349400	0.40276900	1.63146700
C	6.07945900	2.29150700	2.35815400	C	4.58208100	0.60975400	-1.60848400
H	6.90060200	2.08257600	3.02800800	H	3.10002900	-0.41563800	-0.44009300
C	3.60267400	-2.94329200	-1.34635100	H	6.77083600	1.07456100	-0.22500800
O	5.05510300	1.37702900	2.66948600	H	5.49414300	0.33958500	-2.13941600
H	4.41141500	1.37008100	1.96442100	O	4.85965400	1.61796000	-0.65708800
H	5.74742600	3.31028600	2.52897300	O	5.99729500	2.37236400	1.13132600
H	8.01669400	-1.90706300	0.20468500	O	3.96407400	-1.69293800	-1.81781400
H	8.28914200	-2.78074300	1.69802800	C	3.62644100	1.25908100	-2.59389100
O	8.24659300	-0.72430800	1.81403600	H	4.15115700	2.07562400	-3.08196800
H	7.96881000	-0.68282100	2.72306300	H	3.31576600	0.54907700	-3.33891400
H	4.47843200	-3.39000600	-1.80135800	O	2.47043500	1.73759000	-1.96165900
H	2.74981000	-3.59680200	-1.49157300	H	2.72151200	2.37387100	-1.30381100
O	3.80177700	-2.74165600	0.03626900	O	4.44905900	-2.11226100	0.91420700
H	7.27692100	2.84681200	-2.75019600	H	5.13246200	-2.50051900	1.45880300

C	6.72952400	3.46473900	0.75998300
C	7.08080700	3.75681300	-0.54652800
C	7.11744100	4.30340700	1.79578000
C	7.84115700	4.89011600	-0.80452900
H	6.75298700	3.13677000	-1.35659900
C	7.86615800	5.43113200	1.52229900
H	6.82123600	4.06189100	2.79999900
C	8.23820100	5.72963800	0.21868400
H	8.11266000	5.11460500	-1.82044900
H	8.16210400	6.07733400	2.32906300
H	8.82237700	6.60639400	0.00724300
H	-0.27691600	-1.67271900	-1.10031400
N	6.42656600	-0.11757500	2.11814700
H	6.66985200	0.71757200	2.60140900
C	8.05417800	-1.04701600	3.64116800
H	7.61859700	-1.39658700	4.57091000
H	8.91446900	-1.66567800	3.42554400
H	8.36890200	-0.01867400	3.76602900
N	2.99733400	-4.12174200	-0.65616400
H	3.80082300	-3.75183300	-0.19880800
C	4.04850800	-6.22947000	-0.13652300
H	3.72398700	-6.78091900	0.73818600
H	4.35486500	-6.95118000	-0.88333000
H	4.89327700	-5.60665000	0.12859300
C	2.86418500	-5.45639900	-0.67908100
O	1.89330400	-6.05522000	-1.08713400
C	7.03736000	-1.24052700	2.53741500
O	6.81845000	-2.35046100	2.10774800
C	-5.17351600	-0.76881500	3.81237800
C	-4.72309300	-2.19027900	3.49924900
C	-3.75334200	-2.17746000	2.32110200
C	-3.05862800	0.05224800	2.75104400
C	-3.98351700	0.17168700	3.95988600
H	-5.79446800	-0.41071100	2.99409800
H	-3.57852800	0.42770600	1.87844800
H	-3.44357900	-0.09171400	4.85983600
O	-2.70447400	-1.29957900	2.54277200
C	-4.12441500	-2.19466000	-0.10308700
C	-2.64080000	-2.08375400	-0.46209500
C	-4.93790800	-1.25756000	-0.98923600
C	-2.57442700	-2.49924100	-1.93886400
H	-2.03671800	-2.74265000	0.14259400
C	-4.51515800	-1.21358100	-2.46820000
H	-5.09602500	-1.95552400	-3.00900600
O	-3.14157700	-1.45075700	-2.67451800
C	-6.98136600	3.49675800	-0.40418300
C	-5.64548700	2.80061000	-0.60911400
C	-4.50983600	3.43689800	0.18667500
C	-4.16397900	2.43219000	-2.47778600
C	-3.16835300	2.87840900	-0.25269600
H	-4.52594500	4.50623600	0.02606100
C	-2.98776700	3.07349600	-1.75238400
H	-4.15575600	2.69138600	-3.52840400
H	-3.14420200	1.82151700	-0.03191500
O	-5.39074500	2.88401700	-2.00025600
O	-4.72379900	3.16067000	1.55667700
H	-4.05840600	3.63783200	2.03754100
O	-2.15172300	3.53418600	0.46516700
H	-1.57032600	2.87084100	0.82893800
O	-2.96795600	4.43411800	-2.08463000
H	-2.32060600	4.85545700	-1.53257400
O	-6.30213100	-1.58435800	-0.98053600
H	-6.61949700	-1.52448300	-0.08889200
O	-2.16840700	-0.77289300	-0.28636300
H	-2.20885400	-0.28722500	-1.10626300
O	-4.05142700	-2.75014000	4.58751400
H	-4.57549300	-2.59328900	5.36353500
O	-5.92327700	-0.81707800	4.99734100
H	-6.04153500	0.07104900	5.31224600
O	-4.43907100	1.48960000	4.14575600
H	-4.79549800	1.82899900	3.32997700
H	-3.30476900	-3.15401600	2.20423700
C	-1.76485600	0.83203100	2.91361800
H	-1.97589800	1.79692600	3.35251800
C	-4.79337000	0.17195500	-3.03417000
O	-1.12614600	1.07240700	1.68509400
H	-1.21590200	0.30510400	1.12512400
H	-1.10969200	0.28540700	3.58488200
H	-7.23443300	3.49893300	0.64653900
H	-7.74629400	2.94039600	-0.94129200
O	-6.94589000	4.82787000	-0.82487400
H	-6.61159200	4.84608900	-1.71305700
H	-5.84626100	0.40577600	-2.93348500
H	-4.51726700	0.20737600	-4.08326000
O	-4.00288400	1.06710200	-2.29671700
H	-5.58664200	-2.79251600	3.23187100
H	-4.79074300	-0.27666000	-0.56747700
H	-4.43748900	-3.22378500	-0.25892300
O	-1.34373800	-2.78009400	-2.47936700
H	-2.07875900	2.58368800	-2.08197000

O	-4.49704200	-1.81347300	1.20362300
H	-5.74232900	1.76111700	-0.31104200
H	-3.16346600	-3.39793500	-2.08625700
B6			
C	1.22321300	-2.79627000	-0.02406500
C	2.74577700	-2.60232700	0.00617000
C	3.20654000	-1.96752400	-1.31276600
C	1.11128100	-0.87619800	-1.63507500
C	0.55245700	-1.46584800	-0.34562700
H	2.96393500	-1.91274000	0.81389800
H	0.95194800	-3.49905200	-0.80203100
H	0.83801600	-1.52860400	-2.46330200
H	3.03771800	-2.67132900	-2.12171000
O	2.51568400	-0.78709200	-1.54218200
O	0.74510900	-3.23444900	1.20956200
C	0.60401100	0.52865700	-1.92053500
H	-0.47261200	0.50604100	-2.00121200
H	1.00880300	0.85627000	-2.87470000
H	0.85737900	-4.18023000	1.22491600
O	0.93694400	1.42048600	-0.90120000
H	1.86736800	1.61566400	-0.95937100
C	6.28766400	-1.24365300	0.19863500
C	7.12468100	-0.12868300	0.84331000
C	5.13633500	-0.64648800	-0.62239400
H	6.91153100	-1.82140800	-0.47724700
C	7.53176900	0.88963100	-0.21924500
H	6.49333700	0.38772500	1.55868400
C	5.66576100	0.41874000	-1.58874800
H	4.41432100	-0.19702900	0.04418600
H	8.20832700	0.43143300	-0.93773900
H	6.30567800	-0.07222400	-2.32096200
O	6.41078100	1.37850300	-0.86566300
O	8.16503600	1.93043900	0.43100700
O	4.56866500	-1.69556200	-1.37237900
C	4.61208900	1.22182800	-2.33108300
H	5.12086800	1.87653000	-3.03305900
H	3.95709700	0.56720800	-2.87701600
O	3.81857700	1.97546300	-1.45526500
H	4.37446100	2.58534900	-0.98701000
O	5.71967600	-2.07351900	1.16299300
H	6.43469900	-2.57811200	1.54887100
C	8.95961000	2.80848200	-0.25159100
C	8.94981600	2.95644000	-1.62767000
C	9.80859200	3.57807700	0.53168900
C	9.81314000	3.87242800	-2.21478000

H	8.27068400	2.39478700	-2.23712500
C	10.65528100	4.49069700	-0.06623100
H	9.78846100	3.45357100	1.59877200
C	10.66695700	4.64038300	-1.44619400
H	9.80357700	3.98534800	-3.28403700
H	11.30950300	5.08469600	0.54628300
H	11.32752700	5.34936600	-1.91055300
H	0.73158600	-0.76502600	0.46141200
N	8.35293400	-0.54608100	1.51762900
H	8.91050800	0.23088400	1.79274300
C	10.12771300	-1.75294600	2.62545800
H	9.94026800	-1.92834200	3.67919700
H	10.72224100	-2.57630800	2.25392500
H	10.68032000	-0.82841300	2.51619000
N	3.52675300	-3.80470800	0.24785300
H	4.48072400	-3.59046800	0.43591700
C	4.27092200	-6.06324900	0.65304700
H	4.08048100	-6.54587300	1.60421100
H	4.24748400	-6.82896200	-0.11256400
H	5.25290800	-5.60812700	0.67352700
C	3.14695400	-5.08472600	0.38097900
O	2.00773000	-5.48794000	0.30010300
C	8.80015800	-1.75625000	1.89941400
O	8.21921900	-2.80111500	1.71212000
C	-5.33072600	3.69962000	2.29394000
C	-3.87397100	3.53280100	2.70856900
C	-3.18233300	2.53826500	1.78024900
C	-4.67590200	2.97748800	-0.01261600
C	-5.45405600	4.00437400	0.80591800
H	-5.86084500	2.77205000	2.49913500
H	-5.15780400	2.01322700	0.09407200
H	-5.05866300	4.99436200	0.62077300
O	-3.34480700	2.89281100	0.45122000
C	-3.05163100	0.08770000	1.80987300
C	-2.23765200	-0.02133700	0.52057700
C	-4.15734700	-0.95963200	1.80886600
C	-1.66868400	-1.44526300	0.51972300
H	-1.43942000	0.70728100	0.49464700
C	-3.74171100	-2.34905400	1.29660500
H	-3.40491700	-2.93899700	2.14373000
O	-2.73479000	-2.31517200	0.31282000
C	-9.23109400	-0.99925500	0.77139400
C	-7.88219800	-0.92431800	0.07426200
C	-7.87296500	0.03625900	-1.11096500
C	-6.47991100	-2.47388500	-1.13334500

C	-6.59815700	-0.12280400	-1.92054100
H	-8.72801300	-0.17462500	-1.73876400
C	-6.43836800	-1.57219000	-2.36142800
H	-6.53820500	-3.51508600	-1.42211100
H	-5.75753700	0.15050400	-1.29983500
O	-7.60837000	-2.24748000	-0.35304700
O	-7.96553300	1.35417900	-0.60987200
H	-8.05926000	1.92753500	-1.36093400
O	-6.66131000	0.73156100	-3.03465500
H	-5.84866700	1.23061900	-3.07445500
O	-7.47312500	-1.95730800	-3.21839500
H	-7.54219400	-1.30184200	-3.90224300
O	-4.69416100	-1.15534000	3.08950600
H	-5.02599500	-0.32483700	3.40507400
O	-3.01380100	0.16146600	-0.63457600
H	-3.36372300	-0.68281500	-0.90285500
O	-3.18731700	4.74417500	2.61050600
H	-3.71622500	5.41206500	3.02960700
O	-5.87040900	4.74035500	3.06478300
H	-6.70439500	4.99126600	2.68603400
O	-6.81196100	4.04801800	0.44017100
H	-7.17694900	3.16818000	0.44060900
H	-2.11688600	2.54633300	1.96196700
C	-4.60831000	3.32019700	-1.49073700
H	-5.57739700	3.65620700	-1.83131200
C	-4.93597100	-3.02444000	0.63667000
O	-4.26170800	2.20821100	-2.27687500
H	-3.57770000	1.71038600	-1.83581000
H	-3.89538700	4.12623600	-1.63220100
H	-9.52365900	-0.01410600	1.10577600
H	-9.13399000	-1.64357500	1.64221000
O	-10.23472500	-1.46201900	-0.08203900
H	-9.94261800	-2.28058700	-0.46342600
H	-5.74859500	-3.11489300	1.34659900
H	-4.65558200	-4.01020100	0.28118000
O	-5.30369700	-2.20852700	-0.44868200
H	-3.82918000	3.14901700	3.72379400
H	-4.91442300	-0.56743200	1.14893800
H	-2.37639300	-0.08748700	2.64453900
O	-0.82352400	-1.65586700	-0.55106500
H	-5.47484200	-1.70514300	-2.84506200
O	-3.74392700	1.29460700	2.04949200
H	-7.13380700	-0.60385300	0.79226700
H	-1.16615900	-1.67230700	1.45120100

B7

C	-0.30719200	-2.14203300	1.41576200
C	-1.49665500	-2.12528400	0.43143000
C	-2.55479100	-1.13101700	0.91262200
C	-1.05554900	0.12817100	2.19784400
C	0.14123000	-0.72189600	1.78575800
H	-1.14382500	-1.79511200	-0.53980200
H	-0.63921100	-2.62935400	2.33208700
H	-1.51227700	-0.29386900	3.09099300
H	-3.00352900	-1.49005800	1.83724500
O	-1.97777800	0.10939000	1.13642100
O	0.81441200	-2.78630600	0.90384400
C	-0.74013300	1.58175600	2.47416800
H	0.27773945	1.66866066	2.84288671
H	-1.41642457	1.96314700	3.22633249
H	0.55648600	-3.50529800	0.32936800
O	-0.89313010	2.27022069	1.25647648
H	-1.58176089	1.87154159	0.74394887
C	-5.15364300	0.63457500	0.78151300
C	-6.67199000	0.82693100	0.92812200
C	-4.85477200	-0.79273300	0.32695300
H	-4.76363900	1.32215000	0.04159000
C	-7.47546100	0.26625400	-0.24660300
H	-6.97729800	0.24874400	1.79106900
C	-5.70526200	-1.15270900	-0.88570000
H	-5.10420100	-1.46875900	1.13708800
H	-7.35984100	0.87385900	-1.13321100
H	-5.48248900	-0.46805300	-1.70289500
O	-7.05766300	-1.04324100	-0.51558600
O	-8.79566800	0.22281200	0.14869600
O	-3.51561500	-0.99616100	-0.05987800
C	-5.48105100	-2.57072000	-1.37902900
H	-6.20567900	-2.78724300	-2.15770600
H	-4.49108900	-2.65647400	-1.79876400
O	-5.56512200	-3.51803500	-0.34511400
H	-6.44155600	-3.49674800	0.01909200
O	-4.61877200	0.89711900	2.04674400
H	-3.72788000	1.22563700	1.98396600
C	-9.79705600	0.17572600	-0.77168800
C	-9.63521100	-0.26669400	-2.07412000
C	-11.04477300	0.59120400	-0.32535800
C	-10.72882900	-0.27255100	-2.92963300
H	-8.68327400	-0.62000900	-2.41703300
C	-12.12555100	0.56992900	-1.18461200
H	-11.14418900	0.92713300	0.69008600
C	-11.97418200	0.14161600	-2.49643900

H	-10.59744200	-0.61387400	-3.94105900
H	-13.08820800	0.89425900	-0.83134900
H	-12.81522300	0.12953100	-3.16555900
H	0.62073500	-0.27782000	0.92843100
N	-7.02181500	2.20200800	1.22008600
H	-7.25108500	2.40736900	2.16413000
C	-7.07783700	4.60884800	0.96603300
H	-6.22332300	4.92684400	1.55575200
H	-7.22016000	5.31024700	0.15706300
H	-7.95175300	4.60765800	1.60740800
N	-2.16845200	-3.41856900	0.30482400
H	-3.16084600	-3.40550900	0.40171800
C	-2.60639700	-5.65030100	-0.54964000
H	-2.41675200	-6.06605600	-1.53018100
H	-2.37670000	-6.41661100	0.18332500
H	-3.64868500	-5.37656700	-0.45915200
C	-1.65890800	-4.49290900	-0.33294400
O	-0.50452100	-4.56591300	-0.68406600
C	-6.79792700	3.24038700	0.38333900
O	-6.39499400	3.09756000	-0.74195500
C	7.67975500	-1.98532200	-0.61929300
C	7.15363800	-2.82918800	0.53462200
C	5.69496600	-2.47768800	0.81201200
C	5.31915000	-1.73309300	-1.41316400
C	6.75283100	-2.05629200	-1.82661600
H	7.74049800	-0.94855200	-0.29421800
H	5.27508600	-0.69561600	-1.10612400
H	6.79809100	-3.05415100	-2.24225400
O	4.92591300	-2.55814600	-0.33594600
C	4.65140300	-0.76239600	2.19486900
C	3.22021200	-1.04620600	1.73308000
C	4.87070900	0.74528200	2.29740600
C	2.34010900	-0.42608700	2.82505900
H	3.01972900	-2.09976100	1.64420200
C	3.67645500	1.56852300	2.83032300
H	3.84726700	1.75293600	3.88588600
O	2.41117600	0.96479700	2.66134700
C	6.83969000	4.16122200	-1.20256700
C	5.62733400	3.26587400	-1.00413900
C	5.11228200	2.65474100	-2.30320000
C	3.36525600	3.58559300	-0.21937200
C	3.77121000	1.98043300	-2.07680800
H	5.00693000	3.43708300	-3.04246600
C	2.77935700	2.97463700	-1.48682500
H	2.76729800	4.42541700	0.10883500

H	3.91197700	1.16569300	-1.38245900
O	4.63983500	4.09714600	-0.41510600
O	6.05999800	1.70572900	-2.74393000
H	5.77564600	1.40641200	-3.59917800
O	3.30073500	1.48163200	-3.30126800
H	3.02616700	0.57626600	-3.16953600
O	2.51674000	4.01655000	-2.37905000
H	2.30162800	3.63161400	-3.22055300
O	5.95794700	1.06493300	3.12311800
H	6.74036000	0.67309900	2.75684900
O	2.95644800	-0.46857500	0.48338100
H	2.84984800	0.47227000	0.57791200
O	7.20057200	-4.18780300	0.21960200
H	8.06061900	-4.37457600	-0.13678600
O	8.95906000	-2.46305800	-0.93941300
H	9.21433900	-2.09689500	-1.77772300
O	7.21715700	-1.18920000	-2.83253300
H	7.07057400	-0.28417600	-2.57441100
H	5.27248300	-3.19333500	1.50365400
C	4.31332900	-1.94904800	-2.53219400
H	4.72412500	-1.58549800	-3.46324600
C	3.60439200	2.88988100	2.07766000
O	3.11499200	-1.24648900	-2.31959500
H	2.83233700	-1.35993700	-1.41592600
H	4.12196300	-3.01237600	-2.63204800
H	7.63386500	3.59793400	-1.67103500
H	7.18488500	4.49690700	-0.22725200
O	6.55673100	5.24690800	-2.03296000
H	5.80204400	5.70160600	-1.68053200
H	4.54125700	3.42411400	2.17584700
H	2.79998200	3.50316100	2.47072500
O	3.34719500	2.56854300	0.73129800
H	7.73641800	-2.62332600	1.42802700
H	5.07695400	1.06538100	1.28773500
H	4.79556100	-1.22193700	3.16955500
O	1.02575800	-0.81595900	2.88345700
H	1.85919300	2.46107500	-1.22140700
O	5.68914300	-1.19001100	1.34313700
H	5.89082700	2.46329300	-0.32179600
H	2.73191000	-0.69635900	3.79923100
B8			
C	0.86020900	0.96322200	-1.71778000
C	2.37997900	1.18715500	-1.90248800
C	3.19422100	0.31424000	-0.94749700
C	1.48653100	-1.22020200	-0.57155600

C	0.50916800	-0.51816400	-1.50485800
H	2.63542900	0.88541600	-2.91270100
H	0.52303200	1.51584500	-0.84648100
H	1.38552200	-0.80843400	0.43009000
H	3.06277100	0.65684200	0.07575900
O	2.78679200	-1.00705100	-1.04931100
O	0.15741600	1.34516200	-2.86009900
C	1.28497900	-2.71807700	-0.48900100
H	0.22086721	-2.93806830	-0.49005479
H	1.72418502	-3.09161028	0.42752746
H	0.26929200	2.28864700	-2.96647800
O	1.91129677	-3.27823715	-1.61572019
H	2.56861306	-2.68508073	-1.95091611
C	5.70950700	-1.04725600	0.29592400
C	6.96086100	-1.04294800	1.18992400
C	5.50785300	0.34077600	-0.30935700
H	5.83156000	-1.76142800	-0.50815100
C	8.17062700	-0.36105000	0.54754800
H	6.71896000	-0.44698500	2.06083000
C	6.79997000	0.81511500	-0.96534400
H	5.24883100	1.02973400	0.48689100
H	8.59391000	-0.96654400	-0.24180000
H	7.09550600	0.10738000	-1.73874600
O	7.78937300	0.88412500	0.03156700
O	9.09370500	-0.14673200	1.54885000
O	4.51871100	0.39108900	-1.31100300
C	6.69124200	2.18976000	-1.60046100
H	7.68030400	2.50431000	-1.91842600
H	6.05368200	2.13856000	-2.46942400
O	6.12063800	3.13909200	-0.73742300
H	6.67961900	3.23814600	0.02341700
O	4.64615900	-1.40671600	1.13108000
H	3.99293200	-1.90874900	0.65568500
C	10.40877000	0.04497900	1.25460300
C	10.87418300	0.47959900	0.02471800
C	11.30006300	-0.20767700	2.28912700
C	12.24007200	0.64170400	-0.16516000
H	10.19076000	0.70861800	-0.76845500
C	12.65499400	-0.03194700	2.08872400
H	10.91317800	-0.54085400	3.23420100
C	13.13591400	0.39015700	0.85672700
H	12.59705100	0.97639400	-1.12291100
H	13.33872500	-0.23057800	2.89476500
H	14.19105200	0.52256200	0.70070100
H	0.53718700	-0.99920100	-2.47870500

N	7.28330400	-2.36597800	1.68347500
H	7.05530800	-2.55219200	2.63175400
C	7.76198400	-4.73785200	1.61263000
H	6.77583100	-5.17579800	1.73409700
H	8.36372400	-5.40147900	1.00921600
H	8.20873400	-4.63004100	2.59440600
N	2.82388400	2.56045600	-1.65469300
H	3.76420700	2.63450900	-1.33100900
C	3.04656600	4.96911700	-1.74046100
H	3.10636100	5.55874800	-2.64662100
H	2.49349300	5.54904300	-1.01029800
H	4.04179900	4.77556400	-1.36490300
C	2.25447400	3.71645600	-2.04437000
O	1.17183000	3.80320600	-2.57845200
C	7.63223600	-3.40995600	0.89833900
O	7.81696100	-3.30271900	-0.28649100
C	-8.06114300	-2.40364100	-1.25501200
C	-7.02501900	-2.96196000	-2.22227900
C	-5.62174000	-2.60726300	-1.74019600
C	-6.30222100	-2.44287200	0.52817200
C	-7.74712100	-2.79575400	0.18374000
H	-8.05364800	-1.31787900	-1.32396600
H	-6.19890400	-1.36490100	0.52301400
H	-7.89911700	-3.86133300	0.29395400
O	-5.42334500	-3.00343100	-0.42729700
C	-4.22782600	-0.61938600	-2.05652300
C	-3.07825700	-1.11771800	-1.17861800
C	-4.50311200	0.85222300	-1.76746100
C	-1.88675300	-0.23123200	-1.54223200
H	-2.84918300	-2.15437900	-1.38124400
C	-3.25419100	1.73119200	-1.57485400
H	-2.97441200	2.15239600	-2.53472000
O	-2.15916200	1.03666200	-1.02524400
C	-7.98455800	3.38628100	1.13179200
C	-6.73273100	2.53439000	1.26776300
C	-6.78503400	1.57849800	2.45564200
C	-4.39348800	2.96627700	1.68065100
C	-5.42465100	0.94886600	2.69733600
H	-7.08964000	2.12958000	3.33488600
C	-4.37415200	2.03555900	2.88779400
H	-3.78373300	3.84176300	1.86110500
H	-5.16214000	0.35286700	1.83555000
O	-5.66670900	3.45657800	1.41467700
O	-7.73751700	0.57428000	2.17095100
H	-7.83609500	0.05695500	2.96105600

O	-5.50194400	0.12807200	3.83536800
H	-5.13580500	-0.72534700	3.61546800
O	-4.63712800	2.79905700	4.02820300
H	-4.77814100	2.20086300	4.75236200
O	-5.25516900	1.45129800	-2.78723000
H	-6.08015700	0.99014500	-2.86507300
O	-3.34463500	-1.00248800	0.19183400
H	-3.08108700	-0.13353300	0.47973300
O	-7.10189100	-4.35428300	-2.28603100
H	-8.01451300	-4.59011200	-2.40085100
O	-9.31193900	-2.90095300	-1.64883200
H	-9.93585700	-2.72894600	-0.95375600
O	-8.65998000	-2.17552500	1.05549800
H	-8.47800300	-1.24184000	1.10642200
H	-4.88946300	-3.14393400	-2.32769300
C	-5.87171900	-2.96046000	1.88993900
H	-6.66391100	-2.79794200	2.60661900
C	-3.56961500	2.84424800	-0.58510300
O	-4.73592900	-2.29302500	2.37914900
H	-4.11585500	-2.15819200	1.66702200
H	-5.68774400	-4.02785500	1.81801400
H	-8.85130400	2.74756800	1.03865900
H	-7.89864600	3.98395300	0.22724300
O	-8.18698800	4.19827200	2.24931300
H	-7.39393900	4.69774100	2.39888300
H	-4.40222300	3.43561800	-0.94503500
H	-2.70296600	3.48360600	-0.45540800
O	-3.88856800	2.21220300	0.63165500
H	-7.17549900	-2.52493000	-3.20521500
H	-5.06357000	0.85533400	-0.84618000
H	-3.93000100	-0.74122800	-3.09555600
O	-0.75777300	-0.70522400	-0.91710000
H	-3.38742600	1.58545400	2.95104600
O	-5.48752100	-1.23097700	-1.88318500
H	-6.60109000	1.95673000	0.35809400
H	-1.74284300	-0.17955000	-2.61360900
B9			
C	1.28662600	-3.54311200	-1.62044300
C	2.66665700	-3.04009600	-1.17063100
C	3.24091700	-2.07977400	-2.22194100
C	1.06515400	-1.45115300	-2.96744800
C	0.40231000	-2.33821000	-1.91736300
H	2.50980800	-2.48773700	-0.25122000
H	1.37733700	-4.12987800	-2.52627900
H	1.17547600	-2.01821800	-3.89138500

H	3.46779500	-2.63856000	-3.12442500
O	2.33808900	-1.06244800	-2.49591800
O	0.67753000	-4.29359100	-0.61834300
C	0.30371400	-0.17308000	-3.27812200
H	-0.65314400	-0.42351000	-3.70520900
H	0.87248000	0.39439000	-4.01030400
H	1.01269100	-5.18063700	-0.69986900
O	0.05903900	0.59696400	-2.13586400
H	0.88198000	0.96387800	-1.82460000
C	5.58156300	-1.00722600	0.16226900
C	5.89693900	0.08452100	1.19392100
C	4.51609400	-0.53643300	-0.83499700
H	6.48936100	-1.21279800	-0.40056400
C	6.24493500	1.37303100	0.45582900
H	5.01647300	0.28123700	1.79527800
C	4.92877800	0.81224000	-1.43474700
H	3.56677000	-0.42676000	-0.33030700
H	7.15737300	1.24133200	-0.12618100
H	5.82461600	0.66086800	-2.03585400
O	5.20461700	1.72362100	-0.38584400
O	6.42700300	2.36212400	1.39664200
O	4.45130300	-1.49777100	-1.86296000
C	3.88606500	1.50646900	-2.29256300
H	4.33051800	2.41153300	-2.69737700
H	3.59038400	0.86882200	-3.10600200
O	2.73020800	1.81877300	-1.56392300
H	2.96039800	2.40936400	-0.85789600
O	5.15408600	-2.19842600	0.75352600
H	5.19364700	-2.15161300	1.70708700
C	7.08963100	3.51557500	1.08447800
C	7.31936700	3.95630600	-0.20734700
C	7.53997100	4.25481700	2.16991000
C	8.02098800	5.13922800	-0.40288300
H	6.94095100	3.41301800	-1.04986100
C	8.22808200	5.43330900	1.95924800
H	7.33876500	3.89505300	3.16198400
C	8.47832100	5.88127400	0.66912900
H	8.19768300	5.47978600	-1.40737800
H	8.57269500	6.00234900	2.80393300
H	9.01562000	6.79756500	0.50654900
H	0.26513800	-1.77454200	-1.01171900
N	7.00733000	-0.26694700	2.06926200
H	7.78313800	0.35670900	2.06807400
C	7.97788300	-0.99214800	4.17529500
H	7.80688500	-0.18538000	4.88116600

H	7.98789000	-1.92488200	4.72001700
H	8.93808700	-0.83478800	3.69882800
N	3.65150600	-4.07102300	-0.88304900
H	4.44274500	-3.70667200	-0.39842500
C	4.82781100	-6.15505500	-0.59394600
H	4.53048400	-6.90818200	0.12533500
H	5.23941500	-6.66885000	-1.45471400
H	5.58997900	-5.52189500	-0.15820400
C	3.58975900	-5.40202200	-1.03696100
O	2.64868600	-6.01200800	-1.49471600
C	6.84033300	-1.01639100	3.18301800
O	5.83873700	-1.65960000	3.38091800
C	-4.38265300	-1.57416200	4.19101400
C	-3.27674600	-0.91439300	3.37862400
C	-3.71845900	-0.72129900	1.93357700
C	-6.02802900	-0.66604900	2.53633900
C	-5.69836900	-0.82647900	4.01853100
H	-4.51034600	-2.59684200	3.84156200
H	-6.19630700	-1.64489800	2.09522600
H	-5.60380000	0.15014300	4.47263700
O	-4.94541000	-0.04162000	1.87833300
C	-3.61610700	-2.16331100	-0.03804300
C	-2.12722300	-2.18289400	-0.39636500
C	-4.37756200	-1.15770300	-0.90219300
C	-2.04856600	-2.51611700	-1.88704300
H	-1.62455900	-2.94453500	0.18461800
C	-3.97206200	-1.24309500	-2.38573800
H	-4.51229900	-2.07658400	-2.83010500
O	-2.59117800	-1.42632600	-2.57720900
C	-7.17406100	3.05767700	-1.13147500
C	-5.72278600	2.59201300	-1.17842800
C	-4.80588000	3.43621500	-0.28795600
C	-4.01656200	2.38842800	-2.82718400
C	-3.33135800	3.17895300	-0.56858900
H	-4.99973000	4.48354700	-0.47693000
C	-3.04175100	3.27488300	-2.06241300
H	-3.92308300	2.55205900	-3.89283400
H	-3.06506900	2.18379900	-0.22533400
O	-5.34115200	2.69404000	-2.52748300
O	-5.09513300	3.23689600	1.08258000
H	-4.93971800	2.32762600	1.32588800
O	-2.53326600	4.13872900	0.07401700
H	-2.78361700	4.18207600	0.98851200
O	-3.21350000	4.58539600	-2.51829400
H	-2.71540200	5.15739600	-1.94773500
O	-5.75603400	-1.38661100	-0.87108900
H	-6.19434100	-0.85008400	-0.21768800
O	-1.53695100	-0.94601900	-0.08741000
H	-1.43269300	-0.39781800	-0.86142300
O	-2.98960200	0.35672700	3.88954300
H	-2.84448900	0.26730300	4.82337100
O	-3.97896100	-1.56897500	5.53510800
H	-4.73063200	-1.77113000	6.07843600
O	-6.72546100	-1.47885100	4.73001600
H	-6.90745700	-2.32116100	4.33156500
H	-3.01121500	-0.09107400	1.42962900
C	-7.25407300	0.18549600	2.28194200
H	-8.08798900	-0.19382900	2.86113300
C	-4.35264200	0.03148100	-3.11789900
O	-7.54712400	0.14146200	0.91247500
H	-7.82835800	1.00218900	0.61355100
H	-7.05237900	1.20209000	2.60588600
H	-7.75540600	2.47274900	-1.82968200
H	-7.23116400	4.09797000	-1.43636400
O	-7.75527400	2.87756000	0.13786700
H	-7.20808700	3.30440200	0.78773800
H	-5.42530400	0.16639300	-3.08128600
H	-4.03697200	-0.03344000	-4.15455400
O	-3.68887800	1.09174700	-2.47915000
H	-2.39171900	-1.54120400	3.38745900
H	-4.14294200	-0.16622000	-0.55487800
H	-4.03131500	-3.14609900	-0.22483900
O	-0.81960400	-2.81089100	-2.43000600
H	-2.03429900	2.92263600	-2.25519800
O	-3.81160800	-1.97676200	1.35324800
H	-5.68172100	1.55858200	-0.85265600
H	-2.65625700	-3.39463500	-2.08059500
B10			
C	1.17792700	-0.98145300	-0.03920800
C	2.67950800	-1.30333600	-0.00996800
C	3.34946100	-0.77975200	-1.28390300
C	1.77132800	0.98296500	-1.50866000
C	0.96817100	0.50956900	-0.29869300
H	3.09968000	-0.77069400	0.83599900
H	0.69186000	-1.53094700	-0.83429600
H	1.32887200	0.53902200	-2.39893900
H	2.95227200	-1.31157400	-2.14301700
O	3.11746400	0.58139900	-1.40103000
O	0.60366700	-1.29749600	1.19341600
C	1.78764100	2.49716100	-1.66283000

H	0.77096100	2.85841200	-1.71059500
H	2.27832900	2.74174300	-2.60224500
H	0.38184700	-2.22263000	1.15551800
O	2.41645900	3.12656200	-0.59055100
H	3.35356100	2.97225500	-0.65507900
C	6.48286400	-1.28724800	0.21472600
C	7.64624500	-0.59398900	0.93676800
C	5.61435900	-0.27170900	-0.53655000
H	6.90101700	-1.96990700	-0.52162700
C	8.40669800	0.26539100	-0.06808300
H	7.25628300	0.06795900	1.70179100
C	6.49853600	0.59887700	-1.43574600
H	5.09120700	0.35623800	0.17047700
H	8.86257400	-0.36176900	-0.83447600
H	6.92203000	-0.03415500	-2.21453500
O	7.53989900	1.16612000	-0.66093700
O	9.38293000	0.95263000	0.61821000
O	4.72056800	-0.99944800	-1.34706300
C	5.81190600	1.78127100	-2.09574700
H	6.52686300	2.26140900	-2.75825900
H	4.97030700	1.44645200	-2.67541600
O	5.33213100	2.69995500	-1.15311400
H	6.06307400	3.03002100	-0.64624500
O	5.67249000	-2.02332500	1.08231300
H	6.01620000	-2.01922900	1.97391600
C	10.44256200	1.51167600	-0.03914000
C	10.47127600	1.75872500	-1.40073300
C	11.53341200	1.83355200	0.75695500
C	11.61262000	2.31738800	-1.96201500
H	9.61921700	1.55024100	-2.01616400
C	12.65724500	2.39678200	0.18548600
H	11.48042300	1.64064800	1.81247100
C	12.70639000	2.63779400	-1.18095600
H	11.63241800	2.50873100	-3.01993000
H	13.49828900	2.64549300	0.80744000
H	13.58235400	3.07420200	-1.62472000
H	1.26869800	1.06996300	0.57977500
N	8.57585700	-1.53557400	1.54615800
H	9.52207400	-1.48041300	1.24209900
C	9.56169300	-2.71706800	3.42668600
H	10.09251900	-1.97101700	4.00977200
H	9.21500000	-3.48981400	4.09712700
H	10.24759900	-3.13983000	2.70243600
N	3.00588500	-2.71082200	0.16246600
H	3.97255900	-2.84420100	0.36581400

C	2.95173600	-5.10300500	0.44615700
H	2.52563700	-5.60124200	1.30800900
H	2.77315500	-5.73263100	-0.41773700
H	4.01851600	-4.98639800	0.58734800
C	2.21774700	-3.79367400	0.23916500
O	1.00935900	-3.79339800	0.14762300
C	8.36793300	-2.06227600	2.77466000
O	7.29551900	-2.00473700	3.32511000
C	-6.74963300	4.35918200	0.89664600
C	-6.03349400	3.70611000	-0.27782900
C	-5.57926100	2.29884700	0.08783000
C	-7.27884400	2.06649800	1.74480500
C	-7.84664300	3.44899600	1.43376000
H	-6.02626700	4.54248100	1.68905500
H	-6.54461100	2.14904000	2.54182800
H	-8.62147500	3.35929100	0.68512900
O	-6.65224000	1.54644600	0.59003700
C	-3.54647600	1.42730400	1.11436600
C	-2.44995300	1.68184600	0.07793800
C	-4.06981900	-0.01048700	1.06440700
C	-1.37709900	0.61725500	0.26573900
H	-2.03347100	2.66852800	0.23698600
C	-2.92639500	-1.02764600	0.91583800
H	-2.44894700	-1.15084400	1.88511000
O	-1.97175900	-0.61338600	-0.02777700
C	-7.98648600	-3.15432400	0.74877000
C	-6.90916800	-2.51773300	-0.12317100
C	-7.46994500	-1.96930700	-1.43933700
C	-4.91715000	-3.24130100	-1.21622400
C	-6.37200800	-1.63497900	-2.43974700
H	-8.10959400	-2.71681900	-1.88904400
C	-5.40982800	-2.80710200	-2.59084700
H	-4.34584200	-4.15715800	-1.29012000
H	-5.81762500	-0.76914000	-2.08910400
O	-5.97576800	-3.54114200	-0.36414200
O	-8.29780300	-0.84384300	-1.21751000
H	-7.79694100	-0.13908300	-0.81479100
O	-6.91735400	-1.37672700	-3.70655700
H	-7.61523000	-0.73960200	-3.61666300
O	-6.04802500	-3.90089300	-3.18024100
H	-6.47831800	-3.59560600	-3.96937600
O	-4.74153200	-0.35655100	2.23923300
H	-5.67049100	-0.15086800	2.19844800
O	-2.94765300	1.64239900	-1.23260400
H	-2.79878800	0.77361400	-1.58753500

O	-6.90191900	3.58056900	-1.36802400
H	-7.29457500	4.43033000	-1.52555900
O	-7.28577100	5.57317700	0.44116100
H	-7.91439300	5.88615100	1.07990100
O	-8.46521300	4.04142900	2.55303800
H	-7.85120700	4.08192600	3.27602900
H	-5.25720600	1.79417600	-0.80344800
C	-8.32568600	1.06089500	2.17454300
H	-8.91589900	1.47215500	2.98545300
C	-3.45006100	-2.37953100	0.46547800
O	-7.66572700	-0.10375700	2.58619000
H	-8.16398400	-0.87305000	2.32282900
H	-8.99121200	0.86153300	1.33982900
H	-7.52520000	-3.56975300	1.63303600
H	-8.45872600	-3.96554400	0.20352700
O	-8.94716900	-2.22312200	1.18659200
H	-9.30119200	-1.76796000	0.43091700
H	-4.10622900	-2.78355300	1.22389500
H	-2.61657600	-3.05698700	0.31185000
O	-4.14436900	-2.19530300	-0.74596000
H	-5.16026700	4.29025800	-0.54680300
H	-4.71179800	-0.11685400	0.20547900
H	-3.12053800	1.58100400	2.09908700
O	-0.36275600	0.82220900	-0.63804900
H	-4.55386700	-2.49597700	-3.18101700
O	-4.56621600	2.40868200	1.02651200
H	-6.44591100	-1.70976600	0.43138900
H	-1.00078300	0.61591100	1.28159400

B11

C	-1.07246100	2.60763500	-1.62171700
C	-2.28911700	2.53190800	-0.68214300
C	-3.05149600	1.22707100	-0.91568100
C	-1.14599800	0.09608600	-1.77097100
C	-0.25983700	1.31751700	-1.55276800
H	-1.94628300	2.53555500	0.34617300
H	-1.43865200	2.72643300	-2.64059200
H	-1.57336900	0.12764600	-2.77190200
H	-3.48896200	1.22893500	-1.91303700
O	-2.18898400	0.13305100	-0.80904600
O	-0.20835200	3.65751000	-1.31552900
C	-0.43344100	-1.23410300	-1.59797700
H	0.32384000	-1.33770100	-2.35638800
H	-1.15861300	-2.03542700	-1.71823300
H	-0.62646600	4.28882300	-0.73561700
O	0.21965200	-1.33979300	-0.36151400
H	-0.40823800	-1.21731400	0.33916000
C	-5.27049100	-1.00047900	-0.11810200
C	-6.67850100	-1.55935000	-0.35895400
C	-5.26684500	0.50951800	-0.32564200
H	-4.96217900	-1.20670900	0.89991900
C	-7.76828600	-0.74840800	0.34854800
H	-6.88082600	-1.46307600	-1.41820500
C	-6.35923700	1.13999200	0.52999000
H	-5.48084200	0.72350100	-1.36602400
H	-7.75498900	-0.91796300	1.41593300
H	-6.19799700	0.88797100	1.57712500
O	-7.58835000	0.61736300	0.08762700
O	-8.97413700	-1.13289200	-0.19539900
O	-4.03646800	1.09794200	0.03461600
C	-6.43164300	2.65010100	0.41343300
H	-7.31771300	2.99881500	0.93385500
H	-5.56460200	3.08868800	0.88151100
O	-6.43472000	3.08818000	-0.92203000
H	-7.22229600	2.77357000	-1.34869100
O	-4.42998200	-1.64998400	-1.02927500
H	-3.53415800	-1.38166300	-0.86111200
C	-10.13877800	-0.93635600	0.48270400
C	-10.30416900	0.00555600	1.48423600
C	-11.20213700	-1.74221100	0.09827200
C	-11.53857800	0.12107600	2.10951000
H	-9.49765900	0.65349700	1.76435000
C	-12.42695800	-1.60871700	0.72194000
H	-11.04806100	-2.46306300	-0.68303900
C	-12.60287400	-0.67787900	1.73676000
H	-11.66166300	0.85145100	2.88929100
H	-13.24550600	-2.23721200	0.41940000
H	-13.55543600	-0.57802800	2.22438400
H	0.19215500	1.25750800	-0.57880700
N	-6.77024200	-2.97226400	-0.05512400
H	-6.79127600	-3.59130400	-0.83127900
C	-6.52468400	-5.01395600	1.22494200
H	-5.52952400	-5.35344000	0.95428100
H	-6.73572400	-5.33369600	2.23482800
H	-7.23576900	-5.46460800	0.54213000
N	-3.21782900	3.63891000	-0.87846100
H	-4.13266700	3.42882200	-1.21562800
C	-4.15328000	5.85262000	-0.49754600
H	-4.32212600	6.32484900	0.46184600
H	-3.83568600	6.62419000	-1.19000600
H	-5.07187800	5.40806000	-0.85494100

C	-3.02272700	4.86204700	-0.34697800
O	-1.99092200	5.17418100	0.20078900
C	-6.56253700	-3.50265200	1.17283200
O	-6.40680500	-2.82582000	2.15581000
C	6.27292000	3.26200300	2.27058100
C	5.02372500	2.39154500	2.29082100
C	4.99881900	1.46337400	1.08331700
C	7.37599700	1.50349800	0.87085200
C	7.52213500	2.40910100	2.09141200
H	6.19992400	3.95875000	1.43764000
H	7.31326900	2.11323500	-0.02654900
H	7.65597900	1.80093100	2.97522100
O	6.19558000	0.73648300	0.99245100
C	4.15858700	1.72651700	-1.19653200
C	2.63602500	1.71432200	-1.03074400
C	4.67988200	0.35246100	-1.62125500
C	2.06126700	1.25670600	-2.37150600
H	2.28709000	2.70849600	-0.79002000
C	3.81354300	-0.28273000	-2.72552300
H	4.11285300	0.15458800	-3.67557800
O	2.43306900	-0.08300100	-2.53535400
C	7.60116900	-3.64066100	-0.57806400
C	6.17835000	-3.12931100	-0.37898200
C	5.67805700	-3.32653700	1.05522000
C	4.01854600	-3.62679900	-1.25424500
C	4.17578400	-3.10885100	1.17643700
H	5.89085300	-4.34068600	1.36562800
C	3.42734900	-3.91405500	0.12005200
H	3.60125400	-4.29724200	-1.99416600
H	3.95099300	-2.05547000	1.03673900
O	5.38997300	-3.85670500	-1.28829700
O	6.37700800	-2.49606400	1.96176600
H	6.22936400	-1.57716400	1.75230300
O	3.71352300	-3.53714100	2.43009200
H	4.24871300	-3.14178400	3.10705800
O	3.55579900	-5.28526000	0.35718100
H	3.32738000	-5.44525500	1.26455100
O	5.97061100	0.42763200	-2.15030800
H	6.63958700	0.26376900	-1.49219200
O	2.26114400	0.86403600	0.02347900
H	2.03005700	-0.00257300	-0.29974800
O	5.01693400	1.57540500	3.42763500
H	5.16408100	2.12988700	4.18401400
O	6.31686900	3.96250100	3.48529200
H	7.18217900	4.33886000	3.58865800
O	8.66552100	3.22945000	2.01742400
H	8.64057800	3.74545800	1.22087600
H	4.22232900	0.73464000	1.21667700
C	8.52330600	0.52990400	0.70245000
H	9.46354000	1.06878200	0.71480500
C	4.03892500	-1.78280500	-2.78621900
O	8.35753300	-0.14116700	-0.51654700
H	8.60599200	-1.05657300	-0.41854200
H	8.52214100	-0.16680900	1.53527900
H	7.86968200	-3.54055100	-1.61991400
H	7.64732500	-4.69394400	-0.31957600
O	8.54518700	-2.90369200	0.16097400
H	8.28355200	-2.90000000	1.07495100
H	5.07366700	-1.98498400	-3.02717900
H	3.40388300	-2.21825700	-3.55161000
O	3.70243900	-2.30782900	-1.52730200
H	4.13945600	3.01881200	2.26206100
H	4.65110900	-0.30096700	-0.76609800
H	4.41618200	2.43510500	-1.97418000
O	0.70402300	1.36447000	-2.57647200
H	2.38455300	-3.61530500	0.11441600
O	4.79871900	2.24491900	-0.04434300
H	6.15462000	-2.07277300	-0.62180800
H	2.50671200	1.84697500	-3.16562500
B12			
C	-1.34192300	-1.93952900	-1.08785000
C	-2.80589600	-1.71293800	-1.51587400
C	-3.52293100	-0.81912800	-0.50273400
C	-1.52323100	0.18606900	0.28478500
C	-0.66373100	-0.63722900	-0.66320600
H	-2.82127400	-1.20783300	-2.47510100
H	-1.34303700	-2.60989300	-0.22995800
H	-1.61945400	-0.33678900	1.23437400
H	-3.61706400	-1.34119300	0.44833800
O	-2.80402800	0.35935600	-0.29698900
O	-0.55340500	-2.46285700	-2.11173100
C	-0.97012600	1.57882300	0.53975200
H	0.01368000	1.50363000	0.97510900
H	-1.62055000	2.09018900	1.24618000
H	-1.07084900	-3.01834100	-2.69067100
O	-0.84155300	2.31583300	-0.64300800
H	-1.69365100	2.39549200	-1.05144900
C	-5.98383500	1.04499800	0.46679400
C	-7.27467300	1.16261100	1.28728500
C	-5.84890000	-0.36614500	-0.09384600

H	-6.01084900	1.74157500	-0.36258700
C	-8.49115300	0.57072600	0.56958600
H	-7.13890100	0.56395500	2.17911300
C	-7.12225200	-0.73501400	-0.84625600
H	-5.72147400	-1.06159000	0.72725700
H	-8.81219000	1.20162700	-0.24727700
H	-7.30069800	-0.01385700	-1.64256800
O	-8.17878200	-0.70621500	0.08200300
O	-9.48891000	0.43782300	1.50980200
O	-4.76617500	-0.49027700	-0.99024300
C	-7.08865700	-2.12064700	-1.46140000
H	-8.07534400	-2.35616600	-1.84668200
H	-6.38849400	-2.13436700	-2.28188900
O	-6.66271100	-3.10243000	-0.55062800
H	-7.29262400	-3.16196800	0.15722800
O	-4.93609400	1.35780000	1.34025500
H	-4.12533300	1.38183600	0.84588500
C	-10.79530600	0.35670000	1.13292600
C	-11.21681600	-0.04722000	-0.12274400
C	-11.72480500	0.69299900	2.10797500
C	-12.57680400	-0.09426400	-0.39899100
H	-10.50659500	-0.33966700	-0.87030200
C	-13.07441000	0.63127400	1.82203600
H	-11.37148200	1.00035600	3.07467000
C	-13.51052500	0.24110100	0.56313500
H	-12.89982500	-0.40518800	-1.37655400
H	-13.78836200	0.89421300	2.58208000
H	-14.56108600	0.19767800	0.34055900
H	-0.47446400	-0.04855700	-1.55208300
N	-7.51800200	2.51625900	1.74022800
H	-7.33442800	2.70243900	2.69821100
C	-7.76934500	4.92100100	1.59729400
H	-6.75634800	5.27806500	1.75606100
H	-8.28429100	5.61778700	0.95237400
H	-8.26839300	4.87437800	2.55841300
N	-3.56555900	-2.95766500	-1.60688400
H	-4.37399900	-3.04611800	-1.02950900
C	-4.43590500	-4.98160000	-2.63801800
H	-4.84411200	-5.05520600	-3.63817500
H	-3.89860000	-5.90155400	-2.43714000
H	-5.23828900	-4.87105900	-1.92209200
C	-3.44266300	-3.84328200	-2.61526300
O	-2.57431800	-3.76451600	-3.45326800
C	-7.71728600	3.57137000	0.91616600
O	-7.83404500	3.45251900	-0.27592200
C	5.81604400	4.15119100	-1.65924900
C	5.08120400	3.58460800	-0.45179200
C	5.12929500	2.06207900	-0.46106400
C	7.13336300	2.02718500	-1.75339900
C	7.21177400	3.55158600	-1.77238600
H	5.25380000	3.90447300	-2.55802600
H	6.58950200	1.68277000	-2.62899800
H	7.80493900	3.89128700	-0.93475400
O	6.45035300	1.60731900	-0.58944400
C	3.68888400	0.36350900	-1.45283900
C	2.38482900	0.45827500	-0.66061100
C	4.57825700	-0.77490300	-0.95066900
C	1.71262500	-0.91147500	-0.69727700
H	1.75270900	1.21437200	-1.10814400
C	3.76749700	-2.05452700	-0.68212700
H	3.57070100	-2.53675800	-1.63686400
O	2.54873700	-1.79833500	-0.02657300
C	9.06349600	-2.30701000	0.74814300
C	7.69039500	-1.87775900	1.25483100
C	7.77914700	-0.89516200	2.42692300
C	5.79630400	-2.94884600	2.23226000
C	6.44304900	-0.72121100	3.13588200
H	8.49001600	-1.27176300	3.15007600
C	5.83895400	-2.07718100	3.48089500
H	5.50601700	-3.95975500	2.48648900
H	5.75745000	-0.18709400	2.48400000
O	7.05159600	-3.06822300	1.64579400
O	8.28638400	0.35782200	2.01131800
H	7.70686600	0.75151700	1.36409600
O	6.60729000	-0.01998500	4.33967700
H	7.10388100	0.77104800	4.16994300
O	6.61524600	-2.73921000	4.43441300
H	6.76391100	-2.14023700	5.15575100
O	5.54683700	-1.13134600	-1.89106300
H	6.35417100	-0.63709100	-1.78447900
O	2.60190100	0.83843200	0.67445800
H	2.68038000	0.05203500	1.20323100
O	5.69901500	4.00054500	0.73301000
H	5.79663000	4.94399600	0.69359500
O	5.88166200	5.54305800	-1.49647900
H	6.51538600	5.89169900	-2.11127800
O	7.86067200	4.04286400	-2.92268300
H	7.42500800	3.71898800	-3.70159000
H	4.78329300	1.69701700	0.48769200
C	8.48631100	1.34827400	-1.74474000

H	9.09278800	1.73208600	-2.55697300
C	4.54927900	-3.01903800	0.19137300
O	8.28103800	-0.02968600	-1.89052400
H	8.91470400	-0.51234000	-1.36622700
H	8.99153800	1.57335400	-0.81015100
H	8.93727000	-3.04039300	-0.03513000
H	9.61735300	-2.77216900	1.55757600
O	9.79297700	-1.23819200	0.19476400
H	9.84159100	-0.53514500	0.83260200
H	5.44349500	-3.33254700	-0.32892600
H	3.93714600	-3.88800600	0.40918900
O	4.87571400	-2.35304700	1.38783300
H	4.04271800	3.89632000	-0.47447700
H	5.03325800	-0.47534000	-0.02094400
H	3.43943500	0.14877000	-2.48552300
O	0.52817000	-0.92054300	0.02085200
H	4.82384400	-1.93810300	3.83886200
O	4.34534400	1.61761000	-1.51386700
H	7.14935900	-1.41187600	0.43918100
H	1.54360600	-1.24572500	-1.71333600
Cl			
C	0.58071300	2.87987800	-0.76969900
C	-0.89828300	2.94061400	-0.36328100
C	-1.79506000	2.88037800	-1.61510800
C	-0.09918900	1.79066900	-2.88266900
C	0.78918000	1.65389100	-1.65193900
H	-1.08641900	2.06606800	0.24823000
H	0.84569900	3.75870800	-1.34605500
H	0.13921700	2.72213800	-3.39688200
H	-1.70219900	3.81993100	-2.15008400
O	-1.44010500	1.82322400	-2.44172300
O	1.41839500	2.77745000	0.34130300
C	0.01907400	0.65748600	-3.88666000
H	1.02706500	0.64515400	-4.27963300
H	-0.65453900	0.86835300	-4.71498300
H	1.48769100	3.65665000	0.70557700
O	-0.23471800	-0.59264500	-3.33454000
H	-1.15918100	-0.64704000	-3.10695000
C	-4.50001700	2.12405400	0.49999700
C	-5.26990700	0.95781900	1.13751700
C	-3.68208200	1.63338000	-0.70556300
H	-5.19767800	2.87276300	0.13640600
C	-6.04811300	0.19280700	0.06855900
H	-4.53932700	0.27987300	1.56023100
C	-4.61666200	0.89070800	-1.66782400
H	-2.90615700	0.95847600	-0.37622300
H	-6.85891100	0.80544700	-0.31916900
H	-5.39467900	1.58362000	-1.98888400
O	-5.20758100	-0.18809500	-0.96524500
O	-6.55874700	-0.94298100	0.66942400
O	-3.15234400	2.77779900	-1.33508200
C	-3.99963800	0.29603300	-2.92115600
H	-4.80542900	-0.11810300	-3.52136600
H	-3.51805200	1.07601500	-3.48563100
O	-3.03992400	-0.68162000	-2.65331100
H	-3.43260900	-1.49881800	-2.36637600
O	-3.61959100	2.69574200	1.41618600
H	-4.15571800	3.17313200	2.04767600
C	-7.60420300	-1.62431500	0.11241600
C	-7.98910900	-1.50644000	-1.21197400
C	-8.28510100	-2.47903500	0.96825200
C	-9.07608900	-2.24092500	-1.66761500
H	-7.44457800	-0.87861300	-1.88829300
C	-9.35880700	-3.20969200	0.49805300
H	-7.95973000	-2.56141100	1.98903800
C	-9.76532600	-3.09121600	-0.82379100
H	-9.37324800	-2.14721300	-2.69674800
H	-9.88164000	-3.87046600	1.16599200
H	-10.60236500	-3.65816200	-1.18802700
H	0.52308400	0.75673000	-1.12030800
N	-6.23567200	1.31114700	2.17951300
H	-6.76545100	0.52423700	2.48017200
C	-7.51642300	2.38313000	3.92393400
H	-7.03651900	2.33919400	4.89562200
H	-8.09541600	3.29546800	3.88178800
H	-8.17523100	1.53032600	3.81910600
N	-1.29496600	4.10513500	0.41464700
H	-2.21741000	4.00360700	0.77835000
C	-1.35457100	6.16072000	1.67326500
H	-0.91608500	6.18504400	2.66408500
H	-1.22651600	7.14229300	1.23447900
H	-2.41078900	5.93910700	1.75620200
C	-0.58456900	5.16083500	0.83593800
O	0.58859900	5.35493600	0.59975600
C	-6.43872400	2.45108900	2.86351500
O	-5.82657000	3.48109900	2.69304000
C	9.34213000	0.53820900	-0.25234600
C	8.17931300	1.46014400	0.10971500
C	6.90270700	0.65599400	0.33598500
C	8.14088700	-1.29172600	0.94481600

C	9.47800900	-0.58850700	0.76786500
H	9.16636700	0.09852200	-1.22505700
H	7.85997300	-1.76294900	0.01284500
H	9.77588000	-0.15618100	1.72356100
O	7.16052900	-0.33129600	1.30296900
C	5.19949200	-0.23655500	-1.18009400
C	4.30819200	0.98495100	-1.39938800
C	4.55519400	-1.23354900	-0.21094000
C	2.93082100	0.49956000	-1.84088500
H	4.73810300	1.60205100	-2.18468100
C	3.12249900	-1.51674600	-0.68549900
H	3.17715400	-2.05137100	-1.63110600
O	2.40426700	-0.32347500	-0.85895800
C	-1.15398100	-2.29909300	3.15470600
C	-0.87522100	-2.42914300	1.66873400
C	-2.12390800	-2.66522100	0.82856600
C	0.45733100	-3.79854600	0.23165500
C	-1.73452600	-2.96177800	-0.61522100
H	-2.65946600	-3.52504600	1.23121400
C	-0.72372000	-4.10160800	-0.68345900
H	1.09612100	-4.66983400	0.32553400
H	-1.30033900	-2.07357300	-1.04851700
O	-0.01066800	-3.55052000	1.52866000
O	-2.93695600	-1.52922400	0.90481100
H	-3.63776500	-1.61022500	0.26747000
O	-2.88905100	-3.25737800	-1.36741500
H	-3.10013300	-4.17663600	-1.24726300
O	-1.33664500	-5.31597700	-0.31343100
H	-1.32275100	-5.40170700	0.63169200
O	5.23889200	-2.45463700	-0.23410100
H	5.75010500	-2.58573500	0.55669800
O	4.19847200	1.70843900	-0.21115100
H	3.33712300	2.11805300	-0.14848900
O	8.50402700	2.19542200	1.26882300
H	8.26175500	1.69457700	2.03737900
O	10.54905000	1.24837400	-0.34064100
H	10.55795800	1.89454800	0.35642700
O	10.42462500	-1.53231400	0.35876800
H	11.20150900	-1.06638500	0.07380900
H	6.13274900	1.27869600	0.75323000
C	8.13366000	-2.33113800	2.05141300
H	8.73446000	-3.18059200	1.76527600
C	2.35536900	-2.37876500	0.29420400
O	6.82469700	-2.79889200	2.28033900
H	6.34032200	-2.10073700	2.70163400
H	8.54547200	-1.91282400	2.96581000
H	-1.69405600	-3.18015000	3.49842500
H	-1.77224300	-1.43177100	3.33282000
O	0.02741100	-2.12380900	3.87897000
H	0.60393200	-2.84965100	3.67979800
H	2.17378200	-1.82913100	1.21079400
H	2.93571000	-3.26568300	0.51852000
O	1.13531100	-2.73723100	-0.30891400
H	8.02049200	2.18153400	-0.67849800
H	4.52303100	-0.81293900	0.78712600
H	5.29284800	-0.75206300	-2.12912200
O	2.14506500	1.62999500	-2.01892200
H	-0.37640700	-4.22461400	-1.69862000
O	6.53551800	0.11153800	-0.88001400
H	-0.37071500	-1.53603100	1.32227800
H	2.99290100	-0.05014700	-2.77822800
C2			
C	0.62096100	2.88679600	-0.79047500
C	-0.86139700	2.95372700	-0.39643400
C	-1.74822400	2.87593000	-1.65448400
C	-0.04336600	1.76884700	-2.89413900
C	0.83553800	1.64934200	-1.65497000
H	-1.05412000	2.08802500	0.22622300
H	0.89096300	3.75813200	-1.37585300
H	0.19849100	2.69304200	-3.41970800
H	-1.65125400	3.80798300	-2.20160600
O	-1.38751100	1.80734300	-2.46323100
O	1.44991400	2.79753100	0.32809200
C	0.08173600	0.62149700	-3.88120500
H	1.09195400	0.60459500	-4.26824400
H	-0.58724200	0.82002100	-4.71631100
H	1.51714700	3.68108700	0.68197400
O	-0.17410100	-0.62090000	-3.31275900
H	-1.09983000	-0.67252500	-3.08970700
C	-4.47408100	2.15056700	0.44562000
C	-5.24285400	0.99036500	1.09453000
C	-3.64488100	1.64274400	-0.74457700
H	-5.17145300	2.89052500	0.06469600
C	-6.00914100	0.20084200	0.03494400
H	-4.50833700	0.32411800	1.52907300
C	-4.57101400	0.88528000	-1.70323900
H	-2.87237600	0.97145500	-0.39847000
H	-6.82785900	0.79671000	-0.36190400
H	-5.34872900	1.57172100	-2.03852700
O	-5.16263300	-0.18638700	-0.99052600

O	−6.50434700	−0.93321000	0.65329900
O	−3.10827400	2.77737000	−1.38434300
C	−3.94269400	0.27524400	−2.94333300
H	−4.74289900	−0.14823800	−3.54463200
H	−3.45736500	1.04811500	−3.51433500
O	−2.98460400	−0.69702200	−2.65315800
H	−3.37960300	−1.50506100	−2.34363700
O	−3.60359600	2.73835700	1.36051100
H	−4.14551300	3.22709600	1.97802200
C	−7.54705300	−1.63159400	0.11060300
C	−7.92753400	−1.54677900	−1.21751800
C	−8.22718600	−2.46791500	0.98468600
C	−9.01017700	−2.29604900	−1.65899000
H	−7.38262900	−0.93303100	−1.90640400
C	−9.29649800	−3.21398500	0.52849100
H	−7.90528800	−2.52450100	2.00834600
C	−9.69885300	−3.12863800	−0.79709200
H	−9.30433600	−2.22822600	−2.69099000
H	−9.81910200	−3.86057500	1.21030100
H	−10.53250500	−3.70737400	−1.15035800
H	0.56525000	0.75959200	−1.11308100
N	−6.21194800	1.35077800	2.12938900
H	−6.73156300	0.56369800	2.44703500
C	−7.50319700	2.44153900	3.85391300
H	−7.01878800	2.38233300	4.82273400
H	−8.07111700	3.36096700	3.82208200
H	−8.17215500	1.59708300	3.74521000
N	−1.26381400	4.12945600	0.36136900
H	−2.18923000	4.03381700	0.71893600
C	−1.33063100	6.19877400	1.59692500
H	−0.90975000	6.22251700	2.59543300
H	−1.18589200	7.17747500	1.15711200
H	−2.39005400	5.98671800	1.66214100
C	−0.55510800	5.18872600	0.77713400
O	0.62038200	5.37804400	0.54920100
C	−6.43058500	2.50364400	2.78825900
O	−5.83328400	3.53729900	2.59206100
C	9.37277900	0.50814800	−0.19594500
C	8.21014600	1.43144400	0.16310100
C	6.93153600	0.62871900	0.38265900
C	8.16375700	−1.32161600	0.99447700
C	9.50275400	−0.62046400	0.82294500
H	9.20003100	0.07035100	−1.17002600
H	7.88525900	−1.79164500	0.06115900
H	9.79820100	−0.19018400	1.78032900
O	7.18362700	−0.35980200	1.34991100
C	5.23364500	−0.25745400	−1.14232400
C	4.35120900	0.96814100	−1.37480100
C	4.57869700	−1.24682800	−0.17211000
C	2.97337300	0.48742600	−1.81914600
H	4.78925500	1.57671600	−2.16223200
C	3.14529700	−1.52135800	−0.64924800
H	3.19840200	−2.06188600	−1.59163900
O	2.43799400	−0.32334500	−0.83220000
C	−1.15242100	−2.51543300	3.23853900
C	−0.86566100	−2.55212100	1.74190800
C	−2.11218100	−2.77193100	0.88696700
C	0.47940300	−3.80557500	0.21998300
C	−1.73036300	−2.96601500	−0.57388300
H	−2.61481900	−3.67153400	1.23999700
C	−0.69558200	−4.07736600	−0.71274800
H	1.13420100	−4.66866000	0.26222900
H	−1.32080300	−2.04376800	−0.95735500
O	0.01524500	−3.64147400	1.53013900
O	−2.97468000	−1.67313800	1.03726500
H	−3.68342200	−1.74897200	0.40732500
O	−2.88639400	−3.24490700	−1.33061000
H	−3.06362400	−4.17768300	−1.27683000
O	−1.28517600	−5.32358500	−0.41799200
H	−1.24825700	−5.47835800	0.51778500
O	5.25330400	−2.47299100	−0.18985800
H	5.76489300	−2.60472100	0.60059900
O	4.23927400	1.70131000	−0.19254000
H	3.38173000	2.12022300	−0.14117200
O	8.53119900	2.16410000	1.32490600
H	8.28643500	1.66142600	2.09146600
O	10.58124100	1.21638200	−0.27844000
H	10.58880900	1.86114000	0.41994900
O	10.44900600	−1.56543700	0.41570400
H	11.22799700	−1.10062400	0.13472400
H	6.16040300	1.25217300	0.79672500
C	8.15138800	−2.36217000	2.09986600
H	8.75334100	−3.21129400	1.81516600
C	2.36676300	−2.37073400	0.33281300
O	6.84161400	−2.83013400	2.32271400
H	6.35652400	−2.13520000	2.74830000
H	8.55962900	−1.94498200	3.01640000
H	−0.21051300	−2.53433500	3.76918500
H	−1.70713700	−3.40949600	3.52052800
O	−1.82115400	−1.35372100	3.62588800

H	-2.56103900	-1.21522900	3.04873000
H	2.20053200	-1.81943000	1.25131600
H	2.93148600	-3.26781900	0.55470800
O	1.13626200	-2.70352200	-0.26326500
H	8.05562300	2.15454900	-0.62437900
H	4.54695400	-0.82312300	0.82470300
H	5.33014700	-0.77845400	-2.08801400
O	2.19442900	1.62030100	-2.01018300
H	-0.34630600	-4.12985000	-1.73328700
O	6.56940300	0.08559700	-0.83539600
H	-0.39199600	-1.62192400	1.45217700
H	3.03667600	-0.07110900	-2.75116500
C3			
C	1.01131400	2.25630600	-0.66104000
C	-0.46951300	2.55277300	-0.38253000
C	-1.26994200	2.53818100	-1.70050600
C	0.33509000	1.14558600	-2.77116400
C	1.10471400	0.96774200	-1.46983400
H	-0.82741400	1.75531200	0.25833800
H	1.44949300	3.05688400	-1.24603100
H	0.73663600	1.99847300	-3.31855600
H	-0.99834000	3.41706500	-2.27588400
O	-1.01439600	1.38917300	-2.43623200
O	1.73641700	2.08515100	0.51994800
C	0.36401500	-0.06281700	-3.69057400
H	1.38312300	-0.22852500	-4.01369300
H	-0.22868600	0.16823600	-4.57341000
H	1.89325600	2.96162500	0.86541000
O	-0.07755200	-1.22943800	-3.07517900
H	-1.01099800	-1.15125800	-2.89580900
C	-4.22963600	2.36447400	0.20124400
C	-5.20946400	1.38068500	0.85781100
C	-3.38545700	1.65207800	-0.86823400
H	-4.77941500	3.15843600	-0.29536700
C	-5.98933400	0.61920000	-0.21209300
H	-4.62152500	0.65941900	1.41103400
C	-4.32841200	0.94731200	-1.85127000
H	-2.74591600	0.91594200	-0.40487800
H	-6.67010700	1.28924700	-0.73222900
H	-4.97205000	1.70071400	-2.30576900
O	-5.12177500	0.03026000	-1.11838300
O	-6.70313800	-0.37437500	0.43219000
O	-2.64473100	2.65055000	-1.53218100
C	-3.69828800	0.15447400	-2.98225500
H	-4.50199100	-0.21422000	-3.61398600

H	-3.07266600	0.80532600	-3.56829000
O	-2.90310600	-0.90424100	-2.53825500
H	-3.42483400	-1.63339000	-2.22156500
O	-3.36252200	2.90406500	1.14893500
H	-3.87814900	3.51540900	1.67275300
C	-7.77996300	-0.96718400	-0.16475700
C	-8.02741600	-0.93442900	-1.52629100
C	-8.64492600	-1.63605100	0.69029400
C	-9.16144800	-1.56415900	-2.02230100
H	-7.34430000	-0.45343200	-2.19717700
C	-9.76405800	-2.26518600	0.18086500
H	-8.42489000	-1.65643300	1.74187600
C	-10.03291900	-2.22881600	-1.18037200
H	-9.35157700	-1.53672300	-3.08027100
H	-10.42983900	-2.78197700	0.84853500
H	-10.90526600	-2.71632700	-1.57543000
H	0.67689600	0.14873800	-0.91873900
N	-6.20368600	1.96244000	1.76111500
H	-6.86176500	1.28662200	2.07808700
C	-7.47043700	3.36823700	3.26181100
H	-7.08761600	3.36379700	4.27662800
H	-7.91098400	4.33864700	3.07858800
H	-8.22984800	2.60196200	3.16995000
N	-0.74970800	3.81423600	0.28796900
H	-1.70924400	3.88248600	0.55009900
C	-0.60495000	5.91783000	1.45740500
H	-0.29313600	5.92060700	2.49538600
H	-0.25583300	6.83903200	1.00822700
H	-1.68565500	5.88120300	1.40976700
C	0.07647300	4.75988300	0.75876200
O	1.28466400	4.74570300	0.66384100
C	-6.30572600	3.18480400	2.31293600
O	-5.54440400	4.10328700	2.11004100
C	9.09495100	-0.40396400	1.17425400
C	8.80276300	-1.33310300	-0.00102200
C	7.50414600	-0.93342600	-0.69510300
C	7.66876000	1.30973700	0.06251000
C	9.00387200	1.06155000	0.75391000
H	8.36615000	-0.57127900	1.95712500
H	6.87140700	1.10822700	0.76467900
H	9.81131900	1.28052700	0.05417600
O	7.56316500	0.41027400	-1.03547100
C	5.16636900	-1.39260200	-0.44448400
C	4.49134700	-0.09585800	-0.90103500
C	4.31605300	-2.11406700	0.59421200

C	3.08094400	−0.45322900	−1.37077100
H	5.03820200	0.34088200	−1.72744900
C	2.91044000	−2.31715900	0.03623900
H	2.96276600	−2.97193800	−0.83071700
O	2.38618200	−1.07077000	−0.33947600
C	−1.72746600	−2.08946600	3.53303000
C	−1.34908100	−2.42513500	2.10238800
C	−2.54636000	−2.60123200	1.17681900
C	−0.10177500	−4.10915300	0.95101400
C	−2.08514400	−3.10916200	−0.18464400
H	−3.22256600	−3.33568700	1.61453600
C	−1.23553900	−4.36687500	−0.03532400
H	0.40347800	−5.03681200	1.19641700
H	−1.50137600	−2.33848300	−0.66508500
O	−0.63236400	−3.65317100	2.16353900
O	−3.20347200	−1.37230600	1.05726700
H	−3.85515100	−1.43709300	0.36801600
O	−3.20319700	−3.34225900	−1.00970000
H	−3.54889000	−4.20592900	−0.81328900
O	−2.03403900	−5.44341800	0.40074700
H	−2.11095200	−5.41745700	1.34638600
O	4.84859500	−3.37484100	0.89029900
H	5.73991600	−3.25614400	1.19144500
O	4.43659400	0.81728400	0.15845900
H	3.55659900	1.18577800	0.23769900
O	9.86407000	−1.28219300	−0.92711000
H	9.74916300	−0.52762400	−1.49230800
O	10.35281100	−0.67465600	1.73292900
H	10.95903000	−0.84298200	1.02058700
O	9.10150600	1.89735700	1.86961300
H	9.84463200	1.61315900	2.38771600
H	7.41467200	−1.47082200	−1.63193900
C	7.49777200	2.71695400	−0.48006100
H	7.63548700	3.41673700	0.33845400
C	1.96261800	−2.94186300	1.03757800
O	6.26137400	2.89571400	−1.10246300
H	5.57900700	2.49443400	−0.57570600
H	8.25765400	2.91725200	−1.22626900
H	−2.40042200	−2.85521200	3.91633700
H	−2.24390500	−1.14138700	3.55754300
O	−0.59630700	−1.96733700	4.34253900
H	−0.10193100	−2.77409700	4.28298600
H	1.79138500	−2.26239600	1.86408100
H	2.39692700	−3.85944200	1.41702200
O	0.75600100	−3.21327400	0.36609800
H	8.74690500	−2.35722200	0.34077400
H	4.26444800	−1.49580600	1.48428400
H	5.26767000	−2.04787300	−1.30883300
O	2.46555900	0.73369900	−1.73296300
H	−0.82750700	−4.65089500	−0.99410400
O	6.44045400	−1.23240400	0.13894800
H	−0.70397300	−1.64715200	1.71395100
H	3.11882100	−1.12278200	−2.22807600
C4			
C	1.03214800	2.21484200	−0.68086600
C	−0.45208100	2.51160700	−0.41858700
C	−1.23998300	2.47054300	−1.74365300
C	0.37395000	1.05905500	−2.77382300
C	1.13672700	0.91183400	−1.46483100
H	−0.81218200	1.72456000	0.23405300
H	1.47111900	3.00638500	−1.27759200
H	0.77614200	1.90090100	−3.33752900
H	−0.96109100	3.33679900	−2.33441100
O	−0.97816900	1.30610500	−2.45217600
O	1.75028700	2.06733600	0.50730400
C	0.41161400	−0.16937400	−3.66605500
H	1.43242400	−0.33703300	−3.98266700
H	−0.17964600	0.03897600	−4.55551400
H	1.89983900	2.95022100	0.83940200
O	−0.02536800	−1.32459600	−3.02617400
H	−0.95856600	−1.24594700	−2.84585300
C	−4.23428200	2.36372000	0.11480100
C	−5.23169200	1.40735700	0.78574900
C	−3.36959700	1.61319200	−0.91159400
H	−4.77019200	3.14179100	−0.42020000
C	−5.98369300	0.59437900	−0.26571400
H	−4.65554100	0.71135900	1.38226400
C	−4.29424700	0.87068900	−1.88393600
H	−2.73862400	0.89482900	−0.40950200
H	−6.66116700	1.23456800	−0.82595300
H	−4.93491300	1.60423600	−2.37366200
O	−5.09323900	−0.02346100	−1.12863800
O	−6.69889600	−0.37810000	0.40882900
O	−2.61680200	2.58889900	−1.59411200
C	−3.64534900	0.04092000	−2.97681100
H	−4.43902700	−0.35567600	−3.60436700
H	−3.01610000	0.67350300	−3.57862400
O	−2.85037200	−0.99542000	−2.48325800
H	−3.37280100	−1.71271800	−2.14129400
O	−3.38510500	2.93052900	1.06241100

H	-3.90666600	3.56351500	1.55355000	O	2.42759100	-1.09496500	-0.28714600
C	-7.76112900	-1.00597300	-0.17977400	C	-1.67085100	-2.06703400	3.65765300
C	-7.98156400	-1.03920500	-1.54583700	C	-1.29267000	-2.41804300	2.22332100
C	-8.63844900	-1.63939300	0.68931900	C	-2.49647600	-2.61335200	1.30380200
C	-9.10176900	-1.69952400	-2.03335600	C	-0.05673100	-4.10226500	1.07099900
H	-7.28810000	-0.58562900	-2.22518300	C	-2.04843900	-3.11720500	-0.06147700
C	-9.74349300	-2.29981000	0.18860700	H	-3.15332300	-3.35699400	1.75333900
H	-8.43929000	-1.60840200	1.74480400	C	-1.19174500	-4.37003700	0.08863200
C	-9.98554200	-2.32968300	-1.17777100	H	0.45263100	-5.02664400	1.31945800
H	-9.27124100	-1.72351700	-3.09489400	H	-1.47476300	-2.34554200	-0.55234400
H	-10.41919000	-2.78905300	0.86694300	O	-0.57883500	-3.64102100	2.28362700
H	-10.84695300	-2.84141300	-1.56610400	O	-3.18961000	-1.39733600	1.18756900
H	0.71110300	0.10122400	-0.89972000	H	-3.84835200	-1.48010500	0.50618800
N	-6.24637100	2.02373100	1.64042800	O	-3.17644700	-3.36116700	-0.87013700
H	-6.91388900	1.36196500	1.96748200	H	-3.50602000	-4.23124900	-0.67349900
C	-7.53614200	3.48419400	3.06696100	O	-1.98576200	-5.44746600	0.53080700
H	-7.18063300	3.46557500	4.09170300	H	-2.04161100	-5.43245500	1.47826900
H	-7.94248300	4.46730700	2.87408700	O	4.89681200	-3.37479900	0.97325900
H	-8.31466900	2.74024400	2.95334500	H	5.78754100	-3.25033300	1.27375000
N	-0.74117800	3.78445900	0.22592800	O	4.47109400	0.80480700	0.17176600
H	-1.70334300	3.85634800	0.47769200	H	3.58996100	1.17193700	0.24323400
C	-0.61213400	5.90359300	1.36805400	O	9.90476300	-1.29796900	-0.88495400
H	-0.34588900	5.89229300	2.41883500	H	9.78676400	-0.55394000	-1.46333000
H	-0.22882900	6.82205100	0.94236300	O	10.39524100	-0.64180200	1.76322800
H	-1.69031700	5.88457200	1.27471900	H	11.00098300	-0.82120200	1.05318600
C	0.07875000	4.73919400	0.68983700	O	9.13613200	1.92832500	1.85607800
O	1.28772700	4.72652100	0.60609100	H	9.88096100	1.65578900	2.37799300
C	-6.35348600	3.26569700	2.14849600	H	7.45487500	-1.50653600	-1.58277000
O	-5.58294600	4.17206100	1.92976900	C	7.52725500	2.70135500	-0.50582100
C	9.13581700	-0.38495300	1.20158100	H	7.66441200	3.41604900	0.29979800
C	8.84488000	-1.33567800	0.04338900	C	2.01081900	-2.93764200	1.12915600
C	7.54408500	-0.95230200	-0.65577600	O	6.28931700	2.86563100	-1.12921100
C	7.70301000	1.30451700	0.06148300	H	5.60883800	2.47273100	-0.59379700
C	9.03971000	1.07259500	0.75545600	H	8.28530100	2.89032800	-1.25682000
H	8.40857200	-0.54058200	1.98830000	H	-0.78379000	-2.13390700	4.27217800
H	6.90717200	1.11328400	0.76825100	H	-2.38649000	-2.79843200	4.03127700
H	9.84562800	1.28160200	0.05093500	O	-2.15775500	-0.76529000	3.77725900
O	7.59858900	0.38534700	-1.02012400	H	-2.82423300	-0.62026200	3.11828900
C	5.20798000	-1.41348100	-0.39344100	H	1.85355700	-2.24528000	1.94757300
C	4.52856900	-0.12693300	-0.87159200	H	2.44145200	-3.85227100	1.51904800
C	4.36175200	-2.11962500	0.65907100	O	0.79562400	-3.20834100	0.47371600
C	3.11872600	-0.49711300	-1.33220800	H	8.79258700	-2.35372900	0.40337000
H	5.07302800	0.29714500	-1.70610500	H	4.31278600	-1.48724000	1.53935600
C	2.95388000	-2.33367900	0.11069400	H	5.30975300	-2.08317800	-1.24658600
H	3.00251600	-3.00521400	-0.74358500	O	2.50033600	0.68121700	-1.71611200

H	-0.78493900	-4.65512000	-0.87034600
O	6.48252100	-1.23946900	0.18505700
H	-0.66132800	-1.63253600	1.82596000
H	3.15648900	-1.18377300	-2.17587800
C5			
C	0.12771400	-1.74425700	1.22332100
C	1.57864900	-1.36501300	1.55013000
C	2.50882100	-2.46685700	1.01841100
C	0.97831600	-3.04403300	-0.72210800
C	0.01366100	-1.94756000	-0.28439900
H	1.79677600	-0.44186300	1.02601500
H	-0.12836600	-2.67861300	1.70954500
H	0.69613900	-3.97791600	-0.23745900
H	2.32997700	-3.37631500	1.58305700
O	2.28784700	-2.68367400	-0.33412400
O	-0.77645400	-0.75346600	1.60771700
C	1.02014700	-3.26746700	-2.22481400
H	0.03300400	-3.54790800	-2.56666000
H	1.68974500	-4.09921100	-2.43110700
H	-0.89232200	-0.85043100	2.55006400
O	1.40524800	-2.12640700	-2.92386200
H	2.33662500	-1.98519000	-2.78310400
C	5.20238700	-0.26017300	1.41201000
C	6.15822400	0.67762400	0.65959500
C	4.52952600	-1.23441000	0.43555100
H	5.76259900	-0.84801100	2.13321600
C	7.11048200	-0.14367400	-0.20671100
H	5.56226500	1.28963800	-0.00925100
C	5.58405200	-1.93360300	-0.42893500
H	3.84539900	-0.69201100	-0.20129100
H	7.76687800	-0.74233000	0.42137700
H	6.19554000	-2.55967600	0.21965000
O	6.39314300	-0.96182800	-1.06158700
O	7.85404900	0.74619700	-0.95685600
O	3.86239000	-2.20852800	1.20344700
C	5.04789200	-2.79224700	-1.56086600
H	5.88878400	-3.29628800	-2.02906900
H	4.36600300	-3.53135200	-1.18048100
O	4.35372400	-2.02874500	-2.50964400
H	4.94435200	-1.37882000	-2.86889200
O	4.18976600	0.45280500	2.04988000
H	4.59646800	0.91609300	2.78087600
C	9.02639900	0.36332900	-1.54575700
C	9.40289300	-0.95411200	-1.74151400
C	9.85337900	1.39889200	-1.95847400

C	10.62650200	-1.22530200	-2.34005000
H	8.75410700	-1.75951700	-1.46126900
C	11.06334000	1.11319500	-2.55987900
H	9.53322500	2.41325600	-1.80633100
C	11.46096700	-0.20304700	-2.75019200
H	10.91671600	-2.24965700	-2.49052300
H	11.69881200	1.91998500	-2.87815000
H	12.40361300	-0.42423800	-3.21618000
H	0.24734700	-1.03320300	-0.80221400
N	6.98692500	1.55679600	1.48228300
H	7.66226400	2.04843100	0.94157200
C	7.97498200	2.86993300	3.25254800
H	7.48004700	3.82232400	3.40843000
H	8.36811200	2.54175800	4.20496500
H	8.78900600	3.00778600	2.55218200
N	1.88193200	-1.13779500	2.95389800
H	2.77764300	-0.72004000	3.07291100
C	1.80109500	-0.94614300	5.35839800
H	1.24781200	-0.13762900	5.82114000
H	1.73676900	-1.80464000	6.01537700
H	2.83790200	-0.65398600	5.25082500
C	1.12535000	-1.29810300	4.04986100
O	-0.02146200	-1.68823100	4.05149900
C	6.94068300	1.86654100	2.79107100
O	6.14398800	1.41543100	3.58203100
C	-8.42382900	-0.87202000	0.16426300
C	-8.07772500	-1.36890800	-1.23695200
C	-6.63391900	-1.85825300	-1.29653100
C	-6.57831400	-2.29846800	1.03355000
C	-8.03623200	-1.89996300	1.22550000
H	-7.87845500	0.04052700	0.36813900
H	-5.96086700	-1.41911900	1.15603200
H	-8.66355000	-2.78459900	1.10888800
O	-6.42746600	-2.79606500	-0.29044100
C	-4.48091500	-0.90588200	-1.70188900
C	-3.54057400	-1.71890500	-0.80546400
C	-3.94637600	0.50587400	-1.89774000
C	-2.13749900	-1.64926800	-1.40436100
H	-3.85534600	-2.75430900	-0.77337100
C	-2.49621300	0.45182300	-2.37293300
H	-2.47993000	0.00815500	-3.36903100
O	-1.72218600	-0.32804800	-1.49978800
C	-5.40518600	4.59883000	-1.02664600
C	-4.05620100	4.12873800	-0.52228300
C	-3.58332700	4.80151200	0.75732200

C	-1.83663800	3.86946100	-1.35247500
C	-2.18847700	4.28865600	1.10615800
H	-3.53700900	5.87955300	0.59745200
C	-1.23839400	4.45395900	-0.07697400
H	-1.25243600	4.20263200	-2.20184300
H	-2.26642600	3.23951400	1.36115900
O	-3.11681300	4.39915200	-1.55640800
O	-4.49413200	4.51047300	1.77443800
H	-4.09387700	4.73235600	2.60656500
O	-1.70167000	4.94684800	2.24437400
H	-1.31951400	5.77103100	1.96555900
O	-0.95106300	5.82228600	-0.27139000
H	-1.65102200	6.22388300	-0.77104300
O	-4.67496000	1.19190800	-2.87557000
H	-5.38616400	1.67782000	-2.47635600
O	-3.54136900	-1.19538200	0.49410400
H	-2.64678700	-1.03629700	0.79225600
O	-8.94473800	-2.42229000	-1.59663100
H	-8.61958300	-3.23277500	-1.22358200
O	-9.78753100	-0.55781800	0.26841000
H	-10.27547900	-1.21851100	-0.20964800
O	-8.20158000	-1.36663300	2.50712100
H	-9.05258300	-0.94710900	2.54304300
H	-6.47177800	-2.38786900	-2.22800900
C	-6.08936900	-3.37200800	1.98804800
H	-6.26993600	-3.03414300	3.00382400
C	-1.85448100	1.81648100	-2.50229300
O	-4.74241600	-3.68265600	1.78667400
H	-4.24658700	-2.88040400	1.66509400
H	-6.64988200	-4.28615200	1.83164900
H	-5.36803600	5.66316600	-1.24617100
H	-6.15502200	4.43096700	-0.26886300
O	-5.79307000	3.87210400	-2.16302800
H	-5.12338000	3.98009400	-2.82678500
H	-2.40970300	2.38011500	-3.23924200
H	-0.83153200	1.69293600	-2.84169500
O	-1.84978100	2.49654200	-1.26678500
H	-8.23529600	-0.58175400	-1.96017700
H	-3.98479300	1.01039900	-0.94359400
H	-4.53229100	-1.38808200	-2.67730500
O	-1.30179100	-2.36099200	-0.55811200
H	-0.29605000	3.97142000	0.13689100
O	-5.78783600	-0.77180200	-1.19917600
H	-4.10936100	3.06371200	-0.34991900
H	-2.12401400	-2.10109700	-2.39571100

C6

C	0.10367500	-0.28231400	0.05883800
C	1.40240000	0.49842700	-0.18515000
C	2.54636300	-0.51110400	-0.23777000
C	1.09945400	-2.20501900	-1.15799600
C	-0.09648300	-1.25530200	-1.10314600
H	1.35733700	0.99016500	-1.14739600
H	0.20990900	-0.86495500	0.96864700
H	1.11974300	-2.80842700	-0.25268700
H	2.62318200	-1.04038100	0.70980600
O	2.29354400	-1.44533900	-1.25651700
O	-0.99980200	0.53401400	0.30118700
C	1.08262200	-3.12680200	-2.36494700
H	0.19351000	-3.73910300	-2.33563800
H	1.94719300	-3.78472000	-2.31615400
H	-1.17661500	1.10476300	-0.43922400
O	1.05711100	-2.41894900	-3.56962300
H	1.83103800	-1.87281000	-3.61834800
C	5.66508100	-1.33358800	-0.70588500
C	7.00760400	-1.65322400	-0.03871100
C	4.90467700	-0.30804200	0.12430500
H	5.83471100	-0.91910300	-1.69245900
C	7.76515300	-0.39153500	0.38775900
H	6.78934800	-2.19000400	0.87586500
C	5.78865000	0.90674100	0.37566300
H	4.65198900	-0.74602800	1.08198000
H	8.16829900	0.13234600	-0.46770900
H	6.10277900	1.33803700	-0.57386100
O	6.90972600	0.45981700	1.10034600
O	8.77307300	-0.79455300	1.23638200
O	3.72946000	0.11983500	-0.52675100
C	5.11297800	1.99556800	1.18773900
H	5.85083600	2.75099600	1.43894300
H	4.33986300	2.45487000	0.59315800
O	4.49074500	1.50283000	2.34685700
H	5.15527900	1.16368400	2.93355500
O	4.96452300	-2.54268700	-0.79344000
H	4.14551700	-2.39059300	-1.25058900
C	9.86382100	-0.00681000	1.44627300
C	9.88465300	1.36194000	1.23688300
C	10.99603400	-0.65951200	1.91544500
C	11.05425100	2.06818400	1.48444100
H	9.00531300	1.87805100	0.90662100
C	12.14892600	0.05770900	2.16723300
H	10.95252800	-1.72125800	2.07240400

C	12.18774300	1.42809600	1.94869400
H	11.06693800	3.13054800	1.31740100
H	13.02198600	-0.45511500	2.52967400
H	13.08692300	1.98453500	2.14104400
H	-0.15677100	-0.71969000	-2.03767600
N	7.82932500	-2.53575900	-0.84064500
H	7.89682500	-3.47803200	-0.53488000
C	8.98397800	-3.37040700	-2.79877200
H	8.23315700	-3.99613800	-3.27162400
H	9.62381900	-2.95889400	-3.56551400
H	9.56866800	-3.98878700	-2.12737100
N	1.63060100	1.51431100	0.81145500
H	2.20746800	1.31857100	1.60011600
C	1.40946400	3.74550700	1.75973700
H	1.88837000	4.61797000	1.33157800
H	0.45810200	4.06254600	2.17025000
H	2.03313800	3.34341100	2.54769400
C	1.13376000	2.75905700	0.65019400
O	0.50069900	3.07455200	-0.32818500
C	8.29633900	-2.23415400	-2.07426600
O	8.16705700	-1.14570000	-2.57158500
C	-8.06481000	-2.13152400	1.73362000
C	-7.95840100	-2.78956500	0.36024200
C	-6.50003200	-2.91284500	-0.07007400
C	-5.72721500	-2.86309600	2.17334900
C	-7.13897000	-2.80761300	2.74318000
H	-7.77388900	-1.09154600	1.65828800
H	-5.38479400	-1.85150600	2.00330500
H	-7.48957500	-3.82481100	2.92190700
O	-5.77895900	-3.55512400	0.93143300
C	-4.89267200	-1.56275900	-1.20772900
C	-3.57188800	-1.96667300	-0.54227500
C	-4.81767300	-0.11588100	-1.67216100
C	-2.44017800	-1.71929200	-1.53541800
H	-3.58484100	-3.01694400	-0.28584000
C	-3.59208700	0.08830900	-2.56227900
H	-3.75638000	-0.45039400	-3.49504600
O	-2.42494400	-0.38695000	-1.93803000
C	-6.71348800	3.57725600	-0.37035200
C	-5.20322800	3.46508600	-0.32615800
C	-4.52178700	4.32453900	0.72733800
C	-3.36477200	3.63332500	-1.83392900
C	-3.00965100	4.15331900	0.60520100
H	-4.77795100	5.37070200	0.55734700
C	-2.54439200	4.42802700	-0.82314900

H	-3.17156700	4.02063000	-2.82630600
H	-2.75917300	3.13625800	0.87872800
O	-4.73020700	3.84768300	-1.61203600
O	-4.97650200	3.92226100	1.98491700
H	-4.40571700	4.30024900	2.64302300
O	-2.35577100	4.98975100	1.52322200
H	-2.24237200	5.84006100	1.11377800
O	-2.67255800	5.80690500	-1.10513400
H	-3.57316600	6.00482900	-1.32888700
O	-5.93790200	0.24034400	-2.42717100
H	-6.57733600	0.67236600	-1.87295600
O	-3.37346600	-1.23038100	0.63395200
H	-2.62655000	-0.63999000	0.55351700
O	-8.54548900	-4.07157300	0.39875300
H	-7.91910000	-4.69086100	0.75346000
O	-9.38693900	-2.14646500	2.20219100
H	-9.76959000	-2.98482800	1.96998700
O	-7.11991900	-2.09373600	3.94475400
H	-8.01749300	-1.89795900	4.18487600
H	-6.43452300	-3.55865300	-0.93786400
C	-4.71968200	-3.57457400	3.05766700
H	-4.73099400	-3.10212300	4.03495800
C	-3.35073800	1.54135900	-2.91857700
O	-3.43539600	-3.57121800	2.50634800
H	-3.24904600	-2.71521300	2.13566800
H	-5.00688200	-4.61209300	3.18103500
H	-7.00614200	4.60546500	-0.56698000
H	-7.12942800	3.27767800	0.57914700
O	-7.24396300	2.71842800	-1.34814100
H	-6.89233500	2.98008800	-2.18973600
H	-4.24068600	1.92808100	-3.39430700
H	-2.51822800	1.60685700	-3.61114100
O	-3.04137300	2.29047500	-1.76190500
H	-8.51514900	-2.21935700	-0.36927900
H	-4.72306600	0.50182400	-0.79228800
H	-5.05979500	-2.19830600	-2.07675500
O	-1.24989200	-2.02564300	-0.88993900
H	-1.49792000	4.18950200	-0.91980500
O	-6.00943400	-1.65463100	-0.36021500
H	-4.94666100	2.43327100	-0.13753400
H	-2.55373000	-2.35602300	-2.41128000
